# Chromatin structure influences rate and spectrum of spontaneous mutations in *Neurospora crassa*

**DOI:** 10.1101/gr.276992.122

**Published:** 2023-04

**Authors:** Mariana Villalba de la Peña, Pauliina A.M. Summanen, Martta Liukkonen, Ilkka Kronholm

**Affiliations:** Department of Biological and Environmental Science, University of Jyväskylä, FI-40014 Jyväskylä, Finland

## Abstract

Although mutation rates have been extensively studied, variation in mutation rates throughout the genome is poorly understood. To understand patterns of genetic variation, it is important to understand how mutation rates vary. Chromatin modifications may be an important factor in determining variation in mutation rates in eukaryotic genomes. To study variation in mutation rates, we performed a mutation accumulation (MA) experiment in the filamentous fungus *Neurospora crassa* and sequenced the genomes of the 40 MA lines that had been propagated asexually for approximately 1015 [1003,1026] mitoses. We detected 1322 mutations in total and observed that the mutation rate was higher in regions of low GC, in domains of H3K9 trimethylation, in centromeric regions, and in domains of H3K27 trimethylation. The rate of single-nucleotide mutations in euchromatin was $2.46\;[2.19,2.77]\times 10^{-10}$. In contrast, the mutation rate in H3K9me3 domains was 10-fold higher: 2.43 $[2.25,2.62]\times 10^{-9}$. We also observed that the spectrum of single-nucleotide mutations was different between H3K9me3 and euchromatic domains. Our statistical model of mutation rate variation predicted a moderate amount of extant genetic variation, suggesting that the mutation rate is an important factor in determining levels of natural genetic variation. Furthermore, we characterized mutation rates of structural variants, complex mutations, and the effect of local sequence context on the mutation rate. Our study highlights that chromatin modifications are associated with mutation rates, and accurate evolutionary inferences should take variation in mutation rates across the genome into account.

New mutations are the source of all genetic diversity, and evolutionary change ultimately depends on the input of new mutations into the population. However, organisms also pay a substantial cost for their ability to evolve, as deleterious mutations are more common than beneficial mutations (Eyre-Walker and Keightley 2007), and mutations can lead to adverse outcomes, such as a decline in fitness, hereditary diseases, and cancer. Therefore, knowledge of the rates and spectrum of spontaneous mutations is fundamental to our understanding of evolution and certain aspects of medicine.

Spontaneous mutations are rare events that were previously difficult to study. However, new sequencing technologies have made it possible to capture a large number of spontaneous mutations for analysis (Katju and Bergthorsson 2018). Mutation rates can now be estimated through direct observations by sequencing mutation accumulation (MA) lines or parent–offspring trios (Ossowski et al. 2010; Ness et al. 2012, 2015; Keightley et al. 2014, 2015; Zhu et al. 2014; Sung et al. 2015; Keith et al. 2016; Wang et al. 2020). These studies have produced highly precise estimates of the rate and spectrum of spontaneous mutations.

The process of mutation is stochastic, but not all mutations are equally likely. Although this has been appreciated for a long time for certain classes of mutations, such as transitions and transversions, there is also variation in mutation rates that seems to depend on the structural features of the genome, such as the organization of chromatin (Makova and Hardison 2015). In particular, the positioning of nucleosomes (Tolstorukov et al. 2011; Chen et al. 2012; Li and Luscombe 2020) and the chromatin structure have a strong influence on mutation rates (Schuster-Böckler and Lehner 2012; Polak et al. 2015; Weng et al. 2019; Monroe et al. 2022). Chromatin structure is associated with chemical modifications of histone H3. In particular, the methylation status of certain lysine residues, such as H3K9 and H3K27 methylation, is associated with closed and silenced chromatin, called heterochromatin, whereas the methylation of H3K36 is associated with open actively transcribed chromatin, known as euchromatin (Kouzarides 2007). Heterochromatin appears to have higher mutation rates than euchromatin (Makova and Hardison 2015). In addition, the local sequence context, such as GC-content, also has a strong effect on mutation rates (Makova and Hardison 2015; Ness et al. 2015; Sung et al. 2015). Although we know that the chromatin structure can shape mutation rates, most data come from humans and a few model species.

Furthermore, to what extent variation in mutation rates determines patterns of observed genetic diversity, along with other evolutionary mechanisms, is understood mostly from population genetic data, rather than from direct observations of mutation, with a few exceptions (e.g., Monroe et al. 2022). There are some tests for selection, such as *d*_N_/*d*_S_ ratios, which are not affected by the mutation rate. However, tests based on the site frequency spectrum to infer mutational effects or demography can benefit from data about the mutation rate if it is available (Keightley and Eyre-Walker 2007), especially if the goal is to examine different categories of genes or regions of the genome. Furthermore, in order to understand how different evolutionary forces, such as background selection, gene conversion, demographic processes, and adaptive evolution, jointly shape patterns of diversity across the genome, obtaining mutation rate estimates will allow us to parameterize population genetic models (Campos et al. 2017; Castellano et al. 2020; Johri et al. 2020). Ultimately, taking all possible effects into account will allow us to make better estimates of how natural selection shapes genetic diversity.

To examine patterns of variation in mutation rates, we performed a MA experiment (Halligan and Keightley 2009) in the filamentous fungus *Neurospora crassa*. In MA experiments, an ancestor is split into multiple lines, and these lines are bottlenecked every generation, which minimizes the efficiency of natural selection (Fig. 1A). This way, even deleterious mutations can accumulate in these lines (Halligan and Keightley 2009). We sequenced the genomes of these MA lines using short-read sequencing. *N. crassa* is a filamentous fungus with a facultative sexual cycle, producing both asexual and sexual spores. It has a small genome of 42 Mb, and the vegetative mycelium is haploid. Furthermore, *N. crassa* has a genome defense mechanism called repeat-induced point mutation (RIP), which detects duplicated regions of the genome in premeiotic cells and induces C → T transitions in the duplicated sequences (Selker 1990). Because RIP does not induce the same specific mutations in both copies, large repeated arrays are seldom perfect in *N. crassa* as the duplicated sequences diverge from each other owing to RIP. As the sequences diverge, the efficiency of RIP decreases (Cambareri et al. 1991). The existence of imperfect repeats makes it possible to use short-read sequencing to sequence repetitive regions, for example, centromeric regions (Smith et al. 2011), where read mapping is normally difficult in plants and animals.

**Figure 1.**
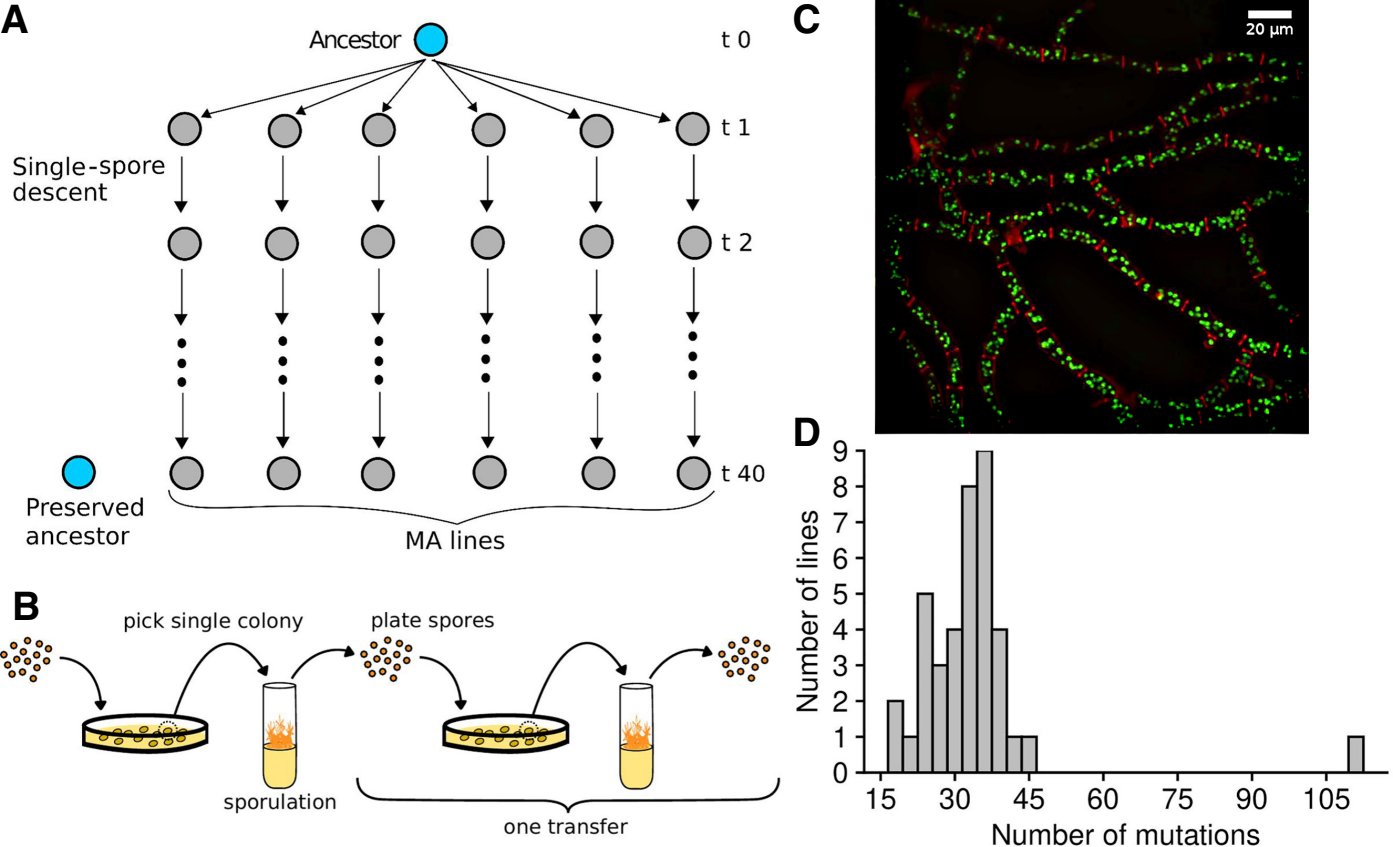
Overview of the mutation accumulation (MA) experiment. (*A*) Ancestors were split into multiple lines, which were propagated via single-spore descent. (*B*) Lines in the MA experiment were transferred by always picking a colony originating from a single spore from a plate, moving this to a test tube with Vogel's medium to allow sporulation, and then diluting spores and spreading them to a sorbose plate. (*C*) Micrograph of *N. crassa* mycelium, showing nuclei in fluorescent green and cell walls in red. (*D*) Distribution of mutations in the MA lines.

A previous study investigated the mutation rate during sexual reproduction in *N. crassa* and revealed that the mutation rate is especially high in regions of the genome targeted by RIP (Wang et al. 2020). However, only a small number of mutations was collected during asexual reproduction, not enough to infer variation in the mutation rate across the genome. Our study complements that of Wang et al. (2020) by allowing us to characterize the determinants of the mutation rate and spectrum during asexual reproduction, when RIP is not active. We used information on the chromatin structure of *N. crassa* to model variation in the mutation rate across the genome. We also resequenced strains obtained from natural populations and compared the predictions of our mutation model to patterns of natural genetic variation in order to assess whether the natural genetic variation reflects the observed mutation rate variation. Furthermore, we examined the effect of local sequence context on the mutation rate, and we characterized patterns of structural variants and complex mutations.

## Results

We initiated the MA experiment with two ancestors that were isogenic, except for the mating type locus. We split both ancestors into 20 lines, giving 40 MA lines in total. We then plated asexual spores (conidia) on plates, picked a single colony, transferred this colony into a test tube, and let the mycelium grow and make asexual spores. We subsequently plated these spores again to isolate a single spore, and this process was repeated for 40 transfers (Fig. 1B).

### Number of mitoses in the experiment

*N. crassa* is a filamentous organism, and it does not have a defined germline. All parts of the mycelium are capable of producing structures that produce asexual spores. Thus, the number of transfers during the MA experiment does not correspond to a generation in a natural way. Therefore, a reasonable unit for measuring the mutation rate is the number of mutations per mitosis.

We estimated the number of mitoses the MA lines went through based on the counts of nuclei in different phases of a transfer (Fig. 1B,C). Based on our estimate, the MA lines went through 25 [25, 26] (median, [95% HPDI]) mitoses in a single transfer. For the whole experiment of 40 transfers, this means that the MA lines went through 1015 [1003, 1026] mitoses.

### Mutations in the MA lines

To detect mutations in the MA lines, we sequenced the genomes of the MA ancestors and the MA lines using short-read sequencing with 150-bp paired-end reads. The sequencing depth was more than 30× on average, and ∼98% of reads were mapped to the reference genome (Supplemental Table S1). The reference genome of *N. crassa* contains 41,108,926 bp, and we called 98.7% of those bases on average. We used a pipeline based on the GATK best practices to call the mutations, followed by a manual inspection of alignments for each mutation.

After sequencing the MA lines, it became apparent that one of the lines had many of the same mutations as another line, likely owing to a mislabeling or contamination at some point of the MA experiment. This line was excluded from the analysis, leaving 39 MA lines in the data.

Accurate mutation calls are crucial for estimating mutation rates. The majority of our mutation calls had a maximum genotype quality score of 99 and were unambiguous (Supplemental Fig. S1; Supplemental Data Files S1, S2). However, to ensure the accuracy of our mutation calls, we verified a sample of mutations by Sanger sequencing. We had two verification sets: a set of mutation calls that were of lower quality based on a visual inspection of alignments, and a second set of randomly selected mutations across different genomic domains. We chose the mutations from the first set because if those calls were correct, then mutations with higher-quality scores are likely to be correct as well. We selected 29 point mutations to be confirmed. PCR or sequencing failed in six out 29 point mutations, and the remaining 23 point mutations were all confirmed. For the 37 small indels, PCR or sequencing failed in 10 of them, whereas 20 were confirmed, and seven were false positives. Of the 16 SVs tested, PCR or sequencing failed in nine, five were confirmed, and two were false positives. The randomly selected mutations of the second set were selected to understand if heterochromatin (H3K9 or centromeric domains) had higher rates of false-positive mutations. We randomly selected 15 point mutations in each of the three genomic domains (H3K9me3, centromeric, and euchromatin), 45 in total. One mutation located in a centromeric region failed to amplify by PCR, and all the rest of the 44 mutations were confirmed.

For point mutations, we never observed a false-positive mutation out of the total 67 mutations checked by Sanger sequencing; these included 23 mutations in euchromatic, 25 in H3K9me3, and 19 in centromeric regions (Supplemental Data Files S1, S3). Because mutations in the first verification set represented mutations with the worst genotype qualities, our genotyping for point mutations was very accurate (see Supplemental Results), although there was some uncertainty for small indels and SVs. All mutations that were false positives were excluded from the data.

To ensure that our pipeline calls mutations in an unbiased way, we simulated mutations to the *N. crassa* genome and then simulated short reads from this genome. We used two different scenarios to explore if the repetitive nature of heterochromatic regions created any bias. In the first scenario, the mutation rate was higher in H3K9me3 domains than in the rest of the genome, and in the second scenario, the mutation rate was uniform across the entire genome. There was no difference between the ratios of H3K9/euchromatin mutation rates whether we used mutations called from the simulated read data or used the true number of simulated mutations (see Supplemental Fig. S2; Supplemental Results).

In total, we observed 1322 mutations, with a median of 33 mutations per MA line. One of the MA lines had an excessive number of mutations (Fig. 1D), and it is possible that a mutation happened in this line that increased the mutation rate. There was a trend of increased C:G → A:T transversions in this line, but the rate was not statistically different from the rest of the MA lines (Supplemental Fig. S3). We did not observe any obvious candidate mutation that affected a DNA repair gene.

The breakdown of different mutation types among the MA lines was 1077 single-nucleotide mutations (SNMs), 134 insertions, 97 deletions, nine complex mutations in which a single mutational event created multiple adjacent nucleotide changes, and five translocations. The total mutation rate during asexual propagation was 0.03 [0.03, 0.04] mutations/genome/mitosis.

### Mutation rate variation across the genome

**Figure 2.**
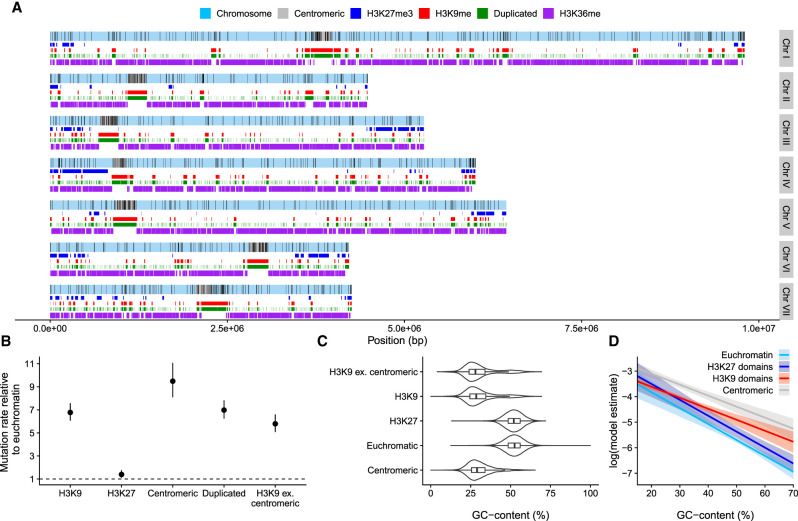
Variation in mutation rate across the genome. (*A*) Distribution of mutations along the seven chromosomes; black lines indicate mutations. Centromeric regions, H3K27 trimethylation, H3K36 dimethylation, H3K9 trimethylation domains, and duplicated regions are shown. (*B*) Relative mutation rates for different genomic domains. H3K9 ex. centromeric are H3K9me3 domains in which overlaps with centromeric regions have been excluded. Posterior medians and 95% HPD intervals are shown. (*C*) Violin plots overlaid with boxplots for GC-content in different domains. (*D*) Model estimates (on a log-scale) for the mutation rate from a model with GC-content, H3K9me3, H3K27me3, and centromeric domains as predictors. Lines are posterior medians, and envelopes are 95% HPD intervals.

To examine whether the chromatin structure influenced mutation rates, we gathered publicly available data for H3K9 trimethylation (H3K9me3), H3K27 trimethylation (H3K27me3), H3K36 dimethylation, and locations of *N. crassa* centromeres (Smith et al. 2011; Jamieson et al. 2013; Bicocca et al. 2018). We also examined regions of the genome containing ancestral duplicates defined by Wang et al. (2020), but the duplicated regions were almost perfectly correlated with H3K9me3 domains (Fig. 2A), so we did not use them in further analyses. Furthermore, H3K9me3 and H3K36me2 domains were nearly perfect mirror images of each other (Fig. 2A). H3K36me2 domains were thus excluded owing to containing the same information as the absence of H3K9me3.

Next, we examined the distribution of mutations across the seven *N. crassa* chromosomes. We observed that mutations were not uniformly distributed along the chromosomes but were concentrated in centromeric regions and regions of the genome marked by H3K9me3 (Fig. 2A). Examining relative mutation rates confirmed that the mutation rate was over sixfold higher in centromeric and H3K9me3 domains (Fig. 2B), whereas the effect of H3K27me3 domains was much smaller: The mutation rate in H3K27me3 domains relative to euchromatin was only 1.4 [1.1, 1.78] times higher.

We also observed that GC-content displayed a bimodal distribution and was lower in H3K9me3 and centromeric domains, barely overlapping with the distribution of GC-content in euchromatin (Fig. 2C). To clarify does the higher mutation rate in H3K9me3 domains arise from the GC-content itself, some other factor related to the chromatin modifications, or a combination of both, we examined the effect of GC-content on the mutation rate within the different domains. We observed that lower GC-content was associated with higher mutation rates within each domain, at different ranges of GC-content (Supplemental Fig. S4). However, the pattern was unclear in H3K27me3 domains, as few mutations were observed in H3K27me3 regions with low GC-content.

To investigate the joint effects of GC-content and chromatin modifications, we fitted models with different predictors, including GC-content, H3K9me3, H3K27me3, and centromeric domains. Based on model comparisons, the model with the best predictions included the effect of GC-content; the effects of H3K9me3, H3K27me3, and centromeric domains; and the interaction between the GC-content and H3K9me3 domain (Supplemental Table S2). Based on model weights, the next best model that included additional interaction between the H3K27 domain and GC-content was also plausible (Supplemental Table S2). However, the overall predictions for these two models were similar. There were only a few mutations in low GC areas of H3K27me domains, creating uncertainty in estimating a different slope for H3K27me3 domains; therefore, we prefer the first model with the highest weight. GC-content had a strong effect on the mutation rate, with areas of low GC having higher mutation rates (Fig. 2D). Within H3K9me3 domains, GC-content had a smaller effect on the mutation rate, and centromeric regions had a statistically detectable increase in the mutation rate in addition to the effect of the H3K9me3 domain (Fig. 2D; Supplemental Table S3), even if centromeric regions always have H3K9me3. H3K27me3 also increased the mutation rate on top of the GC-effect (Supplemental Table S3).

### Genetic variation in natural populations and mutation rate

**Figure 3.**
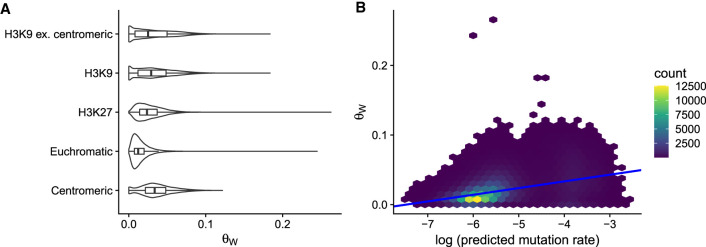
Natural genetic diversity and mutation rate. (*A*) Nucleotide polymorphism, *θ*_*W*_, was calculated across the genome in 200-bp windows, *n* = 202310. Violin plots overlaid with boxplots for the distribution of *θ*_*W*_ in different domains. (*B*) Relationship with *θ*_*W*_ and predicted mutation rate. For plotting, data were binned into hexes because of the high number of overlapping points, and the number of windows falling into each hex is shown by the legend.

To investigate the amount of genetic variation across the genome, we calculated nucleotide polymorphism, *θ*_*W*_, which measures how many polymorphic bases are found in a given length of sequence corrected for the sample size, across the genomes of natural strains in 200-bp windows. We observed that mean *θ*_*W*_ was higher in the other domains compared with euchromatin (Fig. 3A). The median estimate of *θ*_*W*_ was 0.0150 [0.0149,0.0151] in euchromatin; the difference to centromeric regions was 0.0213 [0.0210,0.0216] units; the difference to H3K9me3 domains was 0.0159 [0.0156,0.0161] units; and the difference to H3K27me3 domains was 0.0117 [0.0115,0.0119] units.

To cross-validate our mutation model results and to investigate the role of mutation in the maintenance of genetic variation across the genome, we used our mutation model (Supplemental Table S3) to predict variation in *θ*_*W*_ across the genome. We calculated the predicted mutation rate for each 200-bp window across the genome and observed that a simple linear model predicted a moderate amount of variation in *θ*_*W*_ (Fig. 3B). The slope of the regression line was 0.0096 [0.0095,0.0097], so a 10-fold increase in the predicted mutation rate meant an increase of 0.0096 in *θ*_*W*_. A measure of the model fit, the Bayesian *R*^2^ value was 0.22 [0.21,0.22]. Although this may seem like a low *R*^2^, one should take into account that this is after our mutation model has been challenged with completely new data, and other evolutionary mechanisms besides mutation also influence *θ*_*W*_. The choice of the window size was not important: We tested different window sizes and found the same relationship between the predicted mutation rate and *θ*_*W*_ (Supplemental Fig. S5). Larger windows even improved the fit, as there were fewer windows in which *θ*_*W*_ = 0; thus, our choice of a 200-bp window was conservative. We also checked that our results were robust to the windows in which *θ*_*W*_ = 0 by fitting different models that specifically modeled observations with zero (Supplemental Fig. S6). We obtained the same relationship between *θ*_*W*_ and the predicted mutation rate with these models. We further checked that the action of RIP was not solely responsible for this relationship by looking within the different domains (Supplemental Fig. S7; Supplemental Results). The predicted mutation rate had a positive relationship with *θ* within the different domains; therefore, the action of RIP cannot solely explain these results. Consequently, the mutation rate has a substantial influence on the amount of genetic variation that is present across the genome in *N. crassa*.

### Rate and spectrum of SNMs

Next, we examined the rate and spectrum of different types of mutations. The rate of SNMs across the whole genome was 6.7 [6.32, 7.11] × 10^−10^ mutations/bp/mitosis. The SNM rate in euchromatic regions was 2.46 [2.19, 2.77] × 10^−10^ mutations/bp/mitosis, and the SNM rate in H3K9me3 domains was 2.43 [2.25, 2.62] × 10^−9^ mutations/bp/mitosis. The ratio of transition to transversion (Ts/Tv) rates over the whole genome was 1.08 [0.96, 1.21], which is on the low end of reported Ts/Tv ratios. The Ts/Tv ratio of euchromatic regions was 1.49 [1.17, 1.91], which was higher than the Ts/Tv ratio in H3K9me domains, 0.93 [0.8, 1.08].

**Figure 4.**
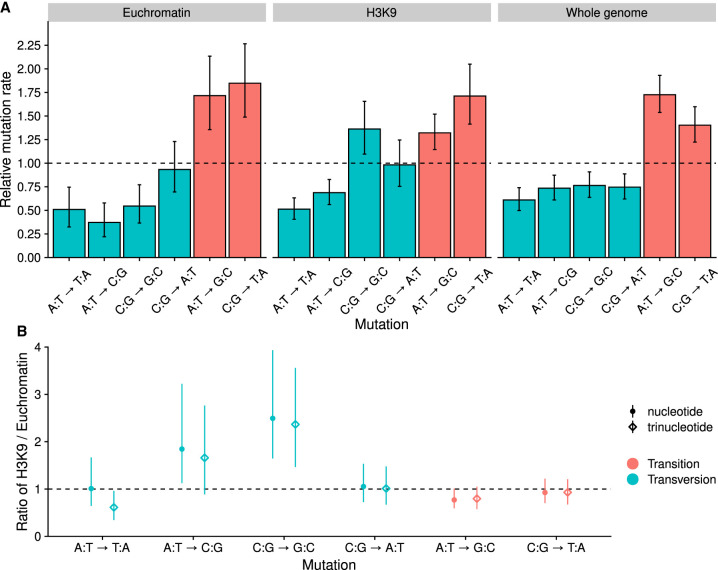
Mutation spectra. (*A*) Spectrum of relative SNM rates; the dashed line shows the expected rate if all mutations occurred at equal frequencies. Nucleotide frequencies were taken into account in calculating the relative rates. Error bars are 95% HPD intervals. (*B*) Ratios of relative mutation rates in H3K9me3 over euchromatin. Points show ratios corrected for nucleotide frequencies, and diamonds show ratios corrected for trinucleotide frequencies. Estimates are medians, and the range shows the 95% HPD interval of the ratio. If the interval estimate is different from one, the mutation rate is different in H3K9me3 domains and euchromatin.

As seen from the different transition to transversion ratios, the spectra of SNMs were different for H3K9me3 domains versus the rest of the genome (Fig. 4A). We calculated ratios of the relative mutation rates in different domains by taking different nucleotide or trinucleotide frequencies (see below) into account in H3K9me3 domains and euchromatin. C:G → G:C transversions were more common in H3K9 domains (Fig. 4B). There was also weak evidence that A:T → C:G and A:T → G:C mutations could have different rates in the different domains. Their ratios were barely different from one when nucleotide frequencies were taken into account, but the ratios barely overlapped with one when mutation rates were corrected for trinucleotide frequencies (Fig. 4B). However, A:T → T:A transversions had a lower rate in H3K9me3 domains after correcting for trinucleotide frequencies. Over the whole genome, the four different transversions occur at similar rates (Fig. 4A) and the two transitions at higher rates. The A:T → G:C transition was the most common SNM and more common than the C:G → T:A transition. The ratio of A:T → G:C to C:G → T:A transitions was 1.23 [1.04, 1.44].

### Effects of local base composition

To better understand factors influencing SNM rates, we looked at the effect of local base pair context. For each SNM, we extracted the two adjacent base pairs to get the trinucleotide context. We combined trinucleotides with respect to sequence complementarity as the strand in which the mutation occurred is unknown; this leaves 32 trinucleotide classes. We calculated trinucleotide frequencies and observed that over the whole genome, the observed frequencies were approximately at the expected frequencies based on GC-content (Supplemental Fig S8). However, in regions marked by H3K9 trimethylation, there were strong departures from the expected trinucleotide frequencies (Supplemental Fig. S8). Prompted by this observation, we investigated whether differences in the trinucleotide mutation rates could explain the observed differences in mutation rates across the genome. We compared different models with the trinucleotide classes and the effects of epigenetic domains. The model that gave the best predictions included an effect of the trinucleotide classes, effect of H3K9me3, centromeric regions, and H3K27me3 regions, but no interactions between the trinucleotide class and any of the epigenetic domains (Supplemental Table S4). We observed the same results regarding the epigenetic domains as before: The mutation rate was 8.1 [6.8, 9.5] times higher in H3K9me3 domains; centromeric regions had an additional increase on top of H3K9me3; and there was a small, 1.5 [1.1, 2.0]-fold, increase in the mutation rate in H3K27me3 domains (Supplemental Fig. S9). Thus, differences in trinucleotide composition were not driving the mutation rate differences in the different domains.

**Figure 5.**
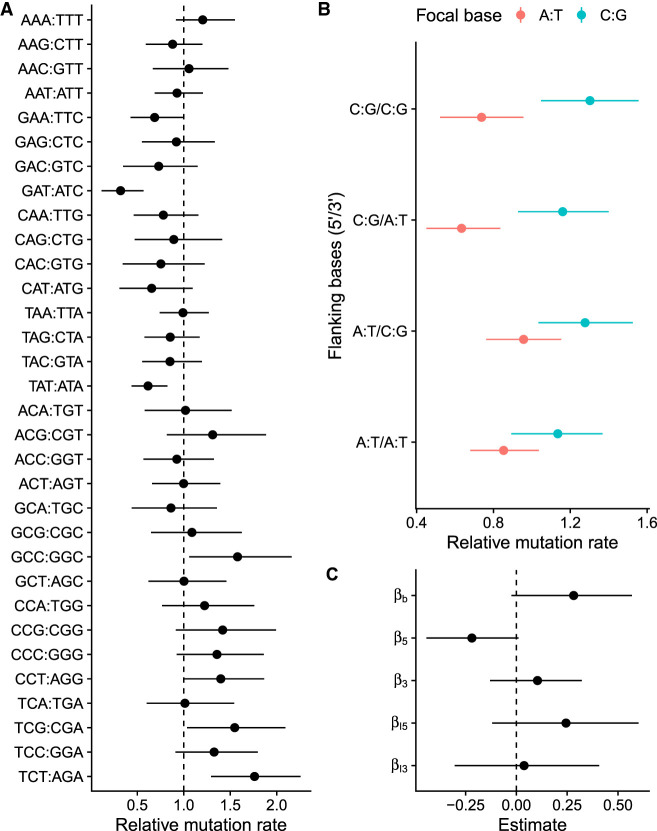
Mutation rate and sequence context. (*A*) Relative mutation rates for the 32 different classes of trinucleotides. (*B*) Model predictions for effects of flanking bases. (*C*) Estimates of model coefficients for effects of flanking bases. *β*_*b*_ is the effect of focal C:G relative to A:T; *β*_5_ is the effect of flanking 5′ C:G relative to A:T; *β*_3_ is the effect of flanking 3′ C:G relative to A:T; *B*_*I*5_ is the interaction effect of 5′ C:G when the focal base is C:G; and *B*_*I*3_ is the interaction effect of 3′ C:G when the focal base is C:G. Range shows a 95% HPD interval of the relative mutation rate.

After taking trinucleotide frequencies, and the effects of the epigenetic domains into account, mutations were not equally distributed across the different trinucleotide classes (Fig. 5A). The trinucleotide class GAT:ATC had the lowest relative mutation rate, whereas TCT:AGA had the highest. Trinucleotides with adjacent C:G pairs tend to have high relative mutation rates.

To investigate the effects of C:G and A:T base pairs in either 5′ or 3′ flanking positions, we fitted a linear model that included the effects of the flanking bases and the mutating base pair, and we also incorporated the uncertainty in the relative mutation rate for each trinucleotide class. The model predictions are shown in Figure 5B, and there was a tendency for trinucleotides with C:G as the mutating base to have a higher relative mutation rate compared with that of A:T trinucleotides. However, this effect was not significant (Fig. 5C). Similarly, for A:T trinucleotides, there was a tendency for 5′ C:G to protect against mutation, but this effect was not significant as the 95% HPD interval barely includes zero (Fig. 5C). When the mutating base pair was C:G, neither 5′ or 3′ base had any detectable effect (Fig. 5C).

### Deletions, insertions, and translocations

**Figure 6.**
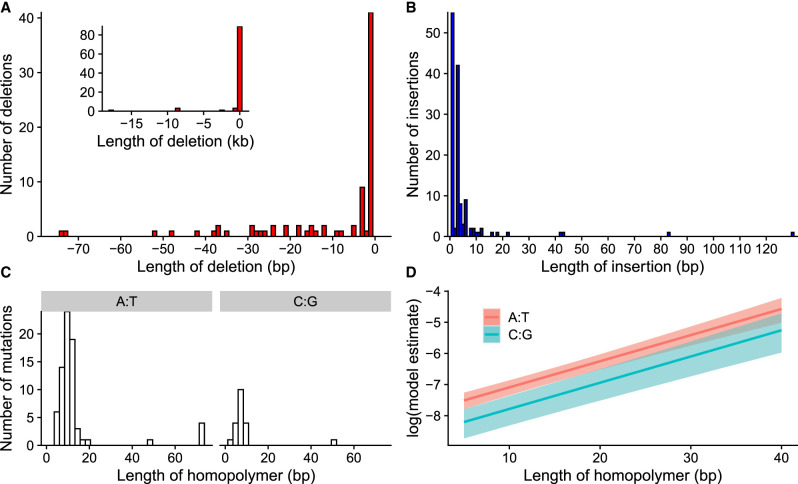
Properties of deletions and insertions. (*A*) Distribution of deletion lengths in the range of 0 to 75 bp; *inset* shows the full distribution. (*B*) Distribution of insertion lengths. (*C*) Distribution of homopolymer lengths for those mutations that occurred in either A:T or C:G homopolymer stretches. (*D*) Model estimates for mutation rate in homopolymers; A:T homopolymers had an overall higher mutation rate, and longer homopolymers had higher mutation rates.

We also examined the structural variants that occurred in the MA lines. We observed 97 deletions and 134 insertions. Frequent lengths for deletions and insertions were changes of 1 bp; 88 out of 96 1-bp indels occurred in homopolymer stretches. The second most common length was 3 bp, which was predominantly changes in microsatellite repeats. Some large deletions were observed: The largest deletion was 17.8 kb; there were three deletions ∼8.8 kb, and one ∼2.5 kb. Otherwise, most deletions were <100 bp (Fig 6A). The largest observed insertion was 130 bp, with most insertions <20 bp (Fig. 6B).

Mutation rates for insertions and deletions are shown in Table 1. When all deletions and insertions were included in the analysis, insertions had slightly higher rates than deletions (Table 1). However, when we excluded homopolymers, microsatellites, and other repeats from the analysis, we observed that the rate of deletions was three times higher than insertions (Table 1). Given that the mean length of deletions excluding repeats was 1160 bp, which was longer than the 27-bp mean length for insertions, there was mutation pressure to lose DNA. Some of the large deletions observed in our data were outliers, but deletions tended to be longer at all scales (Fig. 6). For mutations that occurred in repeats, the mutation rate of insertions was more than twofold higher than deletions.

**Table 1.**
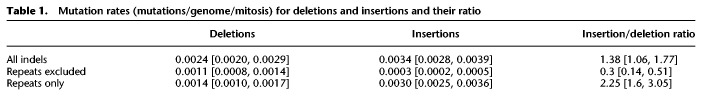
Mutation rates (mutations/genome/mitosis) for deletions and insertions and their ratio

Homopolymer stretches had particularly high rates of indel mutations; we observed 92 mutations in homopolymers, and most mutations in homopolymers were indels of 1 bp. More mutations occurred in A:T than in C:G homopolymers (Fig. 6C). A:T homopolymer loci are approximately 1.7 times more common in the genome, but even when adjusting for frequencies, the mutation rate in A:T homopolymers was 1.79 [1.4, 2.22] × 10^−8^ mutations/locus/mitosis compared with the rate in C:G homopolymers of 8.15 [4.89, 11.86] × 10^−9^ mutations/locus/mitosis. Thus, mutations in A:T homopolymers were 2.2 [1.27, 3.49] times more common than in C:G polymers. We also observed that longer homopolymers had higher mutation rates. In a model with a polymer length and polymer type, the length had the same effect for both A:T and C:G homopolymers (Fig. 6D). This suggests that replication slippage, which is the mechanism suggested to be involved in indel mutations in repeats, tends to occur more often in longer repeats as expected.

There were differences in indel rates in the different genomic regions. We observed that deletions had a higher mutation rate in centromeric regions and in regions marked by H3K9me3 (Supplemental Fig. S10) compared with euchromatin, even when only deletions in repeats were considered. For deletions excluding repeats, H3K9me3 and H3K27me3 domains had a higher mutation rate than euchromatin (Supplemental Fig. S10). For insertions, we did not observe any differences in the mutation rate in different domains (Supplemental Fig. S10).

We observed five translocations in the MA lines, two of which were among the SVs confirmed by PCR and Sanger sequencing. Three translocations were from one chromosome to another, and two occurred among unmapped contigs. The mean translocation length was 316 bp. The translocation rate was 1.19 [0.33, 2.41] × 10^−4^ translocations/genome/mitosis. Because of their rarity, we do not have enough translocations to further analyze their properties.

### Complex mutations

We observed nine cases in which two SNMs or 1-bp indels occurred within few base pairs of each other in the same MA line. Although it is possible that two independent mutations occurred next to each other, it is unlikely. These changes were more likely caused by a single mutational event. The observed complex mutational events are listed in Table 2. To confirm whether these changes were caused by a single mutational event or two independent events, we used Sanger sequencing to check the genotypes of MA lines from intermediate transfers stored during the experiment. We always observed that the two changes appeared together (Table 2). The most parsimonious explanation is that these changes appeared as a result of single mutational events, likely caused by a DNA repair error via an error-prone DNA polymerase.

**Table 2.**
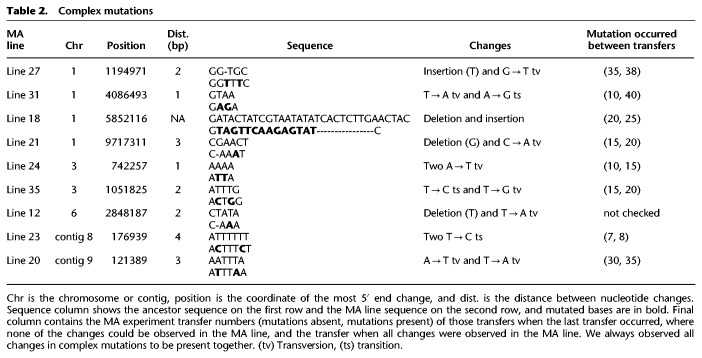
Complex mutations

We treated complex mutations as single events in calculations in which all mutations were used to calculate overall mutation rates. The rate of complex mutations was 5.42 [2.22, 9.3] × 10^−12^ mutations/bp/mitosis. The rate of SNMs over the rate of complex mutations was 123.7 [57.48, 231.49], making point mutations more than 100-fold more common than complex events.

### Comparison of mutation rate and spectra during meiosis and mitosis

Wang et al. (2020) observed an extremely high mutation rate during meiosis, as a result of C:G → T:A transitions induced by RIP. We reanalyzed their data in combination with the chromatin modification data (for details, see Supplemental Results). We observed heterogeneity in the activation of RIP that was not taken into account in the original analysis (see Supplemental Fig. S11; Supplemental Results). Based on our analysis, the mutation rate during meiosis in euchromatin was 7.15 [3.85, 10.72] × 10^−7^ mutations/meiosis/bp and was 1.68 [0.075, 5.09] × 10^−5^ in H3K9me3 domains. The mutation rate was substantially higher during meiosis than mitosis, as observed by Wang et al. (2020). Comparing mutation spectra in mitosis and meiosis shows that in euchromatic regions, A:T → C:G, and C:G → G:C transversions were more common in meiosis, whereas C:G → A:T transversions were less common (Supplemental Fig. S12). In heterochromatin, C:G → T:A transitions overwhelmed all other mutations in meiosis (Supplemental Figs. S11, S12).

## Discussion

We have generated a highly precise estimate of spontaneous mutation rate during asexual growth in *N. crassa*. Our estimate of the point mutation rate across the whole genome of 6.7 [6.32, 7.11] × 10^−10^ mutations/bp/mitosis is higher, although it is close to an estimate of asexual mutation rate of 6.03 × 10^−10^ obtained by Wang et al. (2020), with only 64 observed mutations. A previous estimate from marker gene studies suggested that the mutation rate is 4.10–4.66 × 10^−9^ (Lynch et al. 2016), but neither our results nor the results of Wang et al. (2020) agree with this. The point mutation rate in euchromatic regions was 0.007 [0.006, 0.008] mutations/genome/mitosis, which is in line with results obtained by Drake (1991), who observed that the mutation rate per genome for microbes seems to be around 0.003 mutations per genome per generation with approximately twofold variation around this mean. Thus, the asexual mutation rate in *N. crassa* in euchromatic regions seems to be rather typical for a microbe.

*N. crassa* has a striking difference in the rate and spectrum of mutation during sexual and asexual reproduction (Wang et al. 2020). During sexual reproduction, a genome defense mechanism called RIP is activated, which recognizes duplicated regions in premeiotic cells and induces C:G → T:A transitions in those regions (Selker 1990). RIP presumably protects the genome against transposons and other selfish genetic elements. Mutations induced during sexual reproduction happen mainly in ancestral duplications (Wang et al. 2020), and these regions nearly completely overlap with regions of H3K9 trimethylation. In contrast, during asexual reproduction, although H3K9me3 domains have a higher mutation rate than euchromatin, the difference is much smaller and the spectrum of mutations is different compared with meiosis. For euchromatic regions, our analysis supports a higher mutation rate during meiosis than in mitosis. We also observed that the spectrum of mutations was different in meiosis, but notably, there was no difference in C:G → T:A transitions in euchromatin. Moreover, because gene density is much higher in euchromatic regions, the action of RIP likely does not result in a high genetic load, but increased meiotic mutation rate in euchromatin may do so.

Although many studies have reported effects of chromatin structure on mutation rates, these studies have often been based on indirect inference from species divergence and polymorphism (Washietl et al. 2008; Sasaki et al. 2009; Ying et al. 2010). We observed directly extensive variation across the genome in the mutation rate and mutation spectra owing to chromatin modifications. In *N. crassa*, H3K9 trimethylation determines heterochromatic regions, and H3K27 trimethylation is a mark for facultative heterochromatin (Jamieson et al. 2013). Centromeric regions determined by the presence of centromeric histone variant CenH3 always overlap with H3K9 trimethylation (Smith et al. 2011). Methylation in H3K9 and H3K36 are almost completely mutually exclusive. However, H3K36me2 is not a straightforward mark of euchromatin as it can be deposited by two enzymes: SET-2 and ASH1. Genes marked with H3K36 methylation by SET-2 are actively transcribed, whereas genes marked with H3K36 methylation by ASH1 are silenced and can be further marked by H3K27 trimethylation (Bicocca et al. 2018). SET-2 is responsible for most of H3K36 methylation, and we considered all regions that lacked H3K9me3 and H3K27me3 to be euchromatin. The mutation rate for SNMs in domains marked by H3K9me3 was 10-fold higher than that in euchromatic regions. We also observed that the mutation rate was slightly elevated in H3K27me3 domains, although this effect was much smaller than that for H3K9me3. In centromeric regions, there was an additional effect of an increased mutation rate on top of the H3K9me3 effect.

An increased mutation rate in heterochromatic and centromeric regions has also been found by Weng et al. (2019) in the plant *Arabidopsis thaliana*. These results are also in line with observations of human cancer cells that have higher rates of mutation in heterochromatic regions (Schuster-Böckler and Lehner 2012; Polak et al. 2015). Monroe et al. (2022) observed that in *Arabidopsis*, the mutation rate was affected by several different epigenetic marks; they suggested that the mutation rate was lower in genes that were actively transcribed and was perhaps even fine-tuned for highly expressed genes, possibly owing to the presence of H3K36 methylation or other epigenetic marks of active transcription. However, our data set does not contain enough mutations to address if gene expression levels quantitatively influence the mutation rate in *N.* *crassa*.

What is the mechanism causing mutation rate variation across the genome? Chromatin structure is involved in DNA repair, and this may explain why heterochromatic regions have a higher mutation rate. In yeast, actively transcribed regions contain acetylation at H3K56, which suppresses spontaneous mutations (Kadyrova et al. 2013). Moreover, analyses based on human tumors suggest that DNA mismatch repair works more efficiently in euchromatin than in heterochromatin, and it is DNA repair that is variable, not the supply of mutations themselves (Supek and Lehner 2015). Further evidence supporting the DNA accessibility hypothesis was gathered by a study performed by Yazdi et al. (2015); the investigators observed that mutation rate did not increase in high nucleosome occupancy regions in strains in which DNA repair machinery was knocked out, in contrast to wild-type strains. Recently, Habig et al. (2021) investigated how epigenetic modifications affect the mutation rate in the fungus *Zymoseptoria tritici*. They observed that H3K27 trimethylation increased the mutation rate, as it did in this study. Furthermore, Habig et al. (2021) observed that this effect disappeared from those regions in a mutant lacking H3K27me3. This suggested that H3K27me3 is causal, and DNA accessibility may be the reason. Habig et al. (2021) did not detect an effect for H3K9me3 like we observed, which may indicate that there are species specific differences in heterochromatin.

Differential exposure of heterochromatin to natural mutagens, such as oxidative damage, does not seem to explain our results. As mutations typically associated with oxidative damage—G → C transversions, C → T transitions, and G → T transversions (McBride et al. 1991; Cheng et al. 1992)—were not systematically overrepresented in regions with H3K9 trimethylation, only the relative amount of G → C transversions differed between euchromatic and H3K9 trimethylated domains.

Among the observed mutations, sequence context had an effect on the mutation rate. It is known that local sequence context can influence the probability of mutation, and this phenomenon has been frequently observed (Ness et al. 2015). However, the mechanisms of why some sequence contexts are prone to mutation are less clear. We observed a tendency of 5′ C:G base pair to protect against mutation when the mutating base pair is A:T. A similar phenomenon has been observed before, as the formation of thymine–thymine cyclobutane dimers owing to DNA damage caused by sunlight is dependent on flanking sequence, even if the biophysical basis is unclear (Law et al. 2013). We also observed a tendency for trinucleotides with C:G as the mutating base pair to have higher relative mutation rates, possibly owing to trinucleotides with GG dimers being susceptible to oxidation (Hanrahan et al. 1997).

We did not find evidence that trinucleotide mutation rates were different in regions of H3K9me3. One hypothesis for different trinucleotide mutation rates in heterochromatin would be that the presence of DNA methylation increases the mutation rate in certain contexts. In *N. crassa*, DNA methylation occurs only in H3K9 trimethylated domains (Tamaru and Selker 2001), and deamination of 5-methylcytosine is a known cause of mutations (Cooper et al. 2010). However, deamination should cause mainly C:G → T:A transitions, and we did not observe an excess of C:G → A:T transitions in H3K9me3 domains. Instead, we observed an excess of C:G → G:C transversions in H3K9me3 domains. Therefore, DNA methylation does not contribute substantially to spontaneous genetic mutations in *N. crassa*, possibly because DNA methylation is so rare in this species (Hosseini et al. 2020).

For spontaneous insertions and deletions, we observed that deletions were more common than insertions when repeated sequences were excluded. Because deletions also tended to be longer, mutations have the tendency to reduce genome size. Similar patterns in mutational bias have been observed for *Drosophila melanogaster* (Leushkin et al. 2013). For repeated sequences, we analyzed homopolymer sequences in more detail. We found that although A:T homopolymers are more common in *N.* *crassa*, they also have a higher mutation rate. This is in contrast to observations in nematodes, where C:G homopolymers had much higher mutation rates compared with those of A:T homopolymers (Denver et al. 2004), suggesting that there are species-specific differences in mutation rates of these sequences.

We identified mutations with multiple base changes owing to a single mutational event. Such complex mutations are thought to arise from the action of error-prone trans-lesion DNA polymerases, such as Pol ζ (Stone et al. 2012). A recent large-scale survey of human trios identified many multinucleotide mutations and observed that mutations that were 2–10 bp apart showed an overrepresentation of A:T → T:A, and A:T → G:C mutations (Besenbacher et al. 2016). Our results are compatible with this pattern as most nucleotide changes in complex mutations were either A:T → T:A or A:T → G:C. We did not observe GC → AA or GA → TT tandem mutations, which are the most common tandem mutations in humans (Harris and Nielsen 2014; Besenbacher et al. 2016). In *N. crassa*, complex mutations represented only 0.7% of observed mutations, which is smaller than the ∼3% observed in humans. Thus, the impact of multinucleotide mutations on population genetic inference may be small in *N.* *crassa*.

Finally, the implication for evolutionary genetics is that variation in mutation rates must be taken into account in order to quantify which evolutionary forces act on the genome. Genes residing in different regions of the genome can differ substantially in their mutation loads, and it seems that variation in mutation rates also has a large part in determining how much genetic variation is segregating in a given region of the genome. To accurately estimate imprints of selection on natural genetic variation, we must account for variation in mutation rates in evolutionary models. Therefore, detailed models of mutation rates are needed for different species.

## Methods

### MA experiment

We used two ancestors for the MA experiment: B 26708 and B 26709. These strains have been generated by backcrossing mating type *mat a* from strain 4200 into a 2489 background nine times (Kronholm et al. 2020). There were 20 MA lines for both ancestors, giving 40 lines in total. Common protocols for culturing *N. crassa* were followed (see Supplemental Methods). We propagated the MA lines for 40 transfers.

To estimate the number of mitoses that occured in the MA experiment during a transfer, we used a strain that had been tagged with fluorescent protein at histone H1 (Freitag et al. 2004). Using this strain, we counted the numbers of nuclei that were present in each phase of a single transfer of the MA experiment with confocal microscopy (Fig. 1C), and we used these data to estimate the number of mitoses the lines went through (see Supplemental Methods).

### Strains from natural populations

We obtained 33 strains from the Fungal Genetics Stock Center (McCluskey et al. 2010) and resequenced these strains. In addition, we obtained genome sequencing data for another 23 strains from Zhao et al. (2015). In total, including the laboratory strain 2489, we had 57 strains with sequencing data (Supplemental Table S5).

### DNA extraction

We extracted DNA from the strains using phenol–chloroform extraction and further purified it via polyethyleneglycol precipitation. DNA quality was checked on agarose gels. For a detailed protocol, see the Supplemental Methods.

### Genome sequencing

DNA was sequenced at Novogene (Cambridge, UK), using an Illumina platform with paired-end 150-bp libraries. Libraries were prepared by fragmenting the DNA by sonication, adapter ligation, and PCR amplification. Libraries were sequenced to 30× target coverage. Reads with adapter sequences, >10% N's, or >50% bases of low-quality *Q*-score ≤ 5 were removed.

### Read mapping and genotyping

We mapped reads against the *N. crassa* reference genome (assembly version NC12) using BWA-MEM (Li 2013). The strain used for the original genome project was 2489, so the MA experiment ancestors should be nearly identical to the reference. We did not observe any bias in coverage (Supplemental Fig. S13).

We used the GATK version 4.2.0.0 (McKenna et al. 2010) pipeline to call SNMs and indels. First, we ran Haplotypecaller for each sample and then genotyped all samples together. *N. crassa* is haploid, but we ran Haplotypecaller in diploid mode because mapping errors manifest as heterozygous sites in haploids (Kronholm et al. 2017; see also Li 2014; Ness et al. 2012). We used wormtable version 0.1.5 (Kelleher et al. 2013) and custom Python scripts to filter for high-quality sites. To produce the final set of mutations, we checked all candidate mutations manually by inspecting the alignment in the Integrative Genomics Viewer (IGV; Thorvaldsdóttir et al. 2013); for examples, see Supplemental Figs. S14–S22 and Supplemental Data File S2. SNPs were genotyped using the same pipeline (for details, see Supplemental Methods).

To genotype structural variants, we first compared the performance of different SV genotyping algorithms using simulated data. We simulated SVs by modifying the *N. crassa* genome using SURVIVOR 1.0.7 (Jeffares et al. 2017). We then simulated short reads from these genomes using DWGSIM 0.1.11 (https://github.com/nh13/DWGSIM). We simulated different scenarios with different amounts, types, and lengths of SVs (Supplemental Table S6; Supplemental Fig. S23). Then we proceeded to call the simulated SVs with different SV callers. For details, see the Supplemental Methods. DELLY 0.8.7 (Rausch et al. 2012) and Lumpy 0.2.13 (Layer et al. 2014) were the callers that performed best, and they were selected for genotyping. SVs with a genotype quality score below 30 and read depth below 10 were discarded. Alignments for each called SV were manually inspected in IGV. Simulations were also performed for CNV callers (for details, see Supplemental Table S7; Supplemental Methods).

#### Validation of mutations with Sanger sequencing

To verify a sample of the observed mutations, we performed Sanger sequencing using standard methods. First, we selected a set of mutations that passed our threshold but had the lowest quality scores: 30 point mutations, 37 small indels, and 16 larger structural variants; 83 in total. Second, we selected another set of mutations at random: 15 point mutations from three genomic regions (H3K9, centromeric, and eurchromatin), 45 in total. In total, we attempted to confirm 128 mutations; a list of primers and confirmed mutations are given in the Supplemental Data File S1 and Sanger sequence alignments in Supplemental Data File S3. Additionally, complex mutations, for which a suspected single event created multiple changes, were selected for verification, and we sequenced time points from the middle of the MA experiment to see if the multiple changed sites appeared together in the MA line.

#### Chromatin modifications

We used publicly available data to determine where chromatin modifications occurred. ChIP-seq reads for H3K9 trimethylation and H3K27 trimethylation were obtained from Jamieson et al. (2013) under NCBI Sequence Read Archive (SRA; https://www.ncbi.nlm.nih.gov/sra) accession numbers SRX248101 and SRX248097. Jamieson et al. (2013) used the same strain (2489) that we used as the ancestor of the MA experiment. They also tested whether different growth media had an effect on H3K27me3 regions and observed that they were stable. Jamieson et al. (2013) extracted chromatin from mycelium, which gives rise to the asexual spores used for transfers in our experiment, so the chromatin states they observed should be relevant for our experiment. Taken together, these data provide a very good approximation of the chromatin states that the ancestors of the MA lines had in our experiment. Data for H3K36 methylation were obtained from Bicocca et al. (2018) under SRA accession number SRX4549854. Data for centromeric regions were obtained from Smith et al. (2011). For details, see Supplemental Methods.

### Statistical analysis

We used Bayesian statistics in all statistical modeling. As a rule of thumb for comparison to frequentist methods: If a 95% interval estimate of a parameter does not contain zero, the parameter is statistically different from zero, that is, significant. We tested differences in mutation rates by computing ratios from posterior distributions. If the interval of the ratio did not include one, the mutation rates were statistically different. Estimates were reported as medians and 95% highest posterior density intervals in square brackets.

#### Mutation rate estimates

Mutation rates were estimated using Bayesian Poisson models implemented with the Stan language (Carpenter et al. 2017) interfaced from R 3.6.0 (R Core Team 2019) with the “brms” package (Bürkner 2017). The basic model for estimating the mutation rate was

(1) $$\begin{array}{rl} & y_{i}∼\mathrm{Poisson}(\lambda_{i})\\ & \;\log(\lambda_{i})=\alpha \\ & \;\alpha ∼N(0,10), \end{array}$$

where *y*_*i*_ is the number of mutations in *i*th MA line, *λ*_*i*_ is the Poisson rate parameter, and *α* is the intercept. The linear model part was modified accordingly if other predictors were used. We can then calculate the mutation rate, *μ*, from posterior distributions as

(2) $$\mu =\frac{\exp(\alpha )}{Ntm},$$

where *N* is the number of called nucleotides, *t* is the number of transfers the MA lines went through, and *m* is the number of mitoses per transfer. We used the posterior distribution for *m*, so any uncertainty in number of mitoses is incorporated into our estimated mutation rate. To get the mutation rate per genome, *N* is removed from the denominator. We used a weakly regularizing prior for *α*. Settings for MCMC estimation were as follows: 1000 iterations of warm-up followed by 3000 iterations of sampling with four independent chains. MCMC convergence was monitored by traceplots and $\hat{R}$ values. No convergence problems were observed. For details of the other models, see Supplemental Methods.

#### Mutation rate in repeats

Homopolymer sequences in the genome were detected using MISA (Beier et al. 2017). We used 5 bp as the minimum homopolymer length and extracted repeat counts for all homopolymer loci in the genome. Counts of homopolymers of different lengths were used as an offset term in a model used to estimate mutation rates in homopolymers.

#### Population genetics

We estimated nucleotide polymorphism, *θ*_*W*_, from the SNP data for the population sample following the method of Ferretti et al. (2012), which allows us to deal with missing data.

## Data access

The genome sequencing data generated in this study have been submitted to the NCBI BioProject database (https://www.ncbi.nlm.nih.gov/bioproject/) under accession number PRJNA839531. Other data and scripts are available at GitHub (https://github.com/ikron/mutation_ms) and as Supplemental Material.

## Supplementary Material

Supplemental Material
