# Supplementary material for: Chromatin structure influences rate and spectrum of spontaneous mutations in Neurospora crassa
Source: Genome Res. 2023 Apr;33(4):599–611. doi: 10.1101/gr.276992.122 (PMC10234303; doi:10.1101/gr.276992.122)
Supplement: Supplemental Material [file supp_33_4_599__DC1.html]

Chromatin structure influences rate and spectrum of spontaneous mutations in Neurospora crassa — Chromatin structure influences rate and spectrum of spontaneous mutations in Neurospora crassa — Supplemental Material 

# Chromatin structure influences rate and spectrum of spontaneous mutations in *Neurospora crassa*

## Supplemental Material

- Supplementary\_file\_S1.xlsx
- Supplementary\_file\_S2.zip
- Supplementary\_file\_S3.zip
- Supplememtal\_Scripts\_v2.zip
- Supplemental\_Information.pdf
