# Supplementary figures and images for: Chromatin structure influences rate and spectrum of spontaneous mutations in Neurospora crassa
Source: Genome Res. 2023 Apr;33(4):599–611. doi: 10.1101/gr.276992.122 (PMC10234303; doi:10.1101/gr.276992.122)

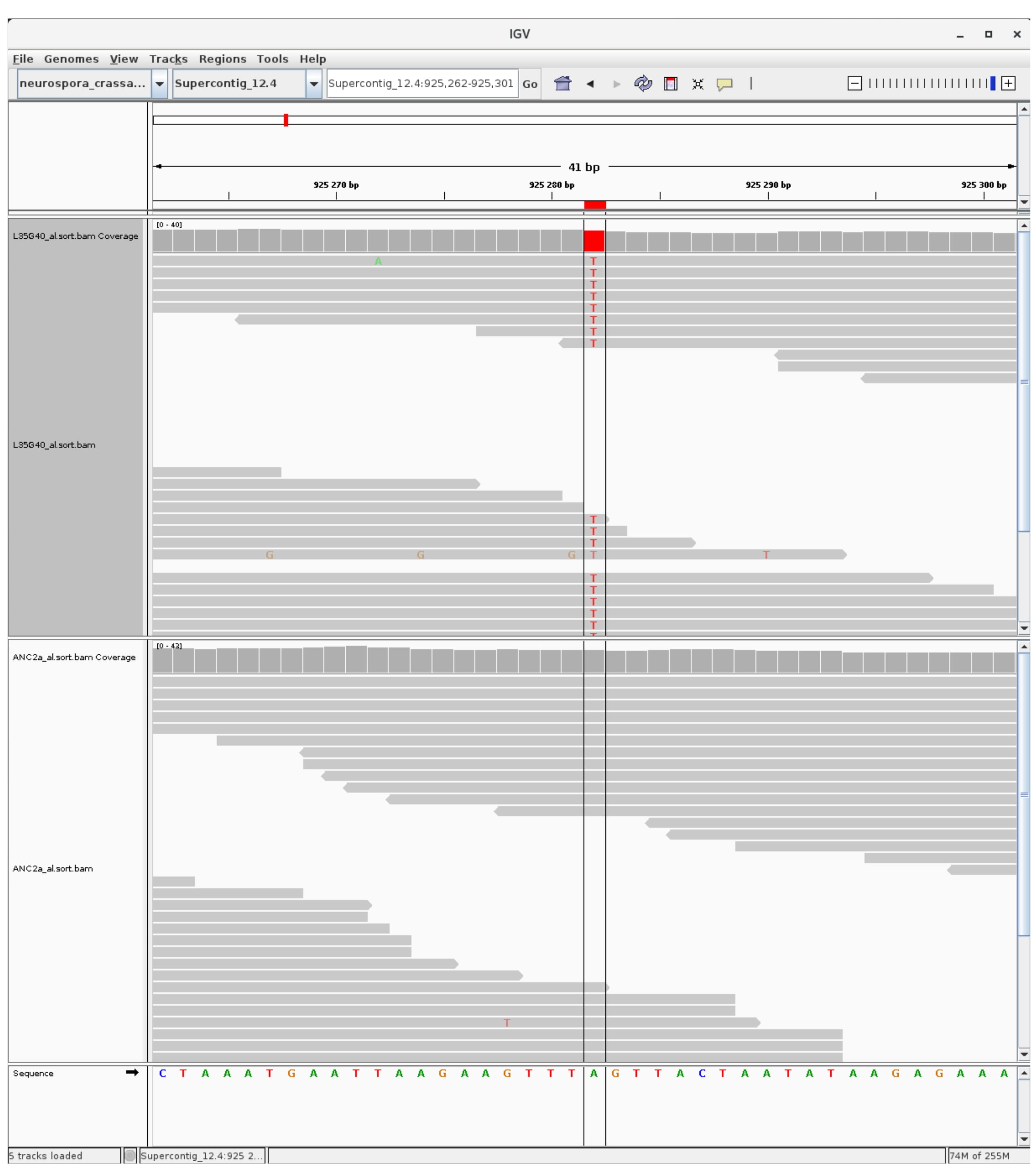

Supplement: Supplemental Material [file supp_gr.276992.122_Supplementary_file_S2.zip › IGV_screenshots/mutation_centromer_1.jpg]

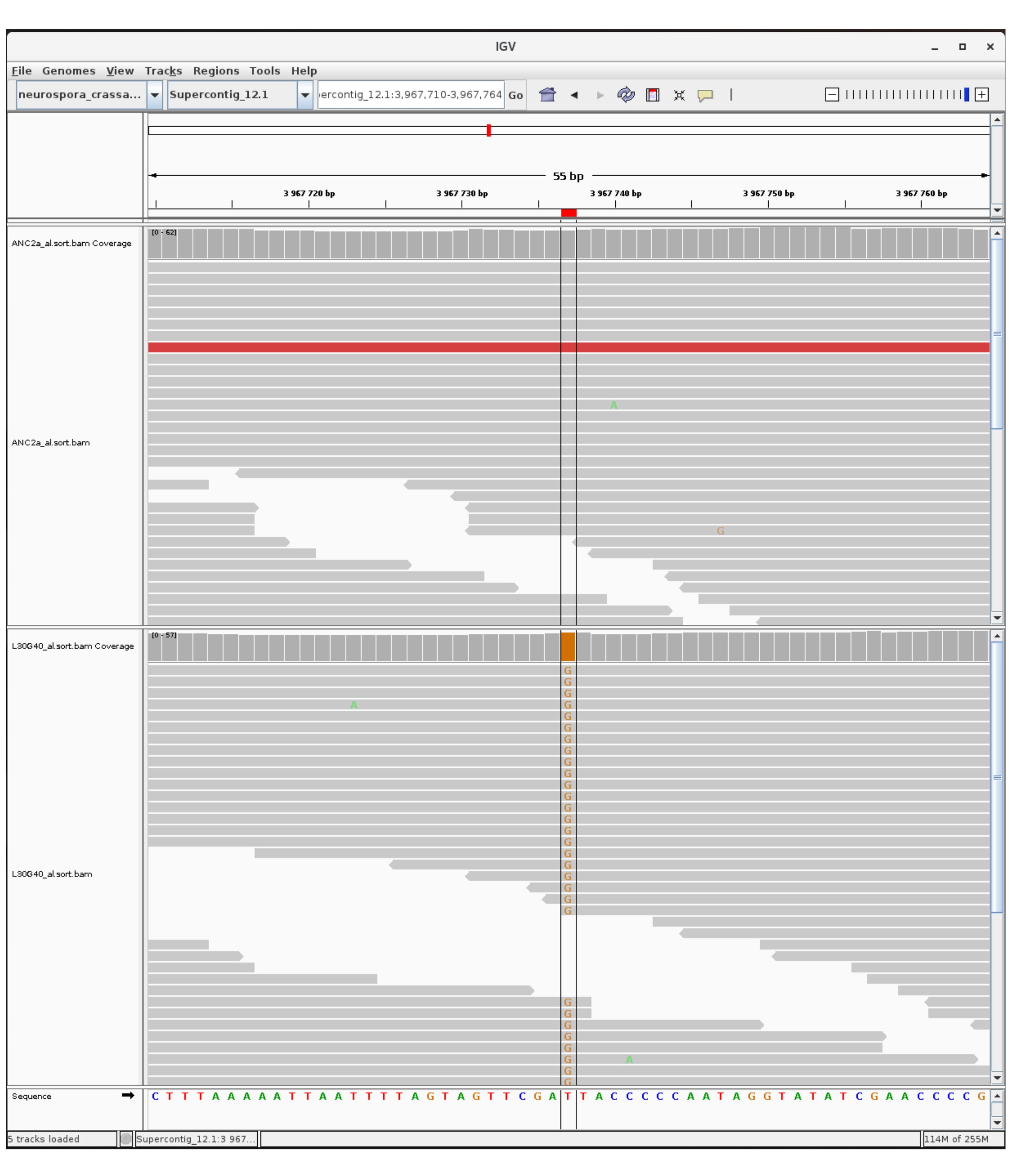

Supplement: Supplemental Material [file supp_gr.276992.122_Supplementary_file_S2.zip › IGV_screenshots/mutation_centromer_10.jpg]

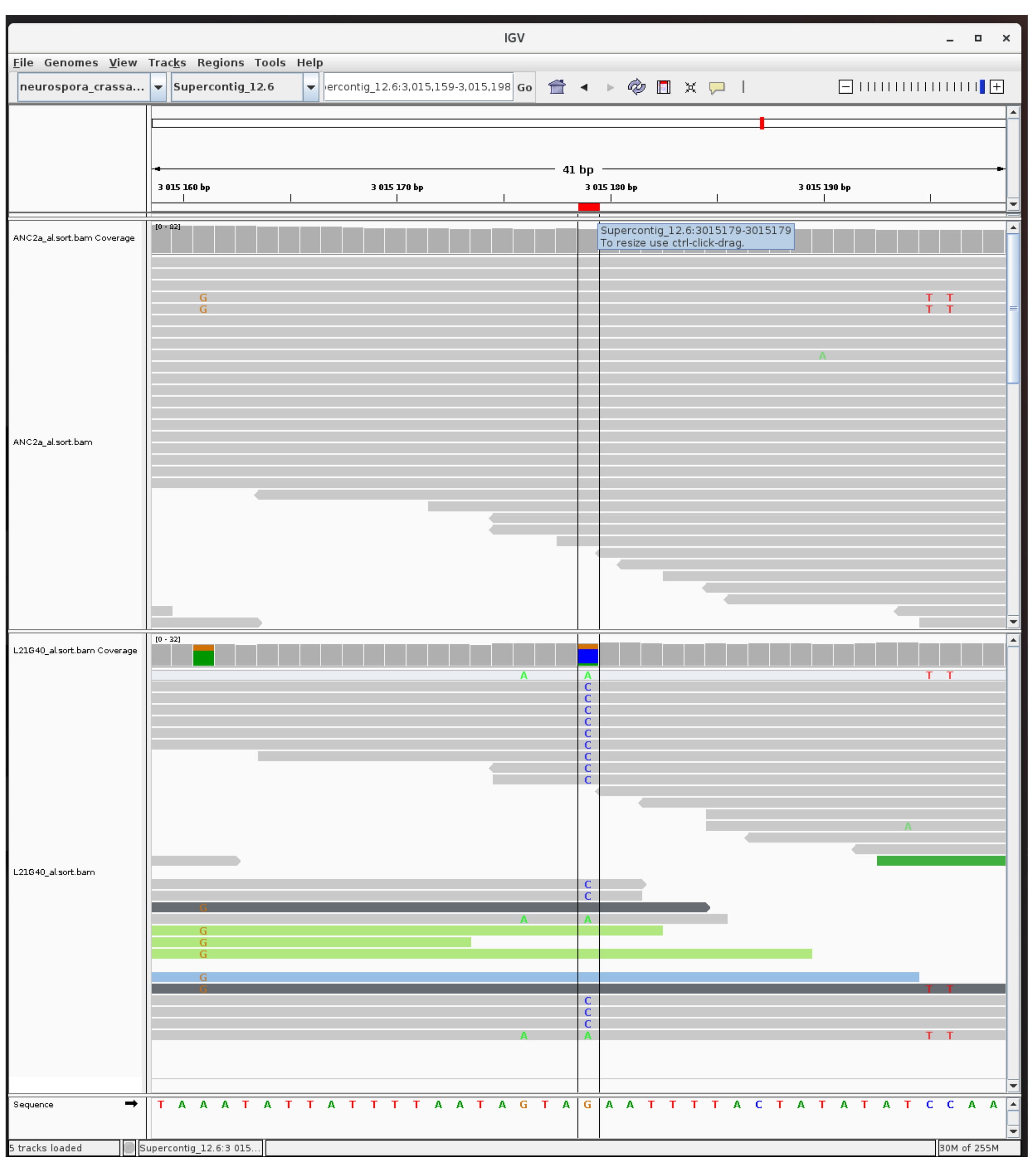

Supplement: Supplemental Material [file supp_gr.276992.122_Supplementary_file_S2.zip › IGV_screenshots/mutation_centromer_11.jpg]

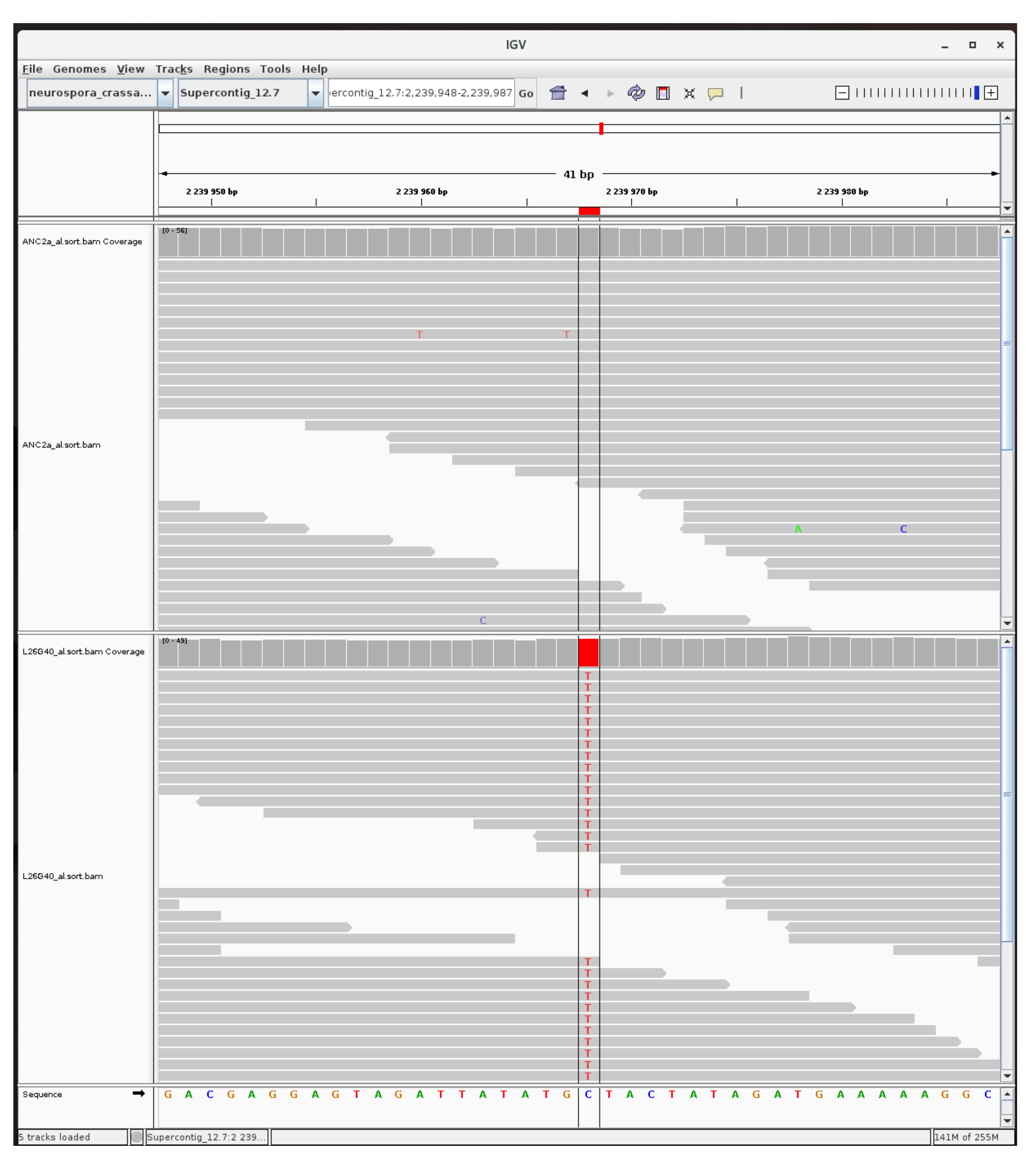

Supplement: Supplemental Material [file supp_gr.276992.122_Supplementary_file_S2.zip › IGV_screenshots/mutation_centromer_12.jpg]

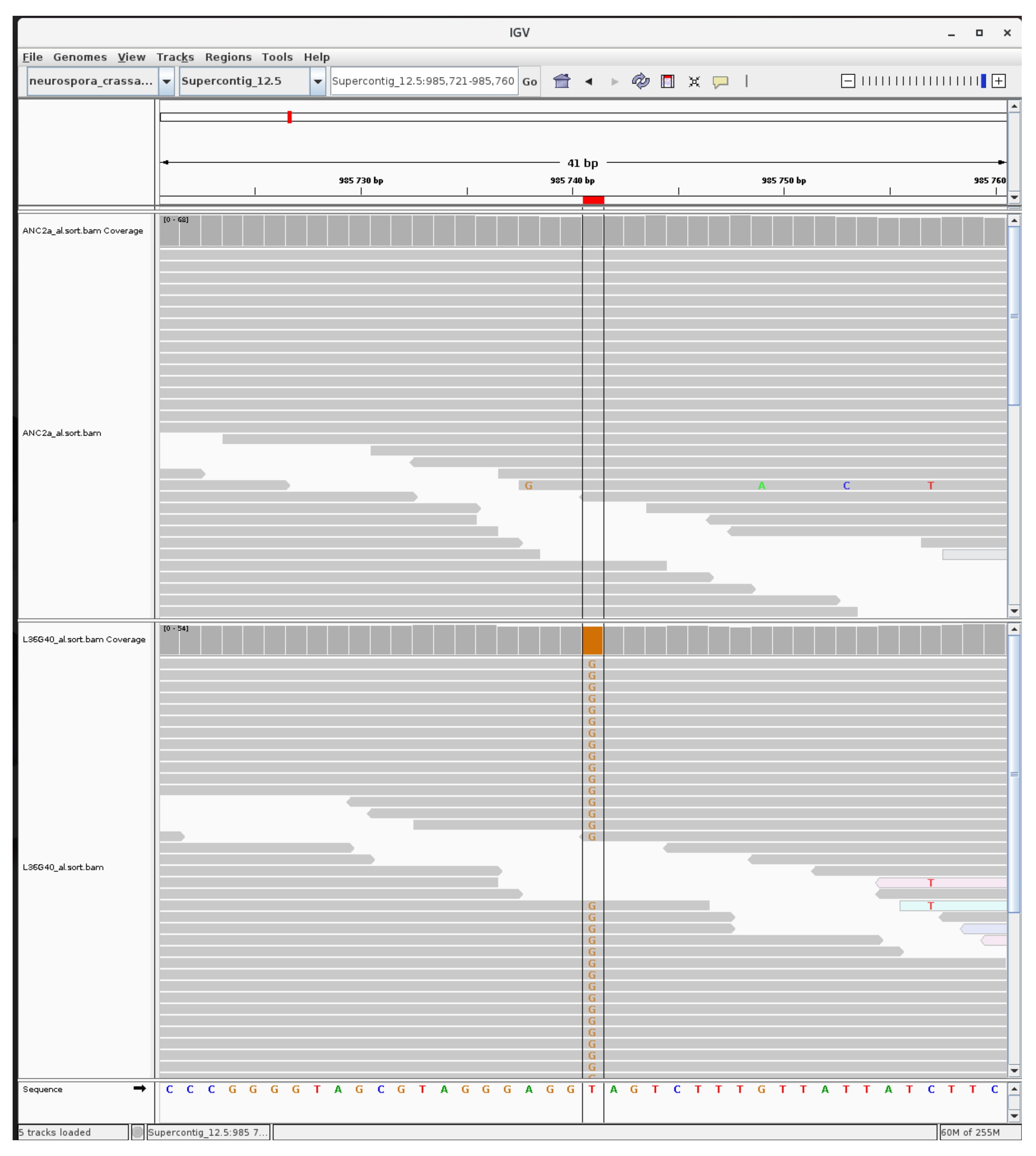

Supplement: Supplemental Material [file supp_gr.276992.122_Supplementary_file_S2.zip › IGV_screenshots/mutation_centromer_13.jpg]

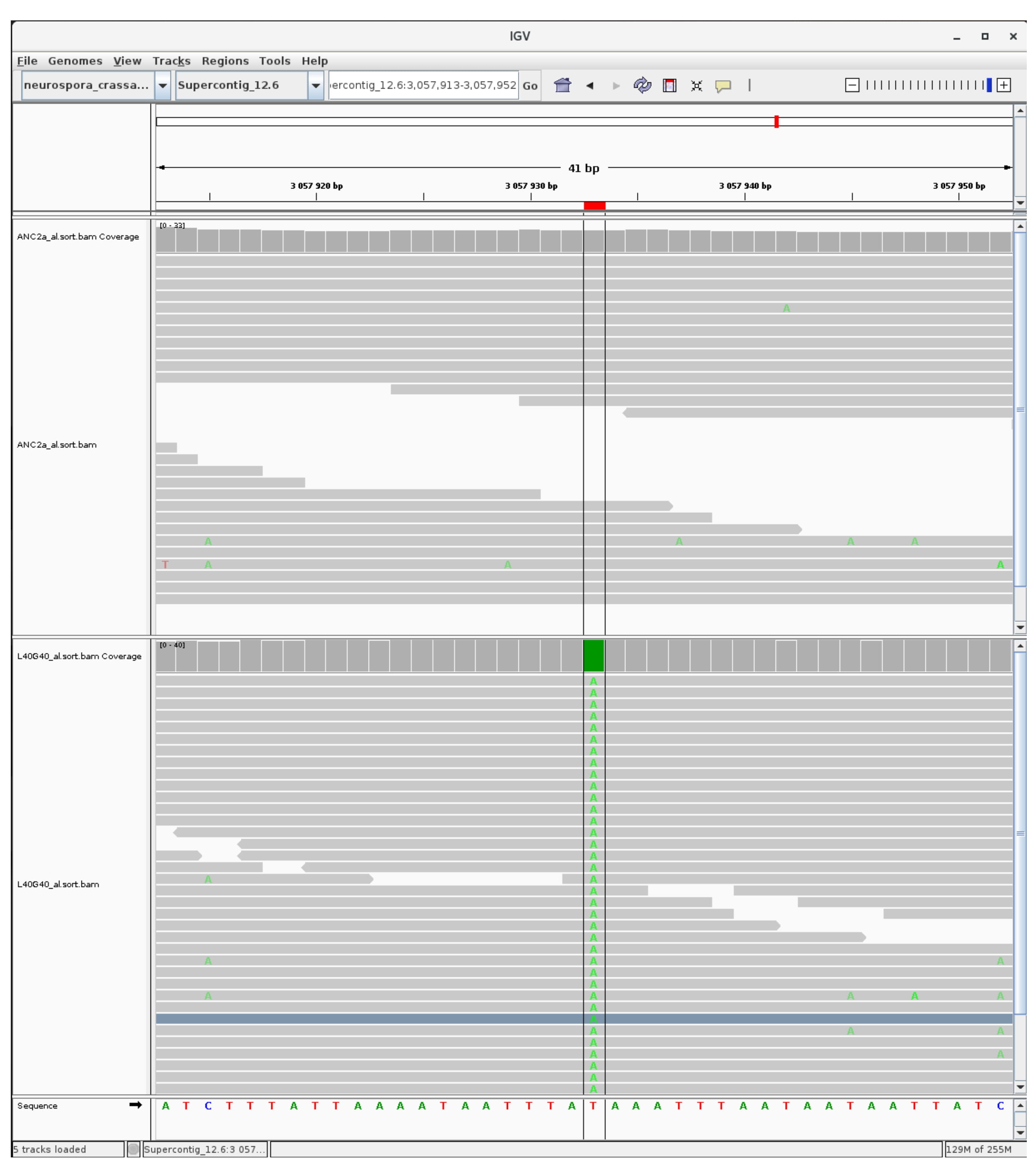

Supplement: Supplemental Material [file supp_gr.276992.122_Supplementary_file_S2.zip › IGV_screenshots/mutation_centromer_14.jpg]

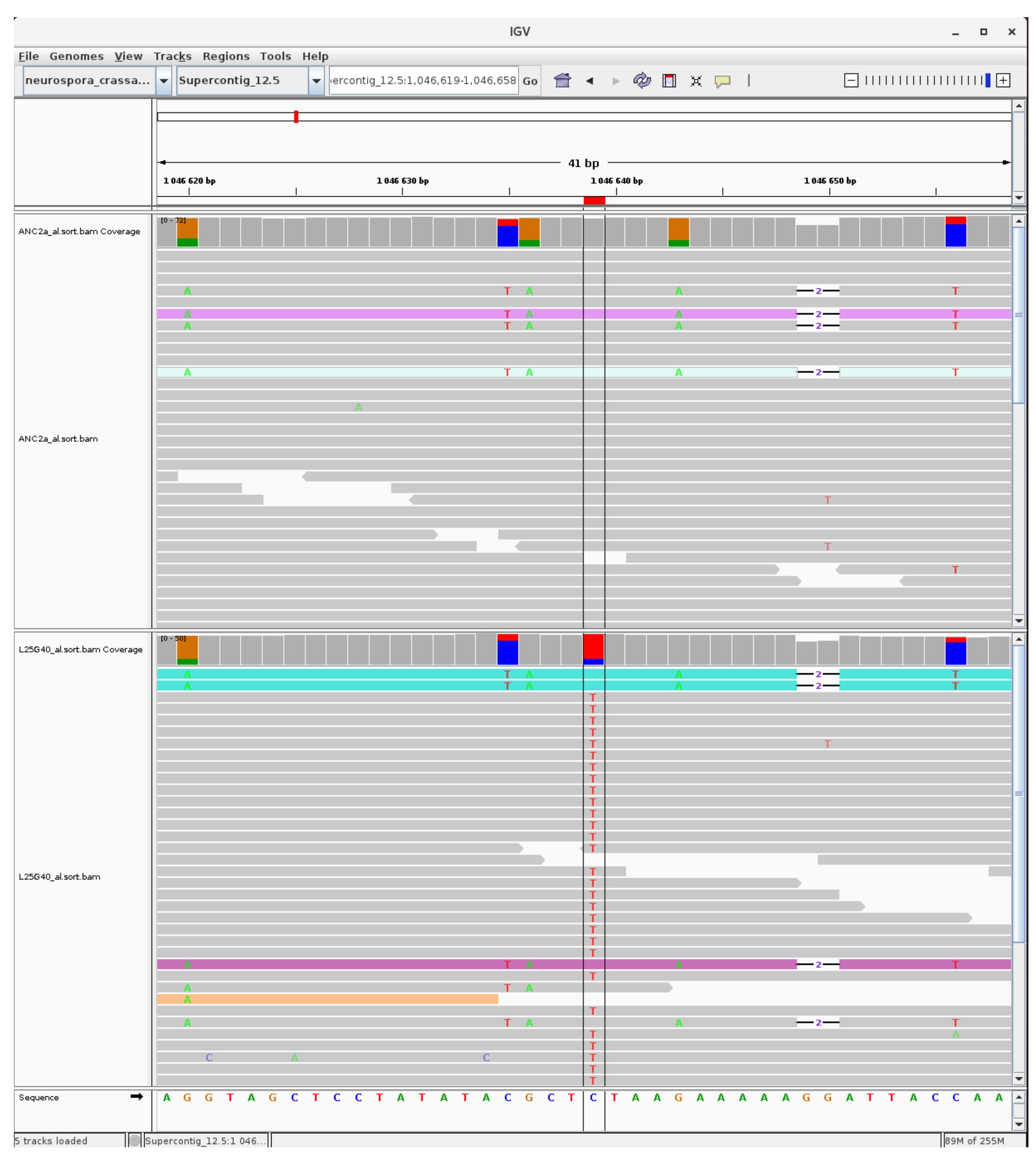

Supplement: Supplemental Material [file supp_gr.276992.122_Supplementary_file_S2.zip › IGV_screenshots/mutation_centromer_15.jpg]

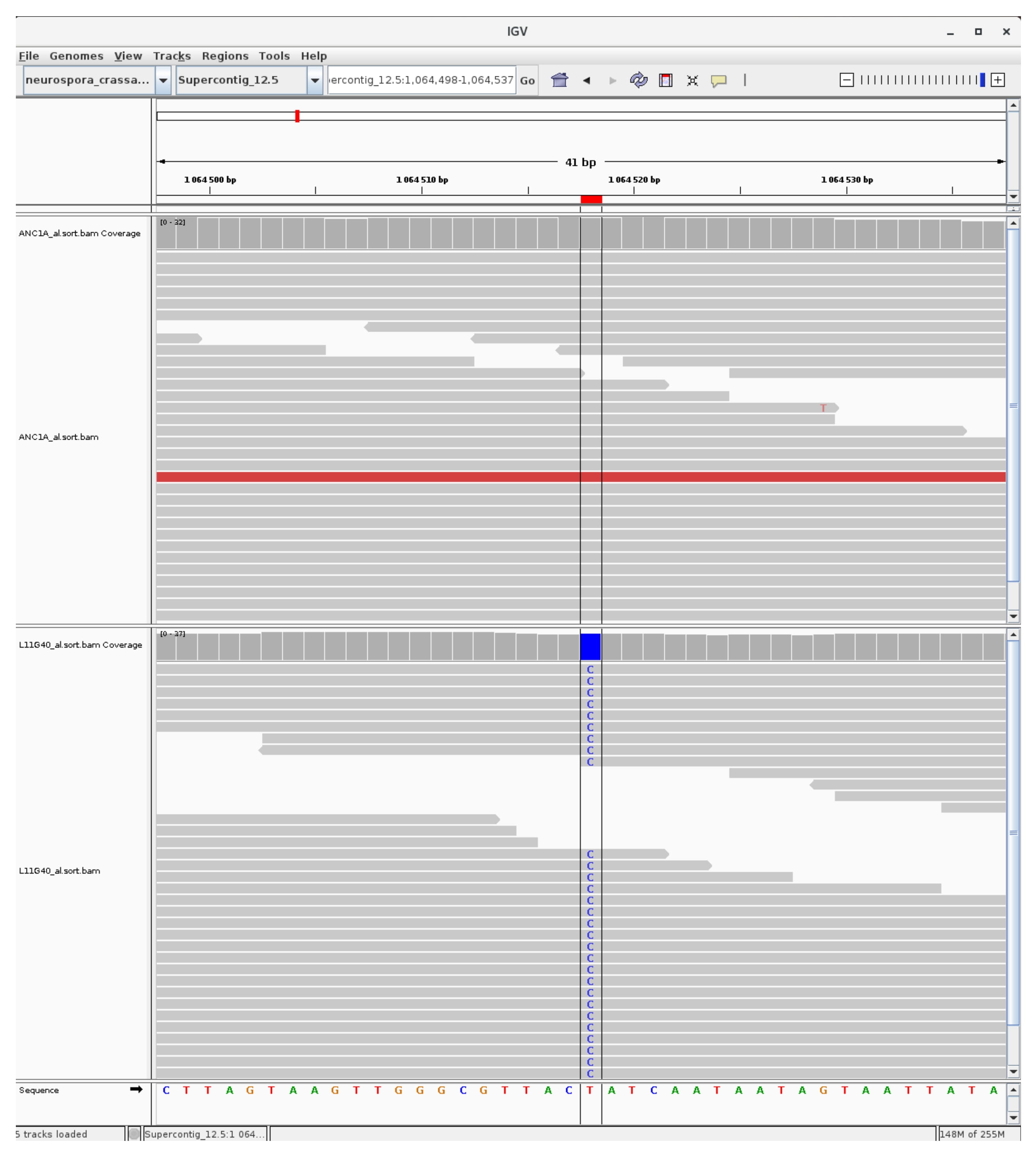

Supplement: Supplemental Material [file supp_gr.276992.122_Supplementary_file_S2.zip › IGV_screenshots/mutation_centromer_16.jpg]

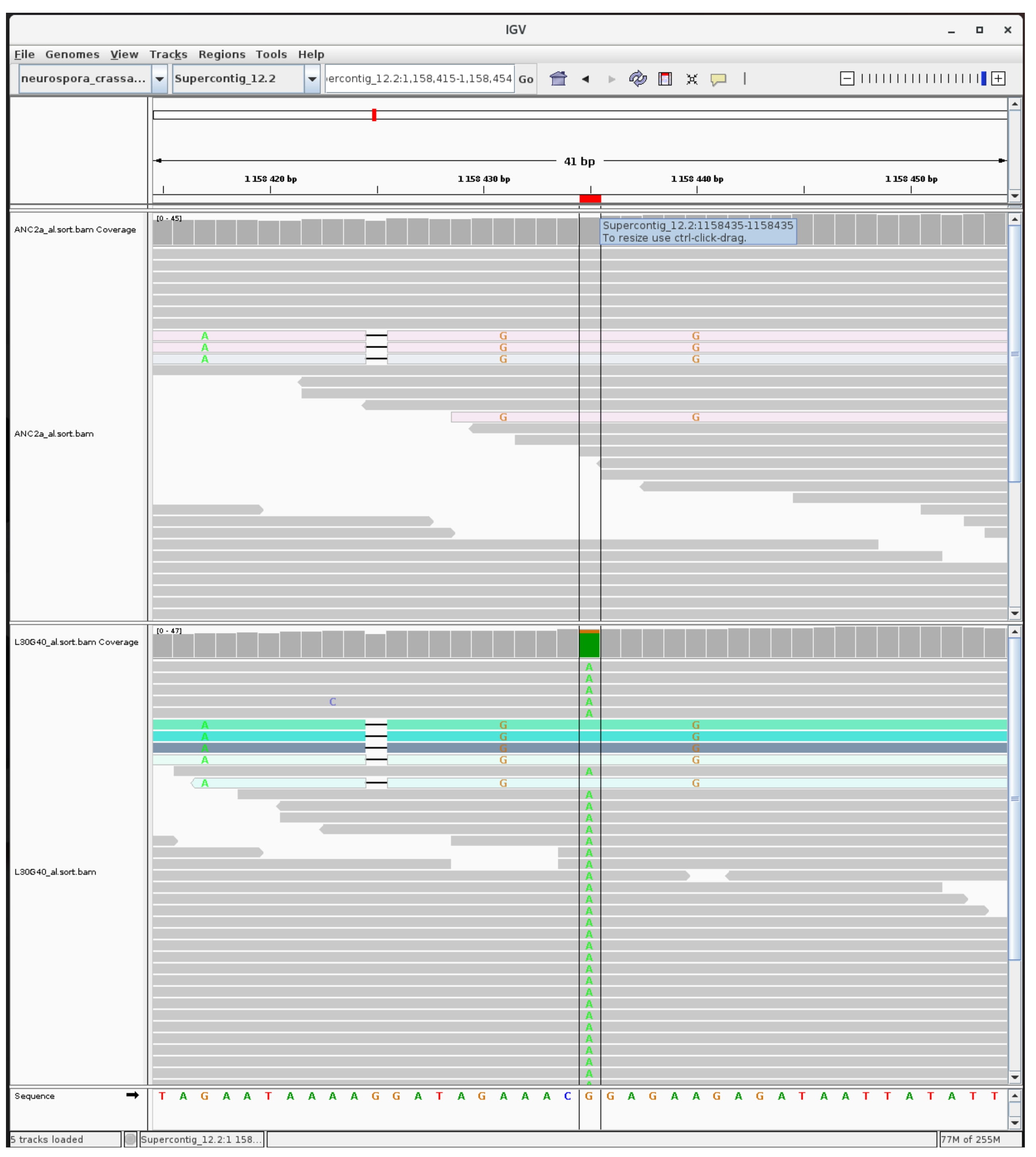

Supplement: Supplemental Material [file supp_gr.276992.122_Supplementary_file_S2.zip › IGV_screenshots/mutation_centromer_17.jpg]

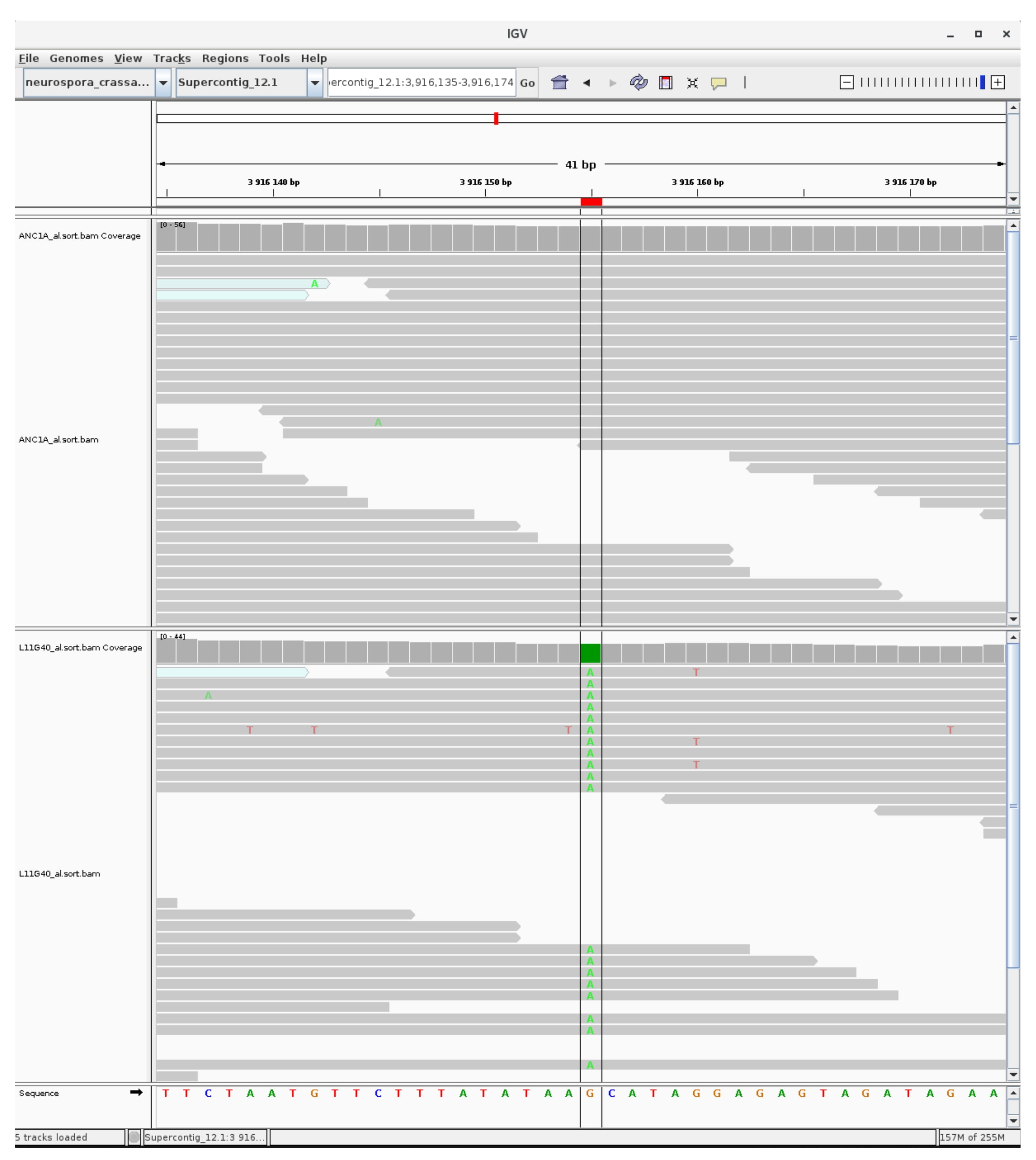

Supplement: Supplemental Material [file supp_gr.276992.122_Supplementary_file_S2.zip › IGV_screenshots/mutation_centromer_18.jpg]

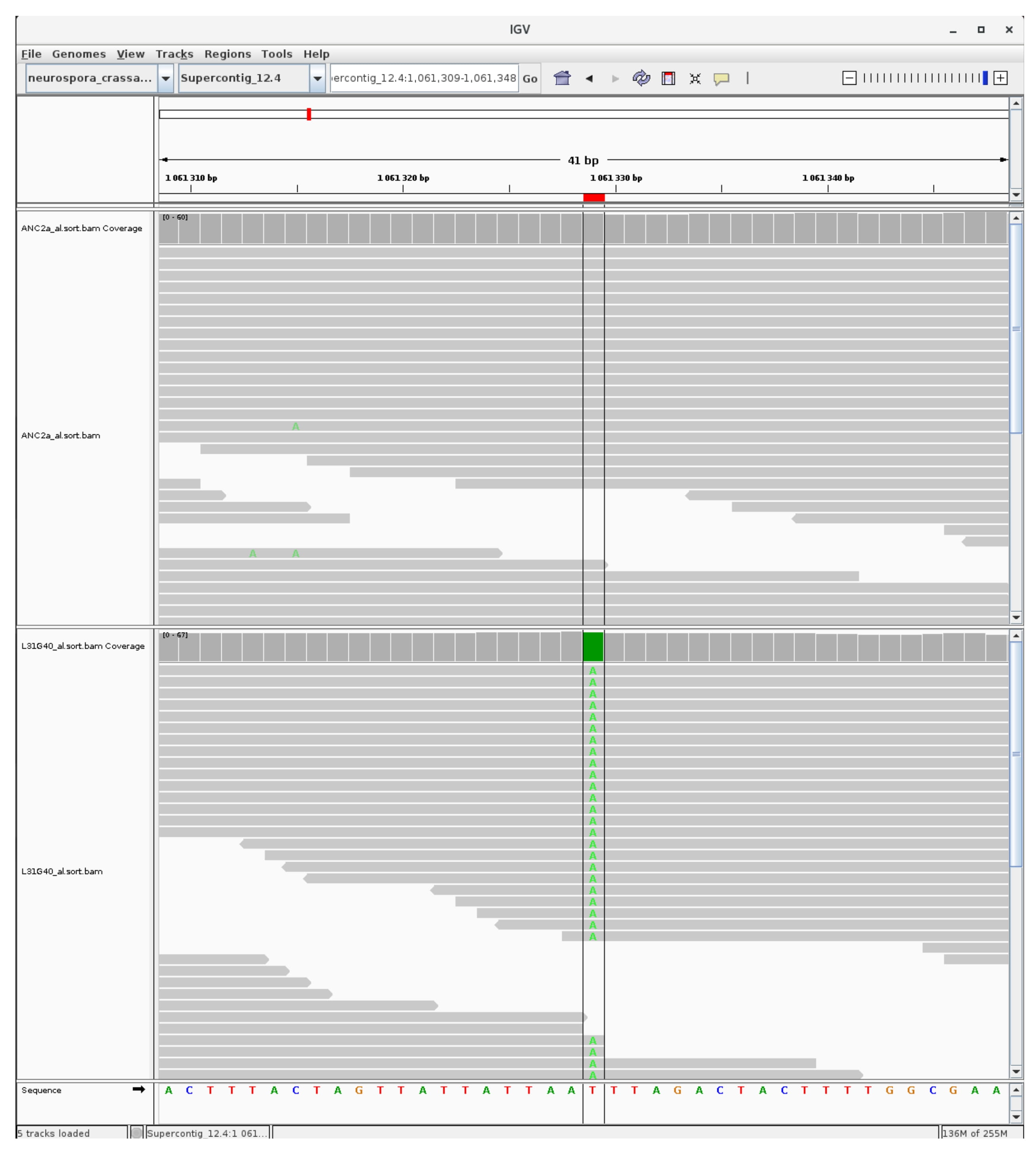

Supplement: Supplemental Material [file supp_gr.276992.122_Supplementary_file_S2.zip › IGV_screenshots/mutation_centromer_19.jpg]

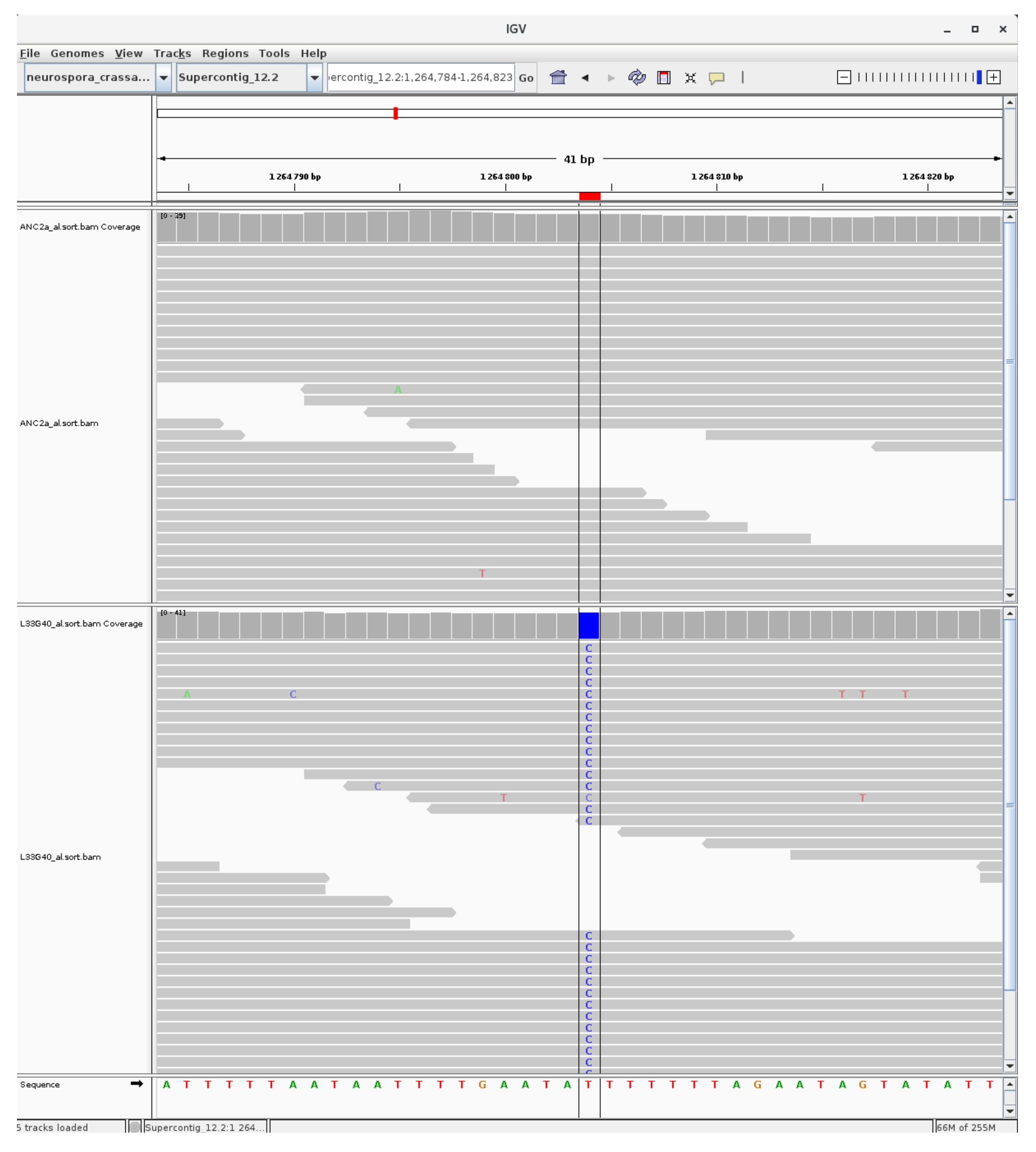

Supplement: Supplemental Material [file supp_gr.276992.122_Supplementary_file_S2.zip › IGV_screenshots/mutation_centromer_2.jpg]

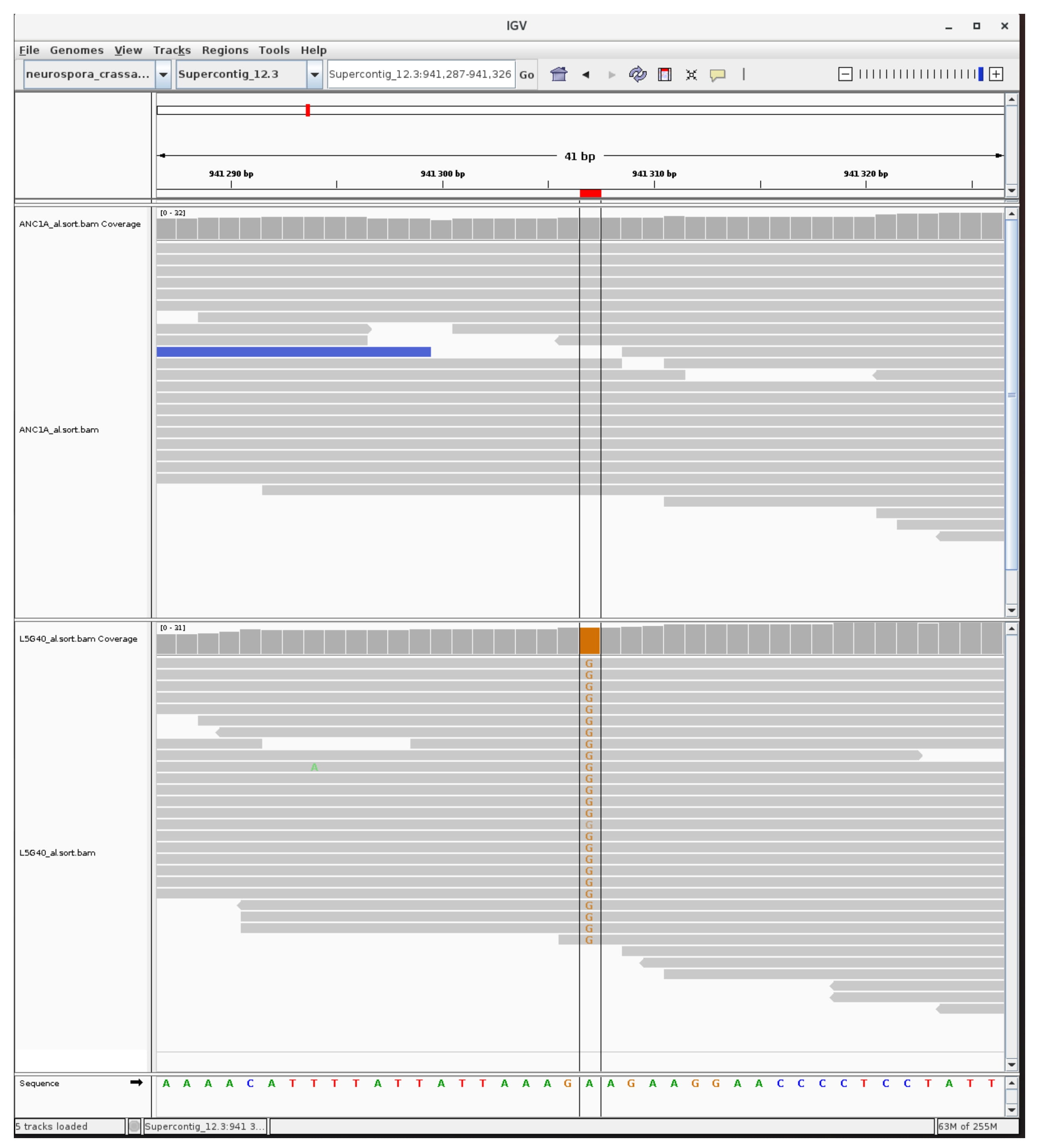

Supplement: Supplemental Material [file supp_gr.276992.122_Supplementary_file_S2.zip › IGV_screenshots/mutation_centromer_20.jpg]

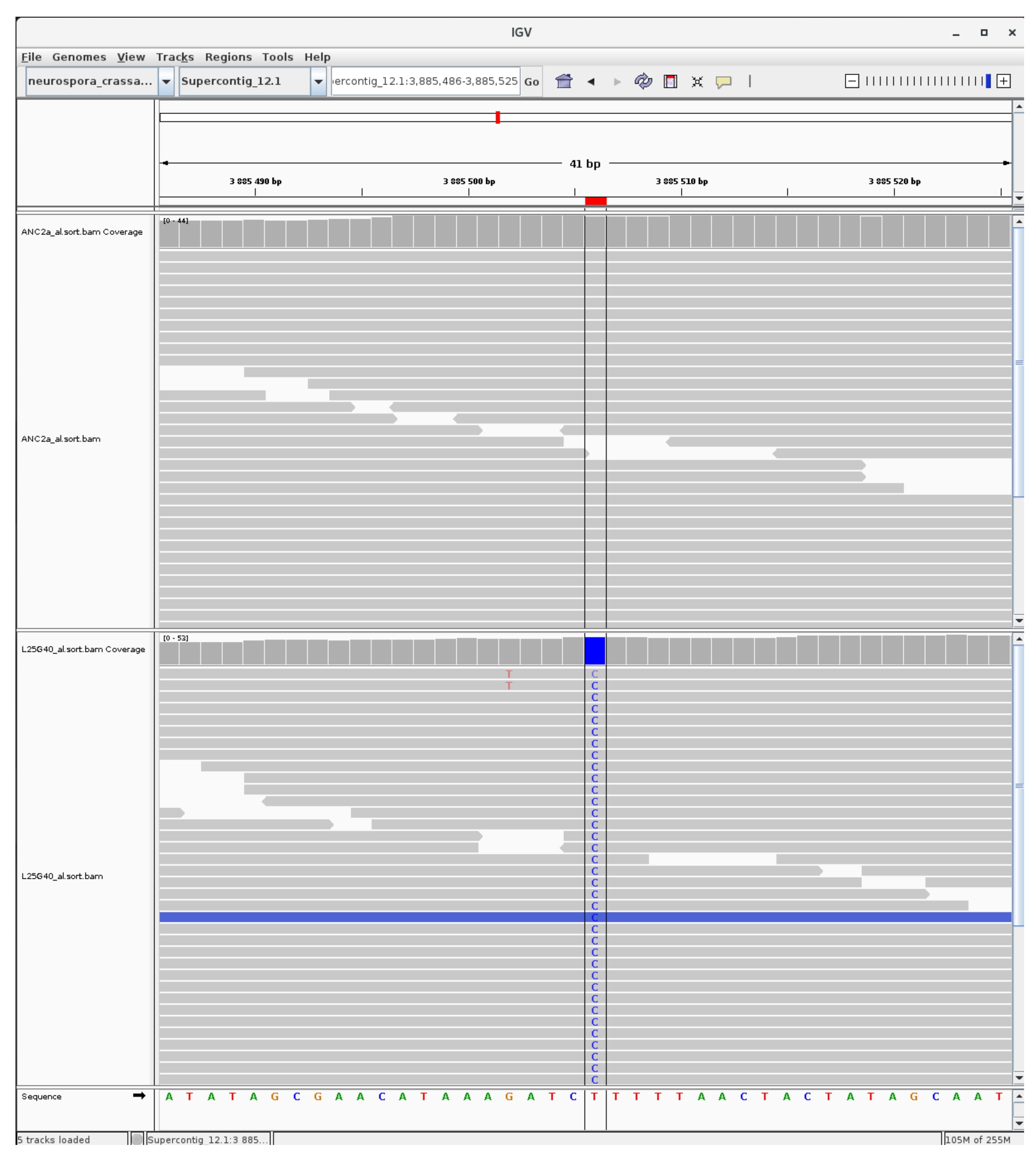

Supplement: Supplemental Material [file supp_gr.276992.122_Supplementary_file_S2.zip › IGV_screenshots/mutation_centromer_21.jpg]

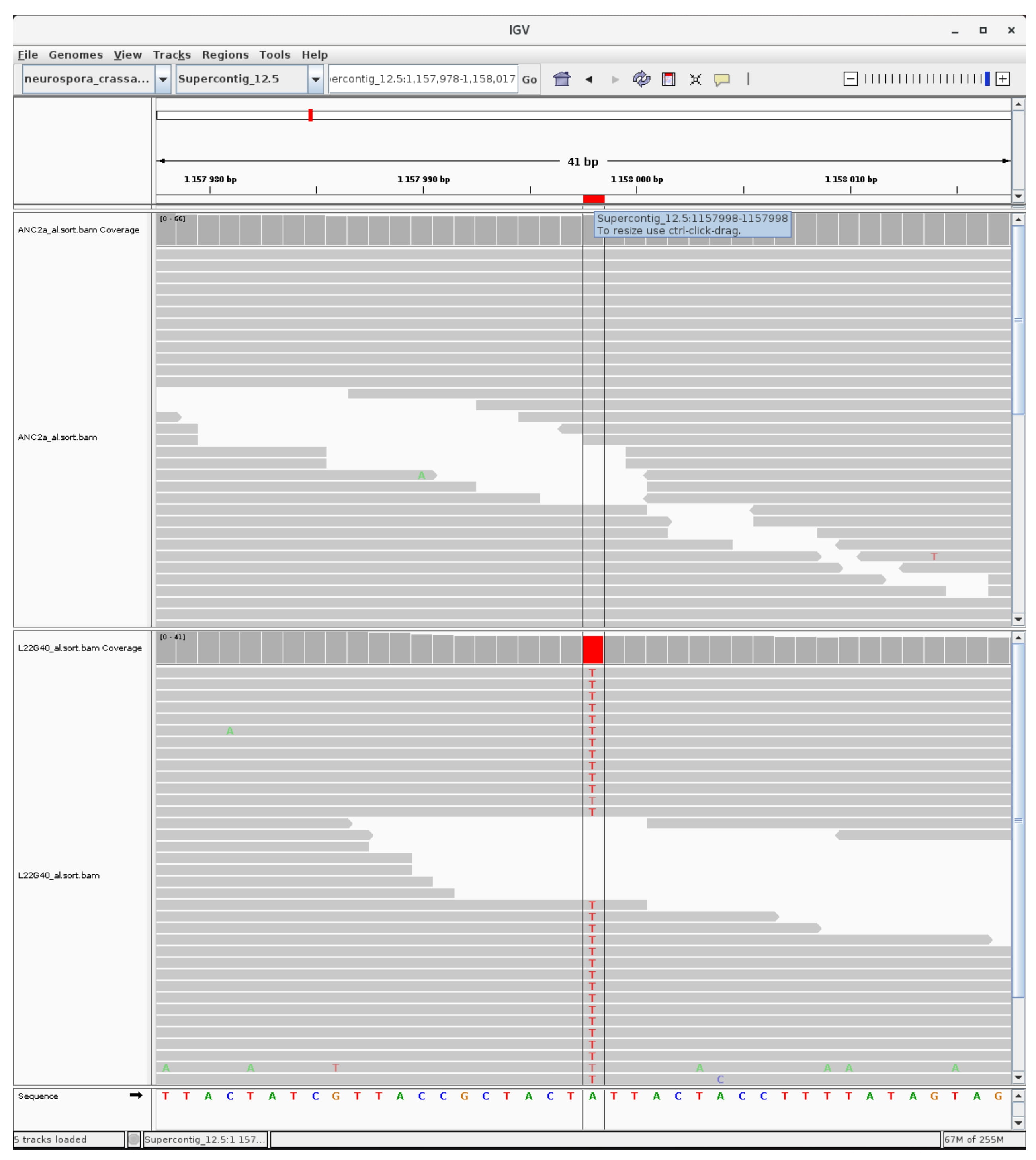

Supplement: Supplemental Material [file supp_gr.276992.122_Supplementary_file_S2.zip › IGV_screenshots/mutation_centromer_22.jpg]

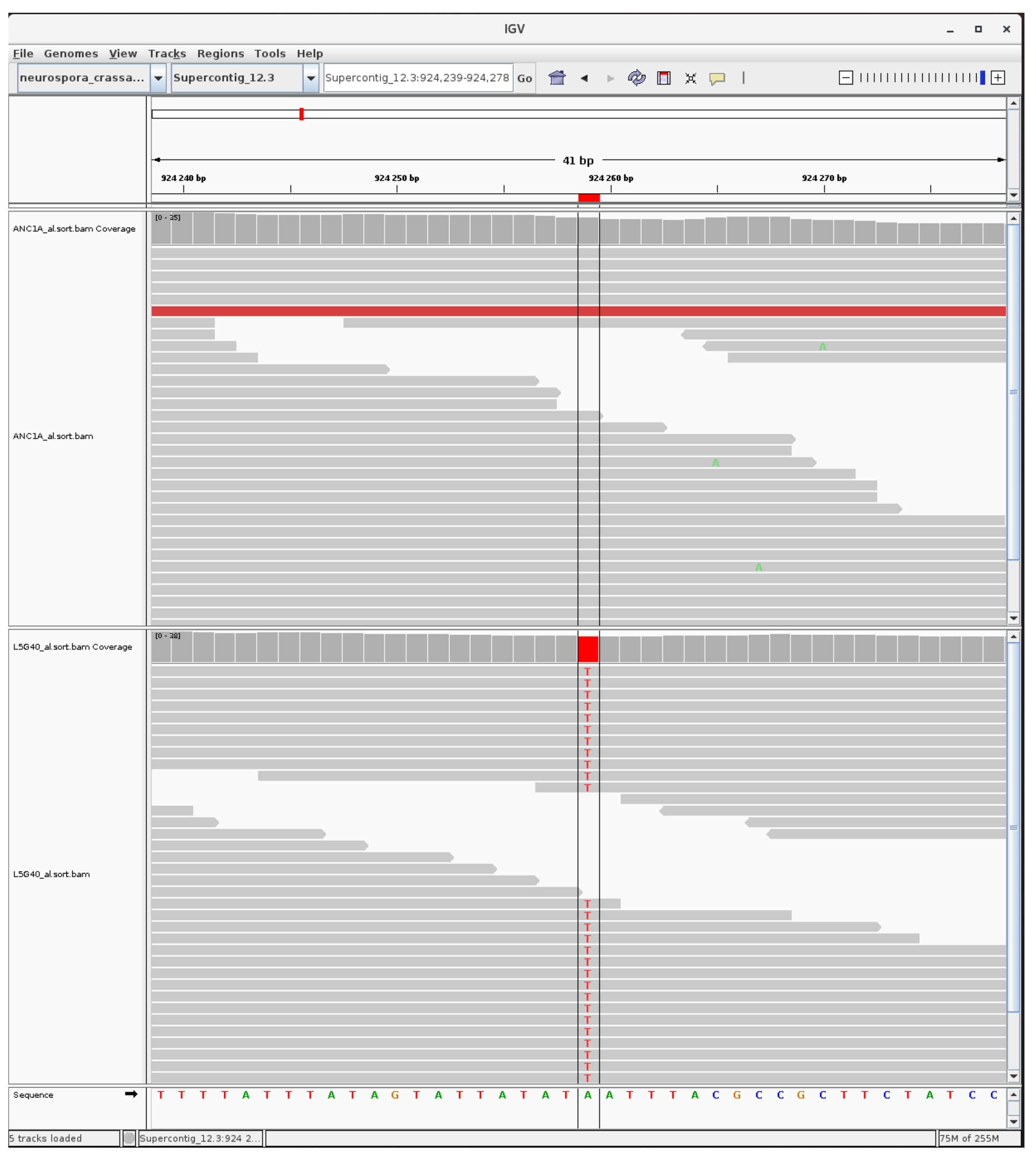

Supplement: Supplemental Material [file supp_gr.276992.122_Supplementary_file_S2.zip › IGV_screenshots/mutation_centromer_23.jpg]

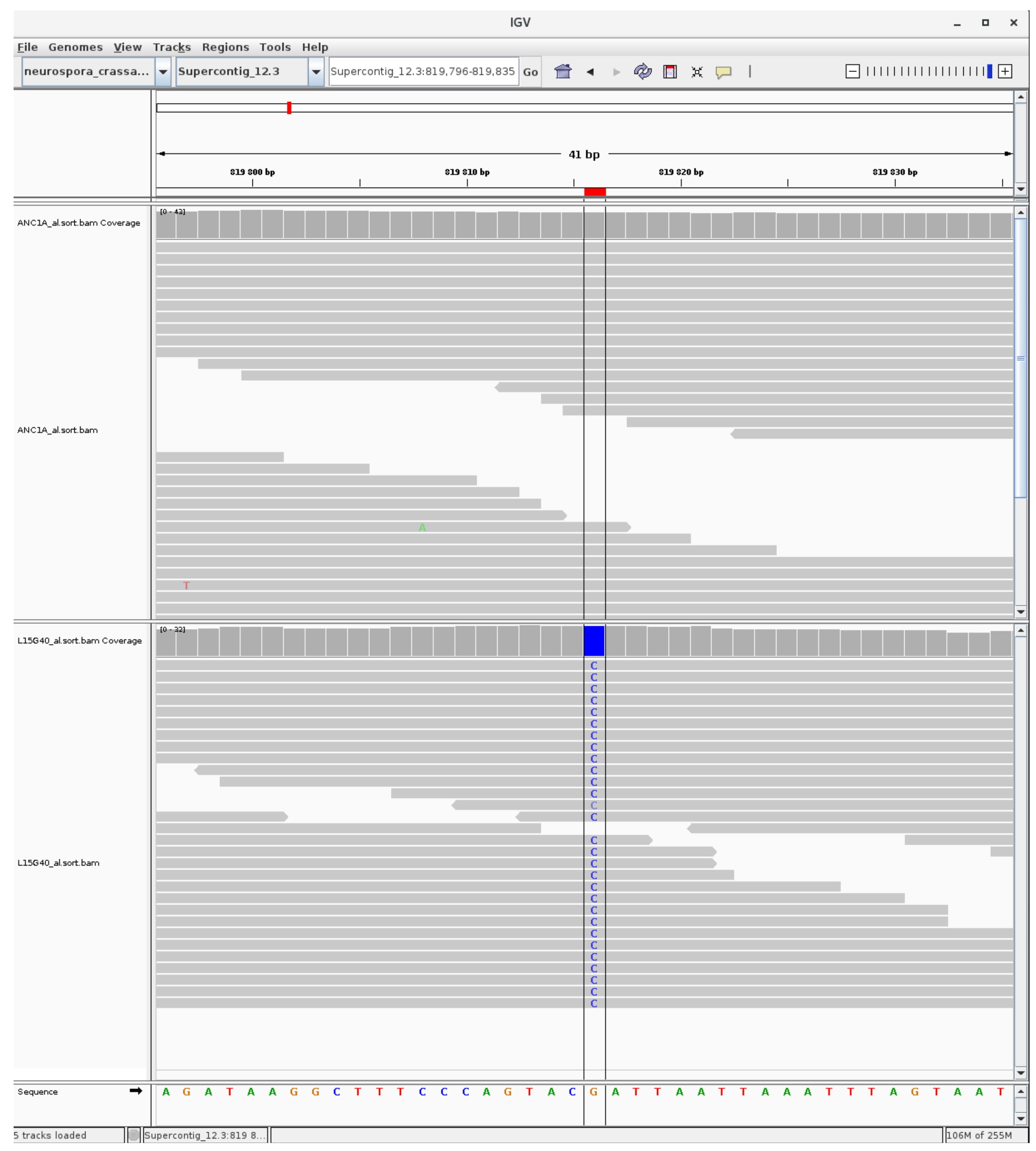

Supplement: Supplemental Material [file supp_gr.276992.122_Supplementary_file_S2.zip › IGV_screenshots/mutation_centromer_24.jpg]

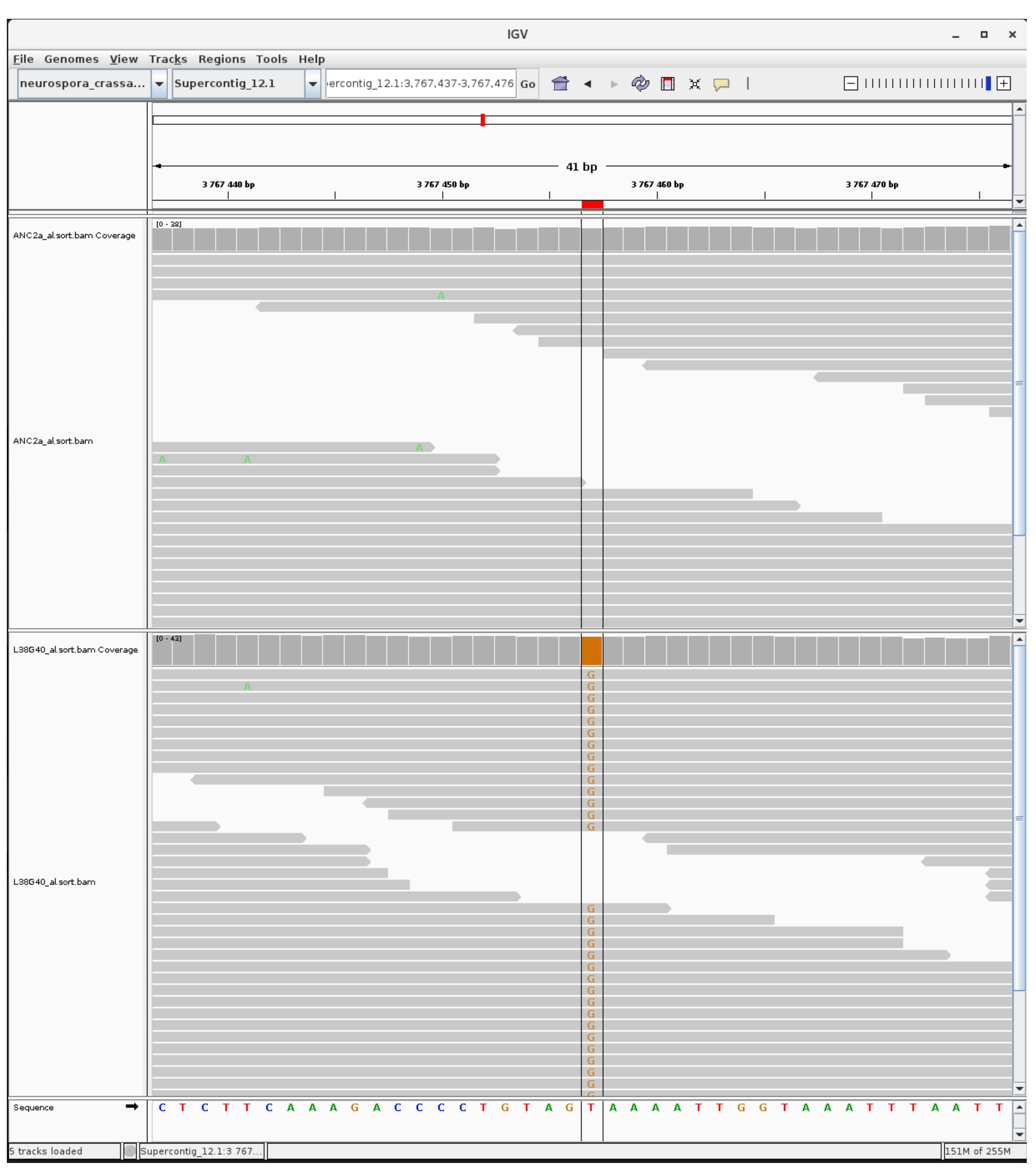

Supplement: Supplemental Material [file supp_gr.276992.122_Supplementary_file_S2.zip › IGV_screenshots/mutation_centromer_25.jpg]

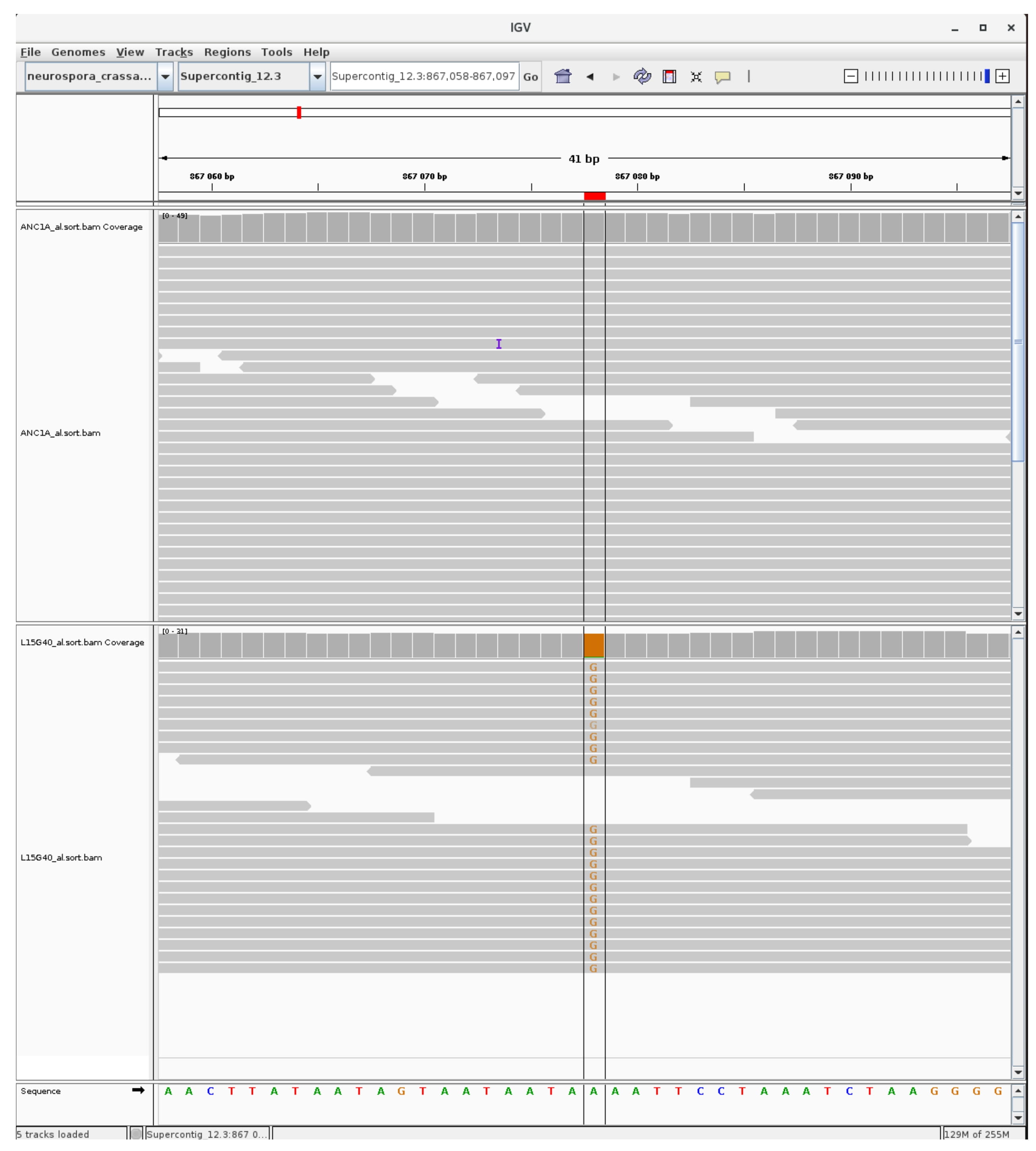

Supplement: Supplemental Material [file supp_gr.276992.122_Supplementary_file_S2.zip › IGV_screenshots/mutation_centromer_26.jpg]

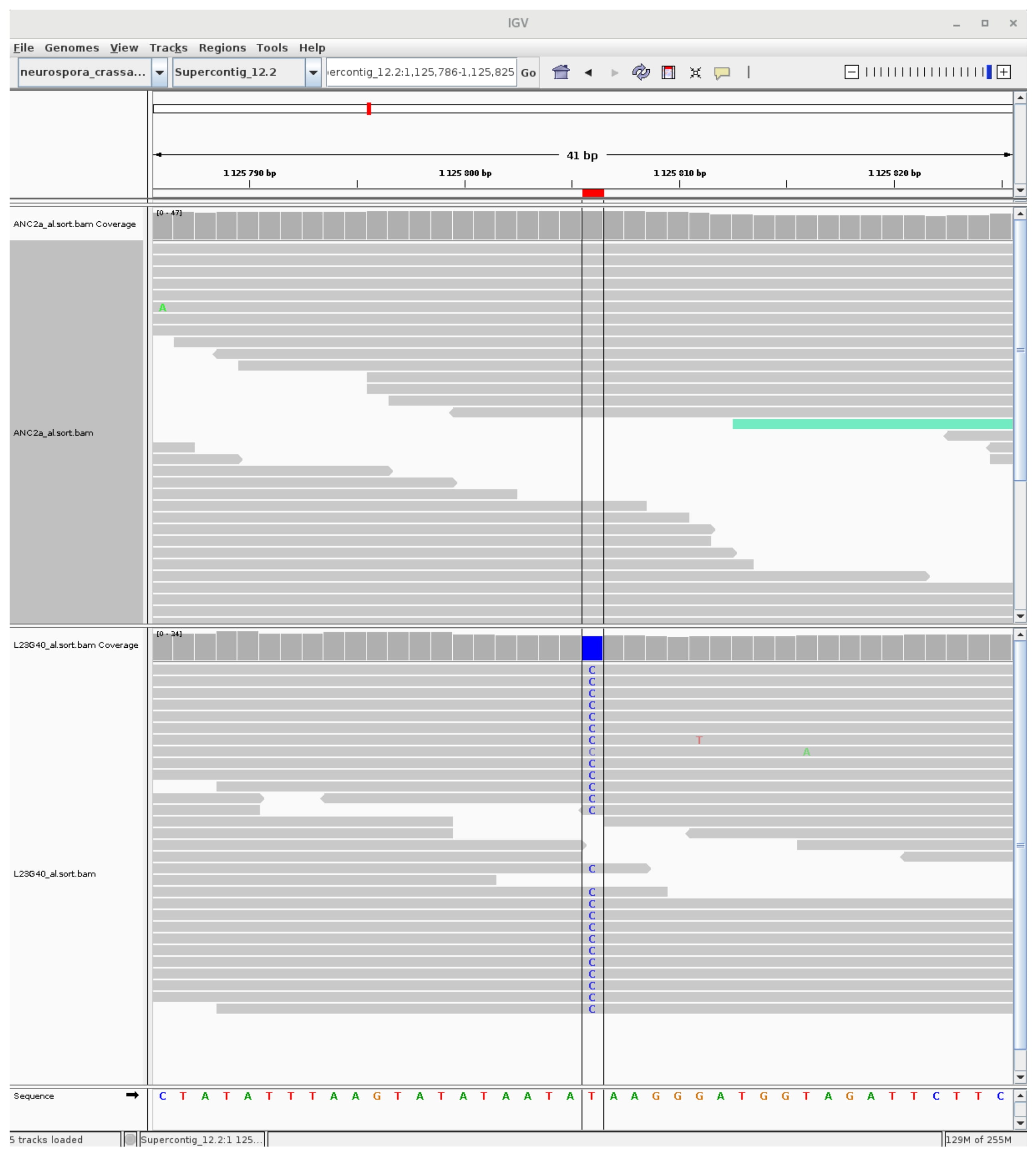

Supplement: Supplemental Material [file supp_gr.276992.122_Supplementary_file_S2.zip › IGV_screenshots/mutation_centromer_27.jpg]

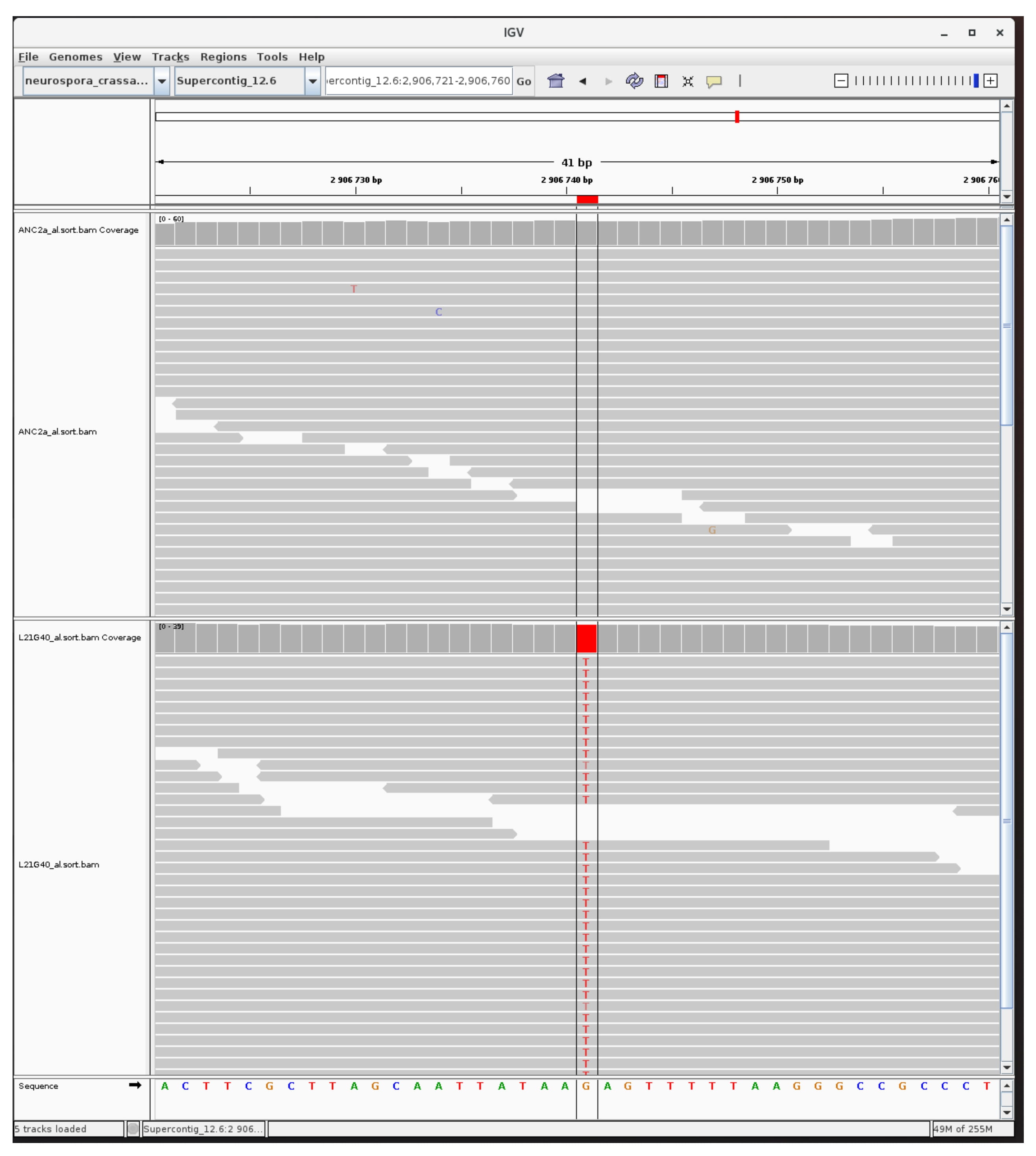

Supplement: Supplemental Material [file supp_gr.276992.122_Supplementary_file_S2.zip › IGV_screenshots/mutation_centromer_28.jpg]

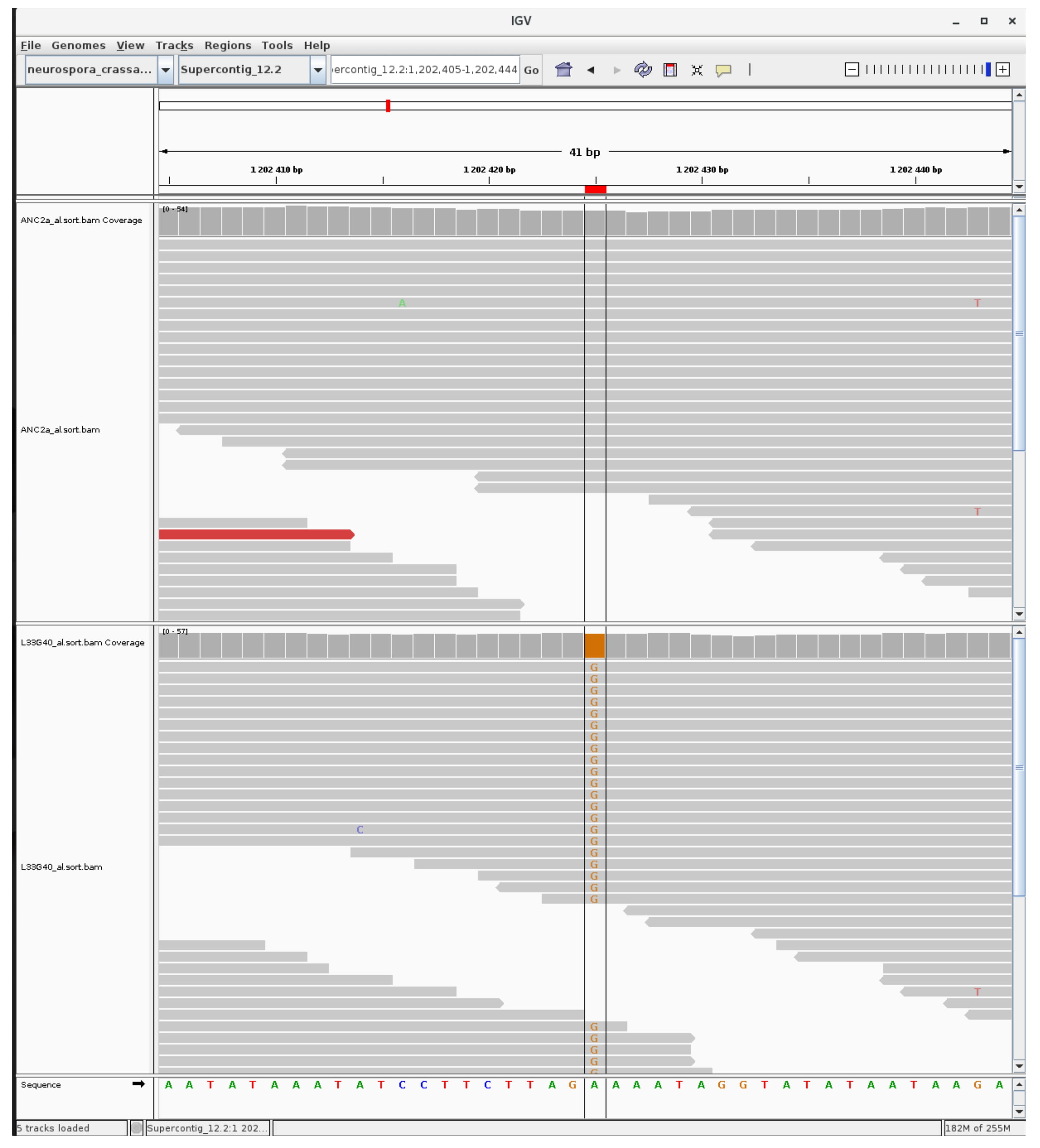

Supplement: Supplemental Material [file supp_gr.276992.122_Supplementary_file_S2.zip › IGV_screenshots/mutation_centromer_29.jpg]

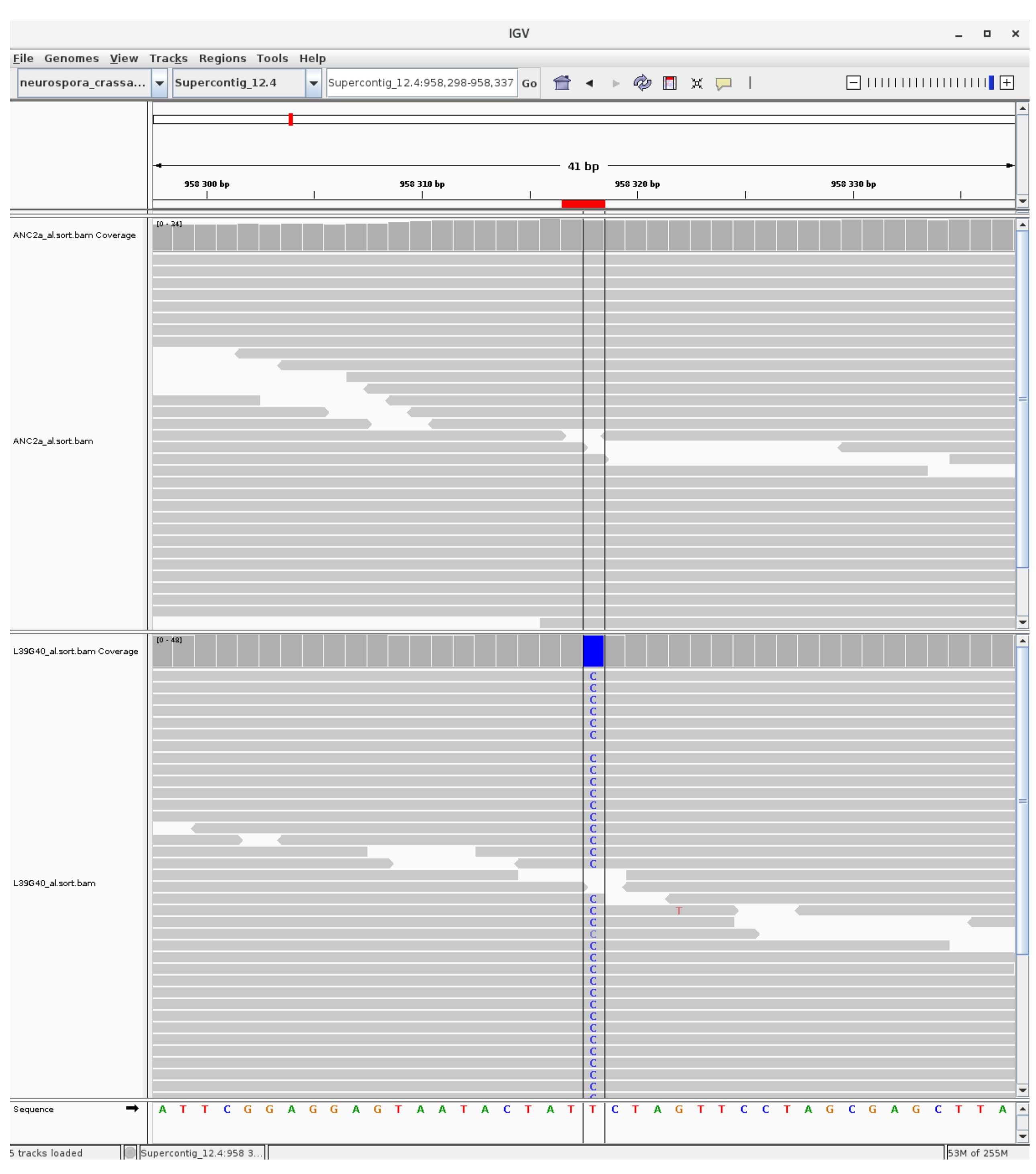

Supplement: Supplemental Material [file supp_gr.276992.122_Supplementary_file_S2.zip › IGV_screenshots/mutation_centromer_3.jpg]

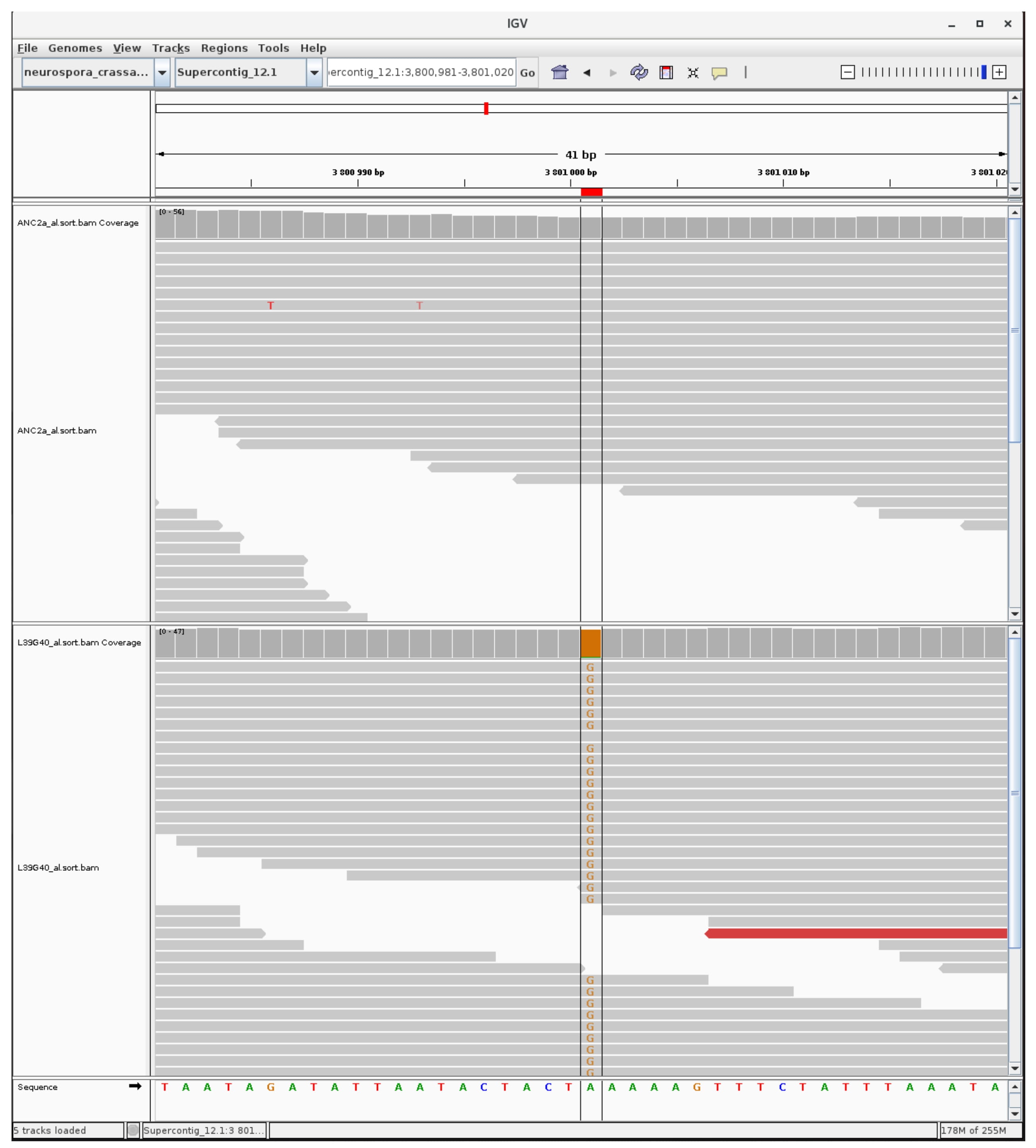

Supplement: Supplemental Material [file supp_gr.276992.122_Supplementary_file_S2.zip › IGV_screenshots/mutation_centromer_30.jpg]

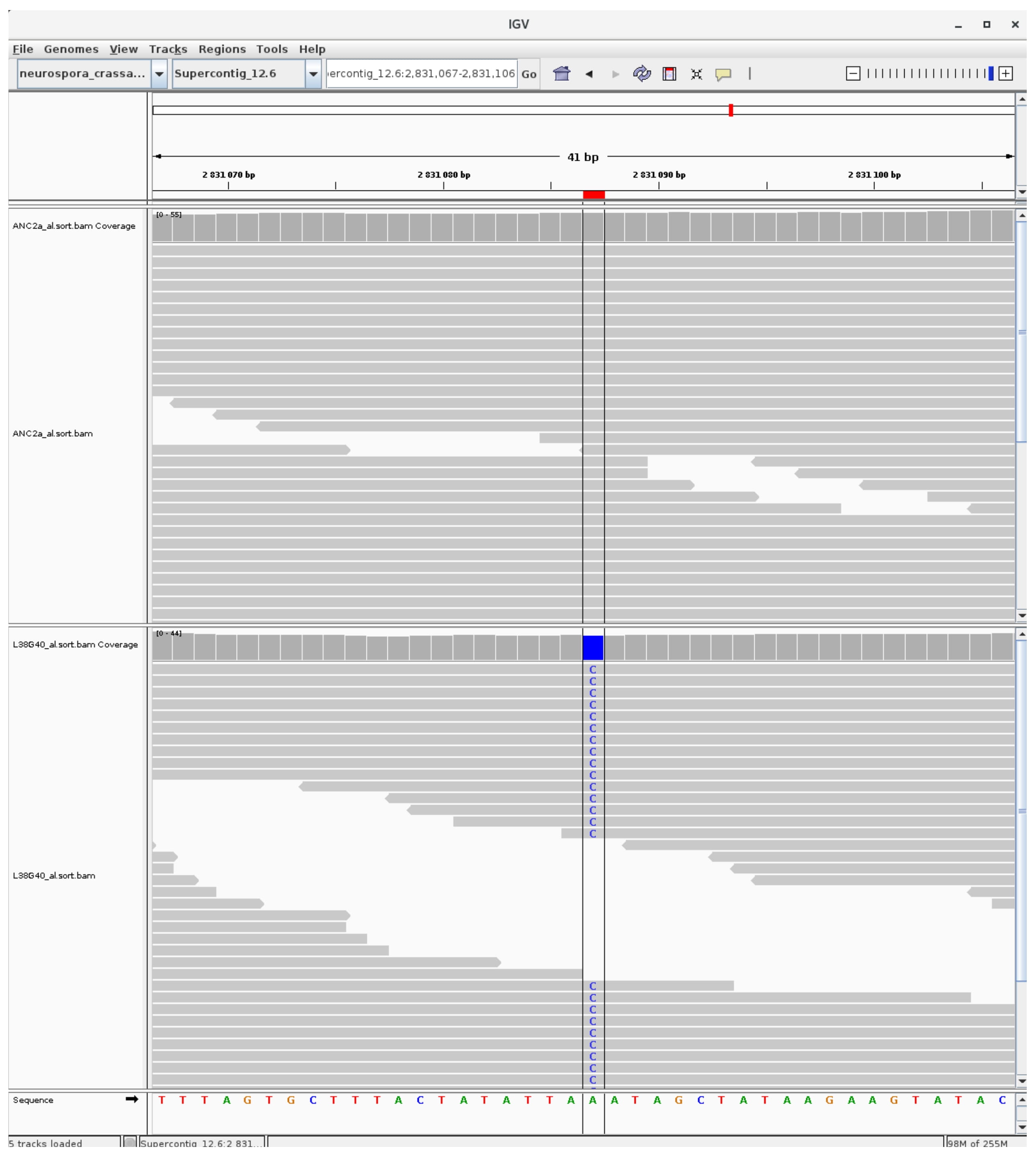

Supplement: Supplemental Material [file supp_gr.276992.122_Supplementary_file_S2.zip › IGV_screenshots/mutation_centromer_4.jpg]

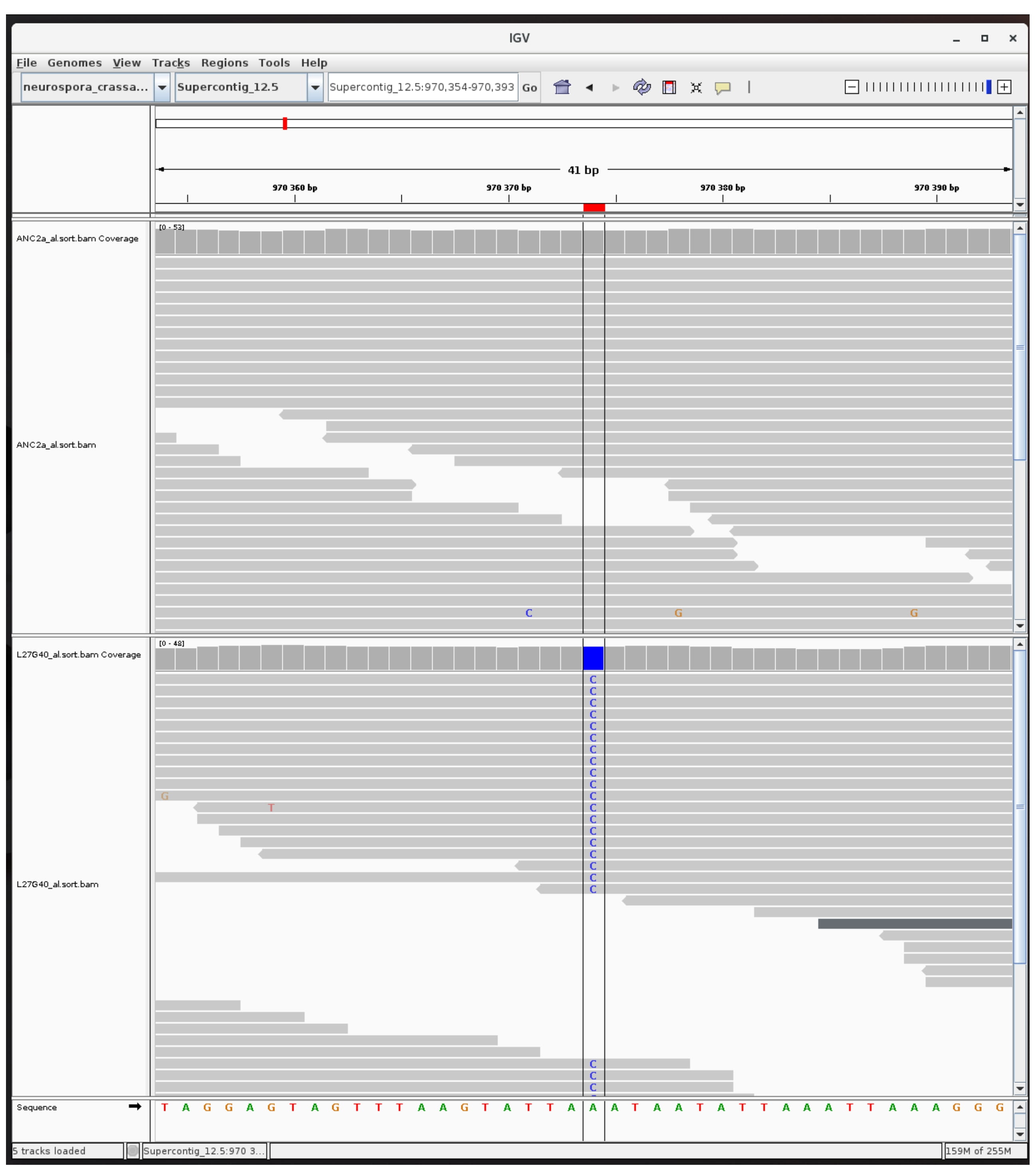

Supplement: Supplemental Material [file supp_gr.276992.122_Supplementary_file_S2.zip › IGV_screenshots/mutation_centromer_5.jpg]

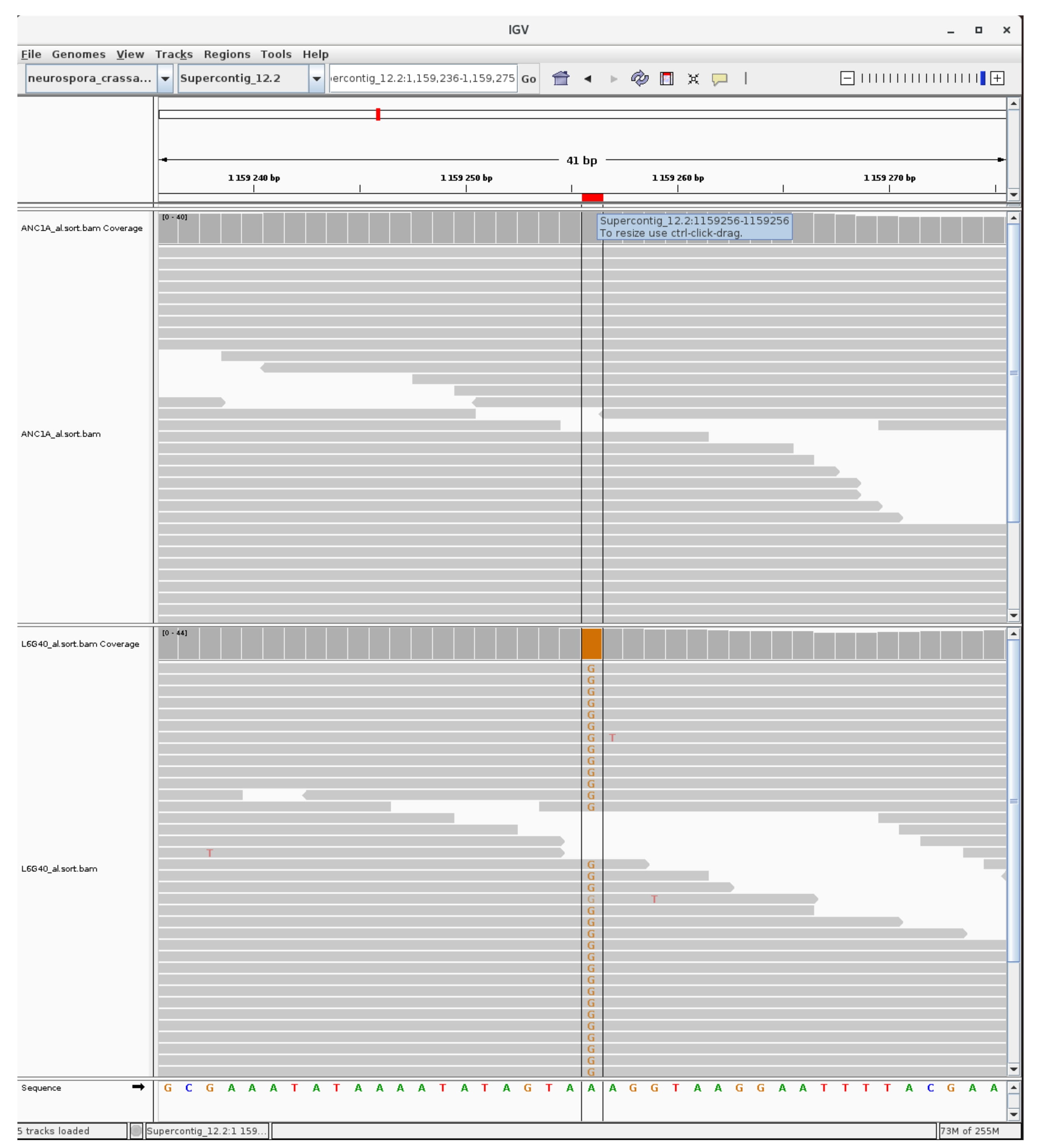

Supplement: Supplemental Material [file supp_gr.276992.122_Supplementary_file_S2.zip › IGV_screenshots/mutation_centromer_6.jpg]

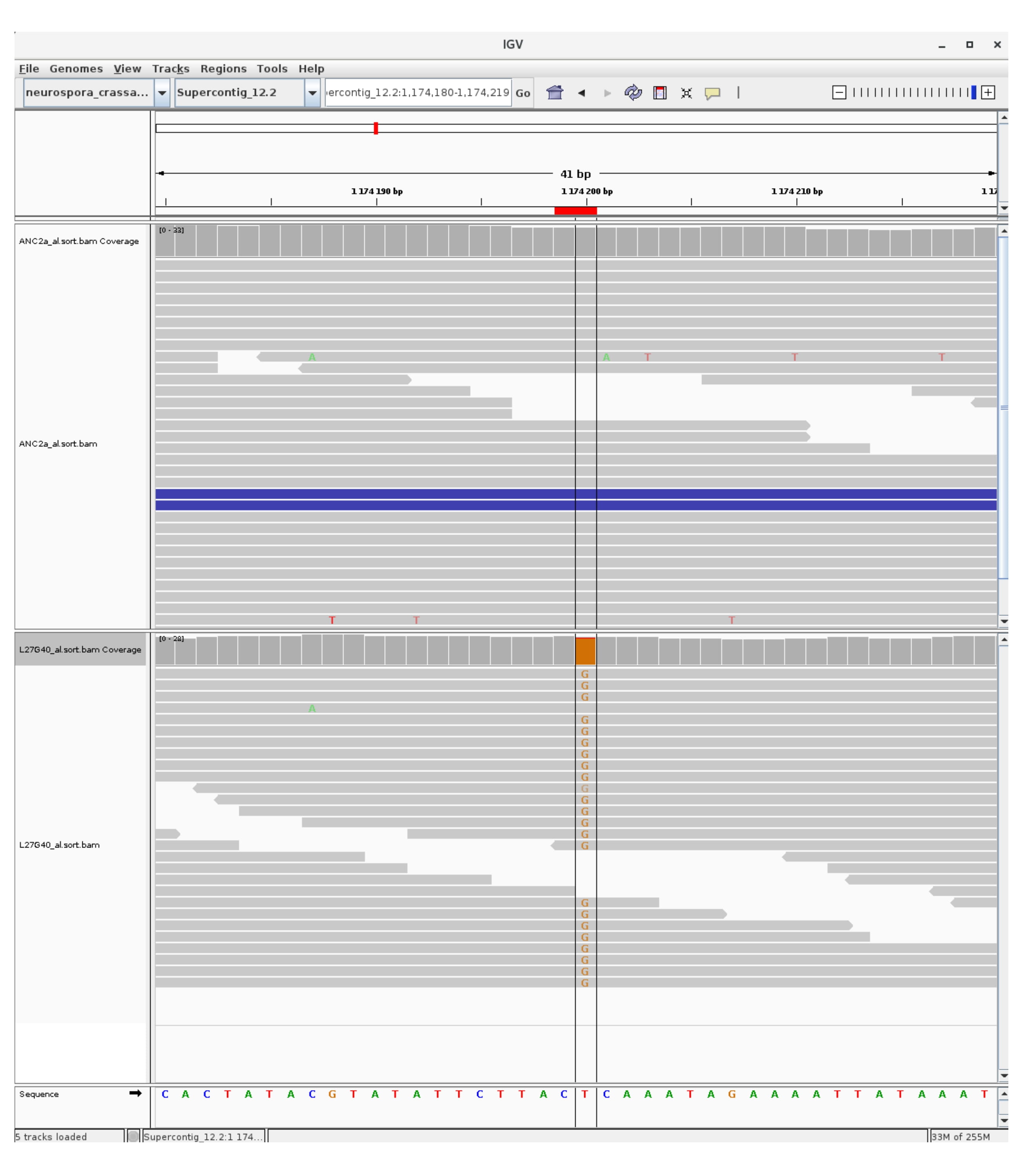

Supplement: Supplemental Material [file supp_gr.276992.122_Supplementary_file_S2.zip › IGV_screenshots/mutation_centromer_7.jpg]

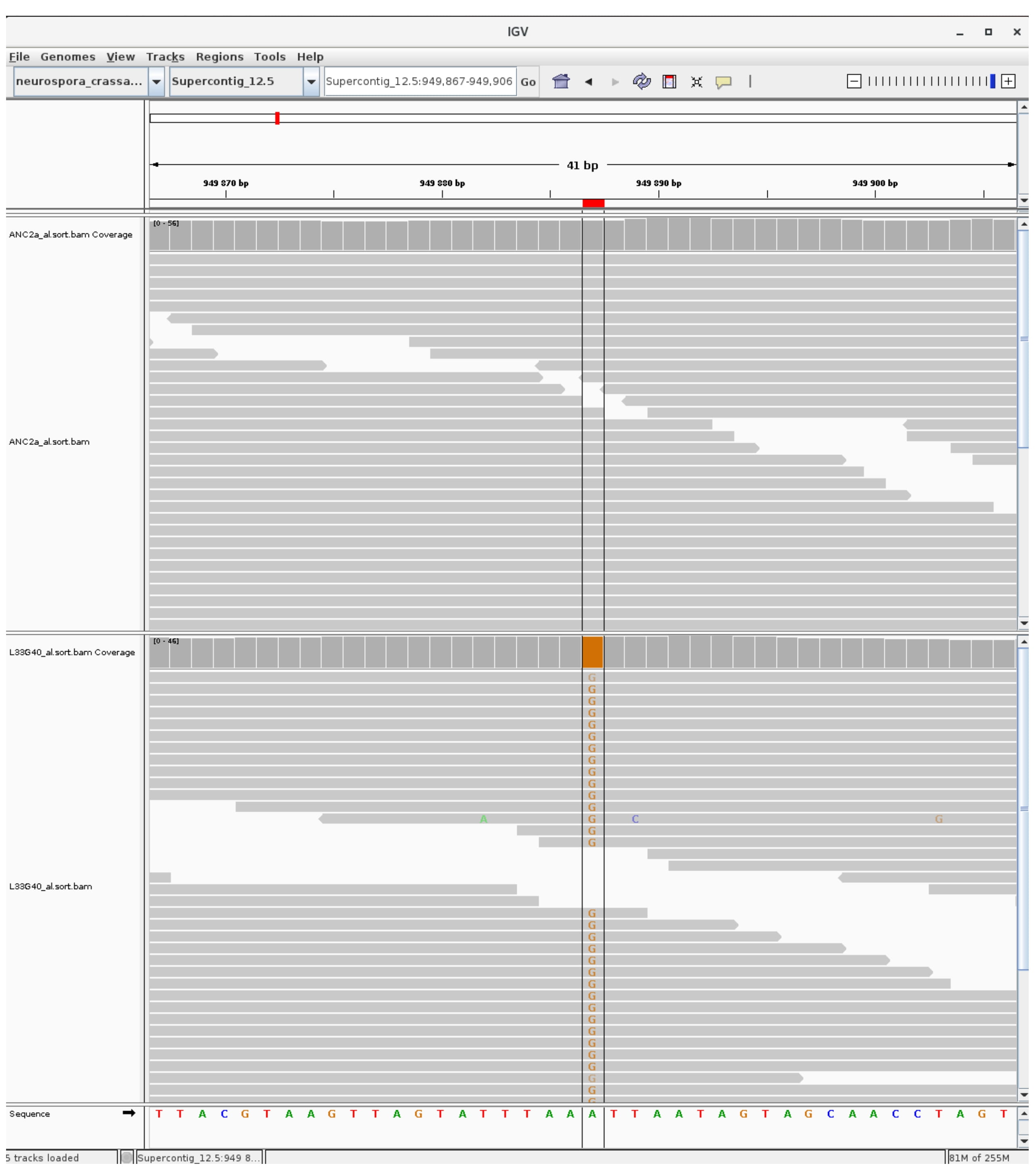

Supplement: Supplemental Material [file supp_gr.276992.122_Supplementary_file_S2.zip › IGV_screenshots/mutation_centromer_8.jpg]

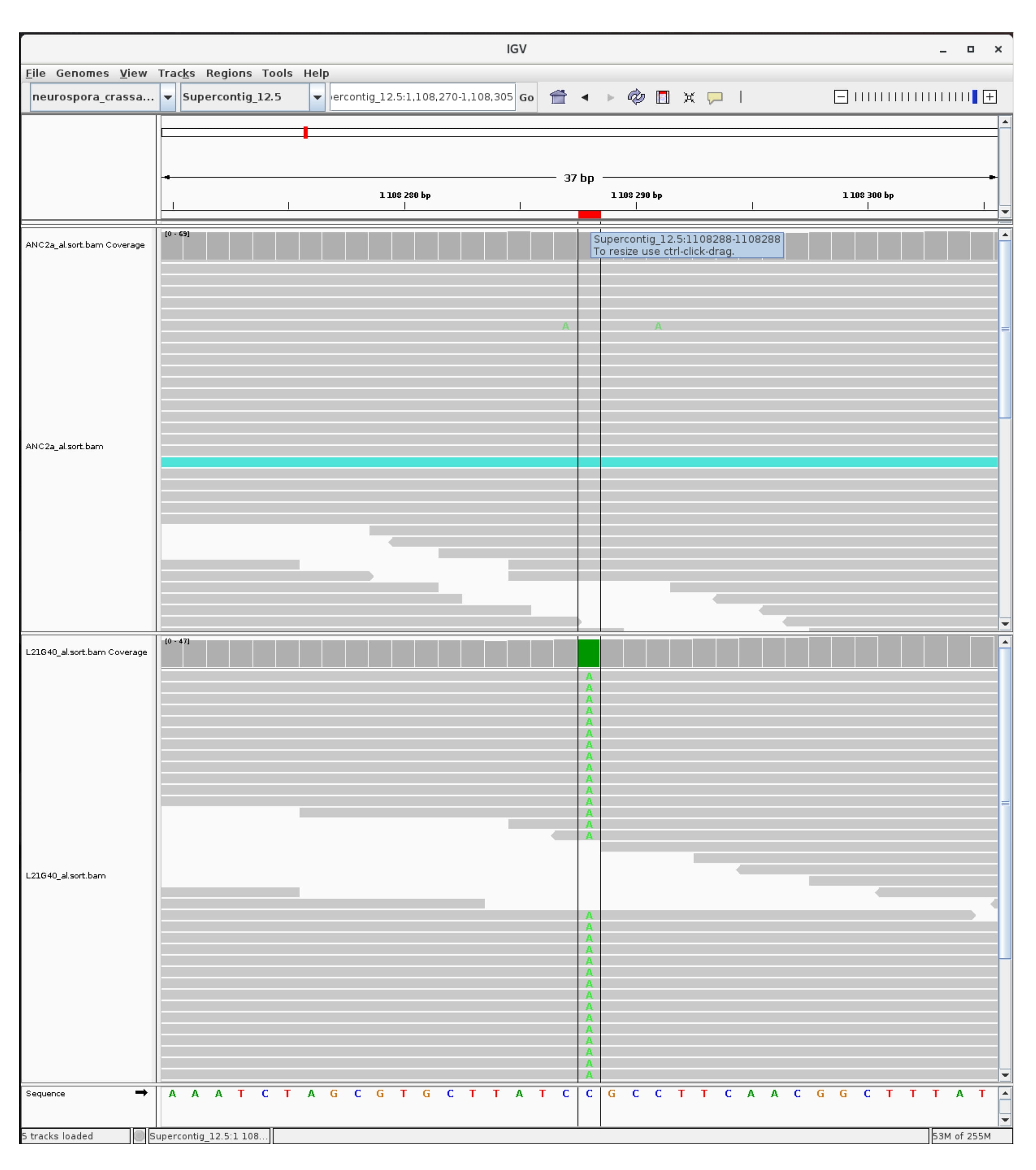

Supplement: Supplemental Material [file supp_gr.276992.122_Supplementary_file_S2.zip › IGV_screenshots/mutation_centromer_9.jpg]

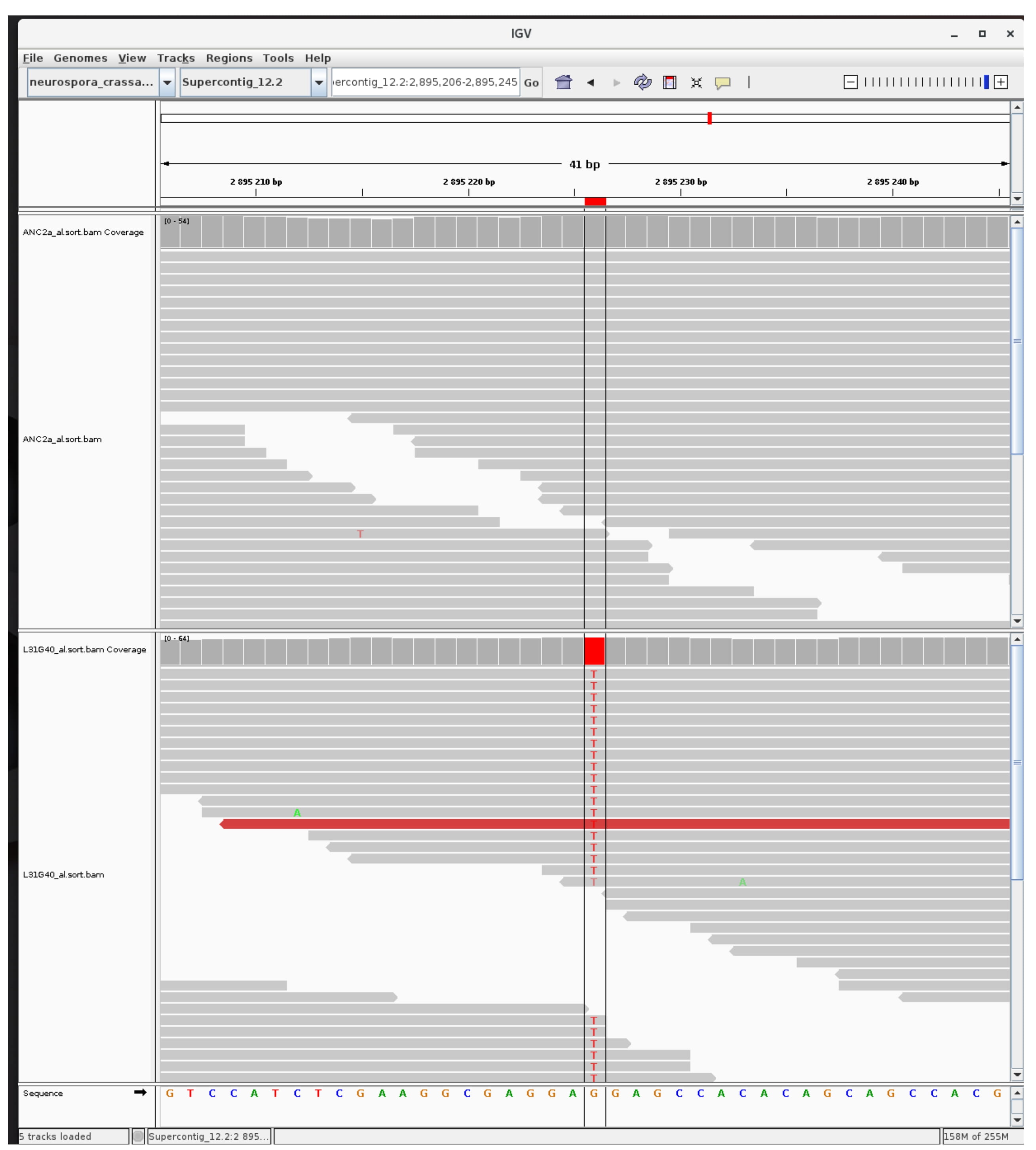

Supplement: Supplemental Material [file supp_gr.276992.122_Supplementary_file_S2.zip › IGV_screenshots/mutation_euchromatic_1.jpg]

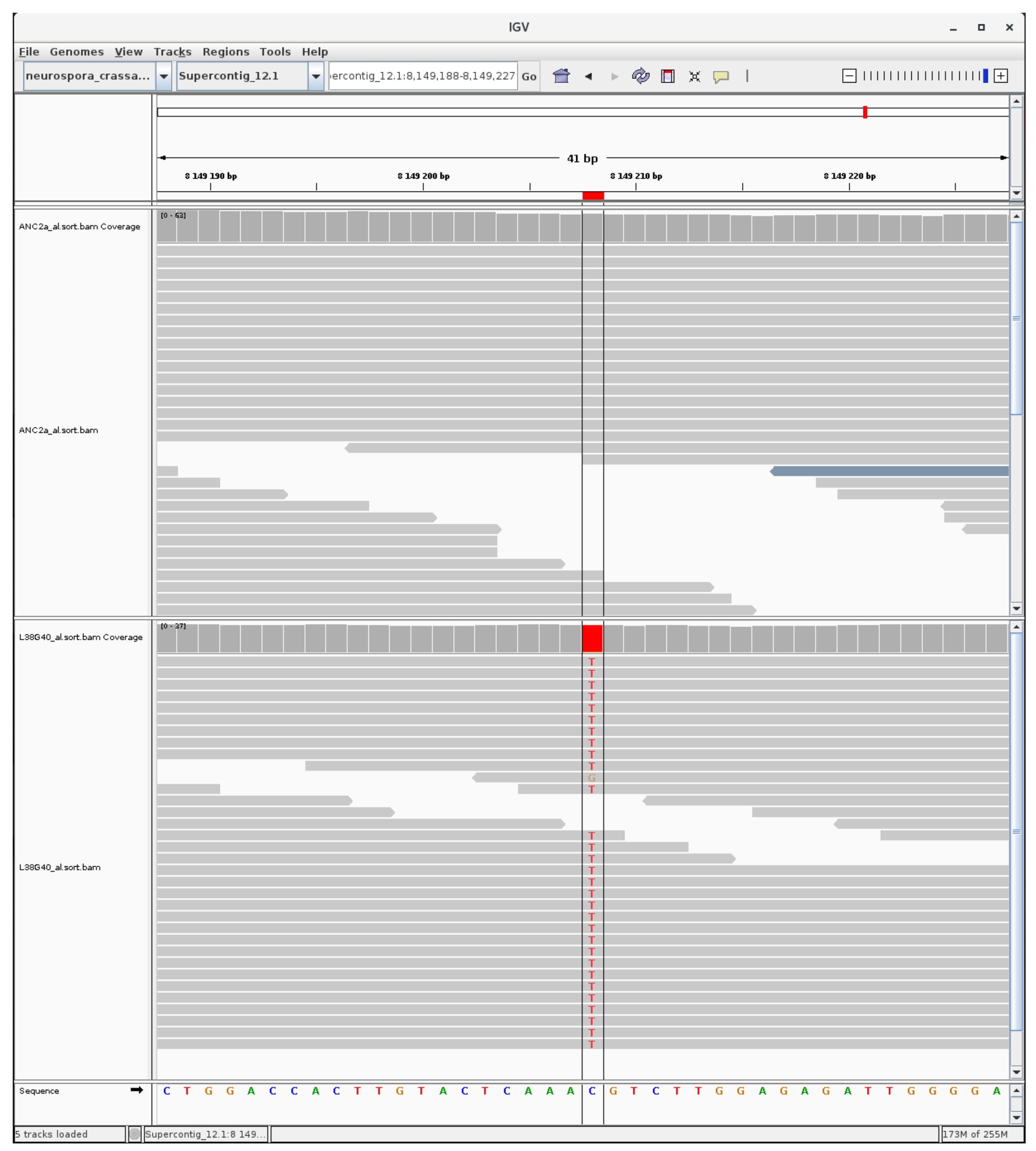

Supplement: Supplemental Material [file supp_gr.276992.122_Supplementary_file_S2.zip › IGV_screenshots/mutation_euchromatic_10.jpg]

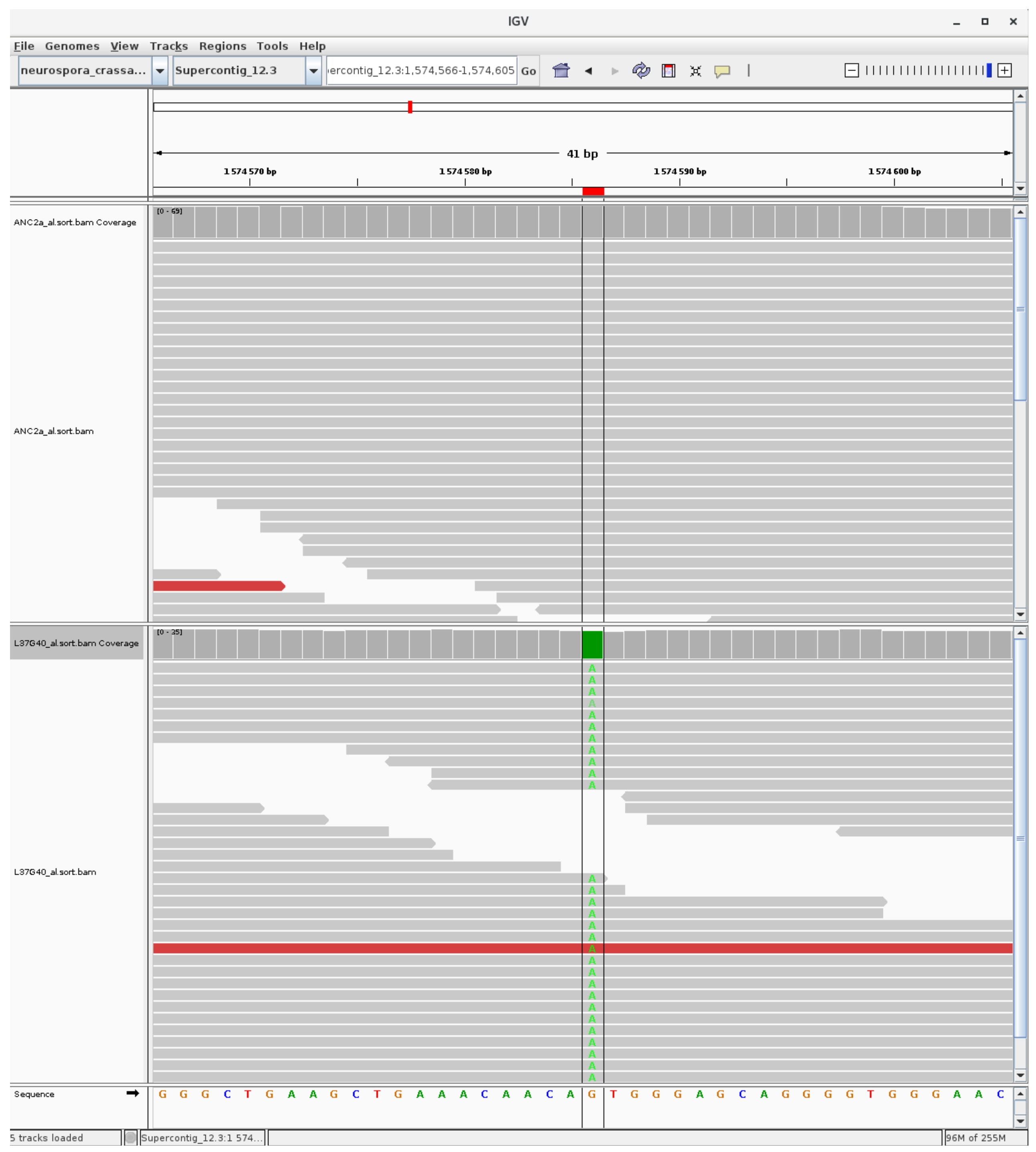

Supplement: Supplemental Material [file supp_gr.276992.122_Supplementary_file_S2.zip › IGV_screenshots/mutation_euchromatic_11.jpg]

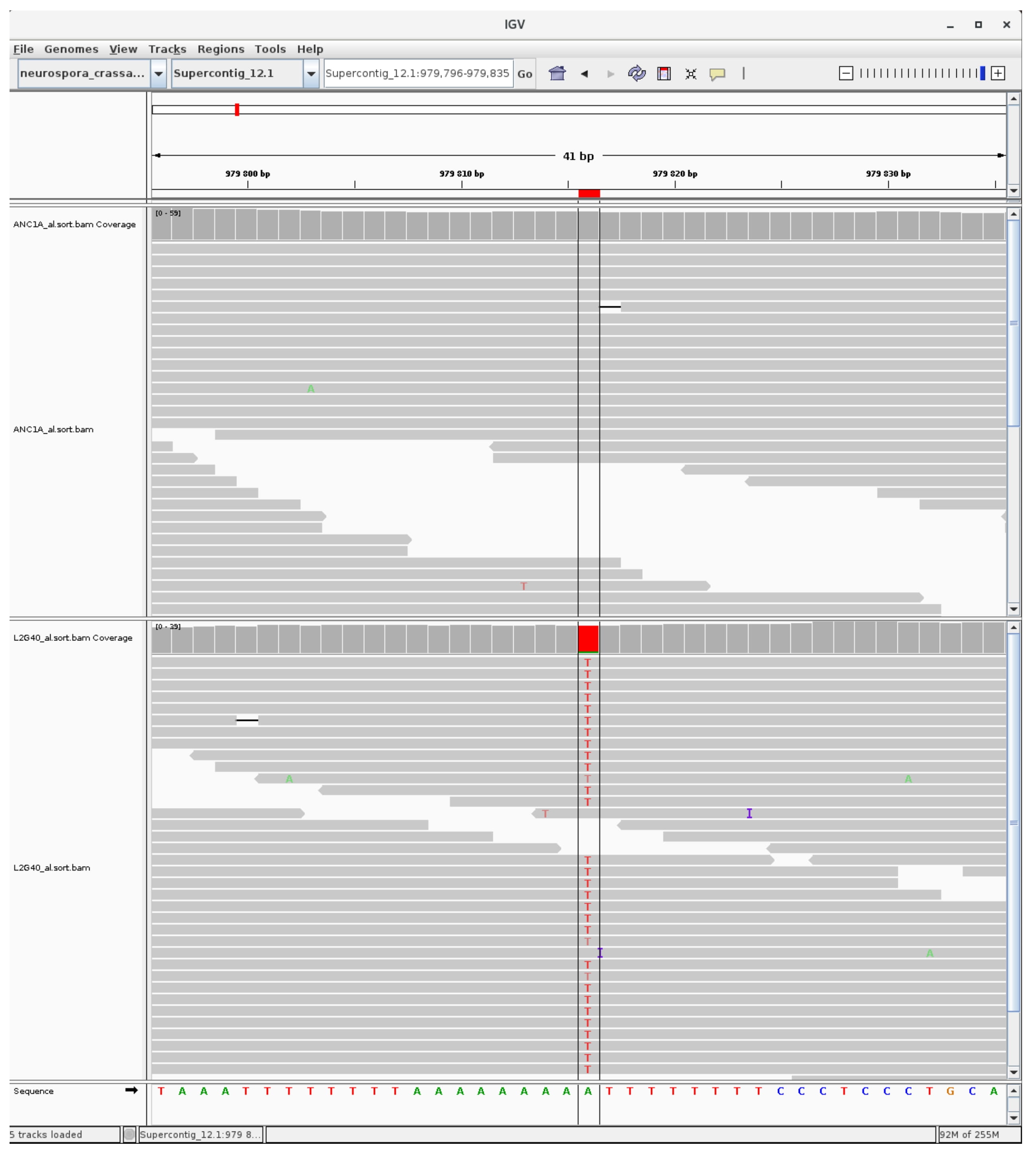

Supplement: Supplemental Material [file supp_gr.276992.122_Supplementary_file_S2.zip › IGV_screenshots/mutation_euchromatic_12.jpg]

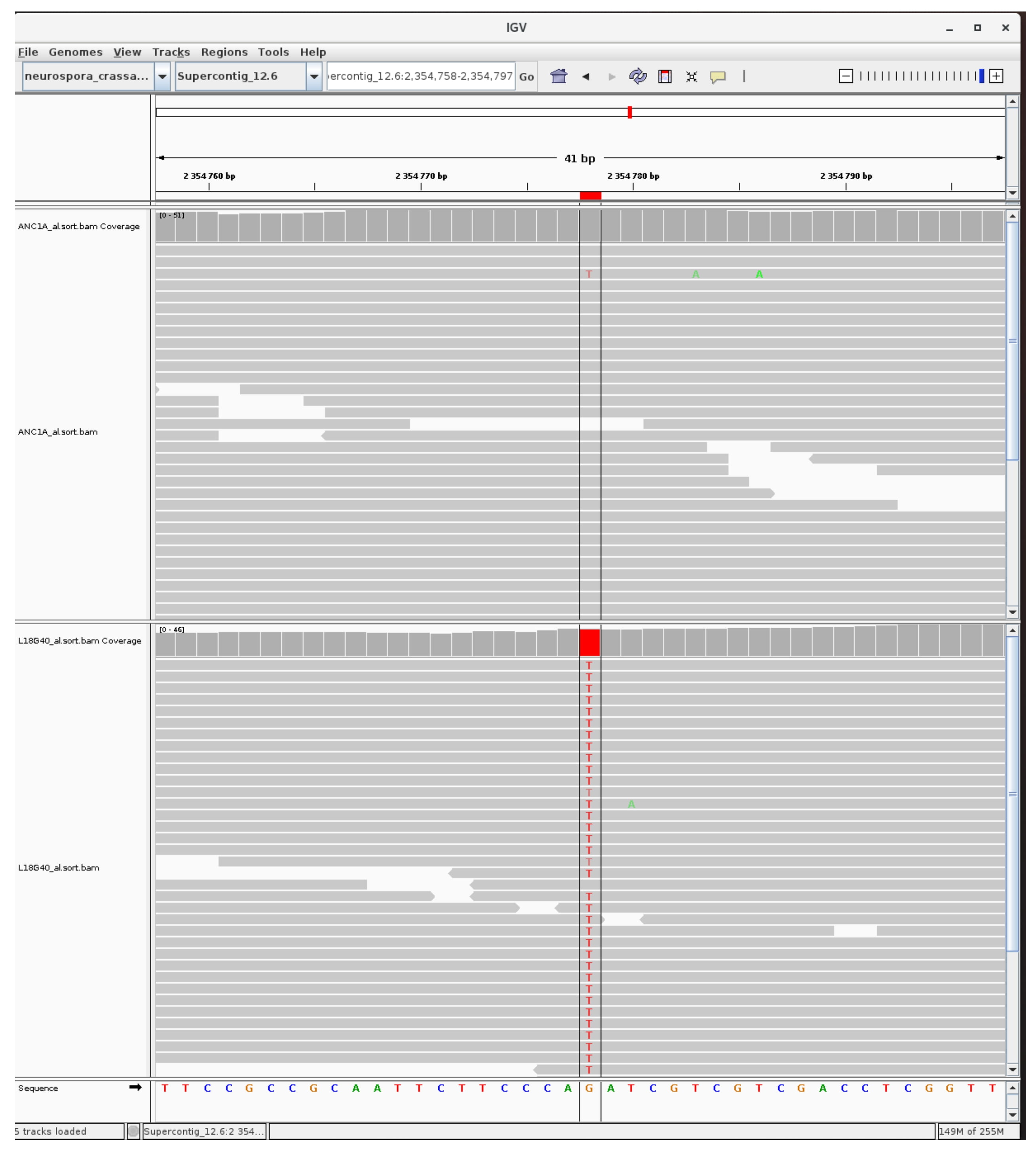

Supplement: Supplemental Material [file supp_gr.276992.122_Supplementary_file_S2.zip › IGV_screenshots/mutation_euchromatic_13.jpg]

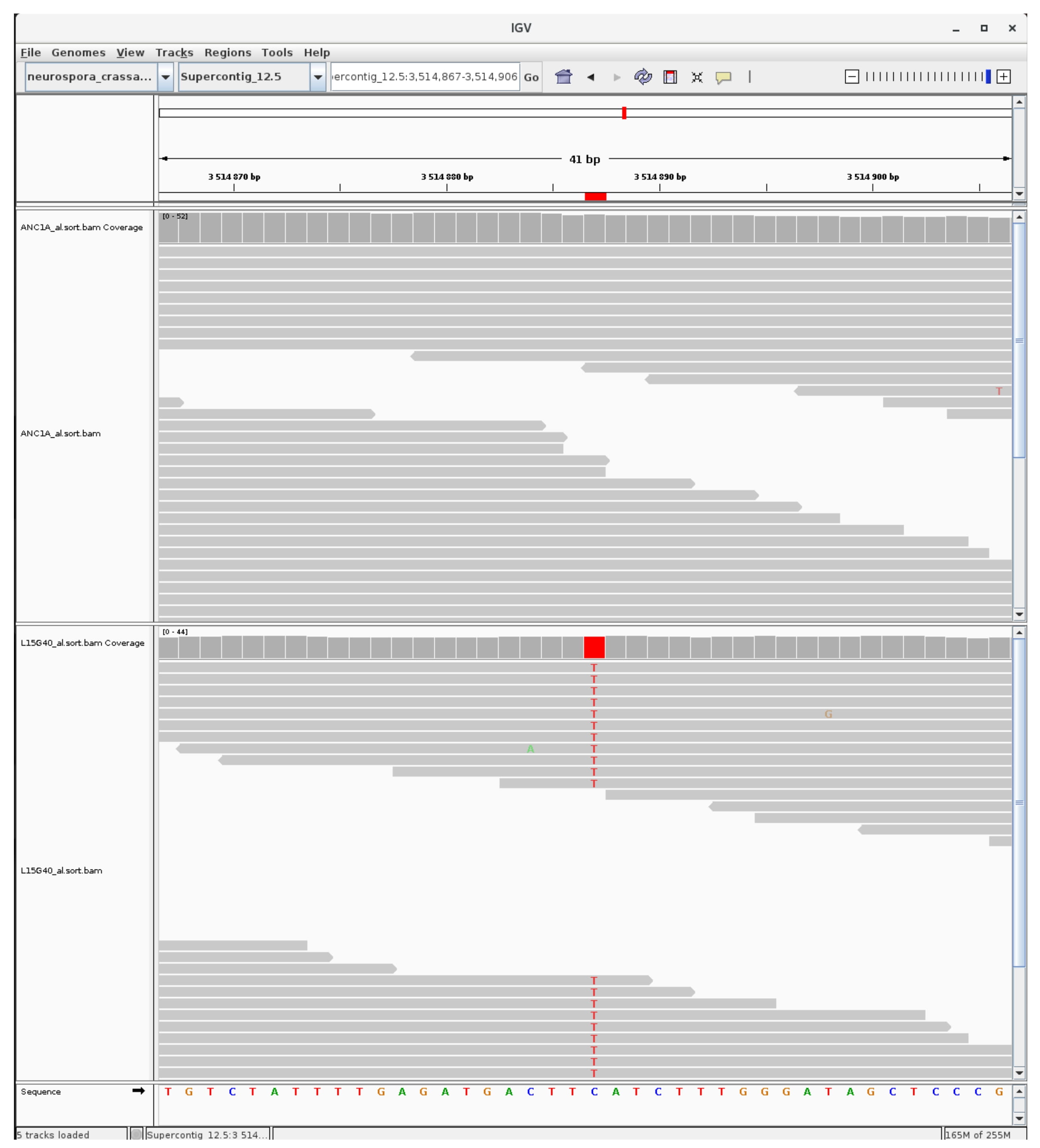

Supplement: Supplemental Material [file supp_gr.276992.122_Supplementary_file_S2.zip › IGV_screenshots/mutation_euchromatic_14.jpg]

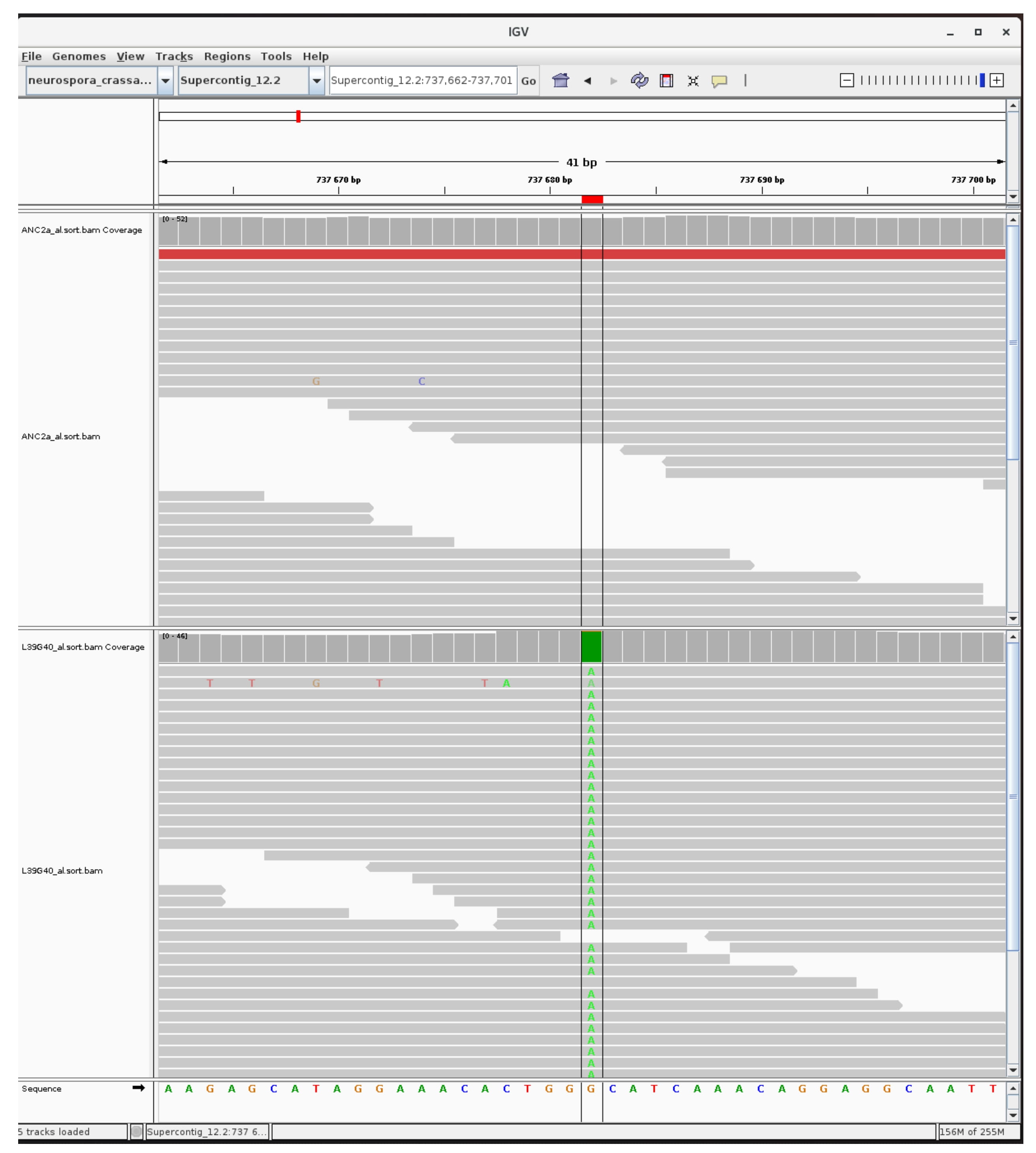

Supplement: Supplemental Material [file supp_gr.276992.122_Supplementary_file_S2.zip › IGV_screenshots/mutation_euchromatic_15.jpg]

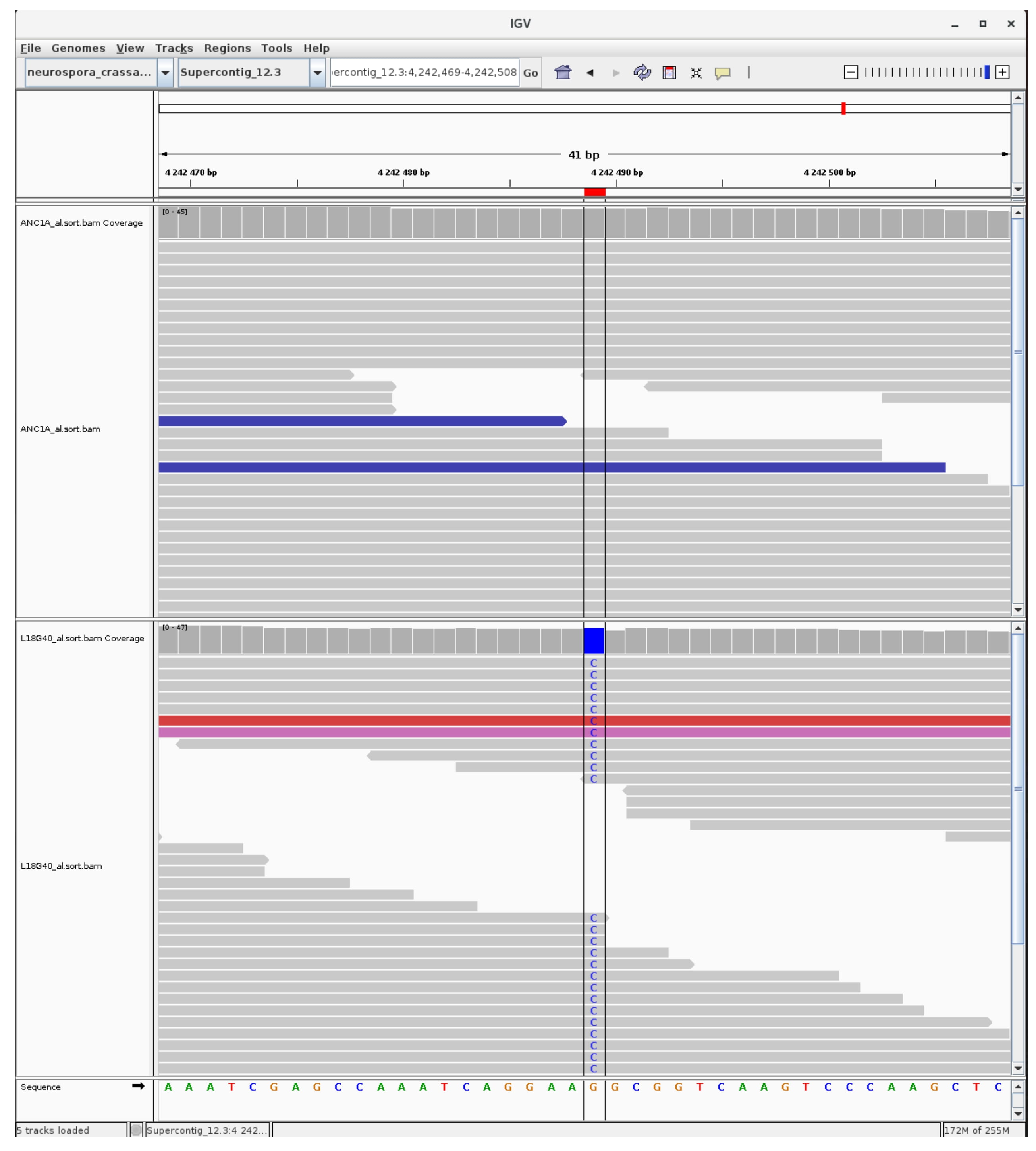

Supplement: Supplemental Material [file supp_gr.276992.122_Supplementary_file_S2.zip › IGV_screenshots/mutation_euchromatic_16.jpg]

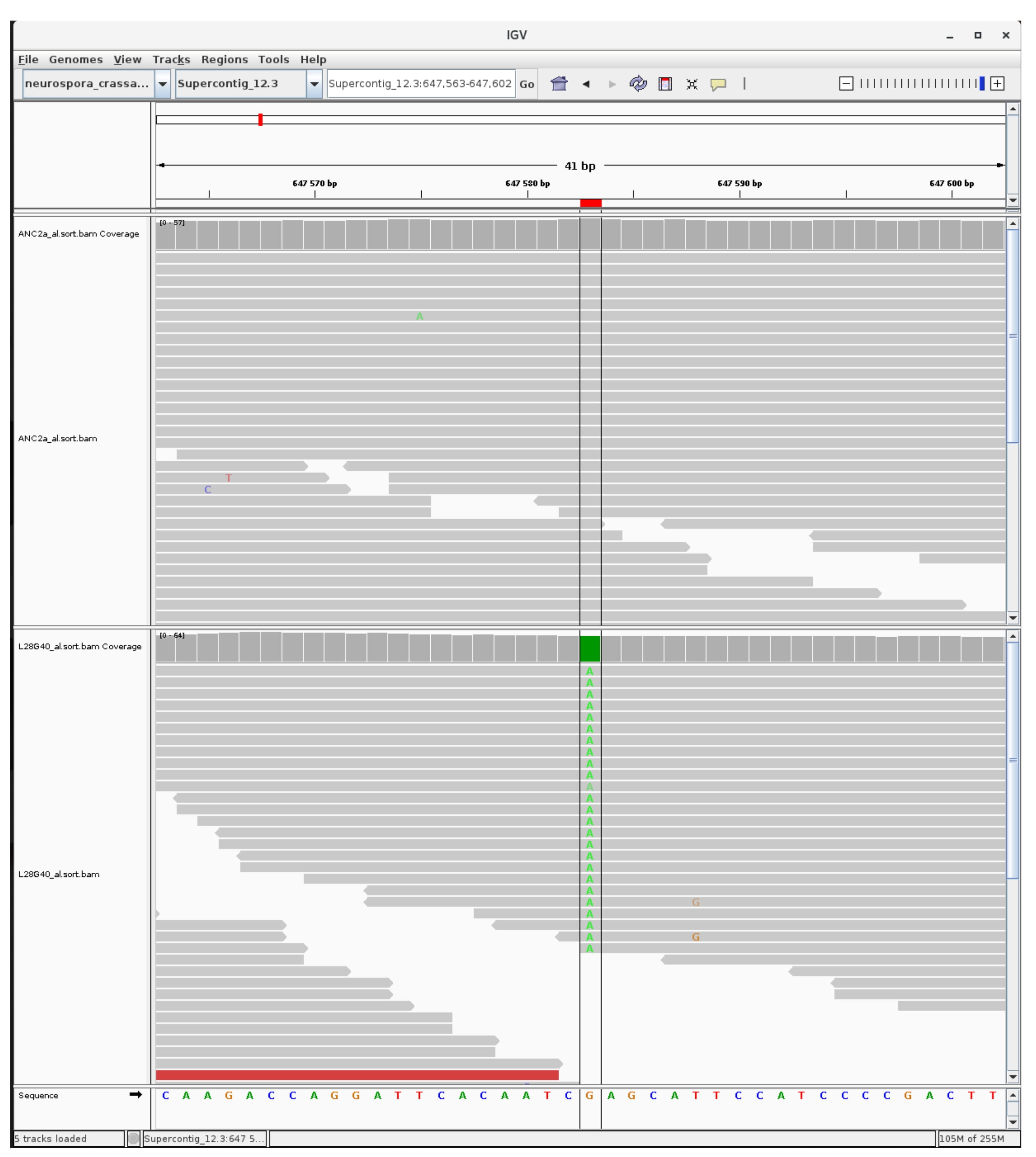

Supplement: Supplemental Material [file supp_gr.276992.122_Supplementary_file_S2.zip › IGV_screenshots/mutation_euchromatic_17.jpg]

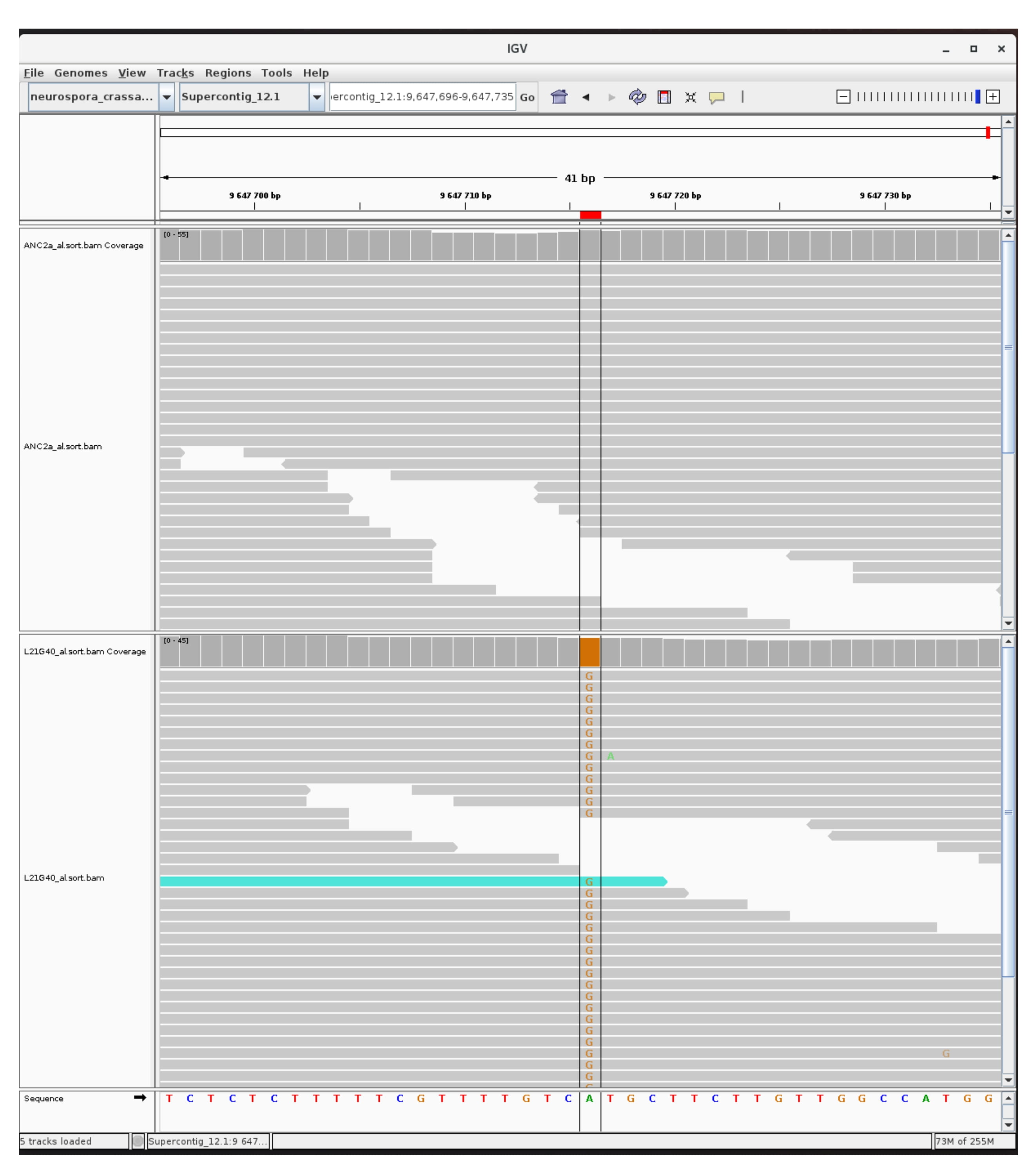

Supplement: Supplemental Material [file supp_gr.276992.122_Supplementary_file_S2.zip › IGV_screenshots/mutation_euchromatic_18.jpg]

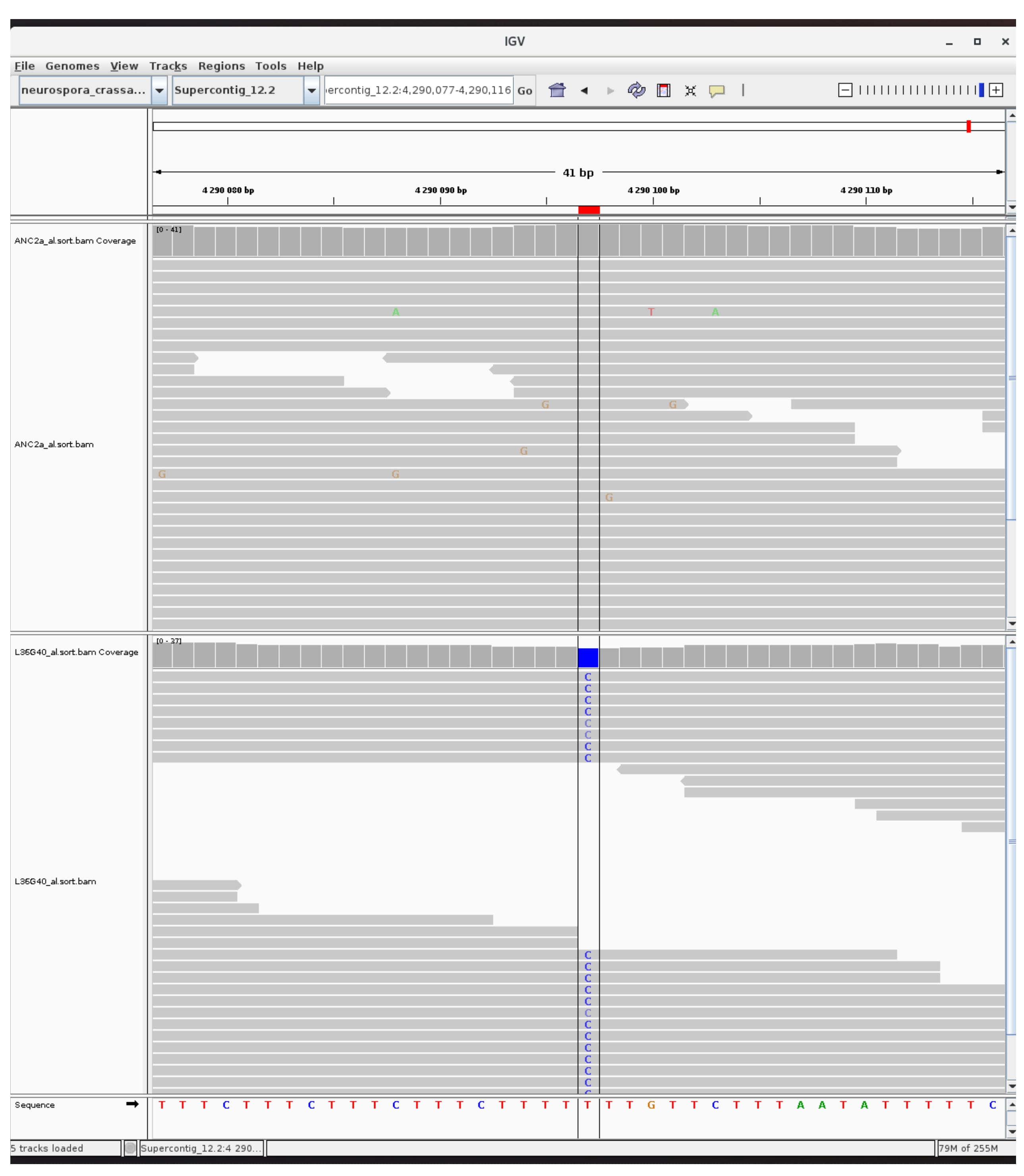

Supplement: Supplemental Material [file supp_gr.276992.122_Supplementary_file_S2.zip › IGV_screenshots/mutation_euchromatic_19.jpg]

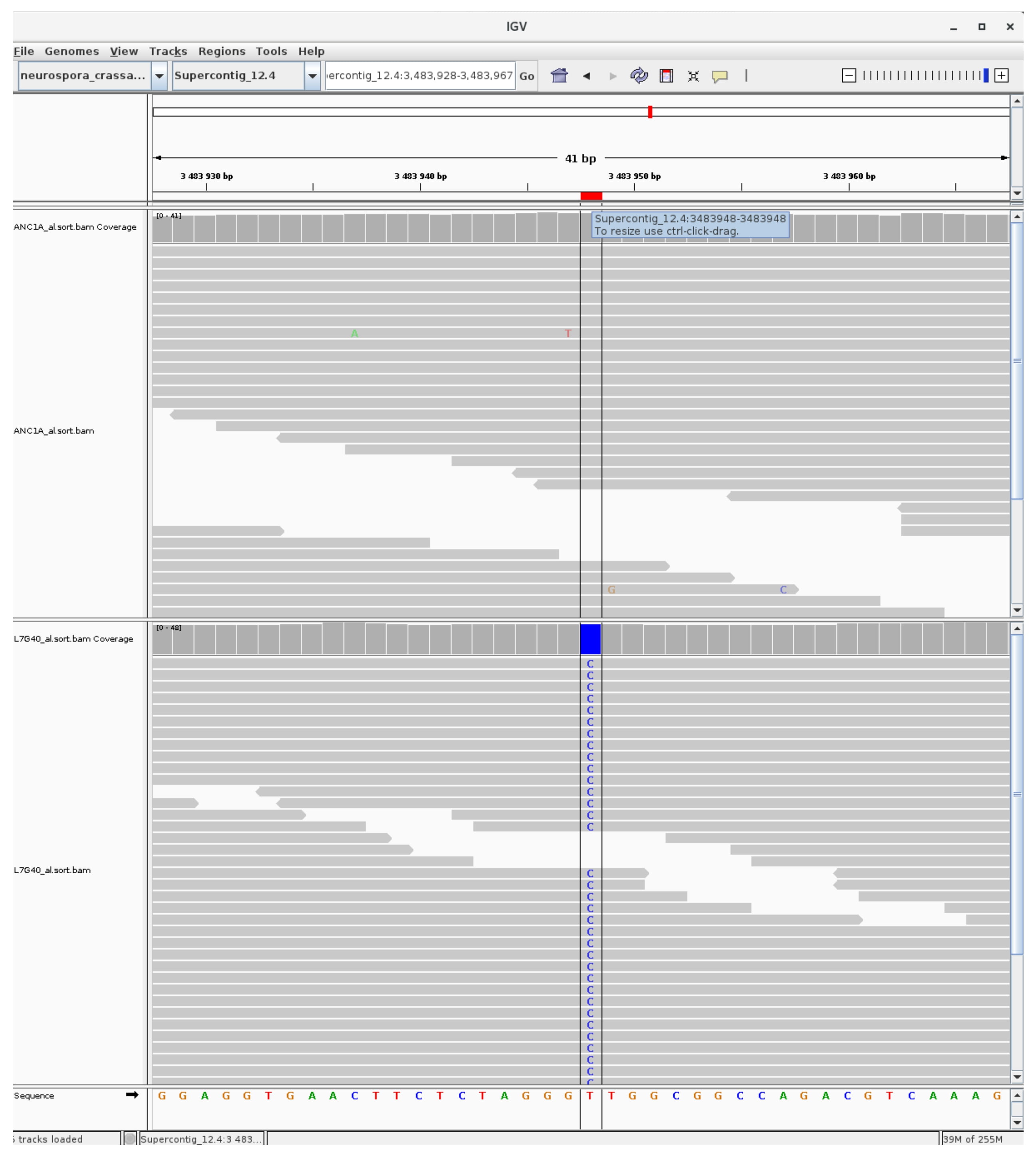

Supplement: Supplemental Material [file supp_gr.276992.122_Supplementary_file_S2.zip › IGV_screenshots/mutation_euchromatic_2.jpg]

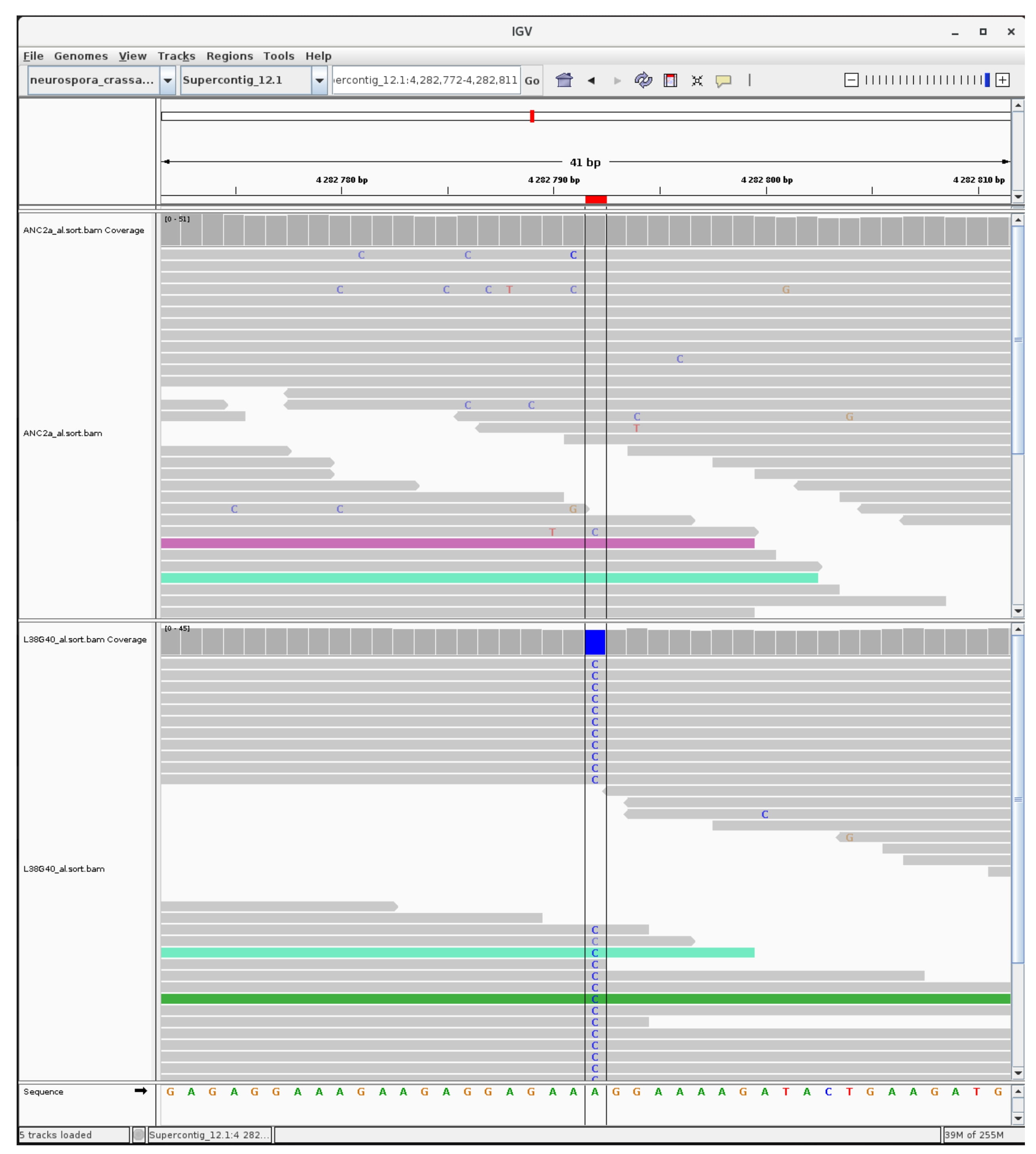

Supplement: Supplemental Material [file supp_gr.276992.122_Supplementary_file_S2.zip › IGV_screenshots/mutation_euchromatic_20.jpg]

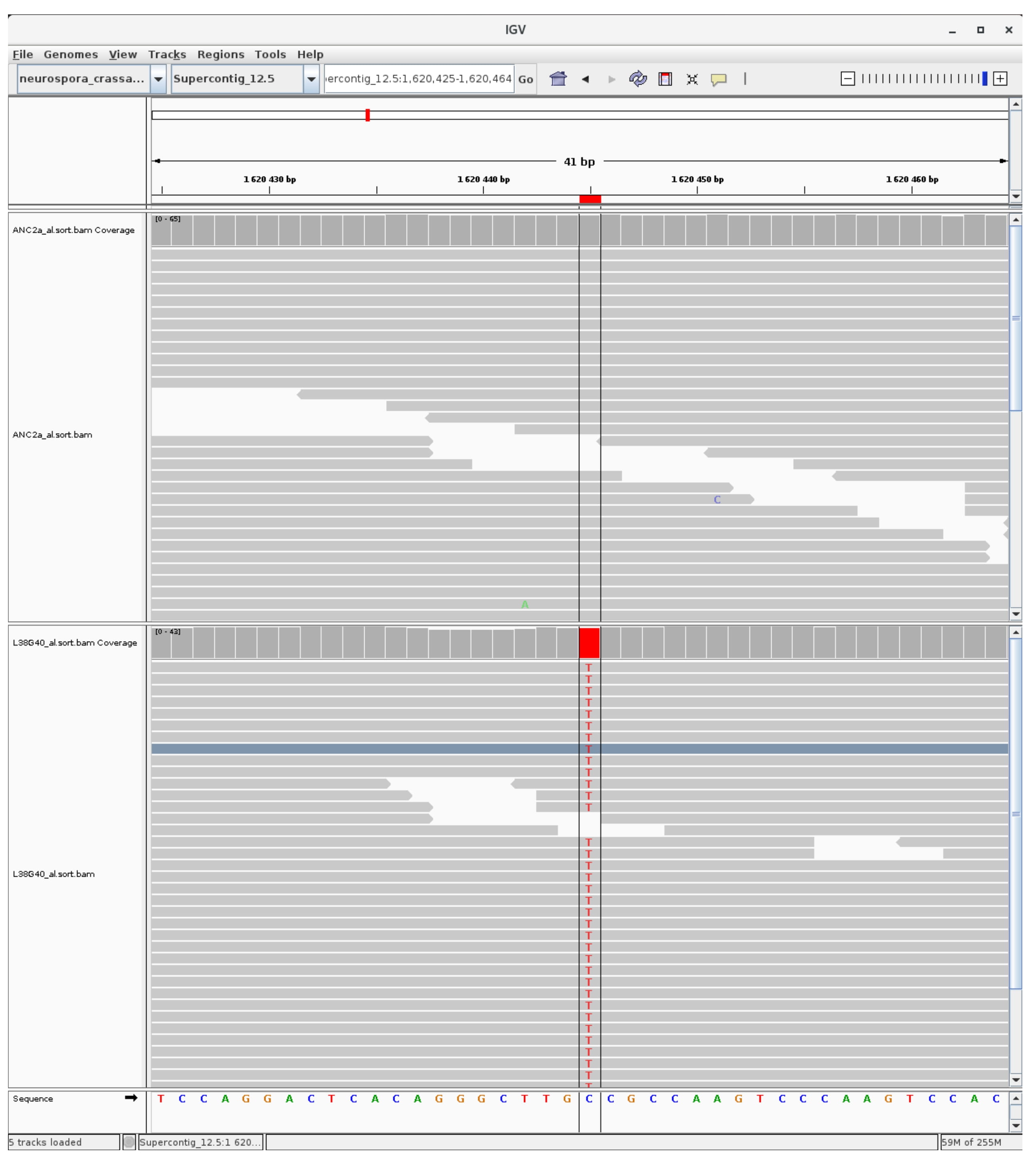

Supplement: Supplemental Material [file supp_gr.276992.122_Supplementary_file_S2.zip › IGV_screenshots/mutation_euchromatic_21.jpg]

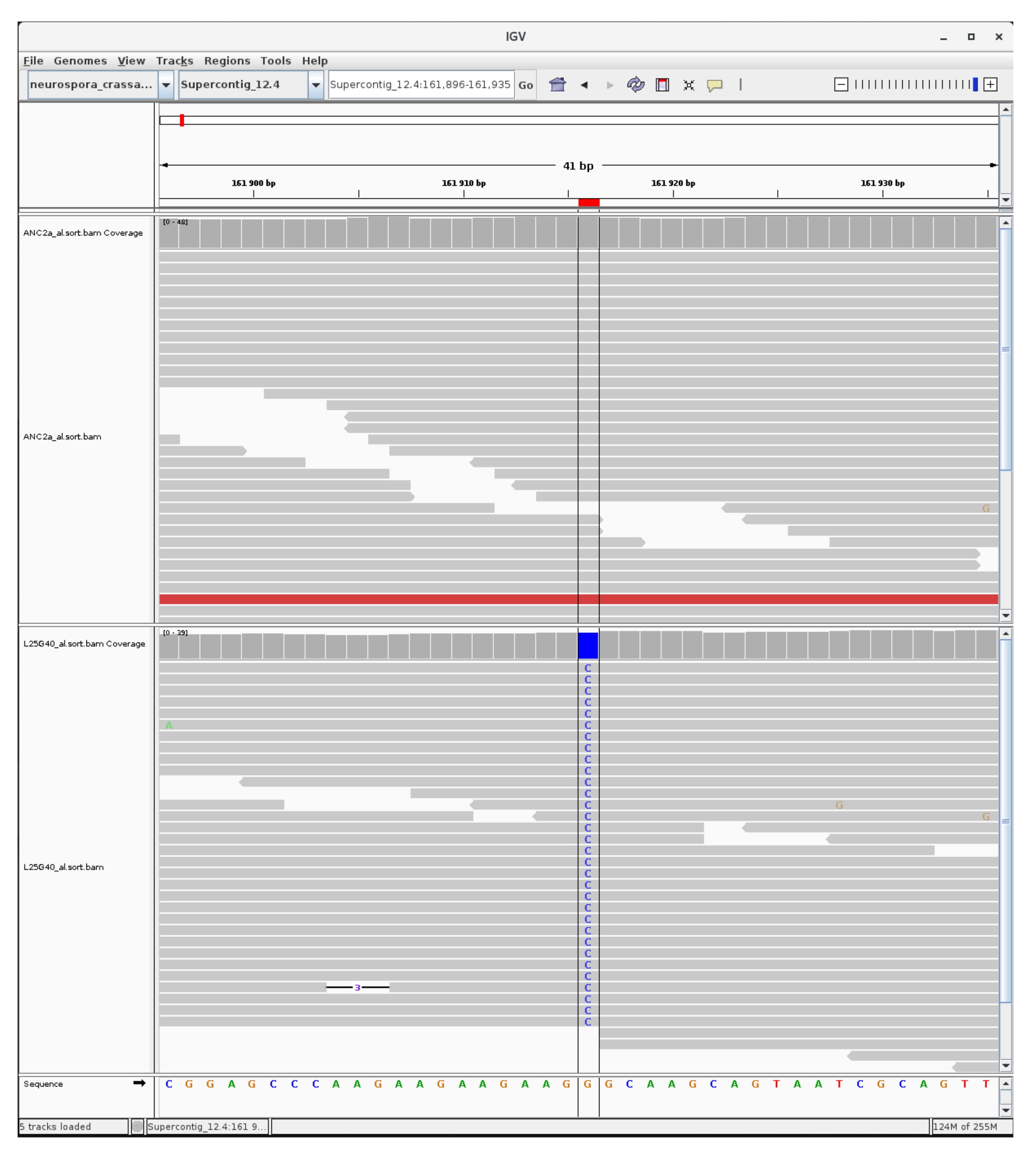

Supplement: Supplemental Material [file supp_gr.276992.122_Supplementary_file_S2.zip › IGV_screenshots/mutation_euchromatic_22.jpg]

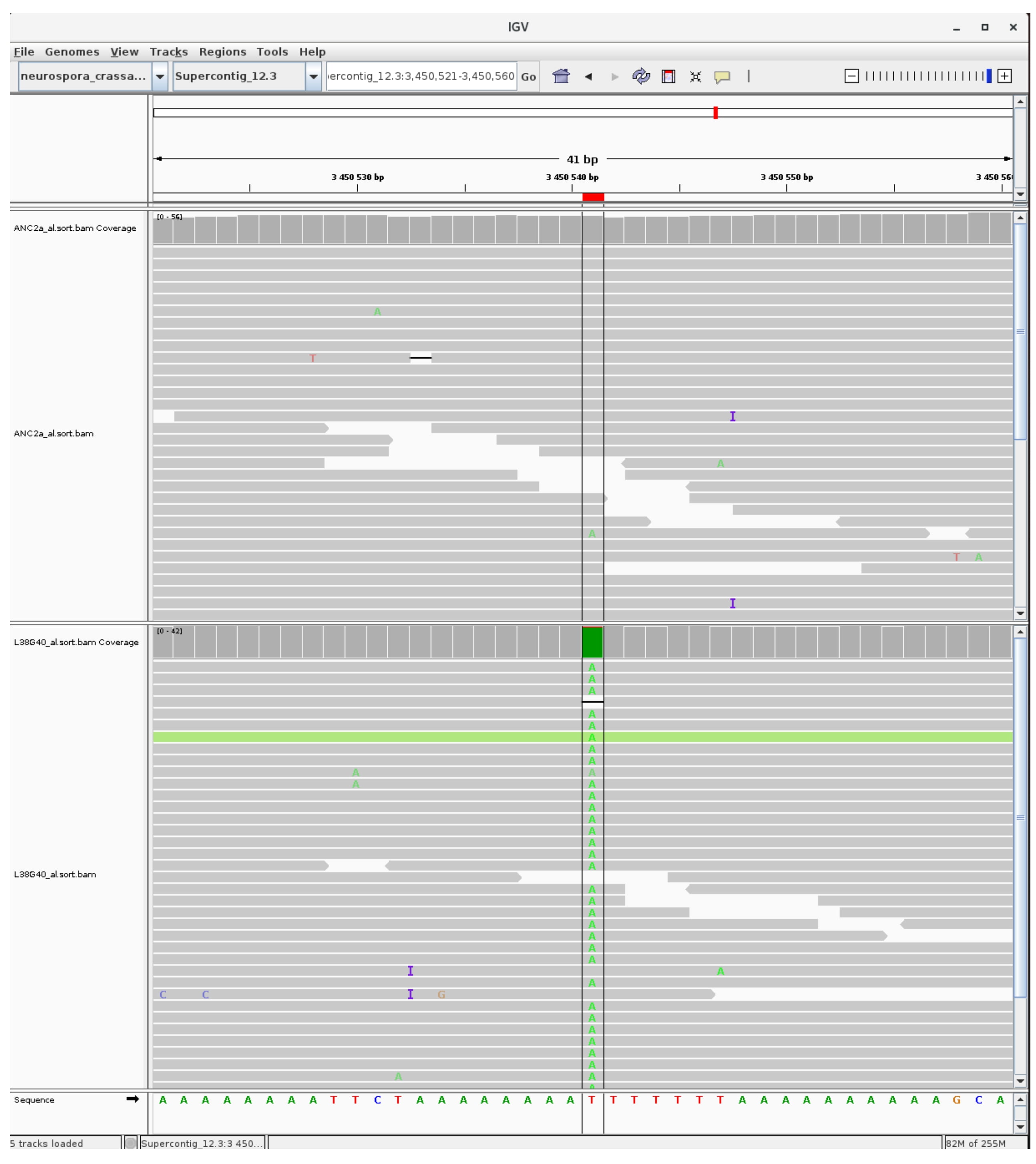

Supplement: Supplemental Material [file supp_gr.276992.122_Supplementary_file_S2.zip › IGV_screenshots/mutation_euchromatic_23.jpg]

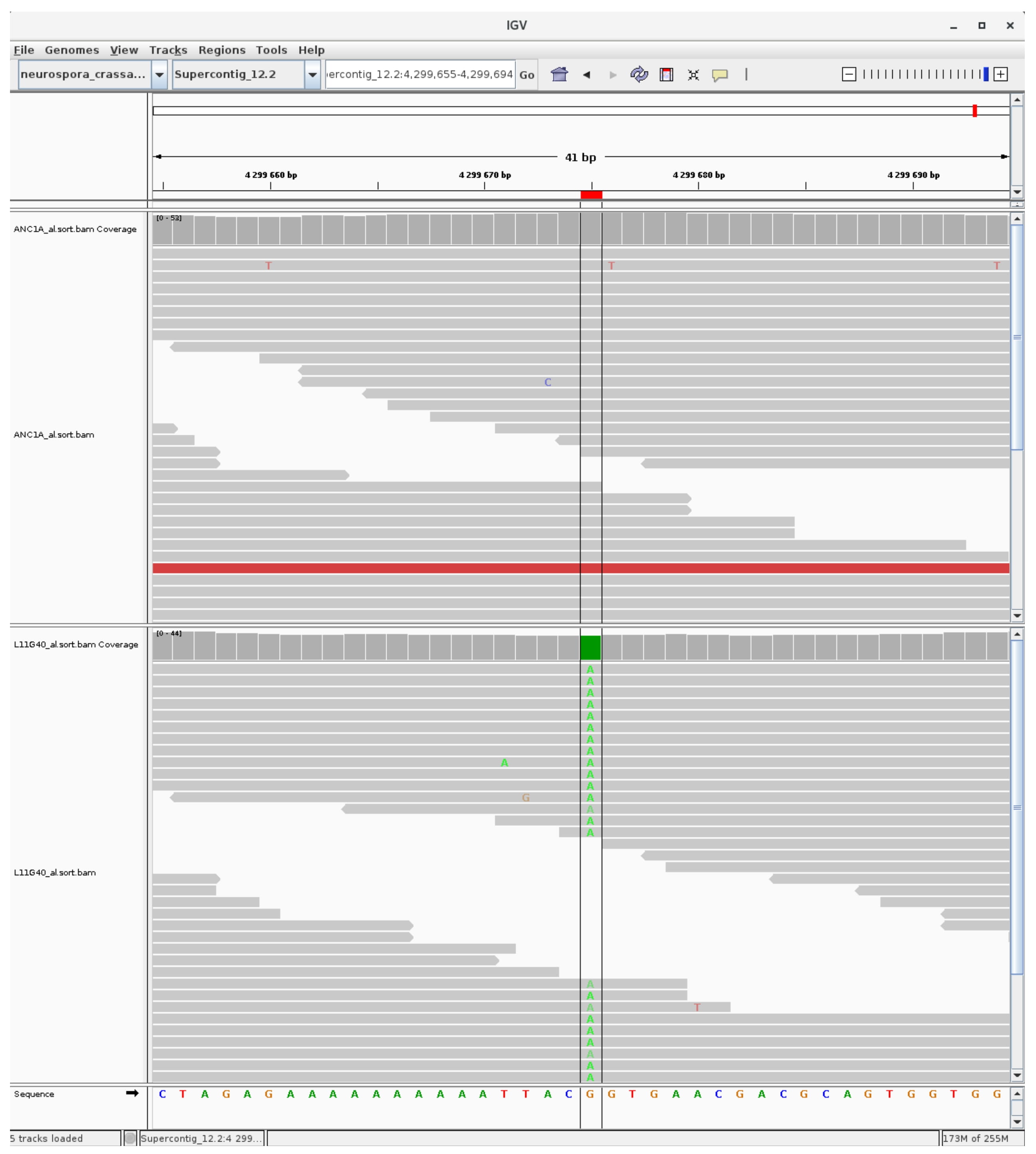

Supplement: Supplemental Material [file supp_gr.276992.122_Supplementary_file_S2.zip › IGV_screenshots/mutation_euchromatic_24.jpg]

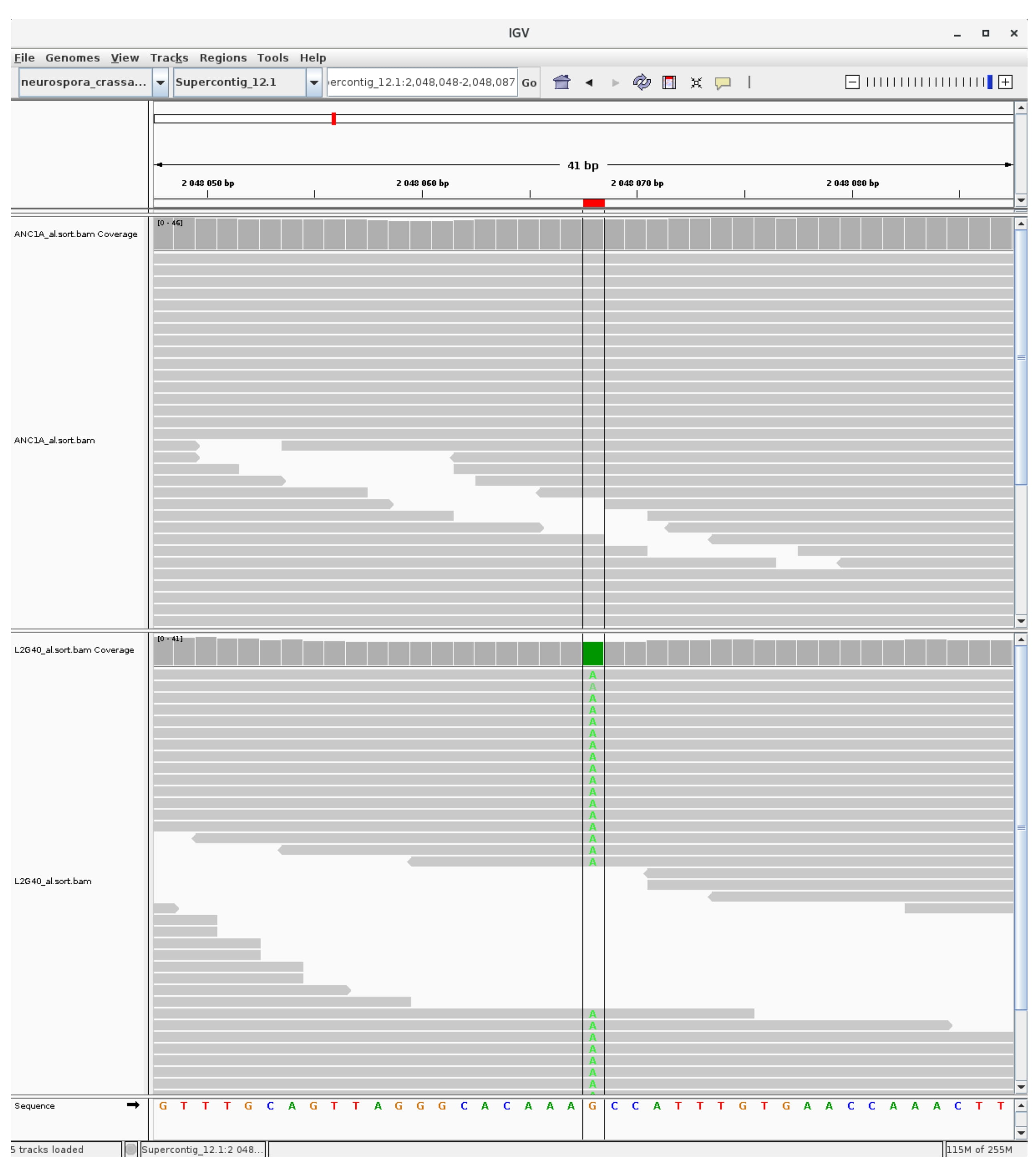

Supplement: Supplemental Material [file supp_gr.276992.122_Supplementary_file_S2.zip › IGV_screenshots/mutation_euchromatic_25.jpg]

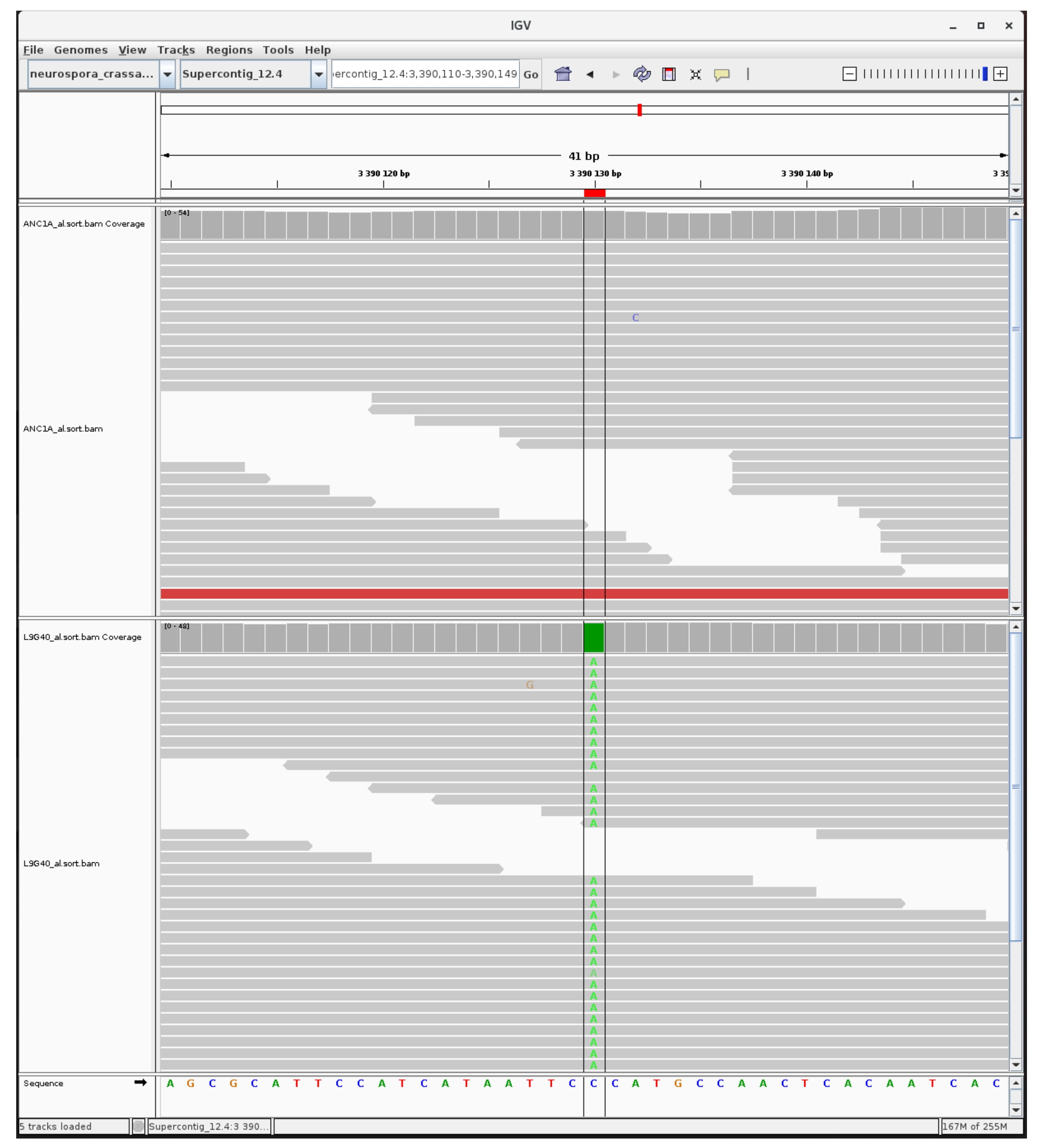

Supplement: Supplemental Material [file supp_gr.276992.122_Supplementary_file_S2.zip › IGV_screenshots/mutation_euchromatic_26.jpg]

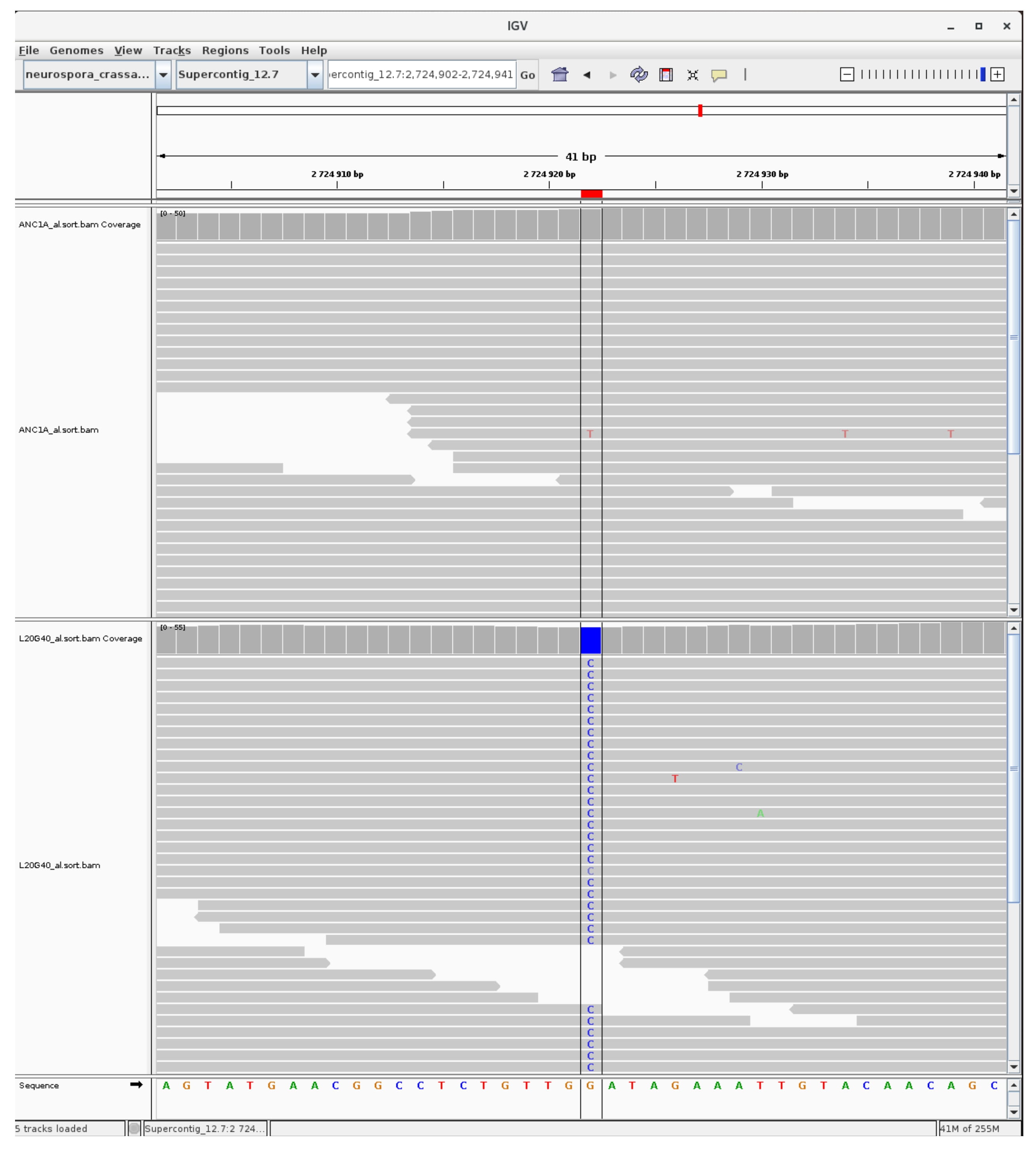

Supplement: Supplemental Material [file supp_gr.276992.122_Supplementary_file_S2.zip › IGV_screenshots/mutation_euchromatic_27.jpg]

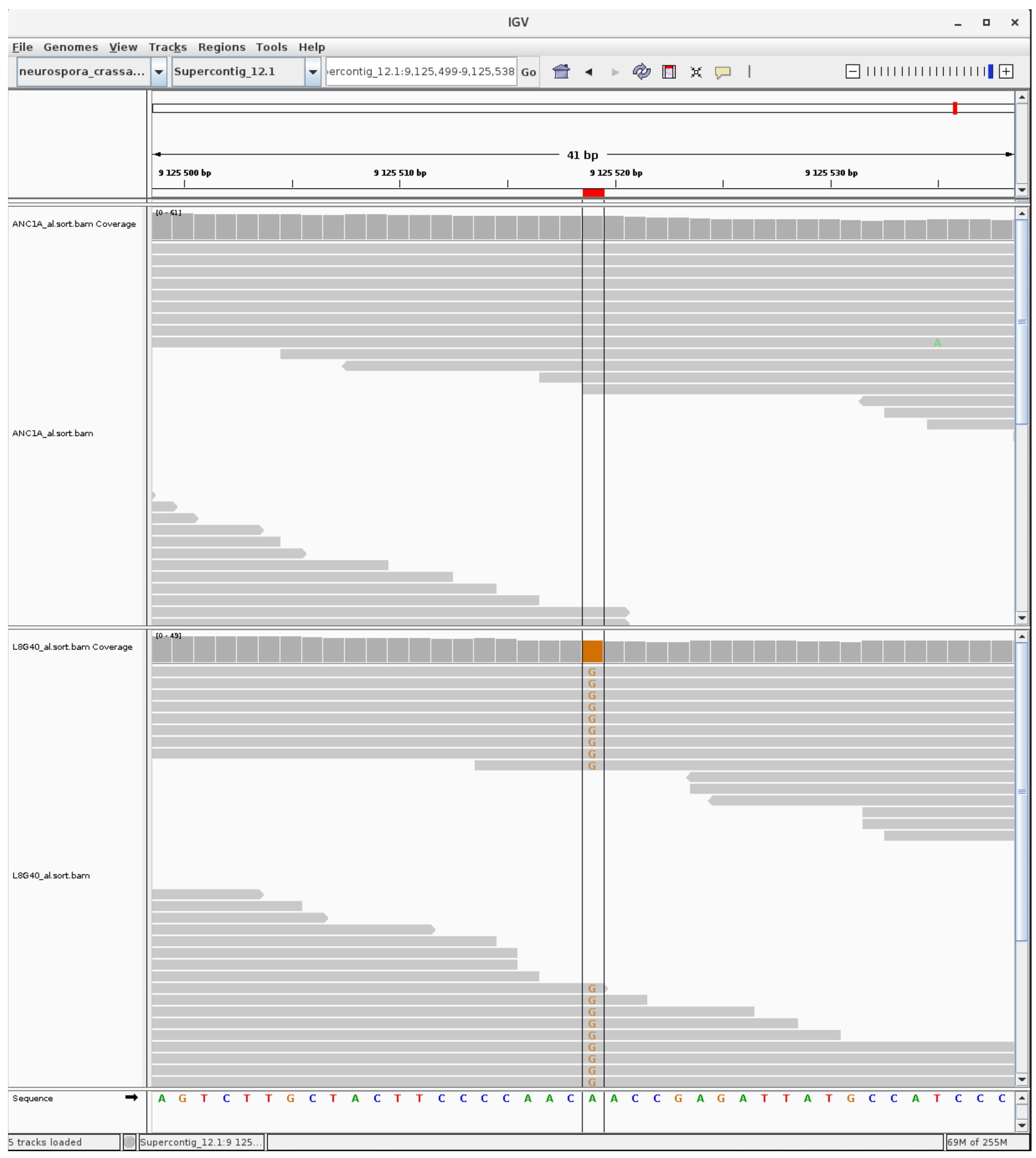

Supplement: Supplemental Material [file supp_gr.276992.122_Supplementary_file_S2.zip › IGV_screenshots/mutation_euchromatic_28.jpg]

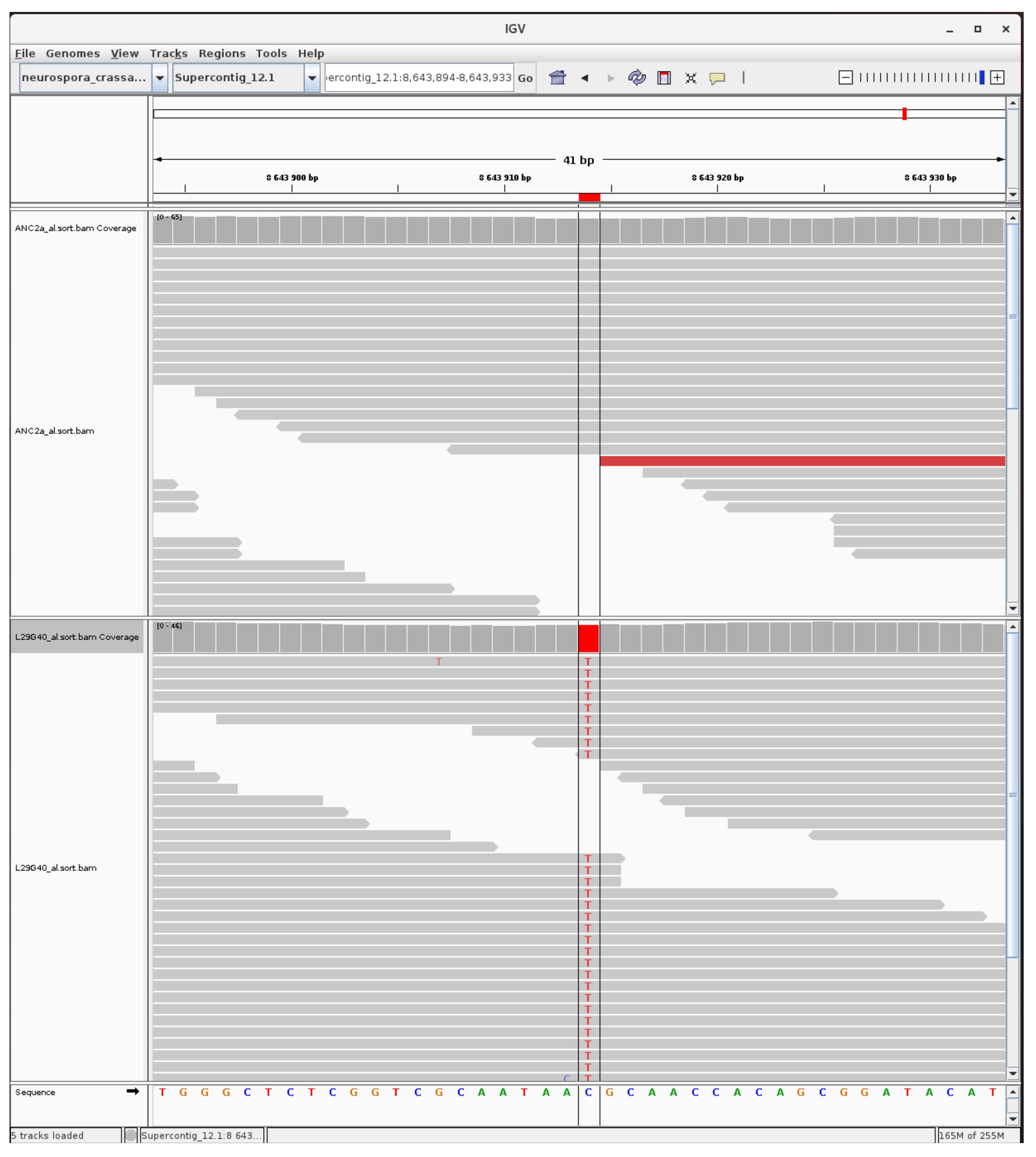

Supplement: Supplemental Material [file supp_gr.276992.122_Supplementary_file_S2.zip › IGV_screenshots/mutation_euchromatic_29.jpg]

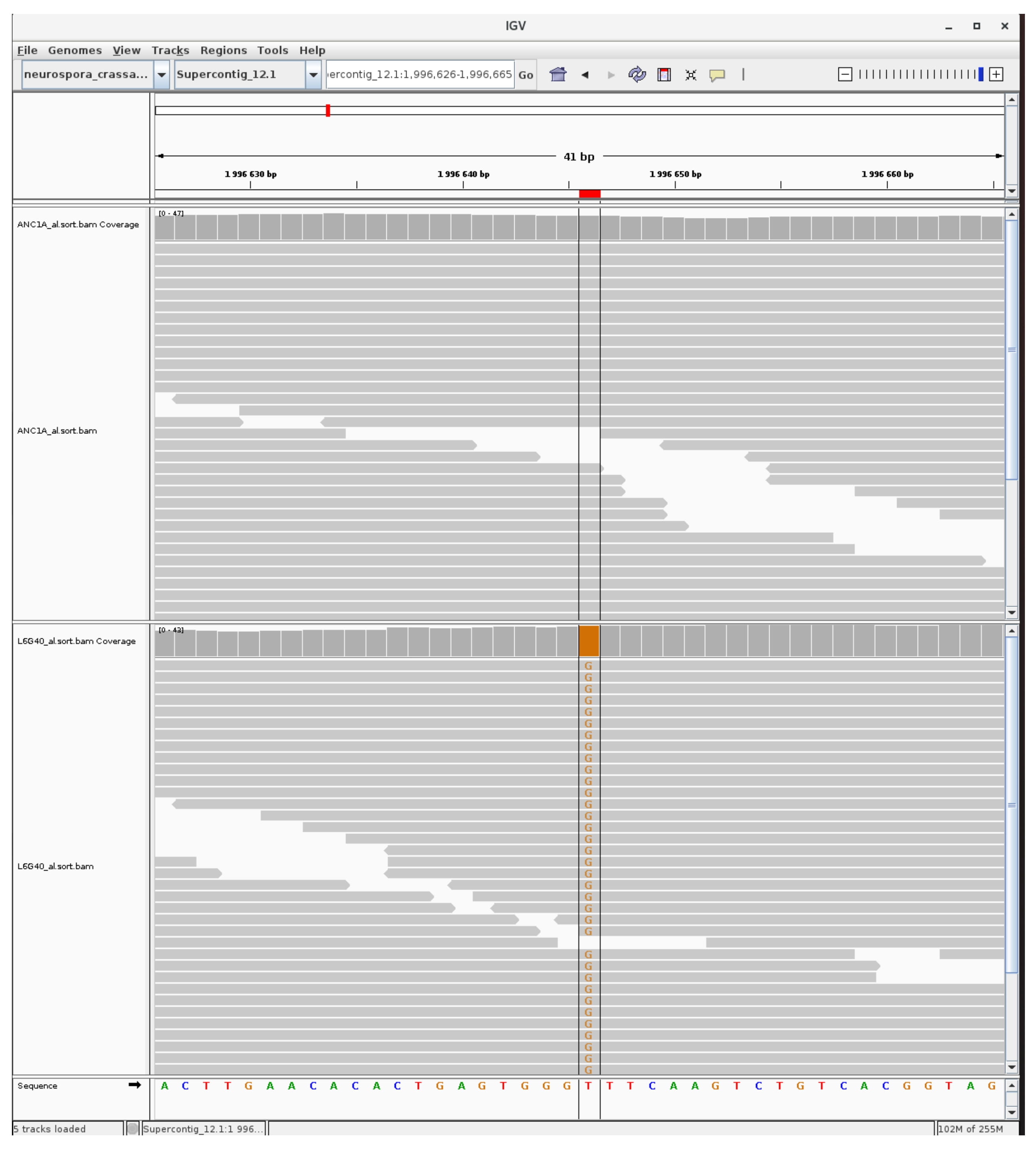

Supplement: Supplemental Material [file supp_gr.276992.122_Supplementary_file_S2.zip › IGV_screenshots/mutation_euchromatic_3.jpg]

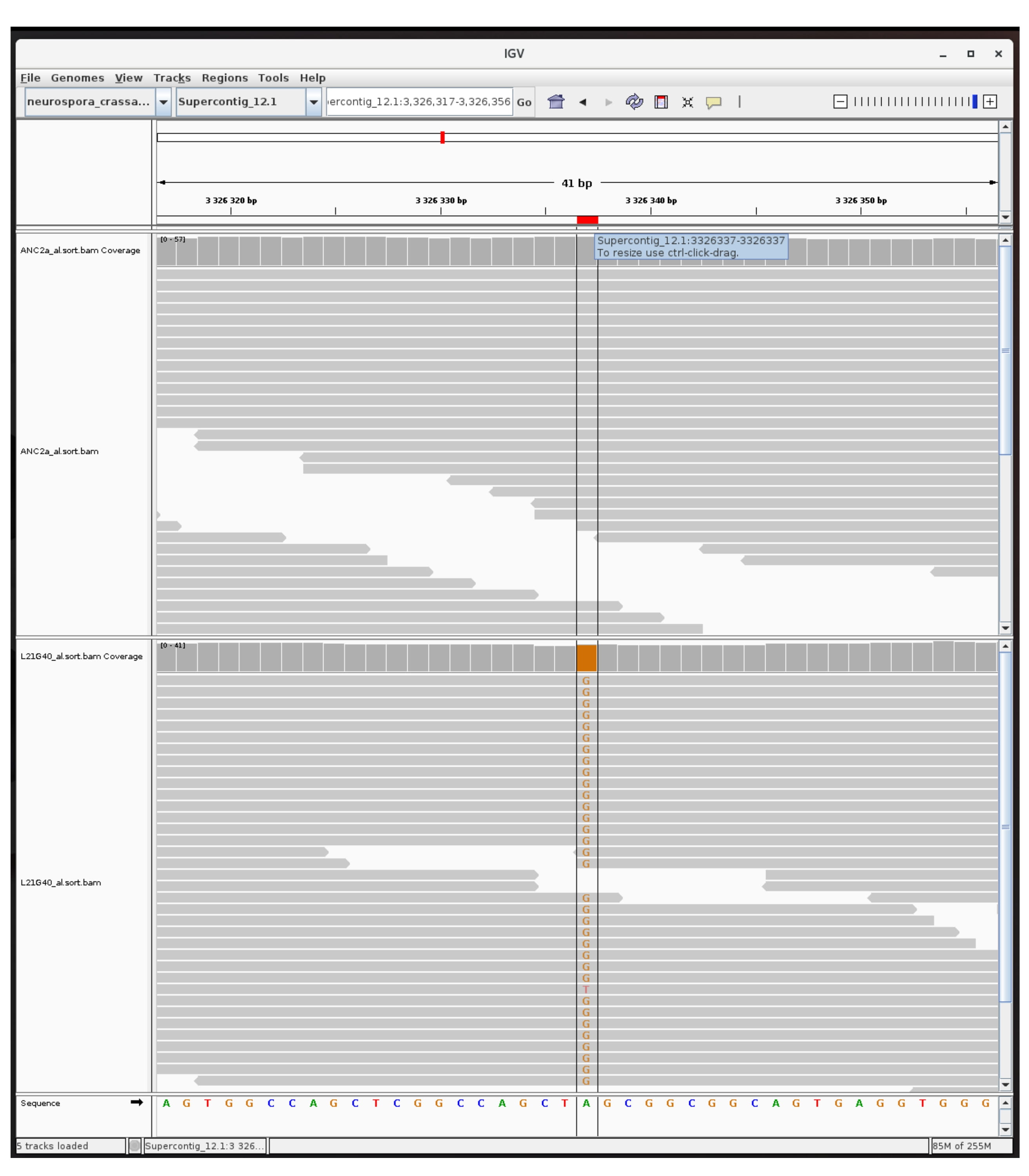

Supplement: Supplemental Material [file supp_gr.276992.122_Supplementary_file_S2.zip › IGV_screenshots/mutation_euchromatic_30.jpg]

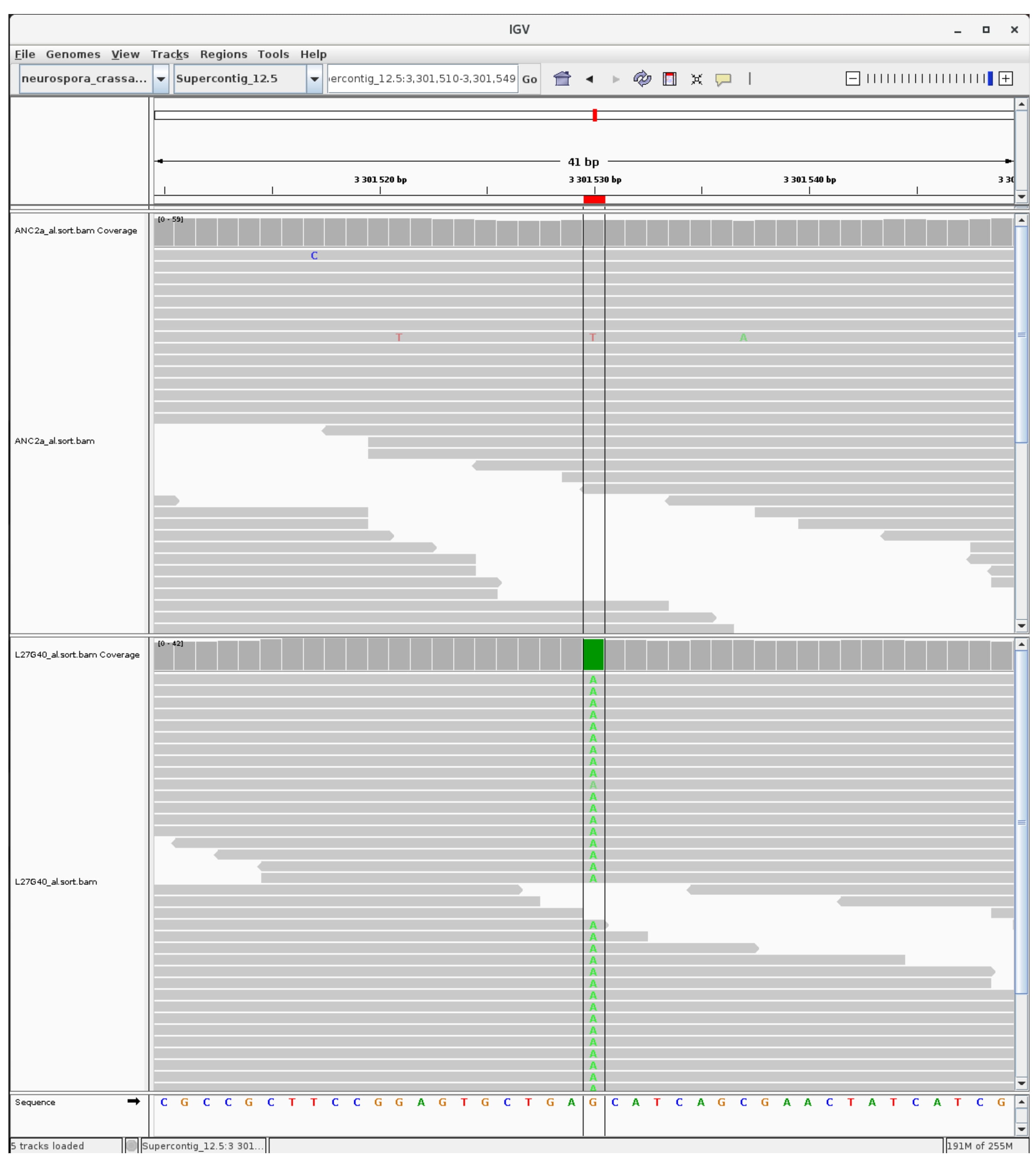

Supplement: Supplemental Material [file supp_gr.276992.122_Supplementary_file_S2.zip › IGV_screenshots/mutation_euchromatic_4.jpg]

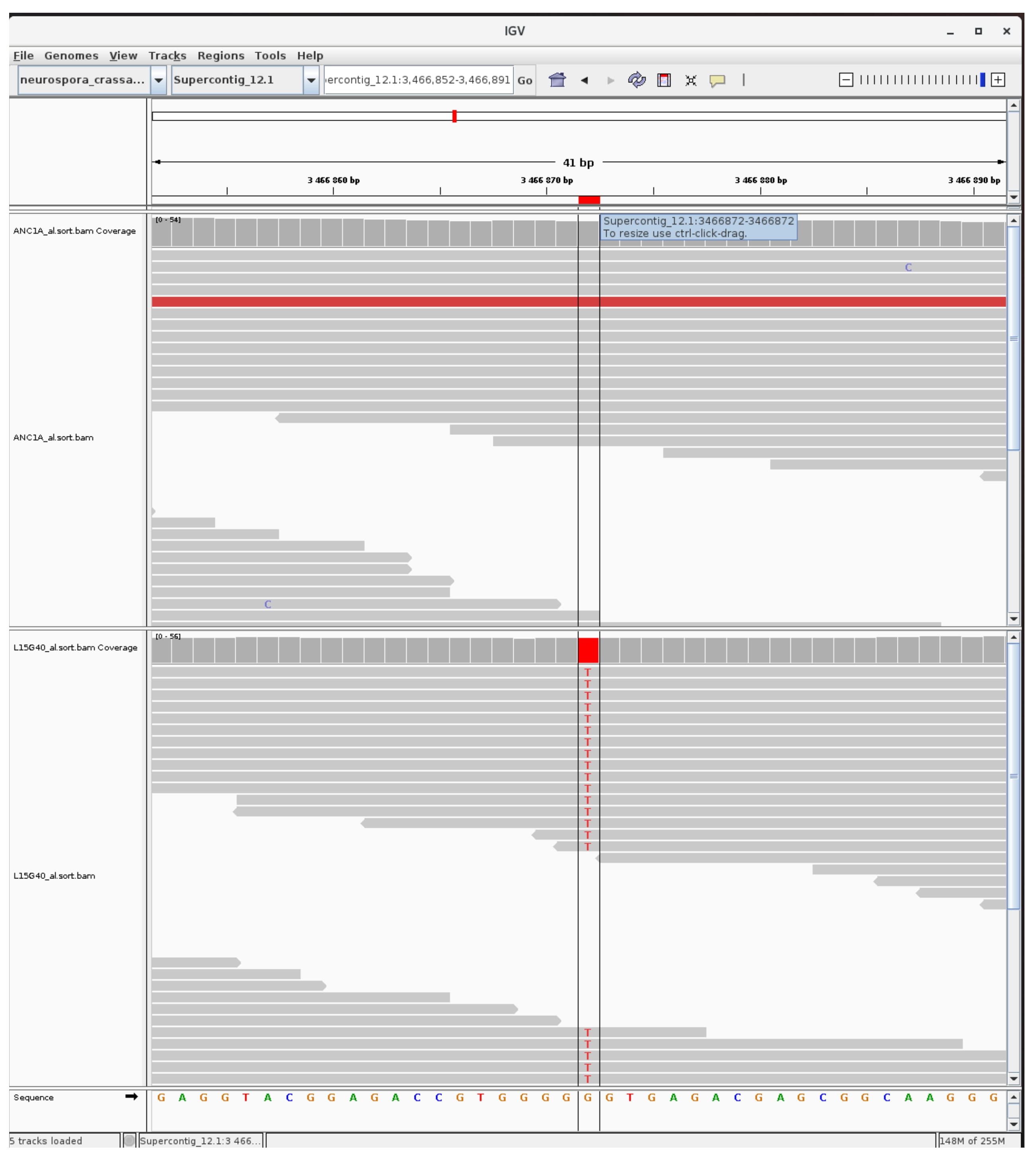

Supplement: Supplemental Material [file supp_gr.276992.122_Supplementary_file_S2.zip › IGV_screenshots/mutation_euchromatic_5.jpg]

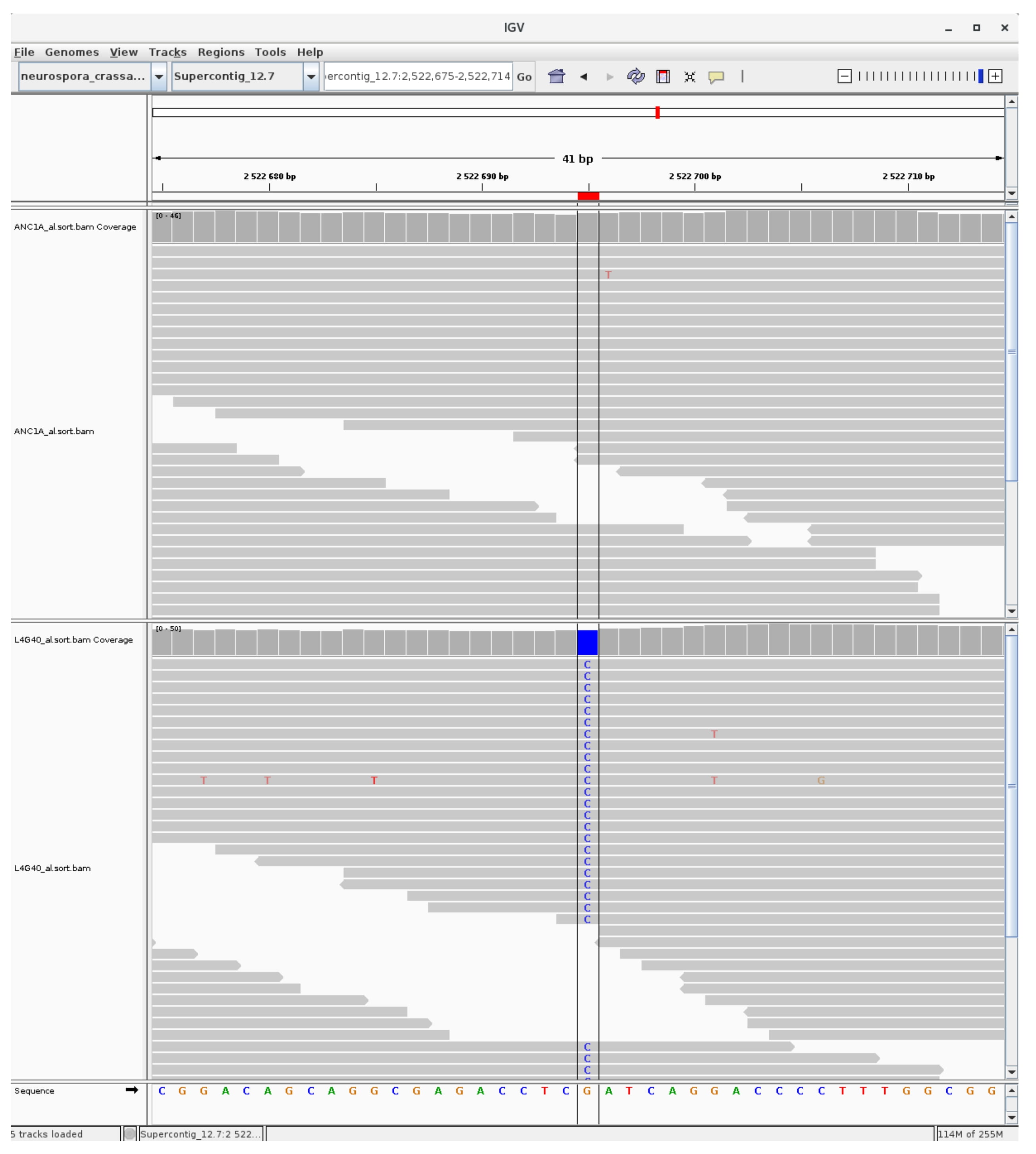

Supplement: Supplemental Material [file supp_gr.276992.122_Supplementary_file_S2.zip › IGV_screenshots/mutation_euchromatic_6.jpg]

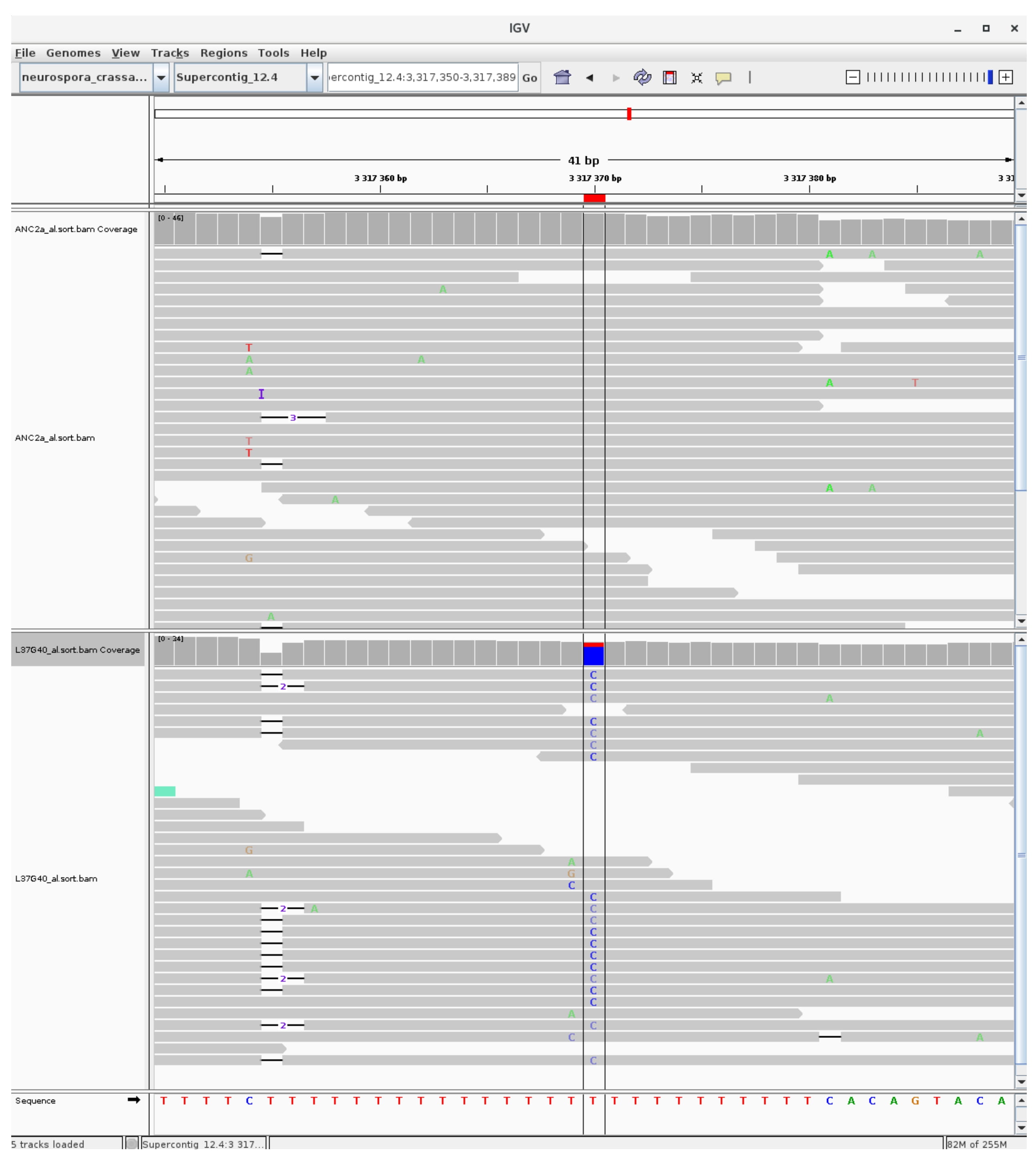

Supplement: Supplemental Material [file supp_gr.276992.122_Supplementary_file_S2.zip › IGV_screenshots/mutation_euchromatic_7.jpg]

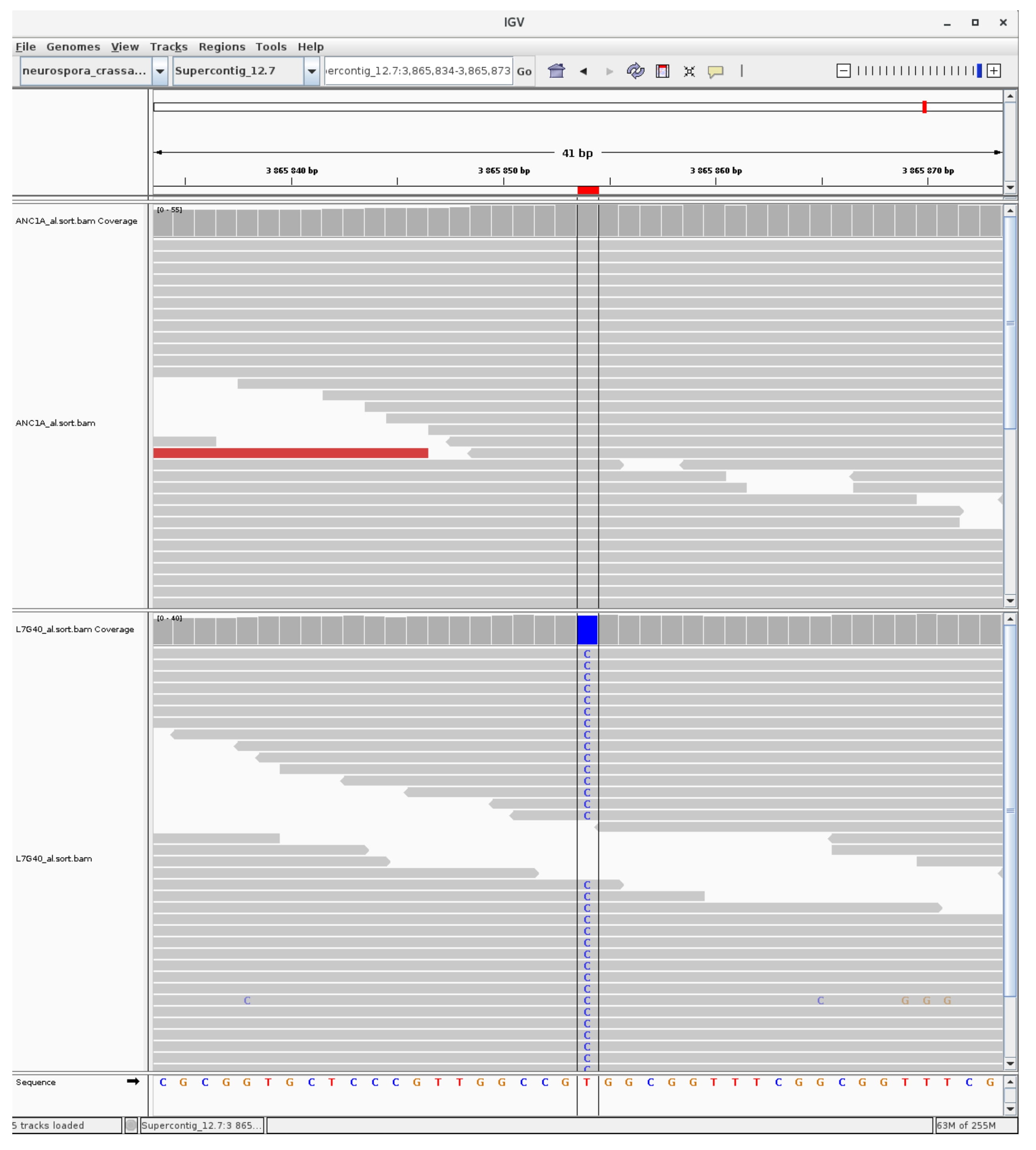

Supplement: Supplemental Material [file supp_gr.276992.122_Supplementary_file_S2.zip › IGV_screenshots/mutation_euchromatic_8.jpg]

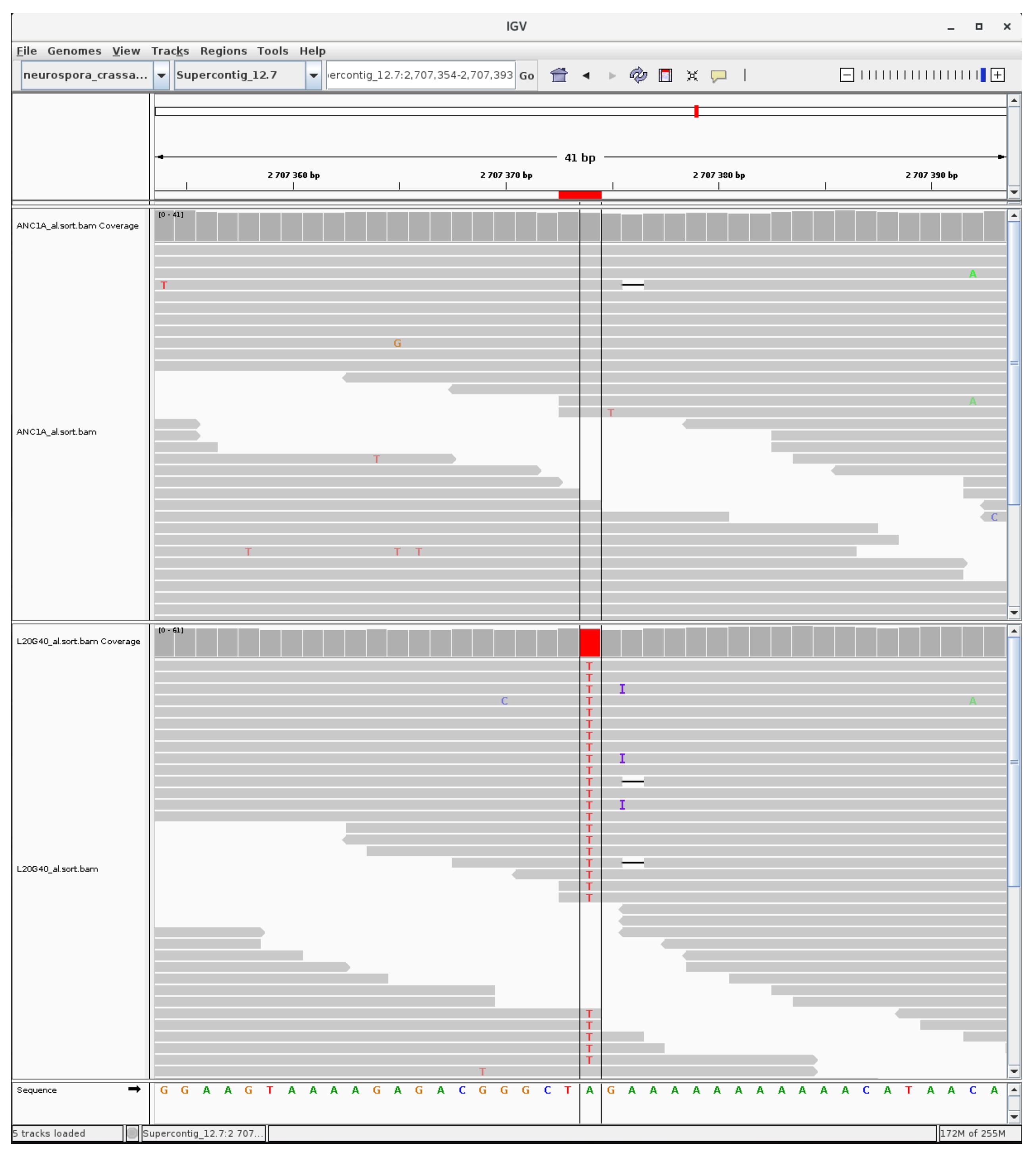

Supplement: Supplemental Material [file supp_gr.276992.122_Supplementary_file_S2.zip › IGV_screenshots/mutation_euchromatic_9.jpg]

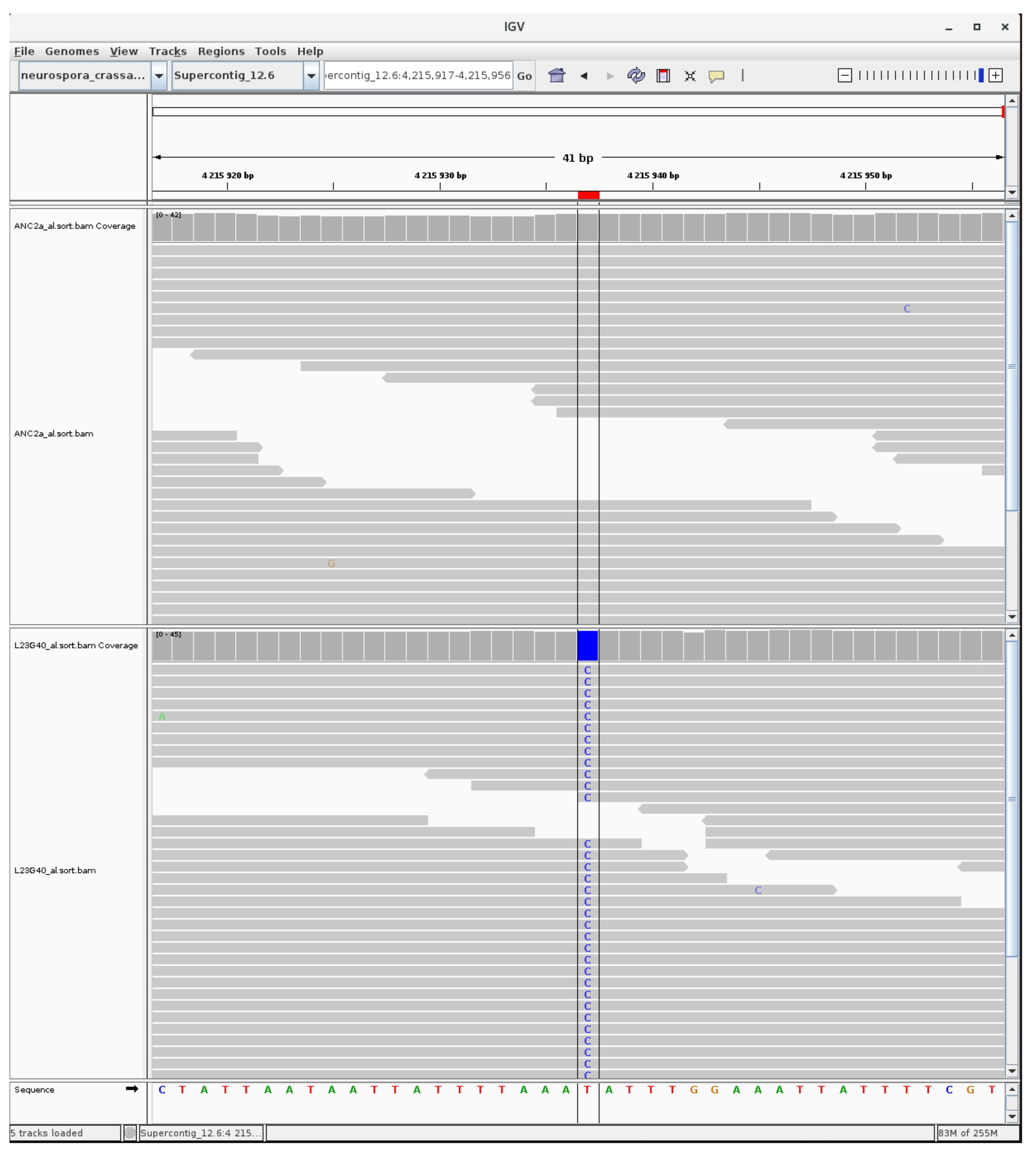

Supplement: Supplemental Material [file supp_gr.276992.122_Supplementary_file_S2.zip › IGV_screenshots/mutation_H3K9_1.jpg]

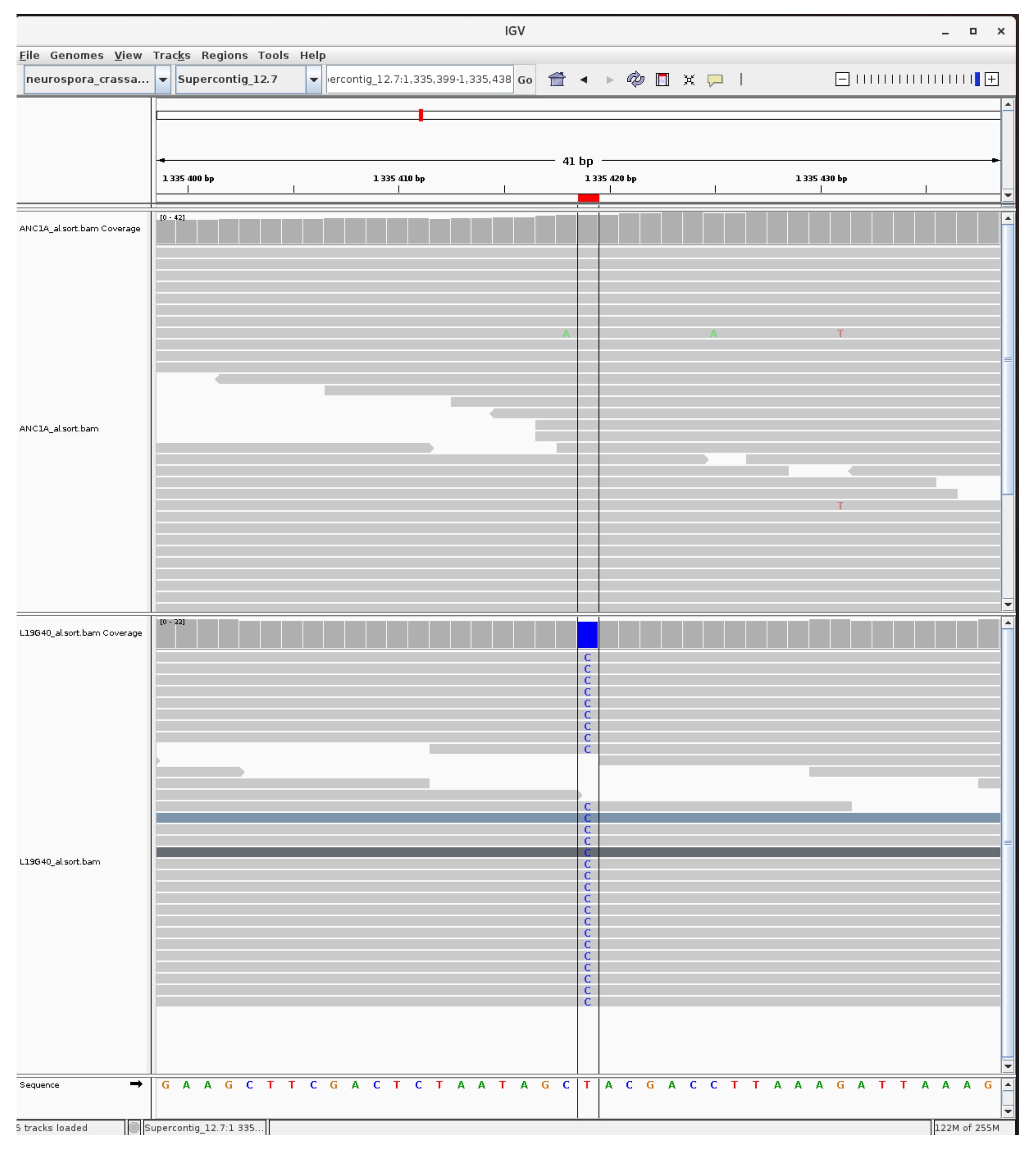

Supplement: Supplemental Material [file supp_gr.276992.122_Supplementary_file_S2.zip › IGV_screenshots/mutation_H3K9_10.jpg]

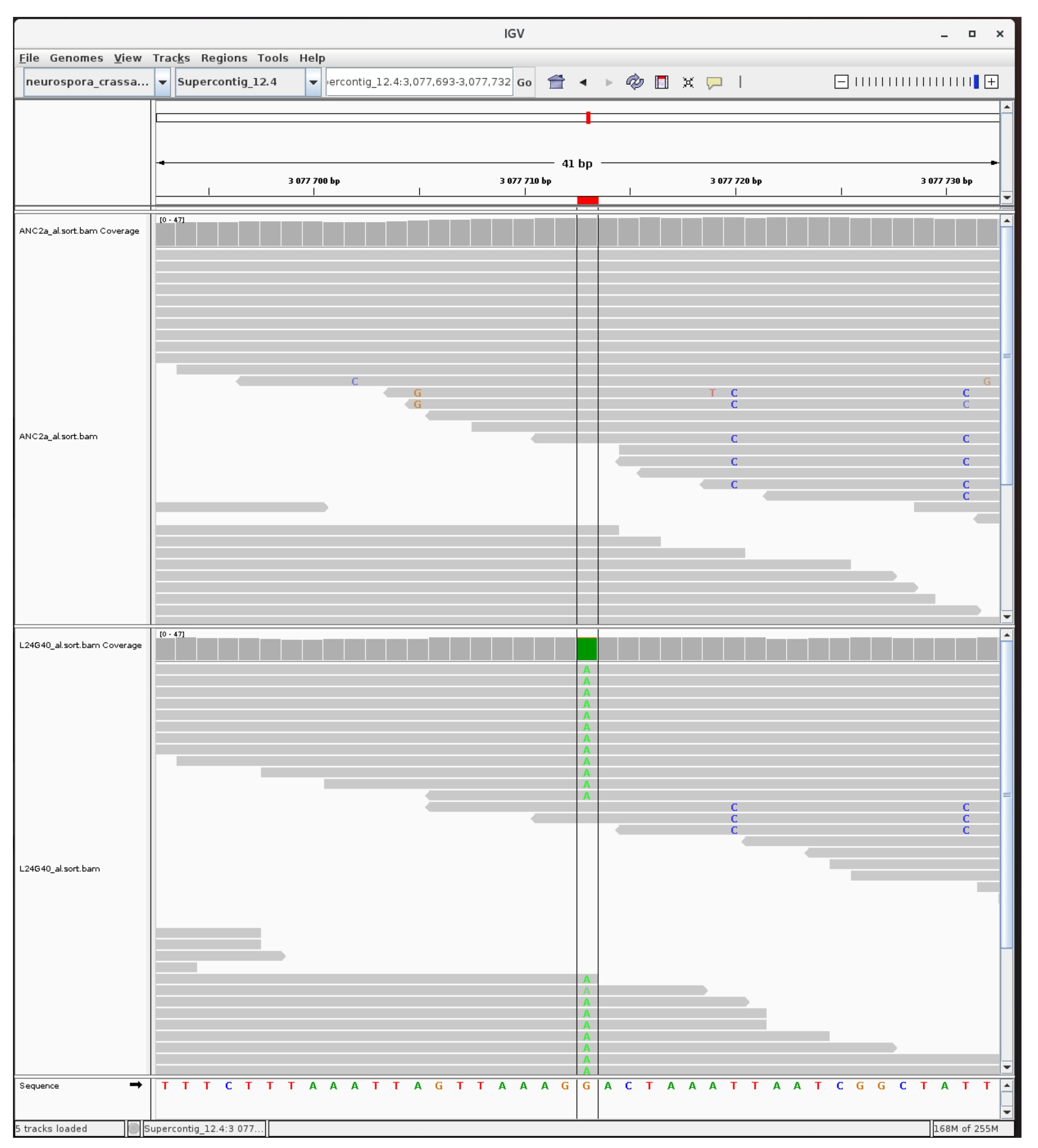

Supplement: Supplemental Material [file supp_gr.276992.122_Supplementary_file_S2.zip › IGV_screenshots/mutation_H3K9_11.jpg]

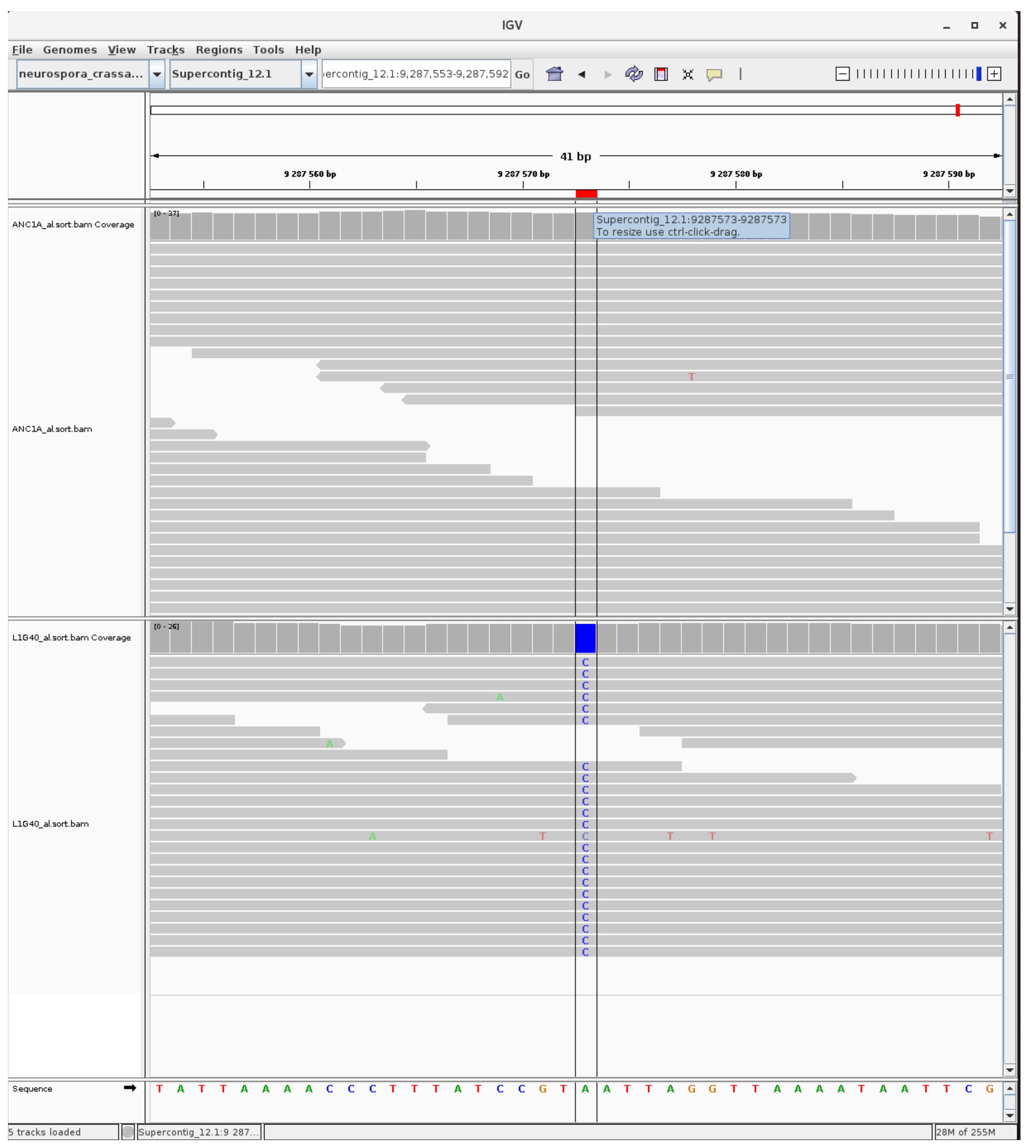

Supplement: Supplemental Material [file supp_gr.276992.122_Supplementary_file_S2.zip › IGV_screenshots/mutation_H3K9_12.jpg]

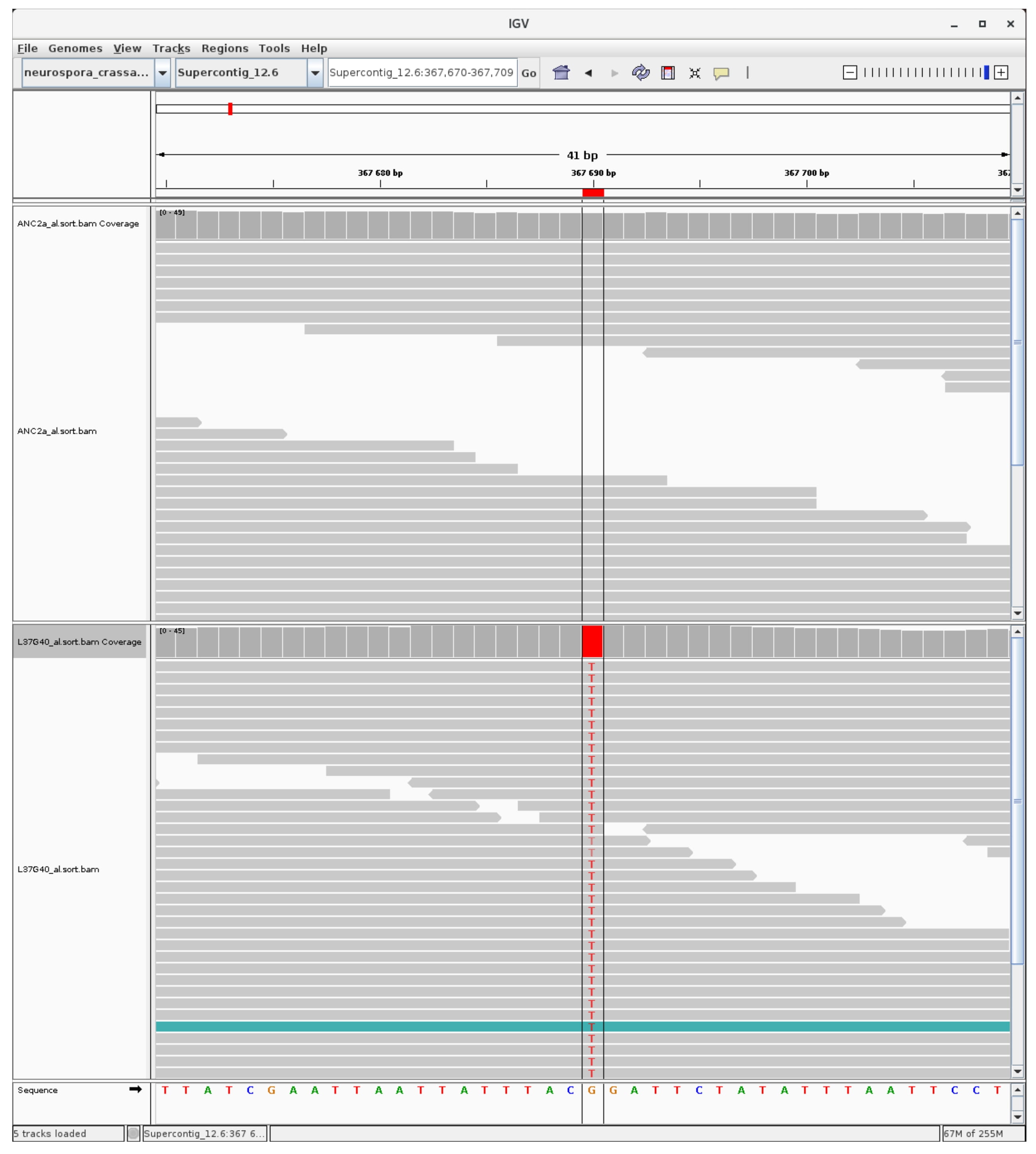

Supplement: Supplemental Material [file supp_gr.276992.122_Supplementary_file_S2.zip › IGV_screenshots/mutation_H3K9_13.jpg]

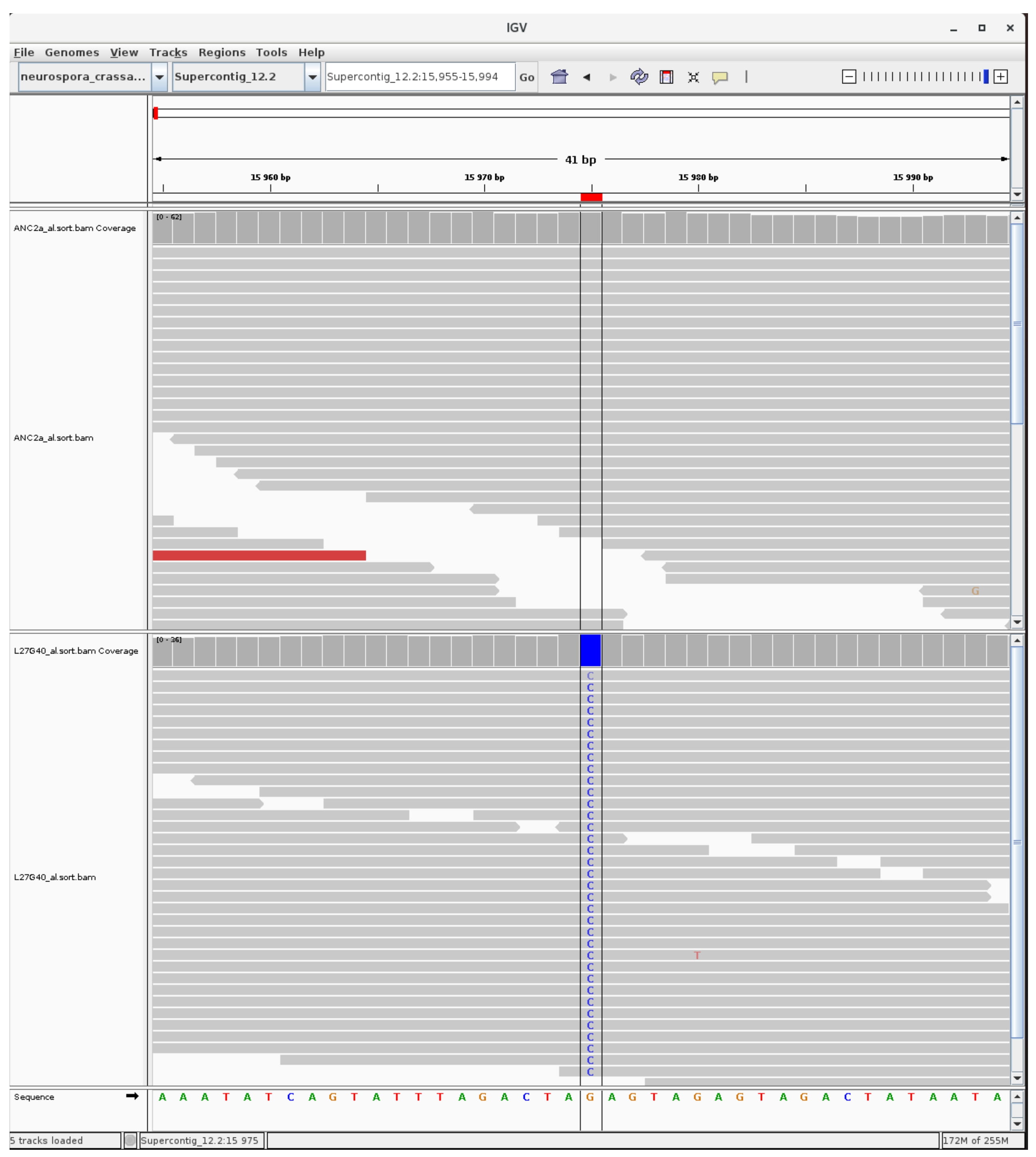

Supplement: Supplemental Material [file supp_gr.276992.122_Supplementary_file_S2.zip › IGV_screenshots/mutation_H3K9_14.jpg]

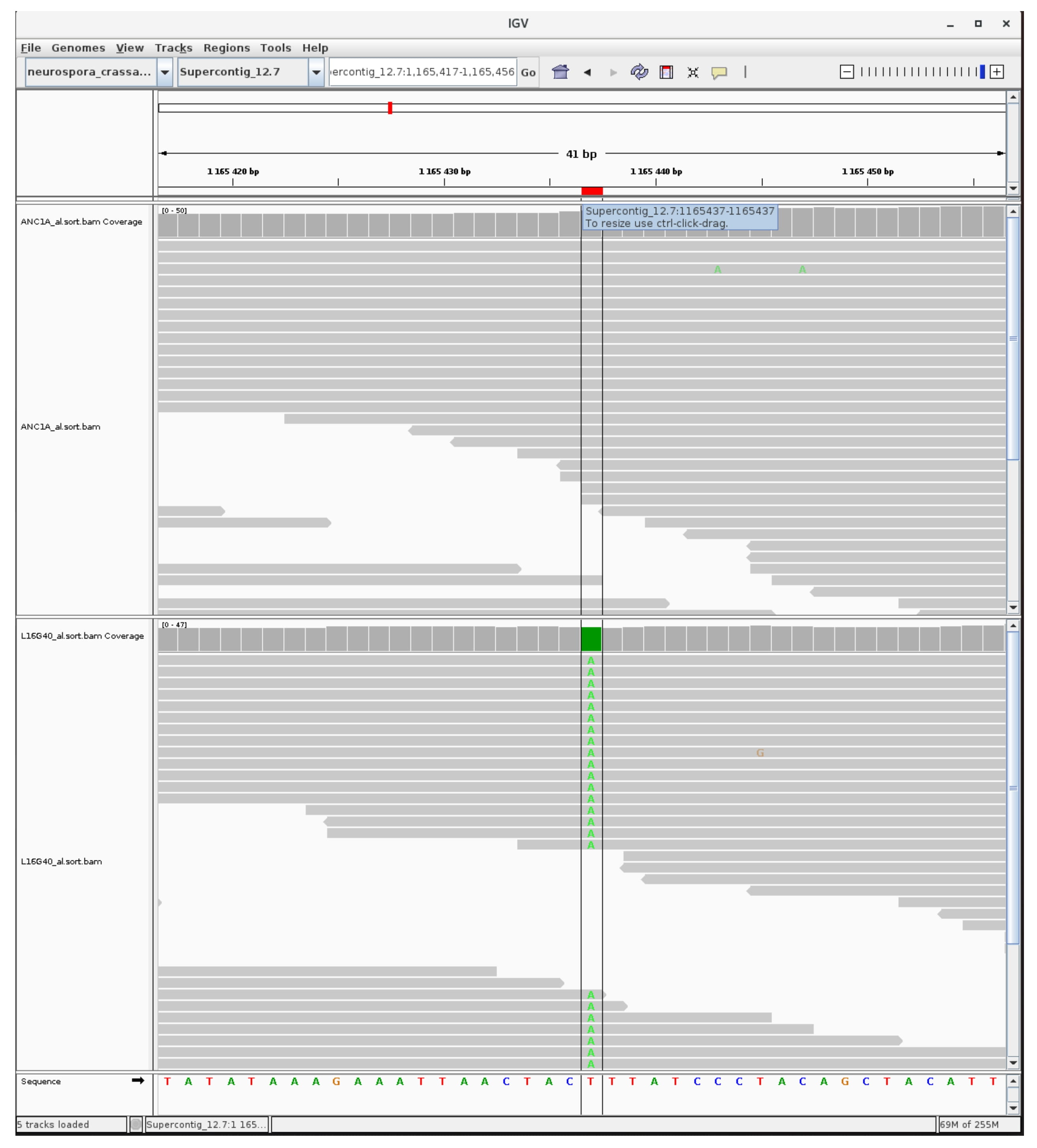

Supplement: Supplemental Material [file supp_gr.276992.122_Supplementary_file_S2.zip › IGV_screenshots/mutation_H3K9_15.jpg]

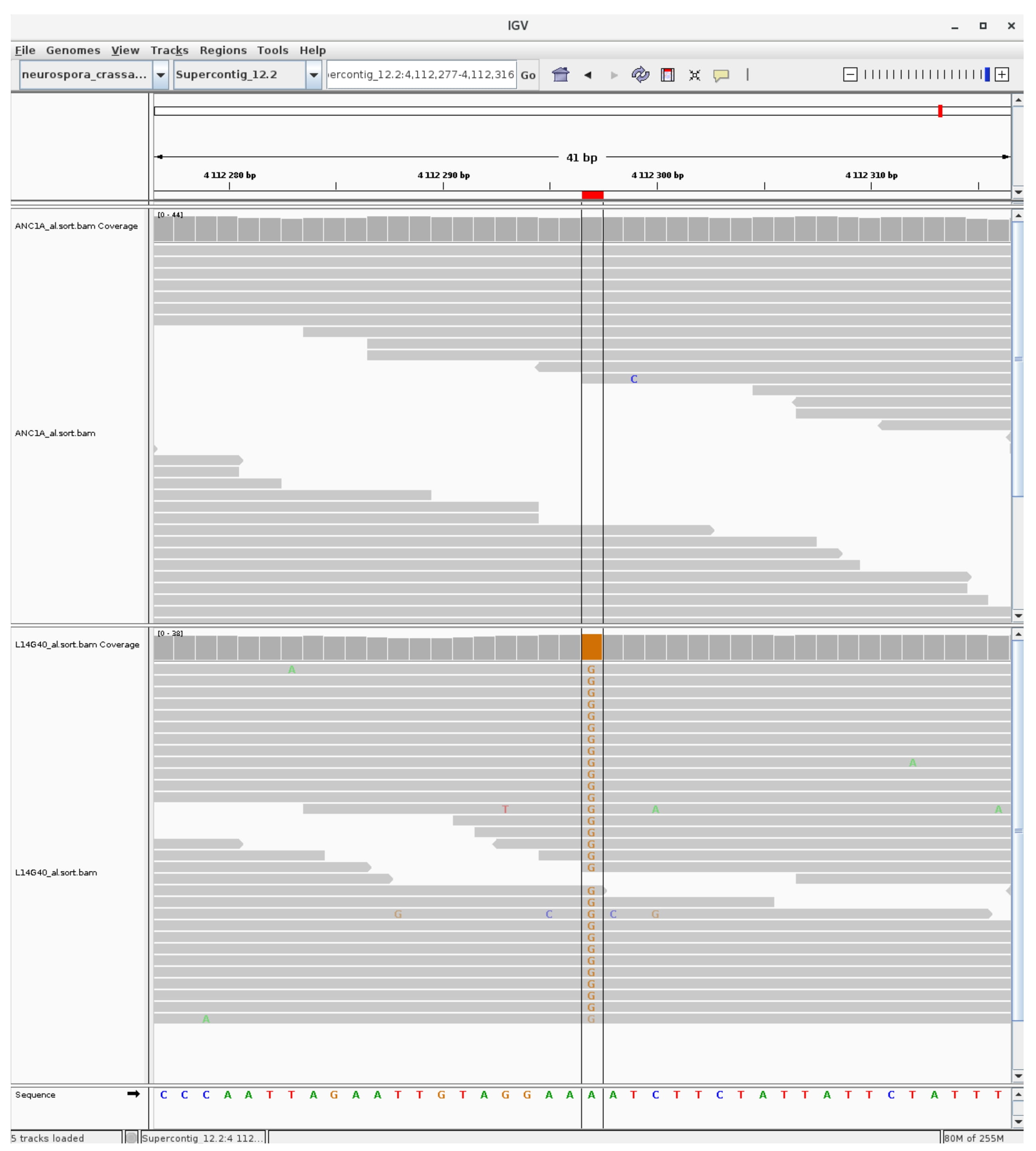

Supplement: Supplemental Material [file supp_gr.276992.122_Supplementary_file_S2.zip › IGV_screenshots/mutation_H3K9_16.jpg]

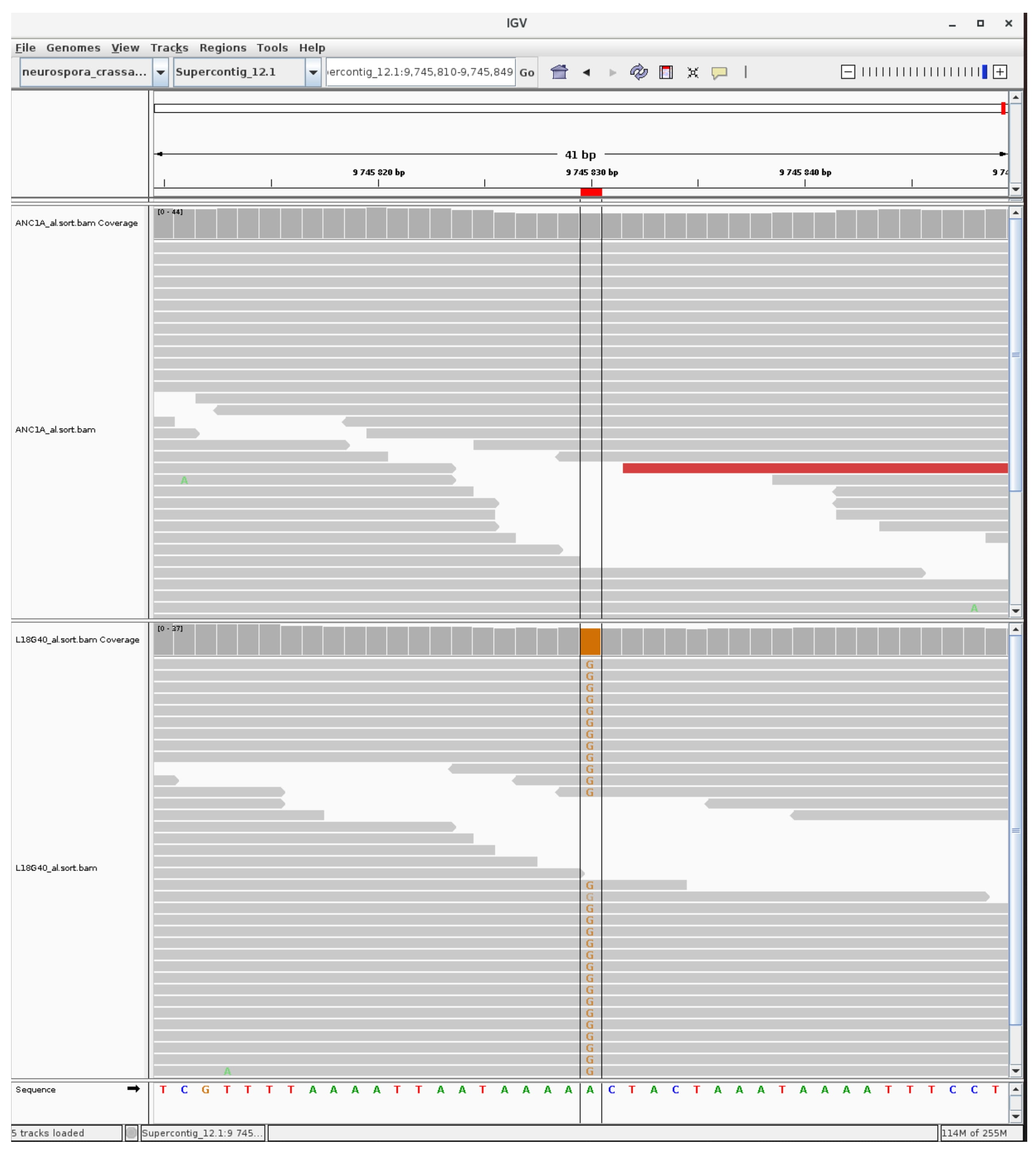

Supplement: Supplemental Material [file supp_gr.276992.122_Supplementary_file_S2.zip › IGV_screenshots/mutation_H3K9_17.jpg]

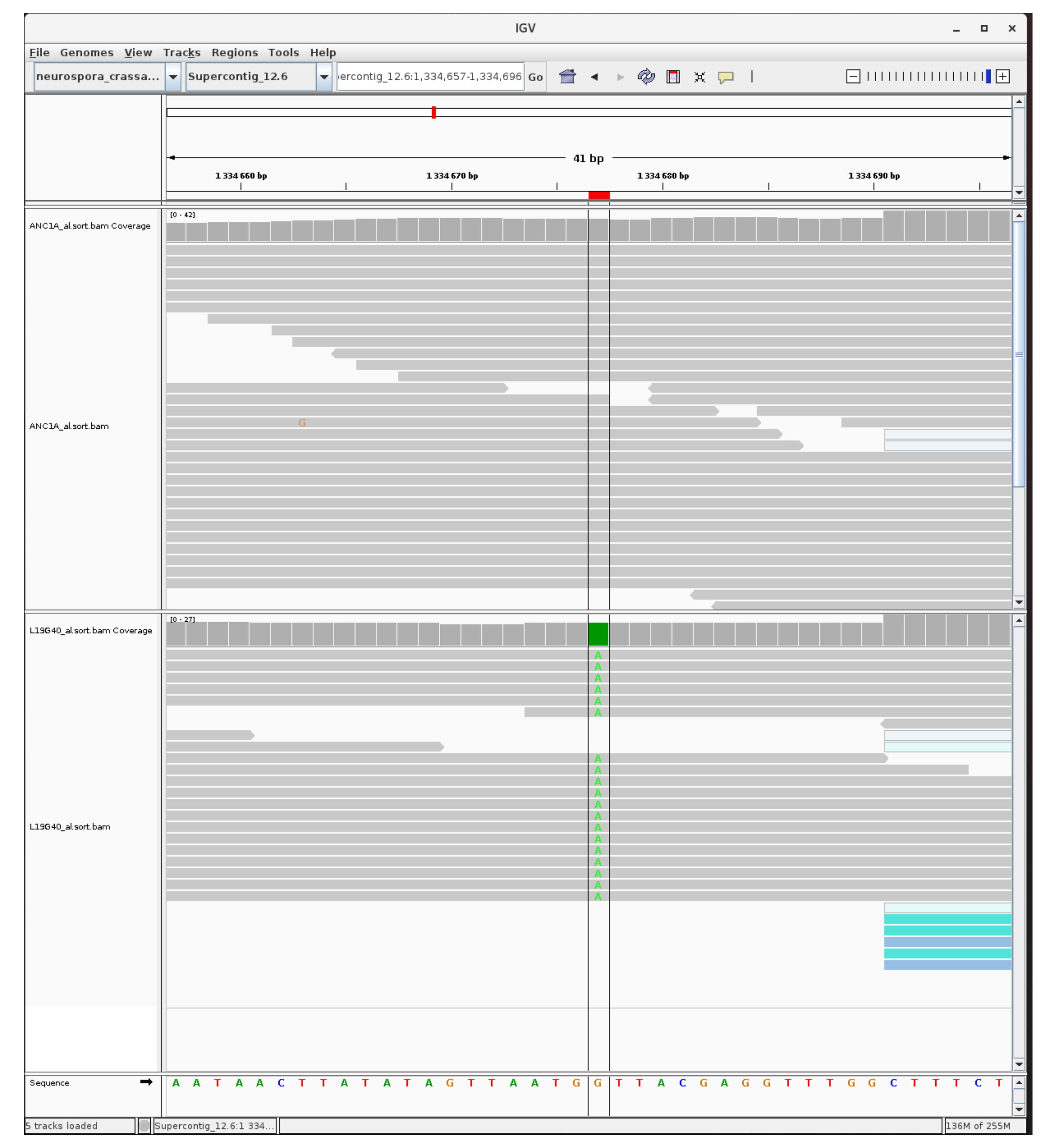

Supplement: Supplemental Material [file supp_gr.276992.122_Supplementary_file_S2.zip › IGV_screenshots/mutation_H3K9_18.jpg]

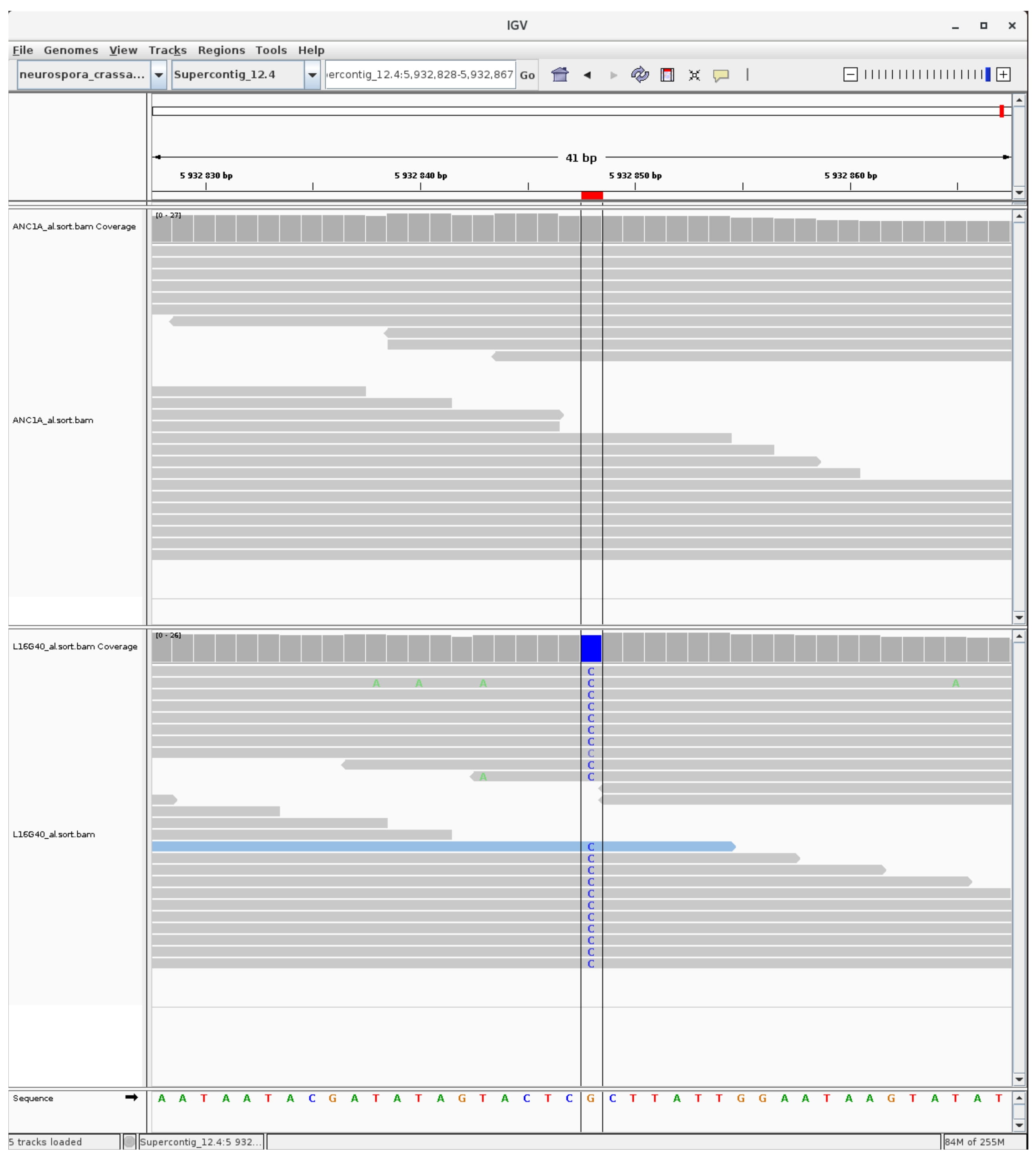

Supplement: Supplemental Material [file supp_gr.276992.122_Supplementary_file_S2.zip › IGV_screenshots/mutation_H3K9_19.jpg]

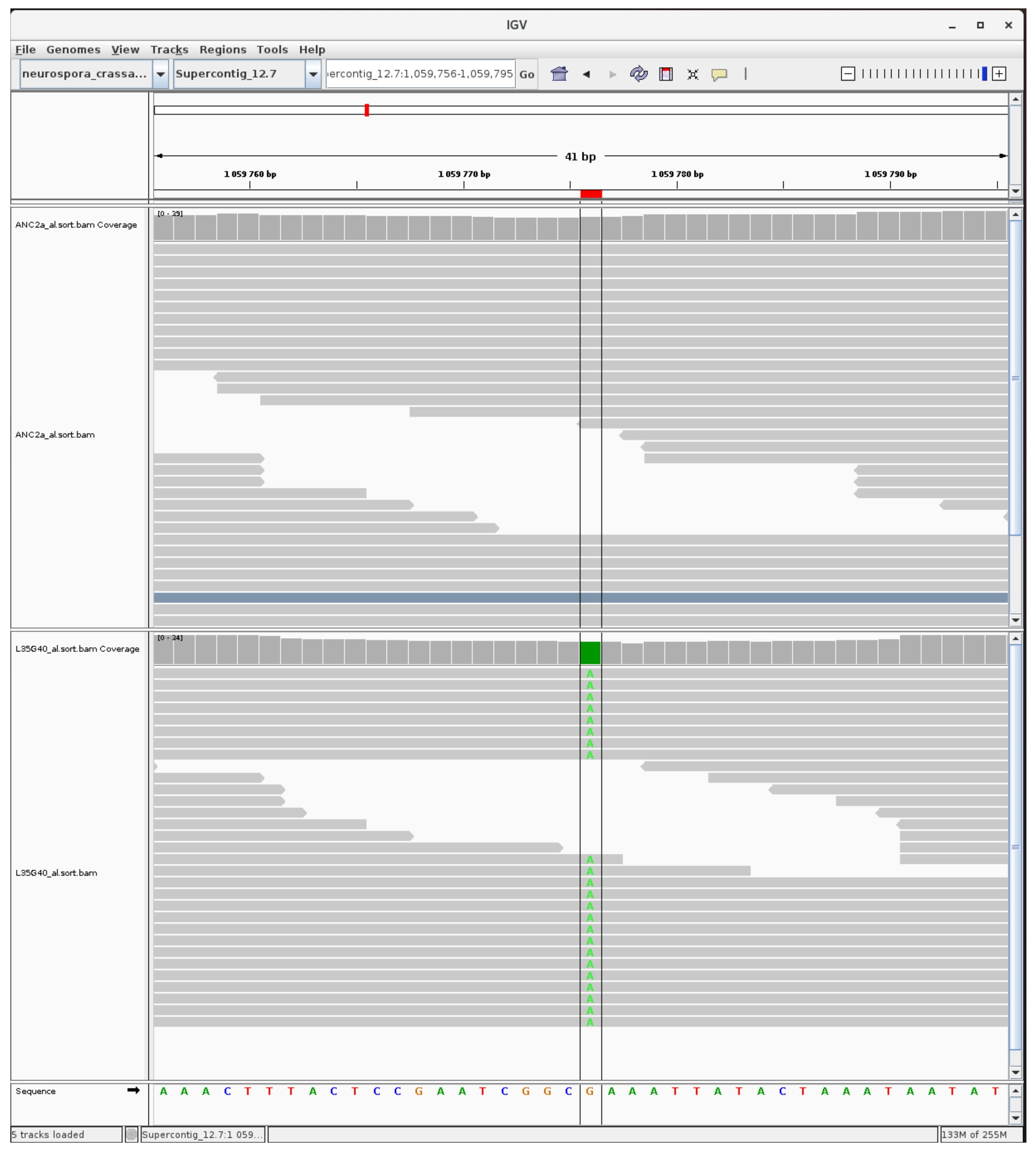

Supplement: Supplemental Material [file supp_gr.276992.122_Supplementary_file_S2.zip › IGV_screenshots/mutation_H3K9_2.jpg]

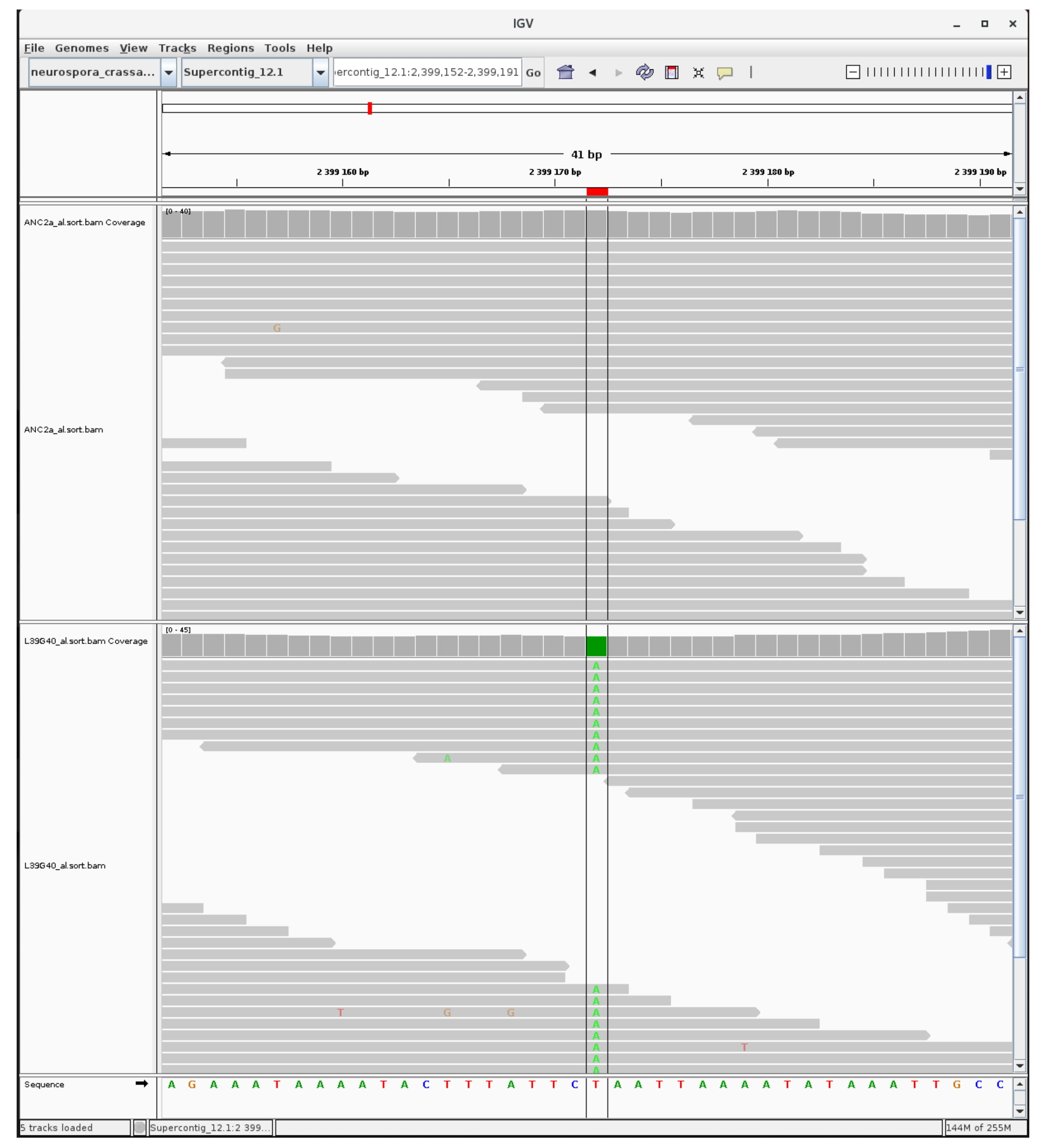

Supplement: Supplemental Material [file supp_gr.276992.122_Supplementary_file_S2.zip › IGV_screenshots/mutation_H3K9_20.jpg]

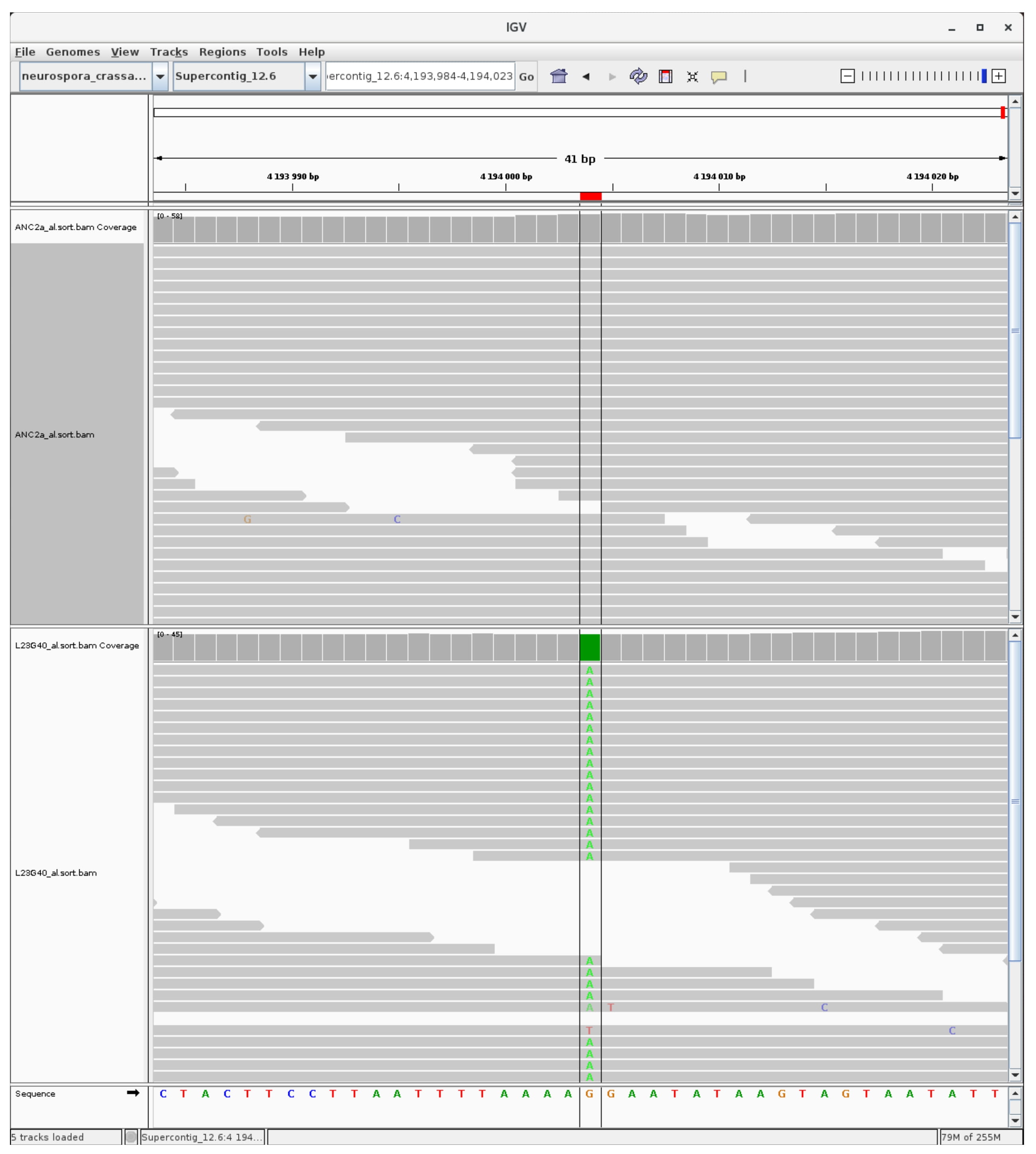

Supplement: Supplemental Material [file supp_gr.276992.122_Supplementary_file_S2.zip › IGV_screenshots/mutation_H3K9_21.jpg]

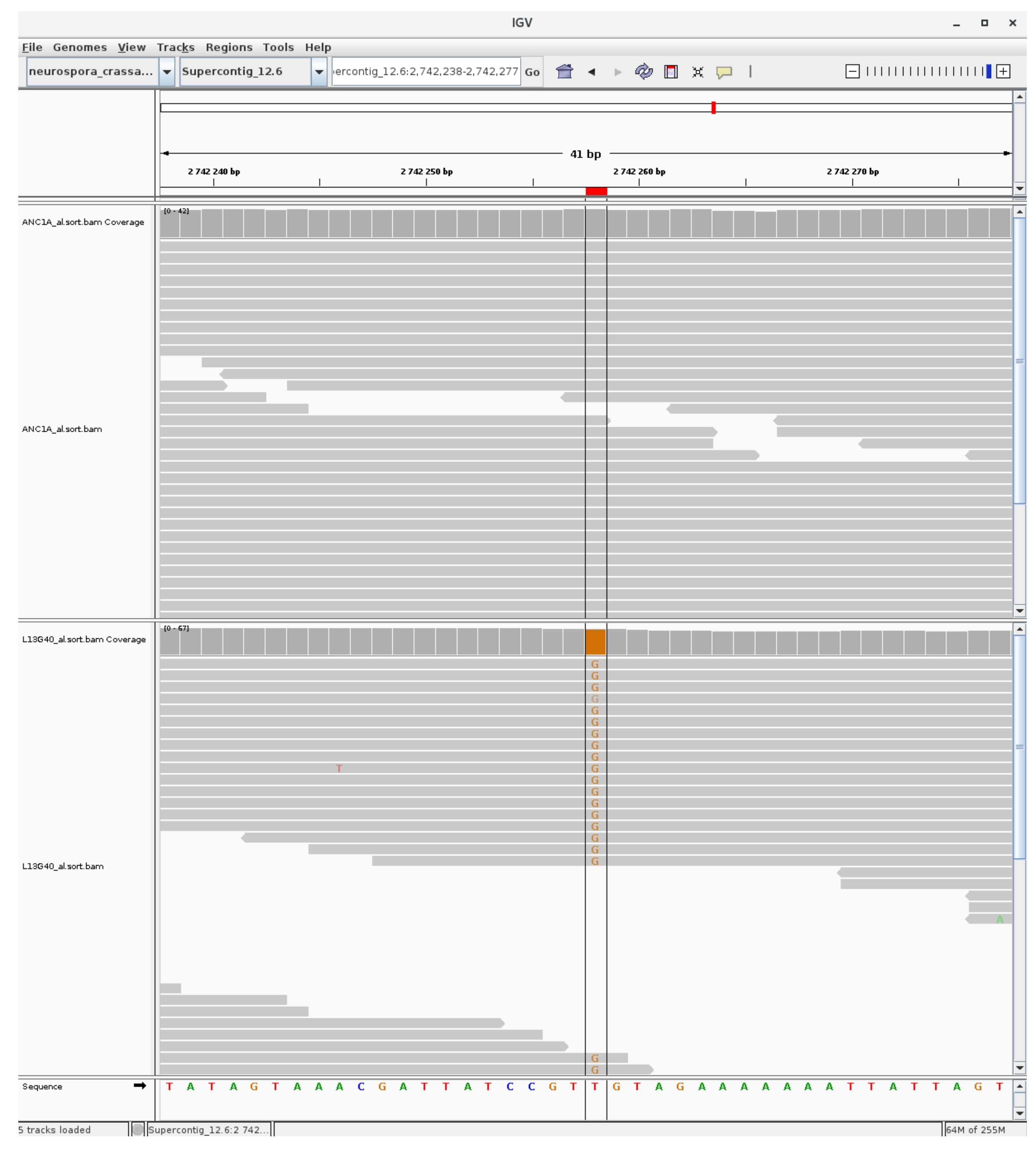

Supplement: Supplemental Material [file supp_gr.276992.122_Supplementary_file_S2.zip › IGV_screenshots/mutation_H3K9_22.jpg]

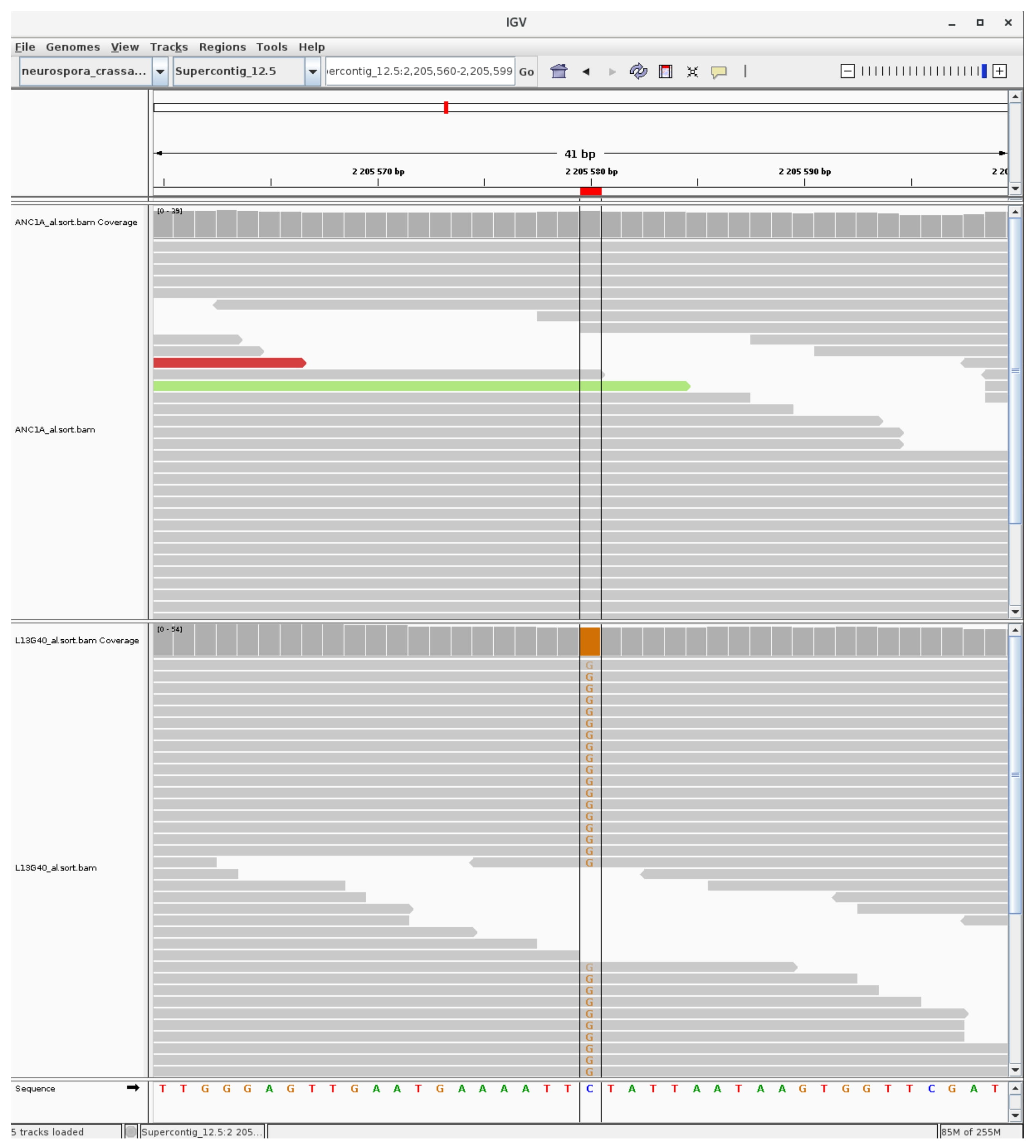

Supplement: Supplemental Material [file supp_gr.276992.122_Supplementary_file_S2.zip › IGV_screenshots/mutation_H3K9_23.jpg]

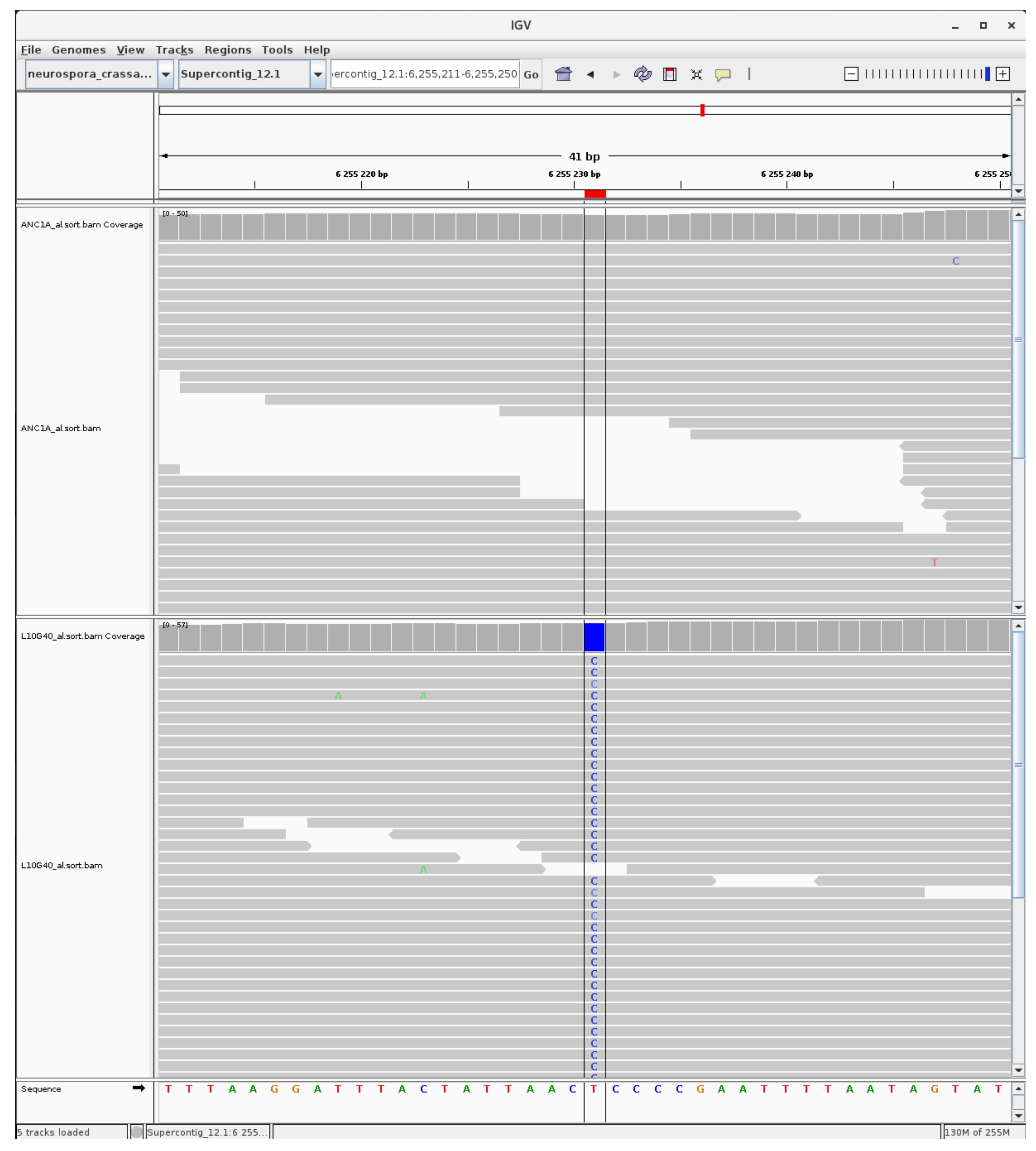

Supplement: Supplemental Material [file supp_gr.276992.122_Supplementary_file_S2.zip › IGV_screenshots/mutation_H3K9_24.jpg]

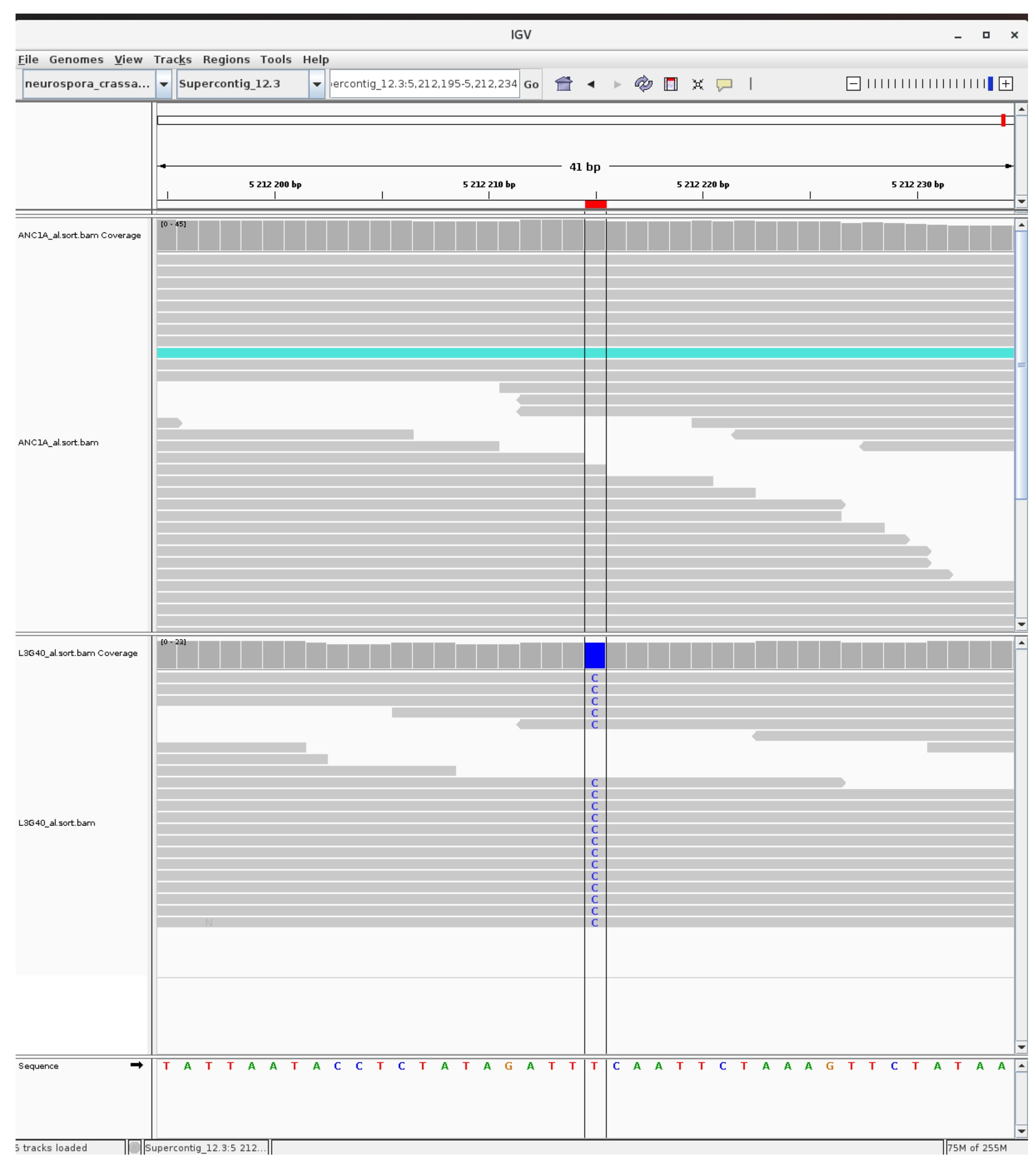

Supplement: Supplemental Material [file supp_gr.276992.122_Supplementary_file_S2.zip › IGV_screenshots/mutation_H3K9_25.jpg]

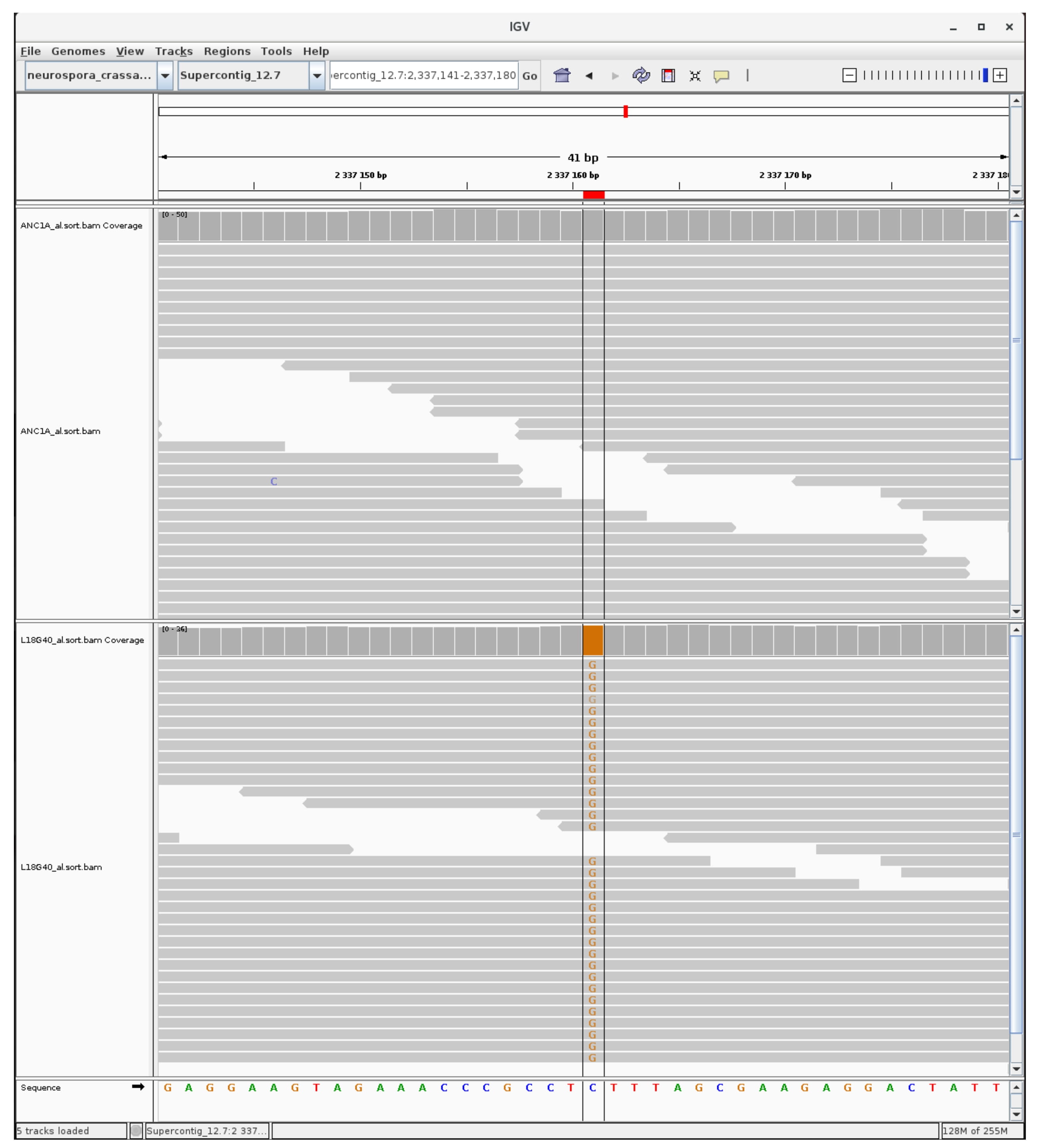

Supplement: Supplemental Material [file supp_gr.276992.122_Supplementary_file_S2.zip › IGV_screenshots/mutation_H3K9_26.jpg]

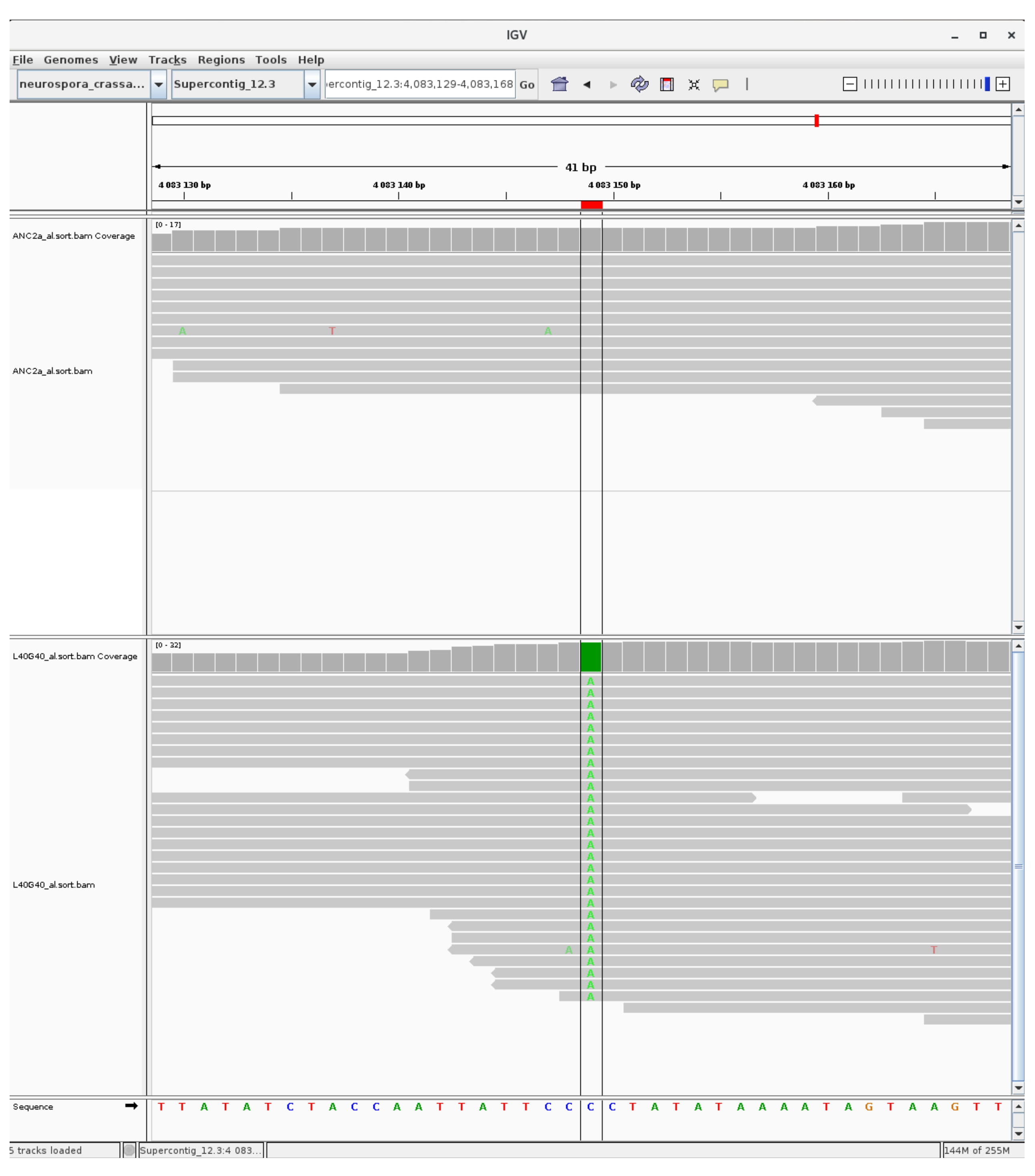

Supplement: Supplemental Material [file supp_gr.276992.122_Supplementary_file_S2.zip › IGV_screenshots/mutation_H3K9_27.jpg]

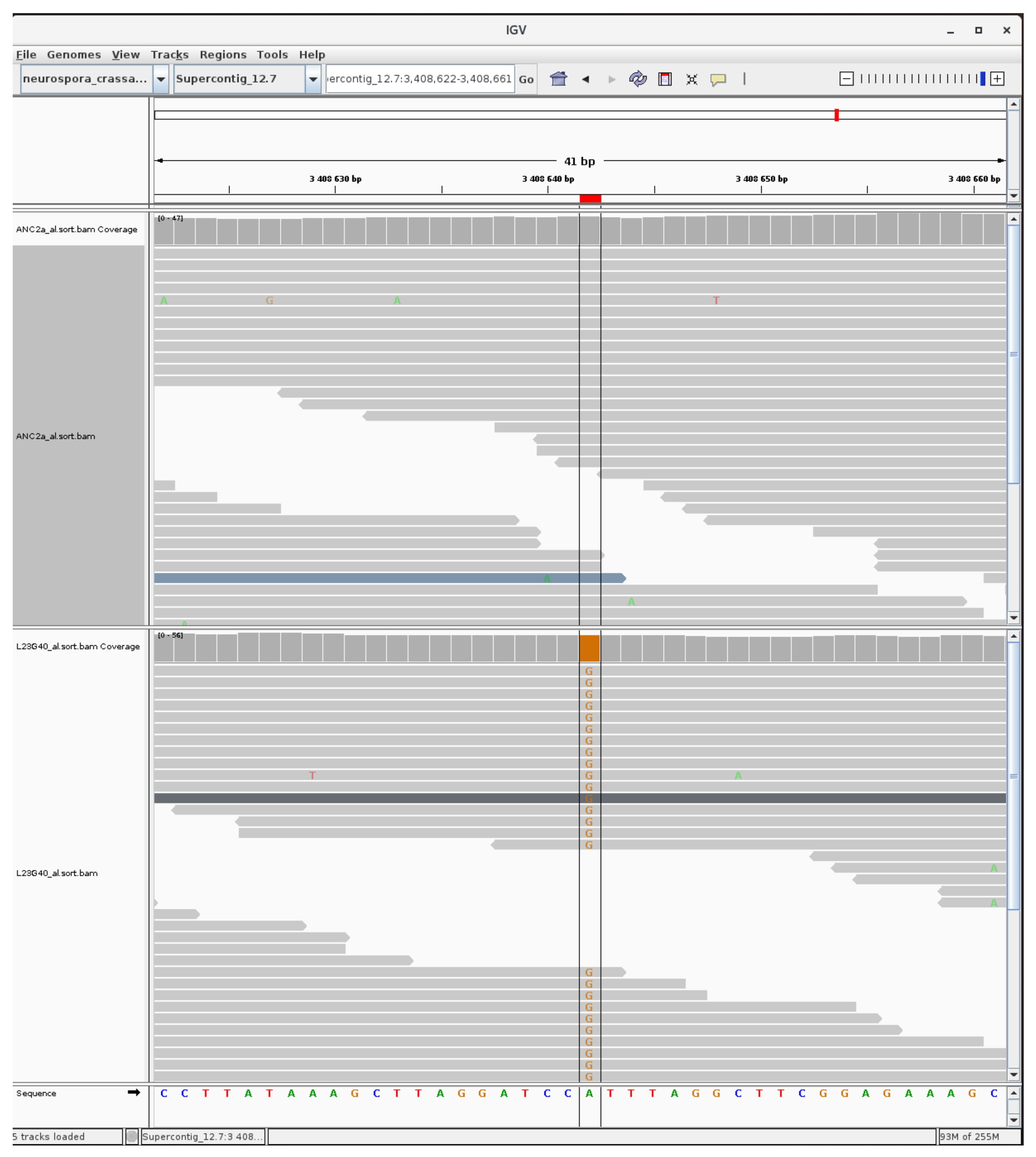

Supplement: Supplemental Material [file supp_gr.276992.122_Supplementary_file_S2.zip › IGV_screenshots/mutation_H3K9_28.jpg]

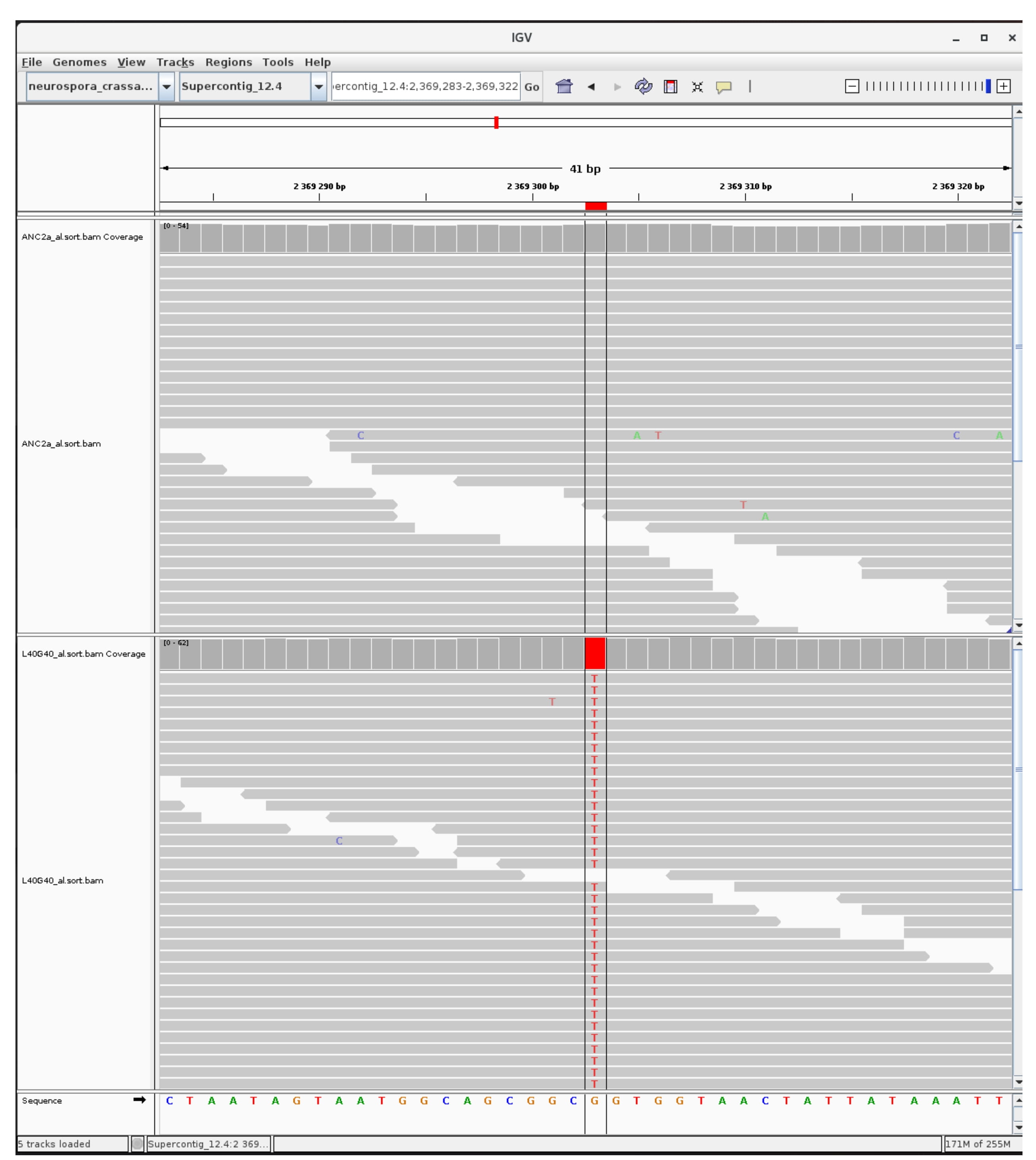

Supplement: Supplemental Material [file supp_gr.276992.122_Supplementary_file_S2.zip › IGV_screenshots/mutation_H3K9_29.jpg]

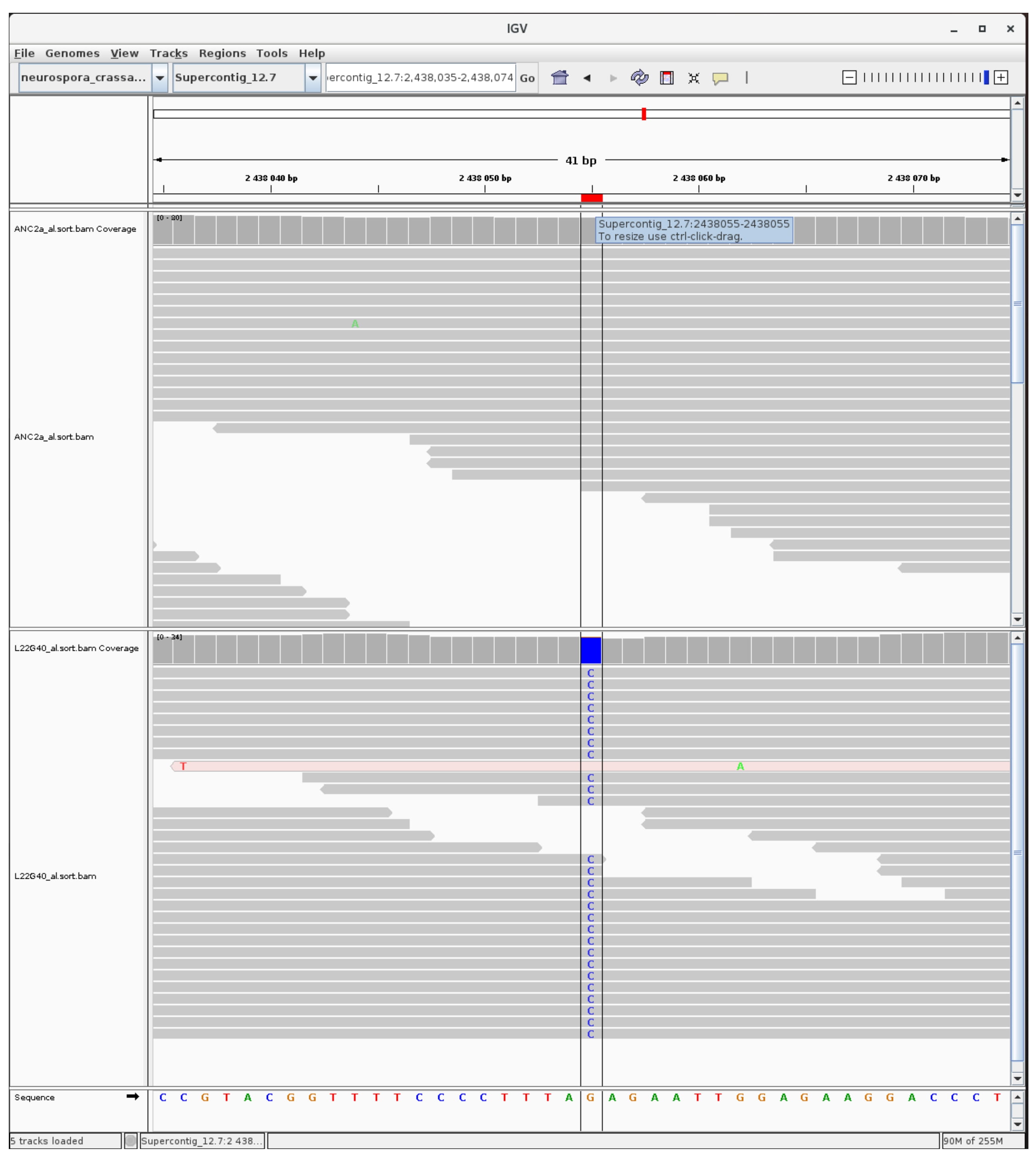

Supplement: Supplemental Material [file supp_gr.276992.122_Supplementary_file_S2.zip › IGV_screenshots/mutation_H3K9_3.jpg]

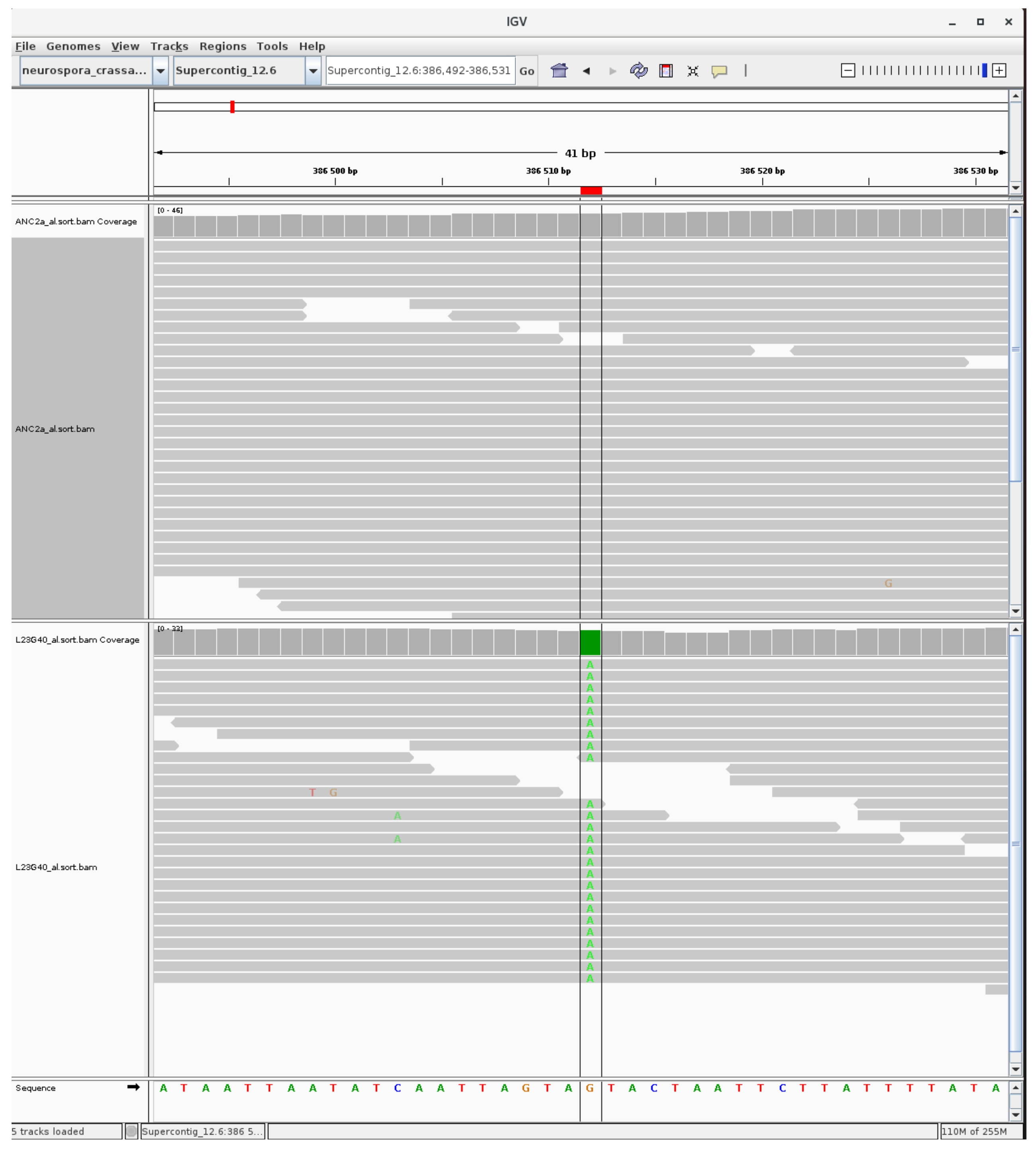

Supplement: Supplemental Material [file supp_gr.276992.122_Supplementary_file_S2.zip › IGV_screenshots/mutation_H3K9_30.jpg]

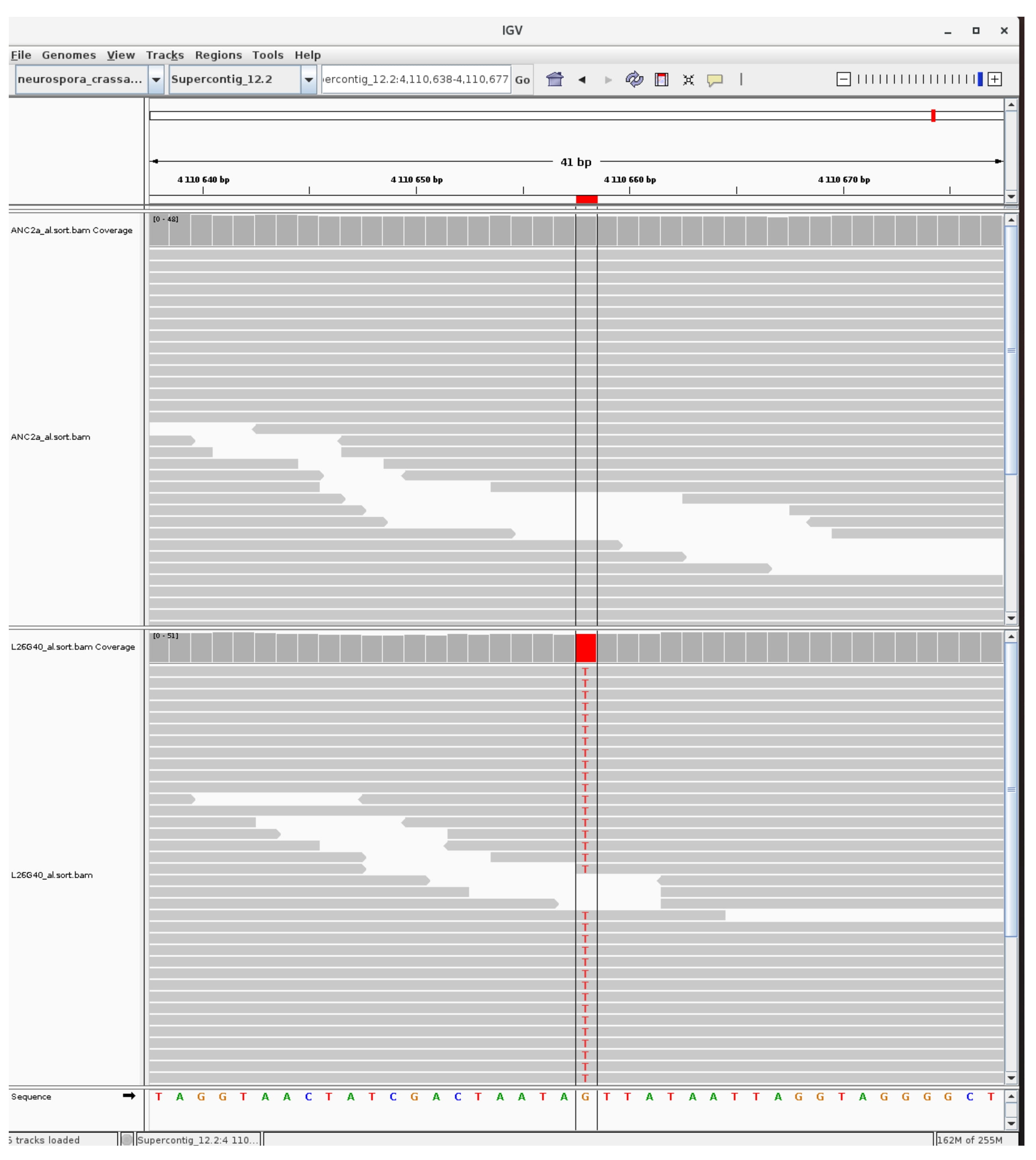

Supplement: Supplemental Material [file supp_gr.276992.122_Supplementary_file_S2.zip › IGV_screenshots/mutation_H3K9_4.jpg]

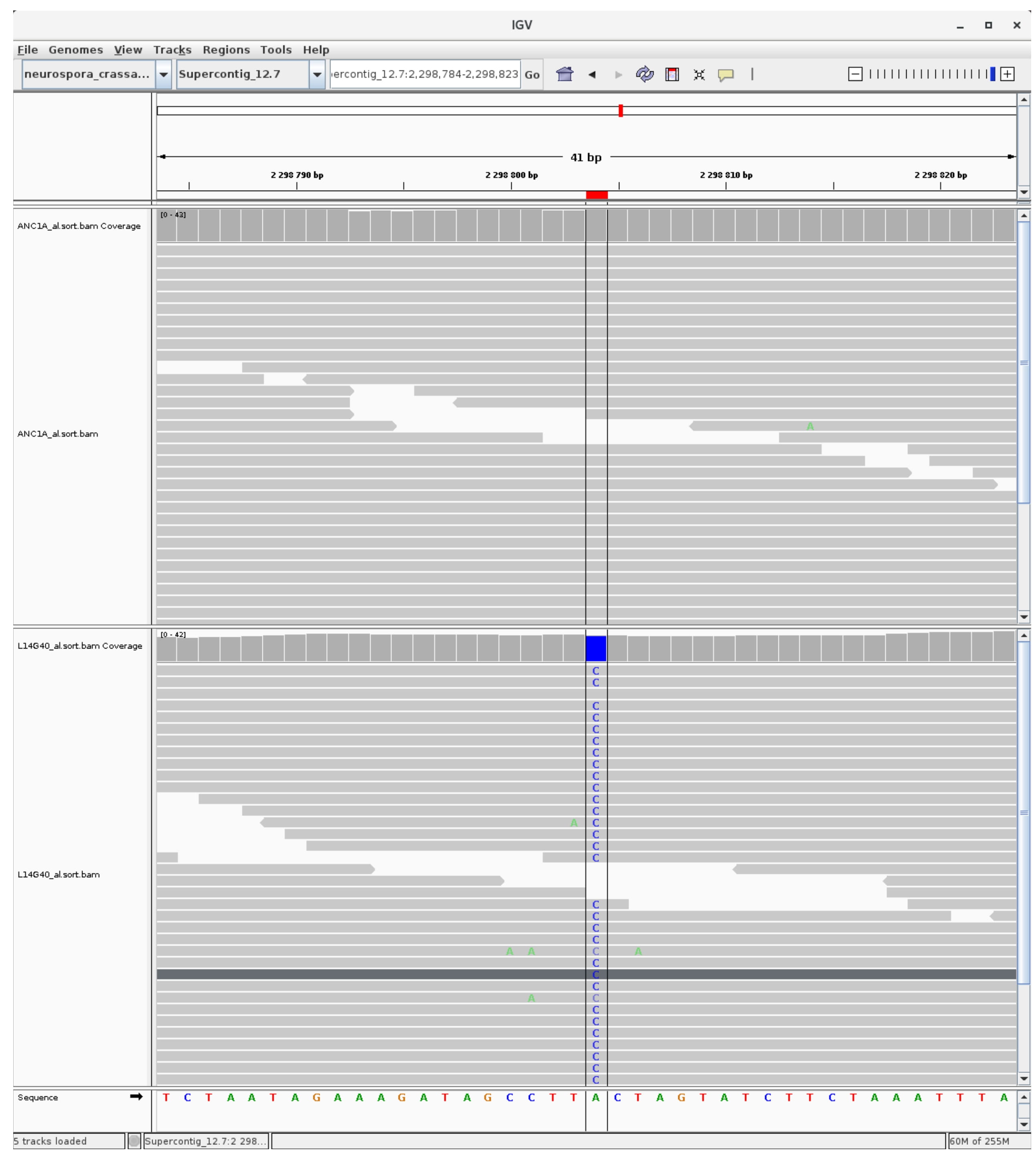

Supplement: Supplemental Material [file supp_gr.276992.122_Supplementary_file_S2.zip › IGV_screenshots/mutation_H3K9_5.jpg]

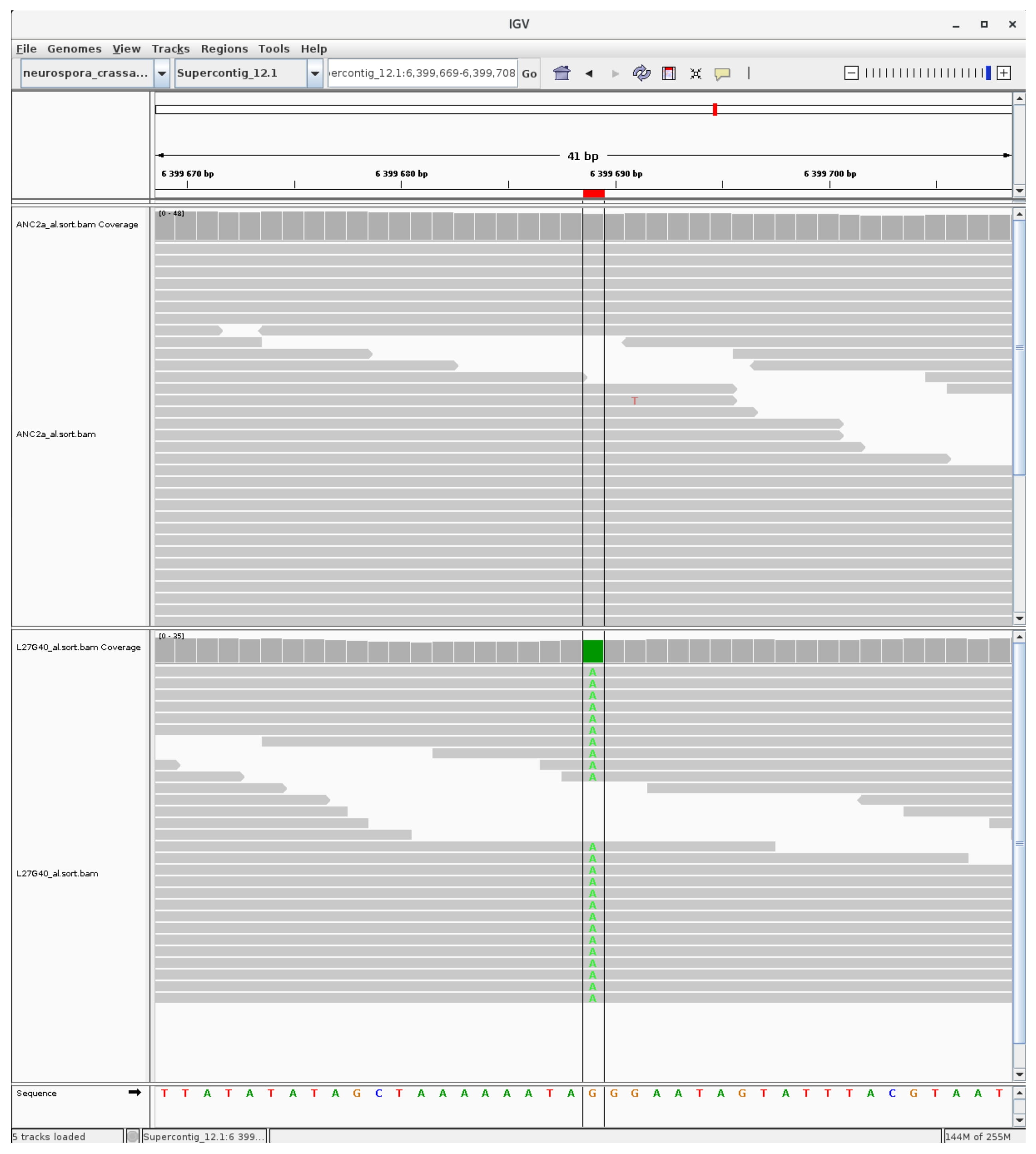

Supplement: Supplemental Material [file supp_gr.276992.122_Supplementary_file_S2.zip › IGV_screenshots/mutation_H3K9_6.jpg]

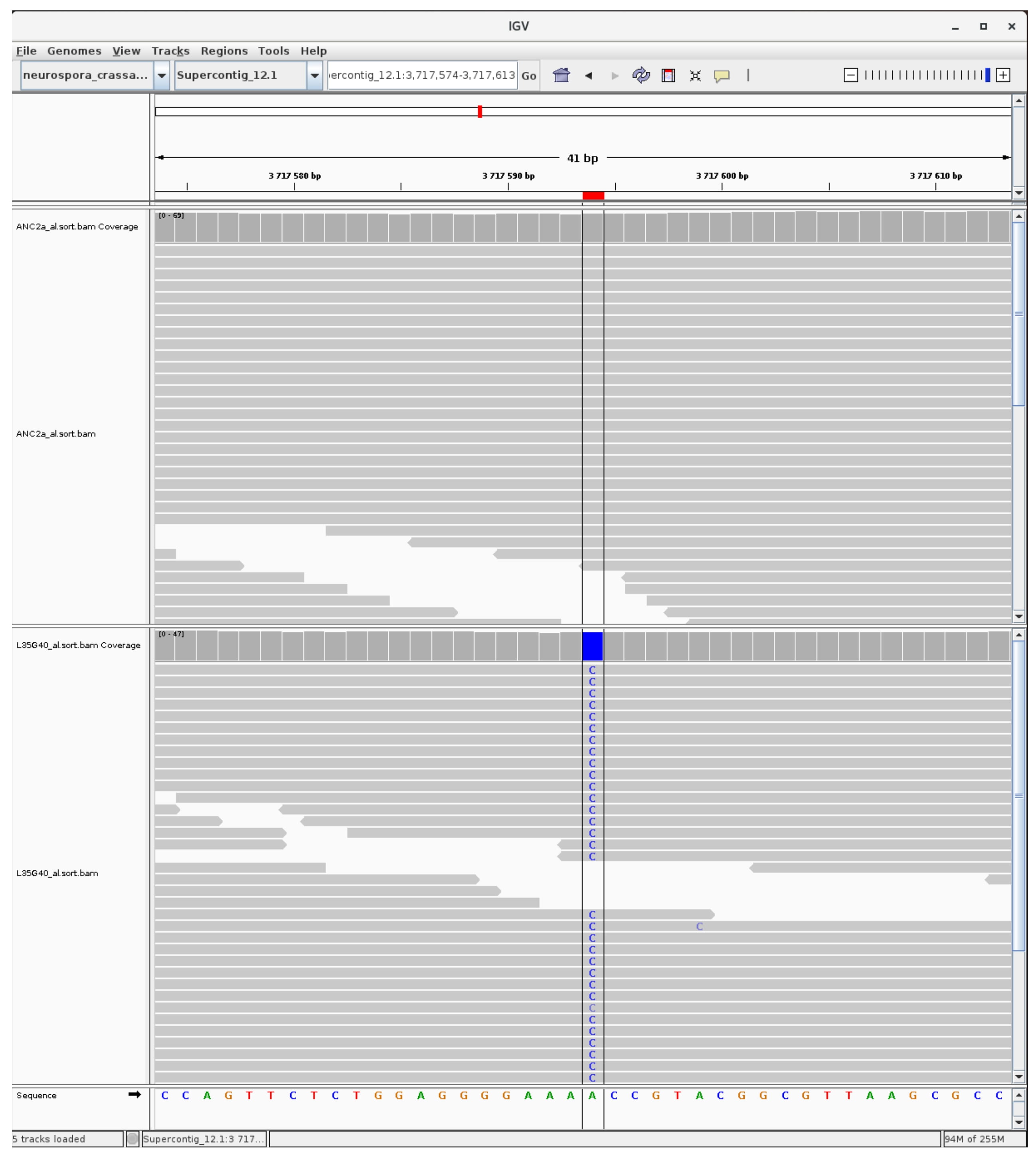

Supplement: Supplemental Material [file supp_gr.276992.122_Supplementary_file_S2.zip › IGV_screenshots/mutation_H3K9_7.jpg]

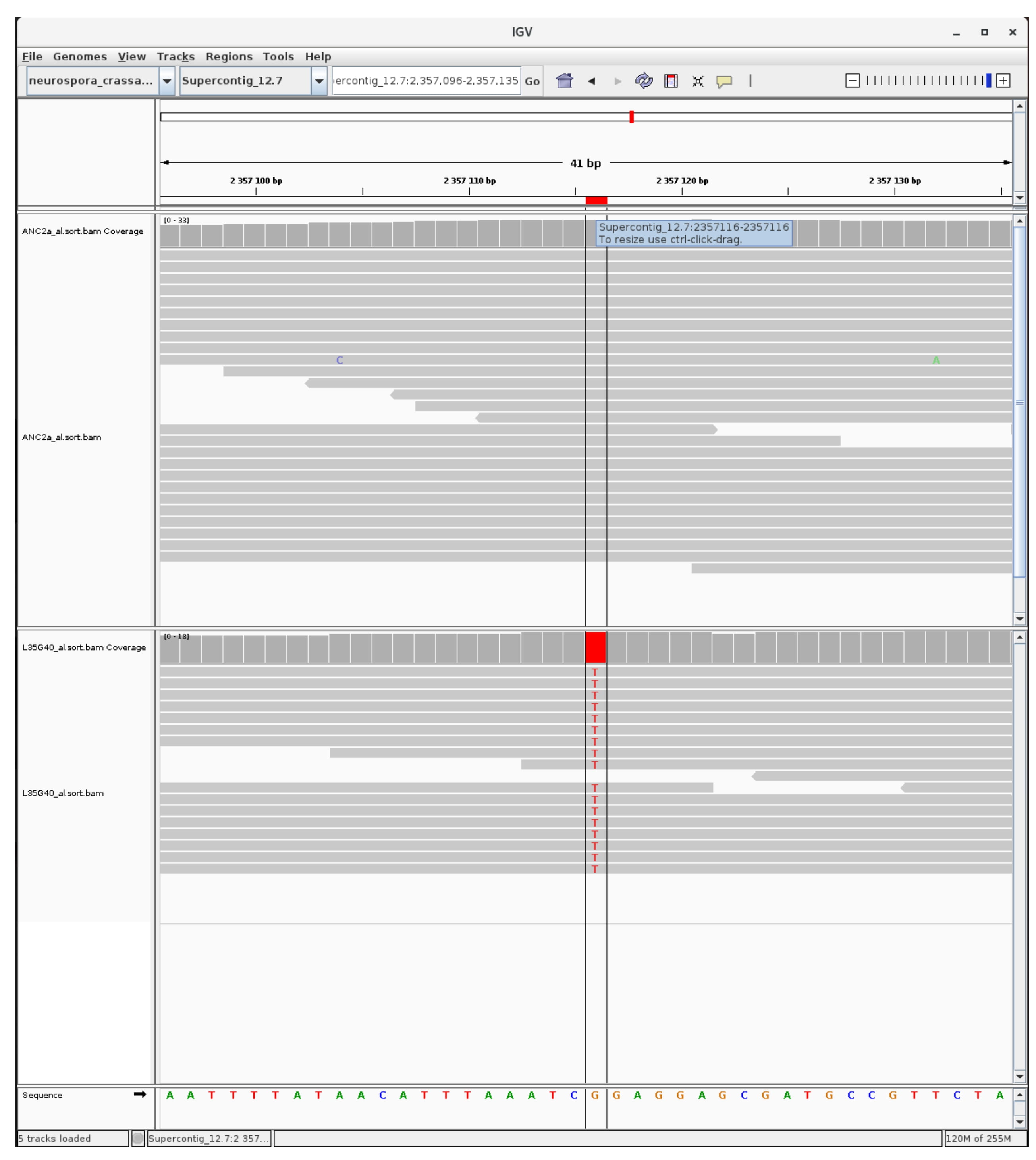

Supplement: Supplemental Material [file supp_gr.276992.122_Supplementary_file_S2.zip › IGV_screenshots/mutation_H3K9_8.jpg]

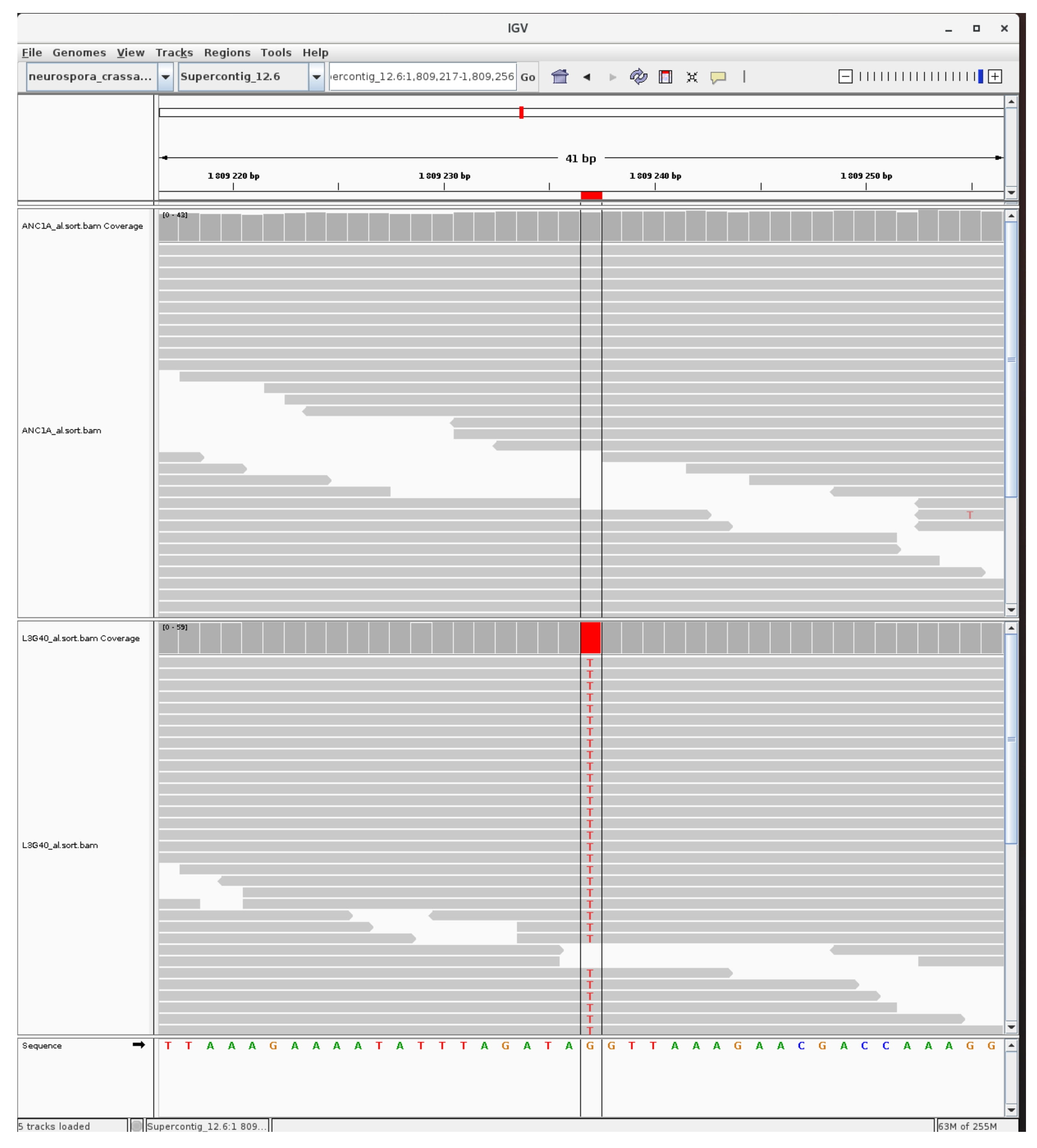

Supplement: Supplemental Material [file supp_gr.276992.122_Supplementary_file_S2.zip › IGV_screenshots/mutation_H3K9_9.jpg]
