# Supplementary material for: Chromatin structure influences rate and spectrum of spontaneous mutations in Neurospora crassa
Source: Genome Res. 2023 Apr;33(4):599–611. doi: 10.1101/gr.276992.122 (PMC10234303; doi:10.1101/gr.276992.122)
Supplement: Supplemental Material [file supp_gr.276992.122_Supplemental_Information.pdf]

Supplementary Information

Supplemental information for the article: Chromatin structure influences rate and spectrum of spontaneous mutations in *Neurospora crassa*. By Mariana Villalba de la Peña, Pauliina A. M. Summanen, Martta Liukkonen, and Ilkka Kronholm.

Contents

Supplementary methods 2

- Mutation accumulation experiment 2
- Estimating the number of mitoses in the MA experiment 3
- DNA extraction 7
- Read mapping and genotyping 8
- Genotyping structural variants 9
- Genotyping copy number variants 11
- Chromatin modifications 12
- Analysis of relative mutation rate for different classes 12
- Mutation rate variation across the genome 13
- Effects of local sequence context 15

Supplementary results 16

- Accuracy of mutation calling 16
- Simulating variation in mutation rate 20
- Robustness of relationship between θ and predicted mutation rate 21
- Re-analysis of data from Wang et al. 2020 23

Supplementary Figures 31

Supplementary Tables 53

Supplementary methods

Mutation accumulation experiment

We started the MA experiment with two different strains: 2489 *mat A* and 2489 *mat a*. We have previously generated these strains by backcrossing mating type *mat a* from strain 4200 into 2489 nine times (Kronholm et al., 2020). The strains differ in their mating types, but should share over 98% of the rest of their genetic background. Their Fungal Genetics Stock Center ID's are: B 26708 and B 26709. We used 20 lines for both these strains, giving 40 MA lines in total. We used two different mating types to later have the possibility to perform crosses between the MA lines. However, for this study the mating types of the lines do not matter as all propagation was asexual.

Common protocols for culturing *N. crassa* were followed, and sorbose plates were used to induce colonial morphology on plates (Davis and de Serres, 1970). The experiment was started by picking a single colony from a sorbose plate for both ancestors and transferring that colony into a 75×12 mm test tube with flat surface of 1 mL of Vogel's Medium (VM) with 1.5% agar and 1.5% sucrose (Metzenberg, 2003). Tubes were incubated at 25 °C for 3 days to allow conidia (asexual spores) to develop. Then we picked small amount of conidia with a loop into a tube with 1.4 mL of 0.01% Tween-80, we then pipetted 1 µL of this conidial suspension into a 50 µL water droplet on a sorbose plate and spread it. We incubated the plates at room temperature for 2 days and picked single colonies to establish the MA lines. The MA lines were transferred the same way, so that a single colony was always picked randomly from a sorbose plate to propagate the MA line (Figure 1B). We tested that 2 days of incubation was enough time for all colonies to appear on plates. Combining the time of 2 days on plates and 3 days in a tube, a single transfer took 5 days. We propagated the MA lines for 40 transfers, the ancestors and the MA lines were stored frozen in suspended animation until sequencing.

Estimating the number of mitoses in the MA experiment

To estimate the mutation rate per mitosis, we needed to estimate how many mitoses happened in the MA lines during the experiment. To estimate the number of mitoses that happened during one transfer, we needed to obtain data about the number of nuclei present in each phase of a transfer: in a colony on a sorbose plate, in the mycelium in a test tube, and the conidia produced in the test tube. To estimate the density of nuclei per µm^2 of hyphae we used the strain *mat A his-3*$^+$*::Pccg-1-hH1*$^+$*-sgfp*$^+$ (FGSC# 9518) which expressed a green fluorescent protein that had been fused into histone H1 (Freitag et al., 2004). We grew the strain on plates with either normal VM medium or sorbose medium, cut out a piece of the agar, and mounted it on a glass coverslip using the inverted agar block method (Lichius and Zeilinger, 2019). We used Congo Red to stain cell walls: a 20 µL droplet with 2 µM Congo Red was pipetted to a glass coverslip and an agar block with the side carrying the mycelium was placed face down in the droplet.

Samples were imaged with a Nikon A1R confocal microscope, GFP was excited with a 488 nm laser and detected with a 515/30 emission filter, Congo Red was excited with a 561 nm laser and detected with a 595/50 emission filter. Plan apochromat air objectives 20x (numerical aperture 0.75) and 40x (numerical aperture 0.95) were used. Laser power was set as low as possible to avoid saturated pixels. We imaged vertical stacks of the mycelium, and used imageJ2 (Rueden et al., 2017) to measure the area covered by hyphae in sections of the image, and counted the number of nuclei in these areas (Figure 1C).

We then estimated the number of nuclei in the different phases of a transfer, and calculated the number of mitoses that the MA lines went through. The number of nuclei can only increase when the old nuclei divide. If we know the number of initial nuclei and the number of nuclei at time t, we can calculate the number of mitoses, m, that separate these time points from the equation

$$m = \log_2 \left(\frac{n_t}{n_i} \right) \tag{S1}$$

where n_i is the initial number of nuclei, and n_t the number of nuclei at time t. Thus, in order to

estimate number of mitoses that happened in the MA lines during one transfer, we need to count how many nuclei were present in the colonies on sorbose plates that were picked and transferred to slants, and how many nuclei were in the mycelium that formed in the test tube, and finally how many nuclei were in the conidia that formed in the test tube (Figure 1B). This allows us to calculate how many mitoses happened during one transfer of the MA experiment, from one spore to a spore. There are multiple sources of uncertainty in these calculations, so we used a Bayesian framework to do the calculations using posterior distributions of the estimates to incorporate all sources of uncertainty in the final estimate.

Nuclei were counted from the microscope images using Fiji2 version 2.0.0-rc-54/1.51g (Schindelin et al., 2012). Short sections of mycelium were surrounded with the rectangular selection tool and the area inside was measured. All nuclei with more than 50% of their diameter inside the selection were counted manually. Multiple sections were counted from each image, with no overlap. In some fainter images, the contrast was enhanced with the enhance contrast tool, with the default value 0.3% saturated pixels and no histogram equalization. To estimate the number of nuclei in a given area of hyphae, we used the counts of nuclei and the hyphal areas measured from the microscope images to obtain the number of nuclei per $\mu\mathrm{m}^2$. We had images for both VM and sorbose plates, in total we collected 519 measurements. To estimate average density of nuclei for VM and sorbose we used a model where we allowed standard deviations to differ for VM and sorbose media:

$$
\begin{aligned}
y_i &\sim \mathrm{N}(\mu_i, \sigma_i) \qquad \text{(S2)}\\
\mu_i &= \rho + \beta_s x_i \\
\log(\sigma_i) &= \alpha_\sigma + \beta_\sigma x_i \\
\rho, \beta_s &\sim \mathrm{N}(0, 0.1) \\
\alpha_\sigma, \beta_\sigma &\sim \mathrm{hT}(3, 0, 10)
\end{aligned}
$$

where y_i is the ith density measurement, ρ is the intercept, β_s is the effect of sorbose medium, x_i an indicator variable for sorbose, α_σ is the intercept for standard deviation, and β_σ is the effect of sorbose medium on standard deviation. The average density of nuclei in VM medium is ρ and the density of nuclei in sorbose is obtained as $\rho_s = \rho + \beta_s$.

To estimate the average size of colonies on sorbose plates, we plated conidia on sorbose plates as in the MA experiment and photographed the plates. Millimeter paper was used as a scale. Colony area was measured from these images with ImageJ2 version 2.0.0-rc-43/1.50e. The pixels per millimeter calibration value was set by measuring the number of pixels per 1 mm of millimeter paper. The images were enhanced with the sharpen tool to make the colony outlines more distinct. The colony area was measured using the elliptical selection tool. We used 10 different genotypes from different MA lines and timepoints in this experiment, including the 2 ancestors. We collected a dataset with 482 area measurements. To estimate the average colony size, we fitted a multilevel model

$$
\begin{aligned}
y_i &\sim \mathrm{N}(\mu_i, \sigma) \\
\mu_i &= \alpha_{g[i]} \\
\alpha_g &\sim \mathrm{N}(\bar{\alpha}, \sigma_g) \\
\bar{\alpha} &\sim \mathrm{N}(0, 3) \\
\sigma, \sigma_g &\sim \mathrm{hT}(3, 0, 10)
\end{aligned} \tag{S3}
$$

where y_i is the ith area measurement, $\bar{\alpha}$ is the overall mean, α_j is the mean for jth genotype, σ_g is the genotype standard deviation, and σ is the error standard deviation. Standard deviations had a weakly informative prior, which was the half-location scale version of Student's t-distribution, where 3 is the degrees of freedom, 0 is the location, and 10 is the scale parameter. We estimated

the number of nuclei in a sorbose plate colony, n_s, as

$$n_s = \bar{\alpha} \times 10^6 \times \rho_s \tag{S4}$$

the average colony size is multiplied by 10^6 to transform the unit from mm^2 to $\mu\mathrm{m}^2$.

Once the sorbose colony is transferred to the test tube, the mycelium will cover the surface of the growth media. We estimated the number of nuclei present in the mycelium, n_v by multiplying the surface of the media in the test tube with the density of nuclei in the hyphae in VM medium:

$$n_v = \pi(d/2)^2 \times 10^6 \times \rho \tag{S5}$$

where d is the diameter of the test tubes (in mm) used in the experiment, area is multiplied by 10^6 to transform the unit to $\mu\mathrm{m}^2$.

To estimate the number of conidia produced by the mycelium in the test tube, we counted conidia by suspending them in 1 mL of 0.01% Tween-80, making a 10000-fold dilution of the suspension, and plating 10 µL of the dilution on sorbose plates. We counted the colonies that were formed, and estimated the original number of conidia produced. We used 10 different genotypes, including the ancestors from the MA experiment to estimate produced conidia. We collected 71 measurements, the model was

$$\begin{aligned} y_i &\sim \mathrm{N}(\mu_i, \sigma) \qquad \text{(S6)} \\ \mu_i &= \nu_{g[i]} \\ \nu_g &\sim \mathrm{N}(\bar{\nu}, \sigma_g) \\ \bar{\nu} &\sim \mathrm{hT}(3, 40, 21) \\ \sigma, \sigma_g &\sim \mathrm{hT}(3, 0, 21) \end{aligned}$$

where y_i is the ith conidial number measurement, $\bar{\nu}$ is the overall mean, α_j is the mean for jth

genotype, σ_g is the genotype standard deviation, and σ is the error standard deviation. Priors followed Student's t-distribution. The number of nuclei contained by the conidia, n_c was estimated as

$$n_c = 2\bar{\nu} \tag{S7}$$

since the mode of nuclei in conidia of *N. crassa* is two.

The number of mitotic divisions separating two time points can be calculated from equation S1. First, we need to calculate the number of divisions that happened when a single spore grows to a colony on sorbose plate, then the number of divisions when the colony grows to a lawn of mycelium in the test tube, and finally the number of divisions it takes to form the final number of conidia. Thus, using the posterior distributions of numbers of nuclei in the different phases of the transfer and equation S1, we can calculate the number of mitoses that happen during a transfer, m, as:

$$m = \log_2\left(\frac{n_s}{2}\right) + \log_2\left(\frac{n_s + n_v}{n_s}\right) + \log_2\left(\frac{n_s + n_v + n_c}{n_s + n_v}\right)$$

which simplifies to

$$m = \log_2\left(n_s + n_v + n_c\right) - 1 \tag{S8}$$

this estimate of the number of mitoses incorporates all sources of measurement error since posterior distributions are used in every step of the calculations.

DNA extraction

To get high quality DNA for sequencing, the natural strains, MA lines, and the ancestors were grown in 5 mL of liquid VM for two days at 25 °C with shaking. We harvested the mycelium and freeze dried it over night in a lyophilizer. Dried mycelium was then ground with a glass bead in Qiagen Tissue Lyzer for two times 20 s with frequency of 25 s^{-1}. Then 500 µL of extraction buffer was added (10 mM Tris pH 8, 0.1 M EDTA, 150 mM NaCl, and 2% SDS), and the powdered tissue dissolved by shaking. Then samples were extracted with 750 µL of 25:24:1 Phenol:Chloroform:Isoamylalcohol and keeping the aqueous phase. We added 2 µL of RNAse A (10

mg/mL) and 50 U of RNAse I to each sample and incubated them for 1 h at 37 °C. Samples were then extracted with 750 µL of chloroform, 1 mL of 100% ethanol was added, and DNA was precipitated for 1 h at −20 °C. Then DNA was pelleted with centrifugation at 4 °C, ethanol aspirated, pellet washed with 70% ethanol, and air dried. We then added 77.5 µL of TE-buffer to elute the samples and incubated at 37 °C to help dissolve the pellets. We observed that occasional small DNA fragments would remain in the samples and to remove these we did a polyethyleneglycol precipitation: we added 12.5 µL of 4 M NaCl, mixed and added 12 µL 50% PEG (P3350), mixed and precipitated DNA over night at 4 °C. DNA was then pelleted with centrifugation and the supernatant aspirated, the pellet was washed twice with 70% ethanol, and aspirated. Pellets were eluted to 55 µL of TE-buffer as above. DNA concentrations were measured with the Qubit Broad Range Kit, and DNA quality was checked by running 2 µL of the sample on an 0.8% agarose gel.

Read mapping and genotyping

To be able to map reads to the mating type locus in the *mat a* strains, we included the mating type *a* region, as well as the mitochondrial genome, as additional contigs. Reads were mapped using BWA-MEM version 0.7.12-r1039 with default parameters (Li, 2013). Alignment files were sorted and indexed with samtools and read groups were added with picardtools. See table S1 for alignment metrics.

We used the GATK version 4.2.0.0 (McKenna et al., 2010) pipeline to call single nucleotide mutations (SNMs) and small indels. First, we ran Haplotypecaller for each sample individually to make a g.vcf file. Haplotypecaller was run with otherwise default parameters, emitting all sites, and in diploid mode. We then consolidated all of the samples together into a database using the GenomicsBDImport function in GATK. Samples were then jointly genotyped with the GenotypeGVCFs function to produce a vcf file with all samples.

We used wormtable version 0.1.5 (Kelleher et al., 2013) to convert the vcf file into an indexed database and then a custom Python script to filter for high quality sites. For a site to be included as a candidate mutation, first we required the genotypes of the ancestor and the MA line to differ

for that site. Second, the site had to have five or more reads from both the ancestor and the sample. Third, the site had to have genotype quality greater or equal to 30 for both the ancestor and the sample, and finally sites that were called heterozygous in either the ancestor or the sample were excluded. There was also a filter that a site could not be called as a mutation if all of the MA lines had the same genotype. Sites were considered as invariant if their reference genotype quality was greater or equal to 30.

To produce the final dataset of curated mutations, we checked all candidate mutations manually by inspecting the alignments from BWA and or Haplotypecaller in IGV (Thorvaldsdóttir et al., 2013). Based on our manual inspection our filtering criteria were stringent enough, for our high coverage haploid genomes, to remove mapping errors and leave only real mutations, as only very few candidate mutations had to be rejected based on manual inspection and most mutations were unambiguous.

For genotyping SNPs in the strains sampled from natural populations, the above pipeline was used to call the genotypes. Other variants than SNPs were excluded. For a site to be included, it had to be polymorphic in the sample, with a mean read depth five or greater, genotype quality 30 or greater, and mapping quality 40 or greater across all samples. Then these same criteria were applied for each individual sample, and if a sample failed to meet the quality filters, its genotype was recorded as missing data. Heterozygous sites were excluded. Sites were also excluded if $> 90\%$ of samples had missing data. Sites were called as monomorphic if the mean reference genotype quality was 30 or greater and read depth 5 of greater across all samples. Then these same criteria were applied to individual samples, genotypes were recorded as missing data if a sample did not pass the filters.

Genotyping structural variants

There are several algorithms available to detect structural variants (SVs) from short-read sequencing data. However, because this kind of data is prone to base calling and alignment errors, none of the available computational algorithms can accurately and sensitively detect all types and sizes

of SVs (Kosugi et al., 2019). To overcome this limitation it is common to use several algorithms and merge their outputs to increase sensitivity and precision. First, we assessed the performance of four different SVs algorithms (DELLY, Lumpy, PINDEL and SVaba) using simulated data.

We evaluated the performance of different SV callers on simulated data created using SURVIVOR version 1.0.7 (Jeffares et al., 2017). SURVIVOR simulates SVs by first modifying a fasta reference file by randomly altering locations according to given parameters of length and number of different SVs types (insertions, deletions, duplications, inversions and translocations). Reads are simulated based on the modified fasta and SVs are detected using the preferred SV caller. Finally SURVIVOR compares the SVs detected against the known simulated SVs, based on this FDR and sensitivity can be calculated.

We simulated four sets with 18, 40, 50 and 120 structural variants with a mutation rate of 0.001 on the reference genome (assembly NC12). The number of each type of SV simulated in each set is presented in the table S6. In set number four we simulated 20 complex SVs in which inversions and deletions occur in the same location. For duplications the min and max length parameter was set to 100-1000 bp, for INDELs 20-500 bp, for translocations 1000-3000 bp and for inversions 600-800 bp.

The SV length distribution across our four simulated sets were very similar (Figure S23), and the distribution coincides with ones reported in the literature, which indicates that short SVs are more common than large ones (Jeffares et al., 2017). The number of reads, the error rate and the coverage of the simulated data represent our sequenced reads. The inflated number of SV per genome is for testing purposes.

Based on the modified fasta we created 150 bp pair end reads with an error rate of 0.003% and a mean coverage of 30X using DWGSIM version 0.1.11 (Homer, 2021). Simulated reads were then aligned to the reference genome using BWA-MEM (Li, 2013) with default parameters, and SVs were called using DELLY version 0.8.7, LUMPY version 0.2.13, PINDEL version 0.2.5b9 and SVaba version 1.1.0 (Rausch et al., 2012; Layer et al., 2014; Ye et al., 2009; Wala et al., 2018). Finally, we used SURVIVOR to evaluate the performance of each SV caller. The SV calls

were considered correct if the simulated and detected SVs were 1) of the same type 2) on same chromosome and 3) both start and stop locations were within 50 bp. The callers that performed the best were DELLY and LUMPY as they showed high sensitivity score and low false discovery rate (FDR) score (Table S6), and they were selected to call SVs on the MA lines.

For calling SVs in the MA lines we first aligned the reads to the reference genome using BWA-MEM, excluded duplicated reads with SAMBLASTER version 0.1.26 (Faust and Hall, 2014), and extracted the discordant paired-end and split-read alignments using SAMTOOLS version 1.9 (Danecek et al., 2021). DELLY was used as indicated in the recommended workflow (Rausch et al., 2012). For LUMPY the read and insert lengths were extracted from alignment files using SAMTOOLS and the SVs were genotyped using SVTyper version 0.7.1 (Chiang et al., 2015). To filter out SVs that were present in the ancestor we used SnpSift version 5.0e (Cingolani et al., 2012). We removed those calls with a genotype quality score lower than 30 and read depth below 10. The analysis with both callers were carried out in somatic-germline mode, considering MA line as somatic and the ancestor as the germline. The signature of a translocation are reads with discordant mate pairs, where both mates are consistantly mapped to other chromosomes for example. Translocation length was determined from the break points of these discordant reads. All of the SVs detected by each caller were manually verified by inspecting the alignment files in IGV.

Genotyping copy number variants

To evaluate the performance of copy number variant (CNV) detection algorithms, we simulated 32 CNVs using SECNVs version 2.7.1 (Xing et al., 2020), then simulated 150 bp paired end reads with an error rate of 0.03% and a mean coverage of 30X using DWGSIM. We scanned for copy number variants (CNVs) using two detection programs, CNVnator version 0.4.1 (Abyzov et al., 2011) and CNV-seq version 0.2-7 (Xie and Tammi, 2009). CNV-seq was used with default parameters while CNVnator was used with two different bin sizes, 75 and 1670. Bins of 75 bp allowed the detection of small events, while bins of 1670 bp, which is the average gene length of *N. crassa* (Galagan et al., 2003), allowed the detection larger-scale events. Both callers togetherperformed better that

any of the callers individually by showing the lowest FDR rate score of 0.482, and good sensitivity score of 0.906 (Table S7).

For genotyping CNVs in the MA lines we excluded MA line sites if the start or stop location of these where within 500 bp of any site detected in the ancestor. Also, we only retained the sites that were detected by both callers CNVnator and CNVseq (if 1000 bp or less overlapped at the start or end location). The remaining sites were manually verified by inspecting the alignment file in IGV. However, we did not find any evidence of copy number changes in the MA lines.

Chromatin modifications

To determine regions of the genome where chromatin modifications occur, ChIP-seq reads for H3K9me3, H3K27me3, and H3K36me2 were aligned to the reference genome using BWA-MEM, and duplicate reads were removed by Picard tools. Domains of chromatin modifications were identified using RSEG 0.4.9 (Song and Smith, 2011). Data for centromeric regions were obtained from Smith et al. (2011) and coordinate corrections for NC12 from Wang et al. (2020). The centromeric regions were defined based on the presence of centromeric histone 3 variant: CENPA. Smith et al. (2011) collected ChIP-seq data against CENPA and other centromeric proteins. Centromeric sequences in *N. crassa* are composed of AT-rich sequences of degraded transposable elements. However, the repeat arrays are heterogenous due to action of RIP, making almost all sequence sufficiently unique to be able to map short reads to the genome (Smith et al., 2011).

Furthermore, we used the data of the duplicated regions that were defined by Wang et al. (2020). Wang et al. (2020) identified duplicated regions using BLAST, with the criteria of at least 100 bp alignment length and at least 65% sequence identity.

Analysis of relative mutation rate for different classes

For cases where the relative mutation rates were computed for different classes of mutations the model was:

$$
\begin{aligned}
y_i &\sim \text{Poisson}(\lambda_i) \\
\log(\lambda_i) &= \log \tau_j + \alpha_{[j]} \\
\alpha_{[j]} &\sim \text{N}(0, 10)
\end{aligned}
\tag{S9}
$$

where τ_j is an offset term for class j that allows taking into account differences in the abundance of certain classes (McElreath, 2015), such as higher frequency of A's and T's than G's and C's in the genome. Priors for different predictors remained the same as in equation 1. Furthermore, if we calculate the expected number of mutations for different classes under the assumption that all mutations in all classes are equally likely, as $\tau_j = f_j n$, where f_j is the frequency of class j and n is the total number of observed mutations, and use τ_j, the expected number of mutations, as the offset parameter, then $\exp(\alpha_{[j]})$ yields the relative mutation rate of class j. Since all estimates for different classes come from the same model, they are simultaneous comparisons in the statistical sense.

Mutation rate variation across the genome

To model the effects of epigenetic domains and GC-content on mutation rate we used the following model:

$$
\begin{aligned}
y_i &\sim \text{Poisson}(\lambda_i) \\
\log(\lambda_i) &= \log \tau_i + \alpha + \beta_{GC} x_i + \beta_{K9} d_i + \beta_{K27} g_i + \beta_C c_i + \beta_I x_i d_i \\
\alpha, \beta &\sim \text{N}(0, 10)
\end{aligned}
\tag{S10}
$$

where y_i is the number of mutations a class of i intervals contained, τ_i is the number of class i intervals in total, x_i the GC-content of those intervals, d_i indicates presence or absence of H3K9me3, g_i indicates presence or absence of H3K27me3, and c_i indicates presence or absence of centromeric region. β coefficients are the corresponding effects and α is the intercept.

Model selection was a combination of biological and statistical reasoning, and we tested models representing plausible biological hypotheses. For instance, we had a clear biological reason to expect that GC-content influences mutation rate, and we saw a large improvement in model predictions when GC-content was included in the model. Therefore we did not further test models without GC-content and with different combinations of other terms. Furthermore, the only biologically realistic interactions are those involving GC-content and one of the domains. There are no regions where H3K27me3 and centromeric regions overlap, or regions where H3K9me3 and centromeric regions do not overlap, hence statistical interactions between domains are not possible in our data. Tested models are shown in Table S2, model comparisons were done using the widely applicable information criterion (WAIC) (McElreath, 2015; Vehtari et al., 2017).

When we assessed how well did the mutation model predict the natural genetic variation we used the predicted mutation rates from model S10 as a response and θ_W calculated from a population sample of strains as a predictor in a simple regression model. Bayesian version of R^2 (Gelman et al., 2019) was used to assess the model fit.

We could not asses the effect of duplicated regions defined by Wang et al. (2020) independently of H3K9me3 regions. Nearly all duplicated regions overlapped with H3K9me3 regions (Figure 2). Those regions that were marked as duplicates, but which did not overlap with H3K9me3 or H3K27me3, contained mainly mutations in microsatellite repeats. Only 10 point mutations were observed in these regions, which was not enough to obtain reasonable estimates of independent effect of duplicated regions on mutation rate. Of those 10 point mutations, 3 were C:G $\rightarrow$ T:A transitions. As C $\rightarrow$ T transitions were not over-represented, action of RIP is unlikely to be responsible for these mutations, which is expected as RIP is active only during meiosis.

Effects of local sequence context

To analyze effects of local base composition on the mutation rate, we estimated the effects of the trinucleotides from a model that included the effects of the epigenetic domains. First, we extracted the adjacent basepairs for every point mutation. There are 64 different trinucleotides, but as we cannot know in which strand the mutation originally occurred we grouped the trinucleotides into 32 different classes based on sequence complementarity. For example, trinucleotides ATA and TAT are complementary and were grouped. Then we counted how many times a given trinucleotide occurs in the genome in all three reading frames. Relative mutation rate was analyzed using the following model:

$$
\begin{aligned}
y_i &\sim \text{Poisson}(\lambda_i) \qquad \text{(S11)}\\
\log(\lambda_i) &= \log \tau_t + \beta_t x_{[t]} + \beta_{K9} d_t + \beta_{K27} g_t + \beta_C c_t\\
\beta &\sim \text{N}(0, 10)
\end{aligned}
$$

We compared different linear models (Table S4) with the same reasoning as above. We did not include an intercept in this model, as we wanted to obtain estimates for all trinucleotide classes, and not set one class as the intercept against which the others are compared. This does not alter any biological conclusions.

We further investigated how the flanking base pairs influenced the relative mutation rates of the trinucleotides. We extracted estimates of the relative mutation rates for the trinucleotides from model S11, and used these as a response in a model where we predicted relative mutation rates with the identities of the flanking base pairs and the mutating base. Since our estimates of the relative mutation rates contain uncertainty, we included the estimated error of the relative mutation rates in the model. The model was:

$$
\begin{aligned}
y_{obs,i} &\sim \mathrm{N}(y_{est,i}, y_{sd,i}) \\
y_{est,i} &\sim \mathrm{N}(\mu_i, \sigma) \\
\mu_i &= \alpha + \beta_b x_i + \beta_5 z_i + \beta_3 g_i + \beta_{I5} x_i z_i + \beta_{I3} x_i g_i \\
\alpha, \beta &\sim \mathrm{N}(0, 10) \\
\sigma &\sim \mathrm{hT}(3, 0, 10)
\end{aligned}
\tag{S12}
$$

where $y_{obs,i}$ is the median of ith observed relative mutation rate, $y_{sd,i}$ is the observed standard deviation of the ith relative mutation rate, $y_{est,i}$ is the ith estimated relative mutation rate, α is the intercept, β_b is the effect of C:G relative to A:T for the mutating base, β_5 is the effect of C:G relative to A:T for the 5' flanking base pair, β_3 is the effect of C:G relative to A:T for the 3' flanking base pair, β_{I5} is the interaction effect of 5' CG when the mutating base is C:G, and β_{I3} is the interaction effect of 3' C:G when the mutating base pair is C:G. x_i, z_i, and g_i are indicators whether the basepair is C:G. We used the half location-scale version of Student's t-distribution as a prior for the standard deviation with 3 degree's of freedom, location 0, and scale 10.

Supplementary results

Accuracy of mutation calling

Estimating mutation rates and particularly estimating differences in the mutation rate in different parts of the genome requires accurate mutation calls. As some regions of the genome, such as centromeric regions, may contain repetitive sequences it is important to verify that the mutations are called accurately in all regions of the genome, and that no region has an excess of false positive mutations. First, we examined sequencing coverage throughout the genome, GC-content does have an effect on sequencing coverage as regions of low GC can be preferentially amplified during library construction, and we observed slight elevation on normalized coverage around 35% GC (Figure

S13). However, overall we observed that sequencing coverage was rather uniform across regions of different GC-content (Figure S13). Centromeric regions and regions marked by H3K9me3 have low GC-content, and while we did observe that coverage went down in regions of $< 15\%$ GC, those regions constitute a very small fraction of the genome. Next, we explored the accuracy of our mutation calls after the mutations had been called by our pipeline and manually inspected in IGV. We observed that overwhelming majority of mutations had the highest possible genotype quality score determined by the GATK pipeline (Figure S1). Median genotype quality for mutations was the highest possible value of 99, and only 8.6% of mutations had genotype quality less than 80 and only 1.9% less than 50. Distribution of quality scores was similar in different regions of the genome (Figure S1). While there was slightly more mutations that had lower quality scores than 99 in regions marked by H3K9me3 and in centromeric regions than in euchromatic regions (Figure S1), overwhelming majority of mutations in those regions have the highest genotype quality score of 99.

If most of the mutations had genotype quality scores of 99, then what kind of confidence we have in those mutation calls? We illustrate genotype quality scores with alignments viewed in IGV that show mutations in different regions of the genome and different genotype quality scores (Figure S14, S15, S16, S17, S18, S19, S20, S21, S22). When mutations had genotype quality score of 99 they were unambiguous (Figure S14, S17, S20). When genotype qualities were around 70 mutations could still be distinguished from unambiguously, even if few reads did not support the mutation or the mutations were in repetitive regions. When mutation genotype qualities were around 45 this was usually a sign that the region had lower mapping quality due to repeats or duplications (Figure S16, S19, S22). Despite of this, even in these regions, real mutations could be distinguished from mapping errors by looking at which reads supported the mutation and which did not (Figure S19, S22).

We have also provided screenshots of the alignments showing mutations viewed in IGV for a random sample of mutations. We selected mutations randomly, by first splitting the mutations into three genomic domains: H3K9me3, centromeric, and euchromatic, then drew a random sample of

30 from each pool, for a total of 90 mutations (see supplementary file S2). Information about the sampled mutations can be found in supplementary file S1.

The reason we chose first to do Sanger-verification for the mutations with the lowest genotype qualities was because for mutations with genotype quality of 99, there was no doubt that these were real mutations. We verified 23 base pair changes, of which 12 were in complex mutations and 11 as single nucleotide mutations. Of the 11 SNMs 5 were in regions marked by H3K9 (excluding centromes), 3 in centromeric regions, and 3 in euchromatin. Of the 12 base pair changes in complex mutations, 3 mutations were in H3K9 regions (5 base changes in total), 1 mutation in centromeric region (2 base pair changes), and 3 mutations in euchromatin (5 base pair changes). In the second verification set we sequenced 15 randomly sampled mutations from each genomic region (euchromatin, H3K9me3, and centromeric). One mutation located in centromeric region failed to amplify by PCR, the remaining 44 mutations were all confirmed. In summary, we confirmed point mutations by Sanger sequencing in centromeric, H3K9me3, and euchromatic regions. We confirmed all point mutations where PCR-amplification and Sanger sequencing were successful, so we never detected a false positive point mutation.

Why were the genotype qualities of the mutations so good in our experiment? There are several factors in this study that contributed excellent genotype calls. First, the ancestors for the MA lines were derived from line 2489 (synonym OR74a), which was the strain used for the original genome project (Galagan et al., 2003). Therefore, the reference genome used for read mapping corresponds to the genome of the MA line ancestors. This is seen in alignment metrics as 98% reads are mapped to the genome in the ancestors and MA lines (Table S1). As such, there are likely not many reads that would erroneously map to an incorrect location because their true source of origin was missing from the reference genome. Second, as explained in the introduction, repetitive sequences tend to diverge from each other in *N. crassa* due to the action of RIP. RIP does not induce the exact same mutations to the duplications, so over time duplicated arrays, such as those often found in centromeric regions, tend to diverge from one another, to the extent that short reads can be mapped to the genome in regions where it is not often possible to the same extent in other species (Smith

et al., 2011). Third, the small genome of *N. crassa* made it possible to sequence the samples to a high depth, on average over 50x in many samples (Table S1). This allowed us to discriminate between true mutations and mapping errors. With this kind of sequencing depth, sequencing errors are simply not an issue anymore and they have no impact on calling the mutations, e.g. Figure S18 shows a mutation in repetitive region that as a consequence has higher frequency of sequencing errors, but with so many reads identifying the real mutation is not a problem. Finally, *N. crassa* is haploid. Combined with high sequencing depth, this makes identifying mutations easy. The only important errors are read mapping errors that may cause some sites to appear as heterozygous. But as heterozygous sites are not expected to occur in our experiment we can filter out sites called as heterozygous. We did inspect heterozygous sites manually, as it is possible that some mutations could have been present in a heterokaryotic state (nuclei with different genotypes in the same mycelium). However, we did not find any evidence of true mutations in heterokaryotic state. Whenever sites appeared as heterozygous, multiple sites were found close together (Figure S19), indicating that read mapping errors were the more likely explanation. Because of these factors, our study differs substantially from studies that need to call heterozygous sites from data with low sequencing depth and the problem of calling genotypes correctly is of different nature.

In summary, overwhelming majority of mutations that our pipeline detected had the highest possible genotype quality of 99, and this was true in regions of the genome with potentially more repetitive and duplicated regions like in centromeric regions and regions marked by H3K9 methylation. Those mutations that had genotype quality of 99 were unambiguously real mutations. Thus, even if we would filter out every mutation with genotype quality less than 99, we would still detect the observed pattern that mutation rate was higher in regions marked by H3K9 trimethylation and in centromeric regions. Differential mutation calling in different regions of the genome cannot explain the observed results.

Simulating variation in mutation rate

Despite our very high genotype qualities, we attempted to further understand could repetitive sequences or other sequence features of heterochromatin in the *N. crassa* genome hinder our ability to correctly estimate differences in mutation rates in different regions of the genome. We simulated data under two different scenarios. First, we simulated a scenario where mutation rate was set to be higher in H3K9me3 domains, with a rate of 2×10^{-5} mutations per site, compared to the rest of the genome, with a rate of 3×10^{-6} mutations per site. In the second scenario, we simulated a uniform mutation rate across the genome, with a rate of 2×10^{-6} mutations per site. We simulated mutations to the *N. crassa* genome using the program Mutation-Simulator (Kühl et al., 2021). We simulated 40 different MA lines for each scenario with a transition / transversion rate of 1.08. We then generated simulated reads from these simulated genomes, using DWGSM (Homer, 2021), with 30X sequencing depth and read length of 150 bp. We tried to imitate the conditions of our real sequenced data, so we set the standard deviation of the base quality scores to two and the per base sequencing error rate to 0.003. The ancestor of the MA lines was simulated by generating reads from the reference genome of *N. crassa*. To call the simulated mutations from the simulated reads, we ran the same pipeline as we used for the experimental data. Thus, we had two simulated scenarios, and for each scenario we had information about the true number of mutations that happened in the simulation, and number of mutations we called with our pipeline from the simulated read data.

In the scenario with the higher mutation rate in H3K9me3 regions, we ended up with a total of 1759 mutations, of which 719 were in H3K9me3 domains, 990 in euchromatin, and 50 in unspecific domains. With our pipeline we detected a total of 1705 mutations, of which 692 were in H3K9me3 domains, 964 in euchromatin and 49 in unspecific domains. All of the called mutations were true positives. However, we failed to call 54 true mutations, that is, these were false negatives. In a similar manner, in the scenario with the uniform mutation rate, we ended up with a total of 3078 mutations, of which 562 were in H3K9me3 domains, 2245 in euchromatin, and 271 in unspecific domains. Our pipeline detected 2978 mutations in total, of which 535 were in H3K9 domains, 2177 in euchromatin, and 266 in unspecific domains. Again, there were no false positive calls. We failed

to detect 100 mutations in this set. In general, the number of false negatives was higher in H3K9me3 regions, with proportion of false negatives 3.75% and 4.80% in H3K9me3 regions, and 2.62% and 3.02% in euchromatin in for the different and uniform mutation rate scenarios respectively.

We found that the estimated mutation rate was higher in H3K9me3 regions in the scenario where the true mutation rate was higher in H3K9me3 (Figure S2), the mutation rate ratio of H3K9me3 / euchromatin was 3.39 [3.06, 3.72]. This mutation rate ratio was not statistically different from the one calculated from the true simulated mutations: the difference was 0.28 [-0.15, 0.74], which includes zero in the interval estimate. Furthermore, when we simulated a uniform mutation rate across the genome, we found no difference among called and true datasets (Figure S2). The mutation rate ratio of H3K9me3 / euchromatin was 1.15 [1.05, 1.27], there was no statistical difference in the rate ratios between called and true simulated mutations: difference was 0.08 [-0.05, 0.23], which includes zero in the interval estimate.

With this simulation data we show that our pipeline can confidently detect a difference in mutation rates in different regions of the genome. This shows that sequence features of the H3K9me3 regions, such as repetitive sequences, do not interfere with mutation calling in a manner that would lead to gross biases in mutation rate estimates in the different domains. While simulated read data cannot capture all of the properties of real data, because of sequences missing from the reference or assembly errors, it does give us confidence that we will be able to detect a real difference in mutation rates. Moreover, since we did not observe any false positive mutations, we are confident that mutation calling cannot generate spurious results in our case. We did observe slightly higher proportions of false negative mutations in H3K9me3 regions. However, if this bias is true for real data, this would make our estimate of the elevated mutation rate in H3K9me3 regions more conservative.

Robustness of relationship between θ_W and predicted mutation rate

We wanted to evaluate the robustness of the observed relationship between θ and the predicted mutation rate. One potential issue is that there are windows in the genome, especially for small window sizes, where the observed θ is zero. Since zero is the minimum value that θ can obtain, and

there is a clumping of $\theta = 0$ observations in the data, this violates the assumption that response is gaussian and could lead to biased estimates. However, since there so many data points, the model may be robust to observations where $\theta = 0$. First, we tested the effect of window size, calculating θ over longer windows reduced the number of windows where $\theta = 0$. Increasing window size slightly improves the amount of variation explained by the model (Figure S5). Thus, results are robust the to different window sizes.

Then we tested whether the results were robust to different models. Data that can take zero or positive values, but is clumped at zero, can be modeled in different ways. One possibility is Tobit regression. Tobit regression is a type of censored regression, where observations are assumed to have an underlying gaussian distribution, but appear as zeros if $y_i \leq 0$ (Min and Agresti, 2002). We used a conventional Tobit regression and robust Tobit regression, for both cases the results were very similar to an ordinary regression model (Figure S6). Then, we tested a log-normal hurdle model. In this model the response distribution is a mixture of two processes, one models the probability that the observation is larger then zero, and the other is a log-normal gaussian model (Min and Agresti, 2002). For the hurdle model, we also observed that that the relationship between θ and predicted mutation rate was positive (Figure S6). Therefore, our results are robust to the clumping at zero phenomenon.

Next, we tested whether the action of RIP could explain the relationship between θ and predicted mutation rate. If level of genetic diversity is very high in H3K9me3 regions due to C $\rightarrow$ T transitions induced by RIP, we want to make sure that this phenomenon does not solely cause the relationship between θ and predicted mutation rate. We cannot determine the exact contribution of RIP to genetic diversity, because we do not know the ancestral states of the SNPs and therefore cannot distinguish between C:G $\rightarrow$ T:A and A:T $\rightarrow$ G:C transitions. Furthermore, we would need to know the population recombination rate to estimate the number of meiotic divisions for every mitosis and thus the frequency of RIP. Therefore, we looked at the relationship between θ and predicted mutation rate within each of the genomic domains, and observed a positive relationship between θ and predicted mutation rate within each of the domains. Although, the effect was weak within cen-

tromeric domains (Figure S7A). We then filtered the SNP dataset to include only transversions and calculated θ across the genome. There was a positive relationship between θ for transversions only and the predicted mutation rate within all domains except H3K9me3 (Figure S7B). These results show that while RIP probably has a large contribution to genetic diversity in regions of H3K9me3, it does not solely drive the relationship between θ and predicted mutation rate.

Re-analysis of data from Wang et al. 2020

Wang et al. (2020) estimated the rate of spontaneous mutation during meiosis in *N. crassa*. During meiosis a genome defence mechanism called repeat-induced point mutation (RIP) induces mainly $C \rightarrow T$ transitions in duplicated regions of the genome resulting in a very high overall mutation rate (Wang et al., 2020). While not made explicit by Wang et al. (2020), the duplicated regions correspond almost completely to the H3K9 trimethylated domains. In order to better compare our results for asexual mutation rate in different domains to the sexual mutation rate estimated in their study, we re-analyzed the data from Wang et al. (2020) provided in their supplementary material, and included the information about chromatin domains. Their data are comprised of mutations in sequenced tetrads, which correspond to the products of a single meiosis. We included only those tetrads originating from crosses between non-mutant strains. This leaves 67 tetrads in the data that originate from five different crosses.

First we split the mutations to those that occurred in euchromatin and to those that occurred in H3K9 trimethylated domains. We observed that the numbers of mutations occurring in euchromatin and H3K9me3 domains for a given tetrad had very different distributions (Figure S11A), number of mutations occurring per tetrad in the H3K9me3 domains had a very long tail. When we examined the number of mutations per tetrad by cross, we observed a median of 22 mutations that occurred in euchromatic regions per tetrad, with some differences among the five crosses. However, the variation among tetrads from the different crosses was similar (Figure S11B). However, there were a median of 38 mutations that occurred in the H3K9me3 domains per tetrad, but a huge variation among tetrads, even within tetrads from a single cross (Figure S11B). For example, some tetrads

from the same cross had 20 to 40 mutations, while others could have hundreds. In cross E the range of mutations was from 27 in one tetrad to 1187 in another. Variation among mutations in the H3K9me3 domains per tetrad suggest that while there probably were some genetic influences on the mutation rate in the different crosses, there was substantial heterogeneity in the activation of RIP that was independent of genetic effects.

We calculated the mutation rate per meiosis for the euchromatic regions of the genome using a multilevel model with cross as a random factor. The model was

$$\begin{aligned} y_i &\sim \mathrm{Poisson}(\lambda_i) \\ \log(\lambda_i) &= \bar{\alpha} + \alpha_{c[i]} \\ \bar{\alpha} &\sim \mathrm{N}(0, 10) \\ \alpha_c &\sim \mathrm{N}(0, \sigma_c) \\ \sigma_c &\sim \mathrm{hT}(3, 0, 10) \end{aligned} \tag{S13}$$

where y_i is the number of mutations in euchromatic regions in the ith tetrad, $\bar{\alpha}$ is the average intercept, α_c is deviation from average intercept for each cross, and σ_c is the cross standard deviation. Prior for σ_c was the half-location scale version of Student's t-distribution, with 3 degrees of freedom, location 0, and scale 10. Based on posterior predictive checks, this model fitted the data. Mutation rate was calculated from posterior distribution of $\bar{\alpha}$ as

$$\mu = \frac{\exp(\bar{\alpha})}{N n_t} \tag{S14}$$

where N is the number of called nucleotides, and n_t is the number of tetrads. The mutation rate in euchromatic regions during sexual reproduction was 1.07 [0.6, 1.67] $\times 10^{-8}$ mutations / meiosis / bp.

The data for mutations that occurred in the H3K9me3 domains are clearly overdispersed. To

calculate the mutation rate per meiosis for the H3K9me3 domains we also modelled the heterogeneity among the tetrads. We fitted a gamma-poisson model, also called a negative binomial model, to the data. A gamma-poisson model allows each observation, a tetrad in our case, to have a different poisson rate allowing us to model this heterogeneity in observed rates (McElreath, 2015). We fitted a model

$$\begin{aligned} y_i &\sim \text{Gamma-Poisson}(\lambda_i, \phi) \\ \log(\lambda_i) &= \bar{\alpha} + \alpha_{c[i]} \\ \bar{\alpha} &\sim \text{N}(0, 10) \\ \alpha_c &\sim \text{N}(0, \sigma_c) \\ \sigma_c &\sim \text{hT}(3, 0, 10) \\ \phi &\sim \Gamma(0.01, 0.01) \end{aligned} \tag{S15}$$

where y_i is the number of mutations in the H3K9me3 domains in the ith tetrad, ϕ is the dispersion parameter, and other parameters were same as above. The prior for ϕ was a gamma distribution with shape of 0.01 and scale 0.01. Posterior predictive check indicated that the model fit the data reasonably well. The mutation rate was calculated from the average intercept as above. The mutation rate in H3K9 trimethylated regions during sexual reproduction was 2.54 [0.11, 7.55] $\times 10^{-7}$ mutations / meiosis / bp. As a result of rate heterogeneity there is quite a bit of uncertainty in the estimate. The ratio of mutation rates in the H3K9me3 regions over the euchromatic regions was 23.7 [0.99, 76.38]. While the 95% interval of the ratio slightly overlaps one due to large uncertainly in mutation rate in the H3K9me3 regions, mutation rate those regions seems higher.

We examined the spectrum of mutations that occurred in the euchromatic and the H3K9me3 regions separately, in the same way we did for asexual mutations. We observed that in the H3K9me3 regions there was a substantial over-representation of C:G $\rightarrow$ T:A transitions due to the action of RIP (Figure S11C). However, the mutation spectra that occurred in euchromatic regions was much

more similar to the one we observed during asexual reproduction in euchromatic regions. There was no difference in the relative mutation rate of C:G $\rightarrow$ T:A transitions during sexual and asexual reproduction in euchromatic regions. Some of the transversions did have different relative rates: A:T $\rightarrow$ C:G, and C:G $\rightarrow$ G:C transversions had higher rate during sexual reproduction, while C:G $\rightarrow$ A:T transversions had a lower relative mutation rate during sexual reproduction (Figure S12).

Our analysis gives somewhat different results compared to those of Wang et al. (2020), who only calculated mutation rates across the whole genome, and did not take variation among tetrads or crosses into account. We do find higher mutation rates during sexual reproduction than during asexual reproduction, suggesting that in *N. crassa* meiosis is mutagenic in addition to the RIP effect in the H3K9me3 domains. However, the mutation rate per meiosis was much smaller than that estimated by Wang et al. (2020). The H3K9 trimethylated regions contain mainly degraded transposable elements, and are quite gene poor. If we compare non-synonymous mutations in euchromatic and H3K9me3 regions, of those mutations that occurred in euchromatic regions 22.16% were non-synonymous, while only 0.17% of mutations were non-synonymous in H3K9 methylated regions. Thus, the very high mutation rate observed in H3K9 regions due to action of RIP, does not necessarily translate into a high genetic load. We suggest that the mutation load during sexual reproduction in *N. crassa* may not be as high as it has been suggested by Wang et al. (2020).

Supplementary References

Abyzov, A., Urban, A. E., Snyder, M., and Gerstein, M., 2011. CNVnator: An approach to discover, genotype, and characterize typical and atypical CNVs from family and population genome sequencing. *Genome Research*, **21**(6):974–984.

Chiang, C., Layer, R. M., Faust, G. G., Lindberg, M. R., Rose, D. B., Garrison, E. P., Marth, G. T., Quinlan, A. R., and Hall, I. M., 2015. Speedseq: ultra-fast personal genome analysis and interpretation. *Nature Methods*, **12**(10):966–968.

Cingolani, P., Platts, A., Coon, M., Nguyen, T., Wang, L., Land, S., Lu, X., and Ruden, D., 2012. A

program for annotating and predicting the effects of single nucleotide polymorphisms, SnpEff: SNPs in the genome of *Drosophila melanogaster* strain w1118; iso-2; iso-3. *Fly*, **6**(2):80–92.

Danecek, P., Bonfield, J. K., Liddle, J., Marshall, J., Ohan, V., Pollard, M. O., Whitwham, A., Keane, T., McCarthy, S. A., Davies, R. M., *et al.*, 2021. Twelve years of SAMtools and BCFtools. *GigaScience*, **10**(2). giab008.

Davis, R. H. and de Serres, F. J., 1970. Genetic and microbiological research techniques for *Neurospora crassa*. *Methods in Enzymology*, **17**:79–143.

Faust, G. G. and Hall, I. M., 2014. SAMBLASTER: fast duplicate marking and structural variant read extraction. *Bioinformatics*, **30**(17):2503–2505.

Freitag, M., Hickey, P. C., Raju, N. B., Selker, E. U., and Read, N. D., 2004. GFP as a tool to analyze the organization, dynamics and function of nuclei and microtubules in *Neurospora crassa*. *Fungal Genetics and Biology*, **41**(10):897–910.

Galagan, J. E., Calvo, S. E., Borkovich, K. A., Selker, E. U., Read, N. D., Jaffe, D., FitzHugh, W., Ma, L.-J., Smirnov, S., Purcell, S., *et al.*, 2003. The genome sequence of the filamentous fungus *Neurospora crassa*. *Nature*, **422**(6934):859–868.

Gelman, A., Goodrich, B., Gabry, J., and Vehtari, A., 2019. R-squared for Bayesian regression models. *The American Statistician*, **73**(3):307–309.

Homer, N., 2021. DWGSIM: Whole genome simulator for next-generation sequencing. github repository.

Jeffares, D. C., Jolly, C., Hoti, M., Speed, D., Shaw, L., Rallis, C., Balloux, F., Dessimoz, C., Bähler, J., and Sedlazeck, F. J., *et al.*, 2017. Transient structural variations have strong effects on quantitative traits and reproductive isolation in fission yeast. *Nature Communications*, **8**:14061.

Kelleher, J., Ness, R. W., and Halligan, D. L., 2013. Processing genome scale tabular data with wormtable. *BMC Bioinformatics*, **14**(1):1–5.

Kosugi, S., Momozawa, Y., Liu, X., Terao, C., Kubo, M., and Kamatani, Y., 2019. Comprehensive evaluation of structural variation detection algorithms for whole genome sequencing. *Genome Biology*, **20**(1):117.

Kronholm, I., Ormsby, T., McNaught, K. J., Selker, E. U., and Ketola, T., 2020. Marked *Neurospora crassa* strains for competition experiments and Bayesian methods for fitness estimates. *G3: Genes|Genomes|Genetics*, **10**:1261–1270.

Kühl, M., Stich, B., and Ries, D., 2021. Mutation-Simulator: fine-grained simulation of random mutations in any genome. *Bioinformatics*, **37**(4):568–569.

Layer, R. M., Chiang, C., Quinlan, A. R., and Hall, I. M., 2014. LUMPY: a probabilistic framework for structural variant discovery. *Genome Biology*, **15**(6):R84.

Li, H., 2013. Aligning sequence reads, clone sequences and assembly contigs with BWA-MEM. *arXiv:1303.3997 [q-bio.GN]*, .

Lichius, A. and Zeilinger, S., 2019. Application of membrane and cell wall selective fluorescent dyes for live-cell imaging of filamentous fungi. *JoVE*, (153):e60613.

McElreath, R., 2015. *Statistical Rethinking - A Bayesian course with examples in R and Stan*. CRC Press, New York.

McKenna, A., Hanna, M., Banks, E., Sivachenko, A., Cibulskis, K., Kernytsky, A., Garimella, K., Altshuler, D., Gabriel, S., Daly, M., *et al.*, 2010. The Genome Analysis Toolkit: a MapReduce framework for analyzing next-generation DNA sequencing data. *Genome Research*, **20**(9):1297–1303.

Metzenberg, R. L., 2003. Vogel's medium N salts: Avoiding the need for ammonium nitrate. *Fungal Genetics Newsletter*, **50**:14.

Min, Y. and Agresti, A. a., 2002. Modeling nonnegative data with clumping at zero: A survey. *JIRSS*, **1**(1):7–33.

Rausch, T., Zichner, T., Schlattl, A., Stütz, A. M., Benes, V., and Korbel, J. O., 2012. DELLY: structural variant discovery by integrated paired-end and split-read analysis. *Bioinformatics*, **28**(18):i333–i339.

Rueden, C. T., Schindelin, J., Hiner, M. C., DeZonia, B. E., Walter, A. E., Arena, E. T., and Eliceiri, K. W., 2017. ImageJ2: ImageJ for the next generation of scientific image data. *BMC Bioinformatics*, **18**(1):529.

Schindelin, J., Arganda-Carreras, I., Frise, E., Kaynig, V., Longair, M., Pietzsch, T., Preibisch, S., Rueden, C., Saalfeld, S., Schmid, B., *et al.*, 2012. Fiji: an open-source platform for biological-image analysis. *Nature Methods*, **9**(7):676–682.

Smith, K. M., Phatale, P. A., Sullivan, C. M., Pomraning, K. R., and Freitag, M., 2011. Heterochromatin is required for normal distribution of *Neurospora crassa* CenH3. *Molecular and Cellular Biology*, **31**(12):2528–2542.

Song, Q. and Smith, A. D., 2011. Identifying dispersed epigenomic domains from ChIP-Seq data. *Bioinformatics*, **27**(6):870–871.

Thorvaldsdóttir, H., Robinson, J. T., and Mesirov, J. P., 2013. Integrative Genomics Viewer (IGV): high-performance genomics data visualization and exploration. *Briefings in Bioinformatics*, **14**(2):178–192.

Vehtari, A., Gelman, A., and Gabry, J., 2017. Practical Bayesian model evaluation using leave-one-out cross-validation and WAIC. *Statistics and Computing*, **27**(5):1413–1432.

Wala, J. A., Bandopadhayay, P., Greenwald, N. F., O'Rourke, R., Sharpe, T., Stewart, C., Schumacher, S., Li, Y., Weischenfeldt, J., Yao, X., *et al.*, 2018. SvABa: genome-wide detection of structural variants and indels by local assembly. *Genome Research*, **28**(4):581–591.

Wang, L., Sun, Y., Sun, X., Yu, L., Xue, L., He, Z., Huang, J., Tian, D., Hurst, L. D., and Yang, S., *et al.*, 2020. Repeat-induced point mutation in *Neurospora crassa* causes the highest known mutation rate and mutational burden of any cellular life. *Genome Biology*, **21**(1):142.

Xie, C. and Tammi, M. T., 2009. CNV-seq, a new method to detect copy number variation using high-throughput sequencing. *BMC Bioinformatics*, **10**(1):80.

Xing, Y., Dabney, A. R., Li, X., Wang, G., Gill, C. A., and Casola, C., 2020. SECNVs: a simulator of copy number variants and whole-exome sequences from reference genomes. *Frontiers in Genetics*, **11**:82.

Ye, K., Schulz, M. H., Long, Q., Apweiler, R., and Ning, Z., 2009. Pindel: a pattern growth approach to detect break points of large deletions and medium sized insertions from paired-end short reads. *Bioinformatics*, **25**(21):2865–2871.

Supplementary Figures

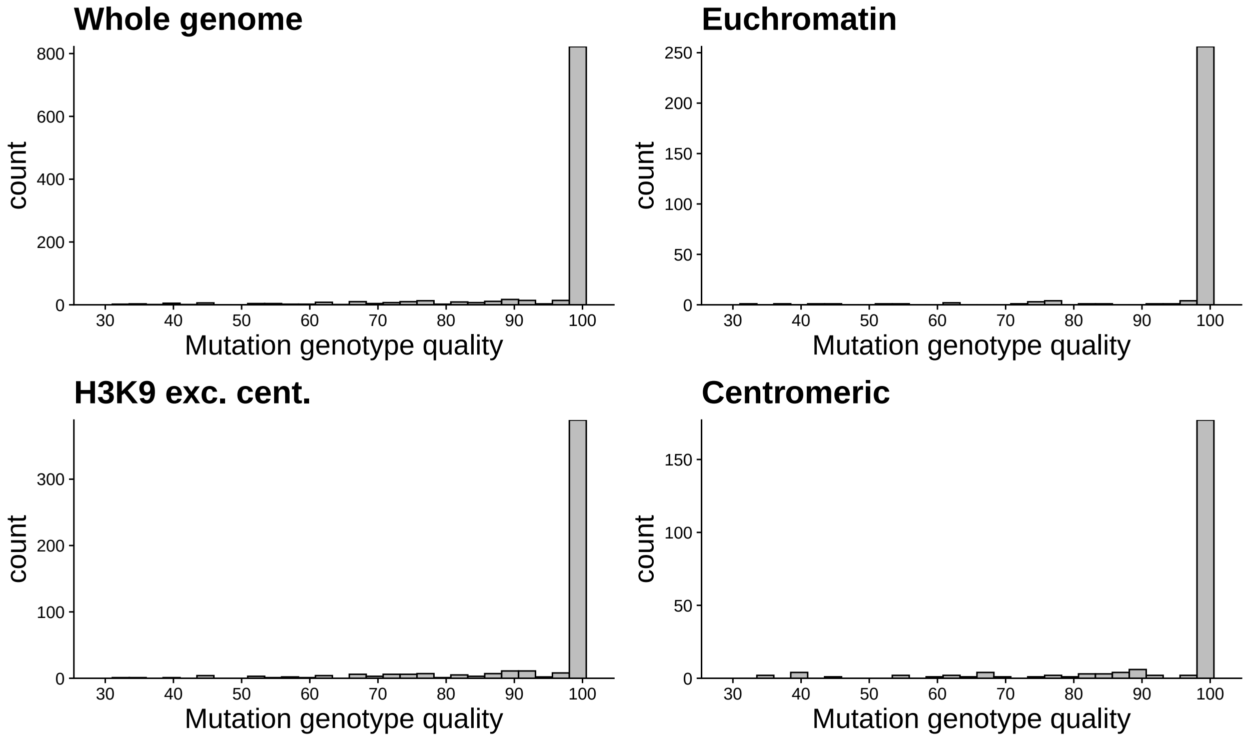

Figure S1: Distribution of genotype qualities of observed mutations given by GATK. Distributions are shown for the whole genome, euchromatin, H3K9me3 domains excluding centromeric regions, and centromeric regions.

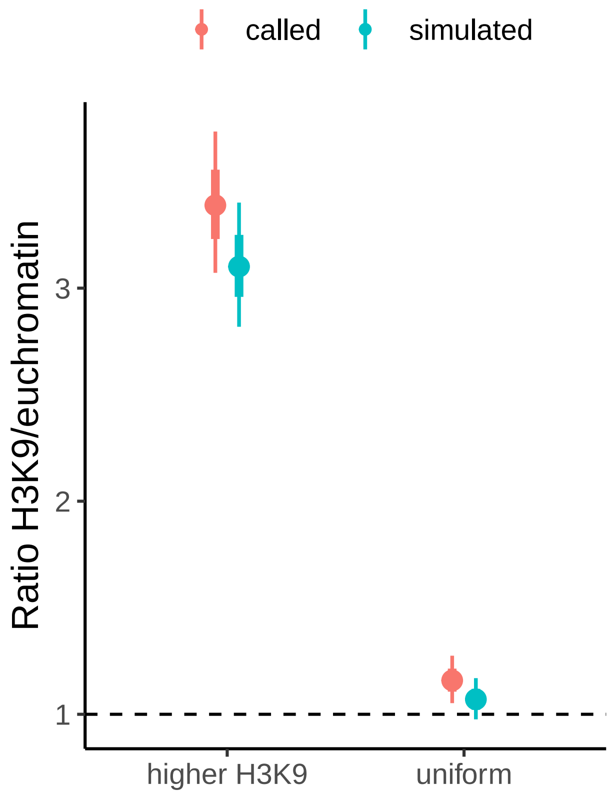

Figure S2: H3K9me3 / euchromatin mutation rate ratio in the simulated data. Estimates calculated from called mutations from the simulated reads are in red, and estimates calculated using the true simulated mutations are in blue. The two different scenarios are: mutation rate was higher in regions of the genome marked by H3K9me3, and mutation rate was uniform across the genome. Points are means and range shows the 95% HPD interval of the ratio. Interval estimates overlap in both scenarios.

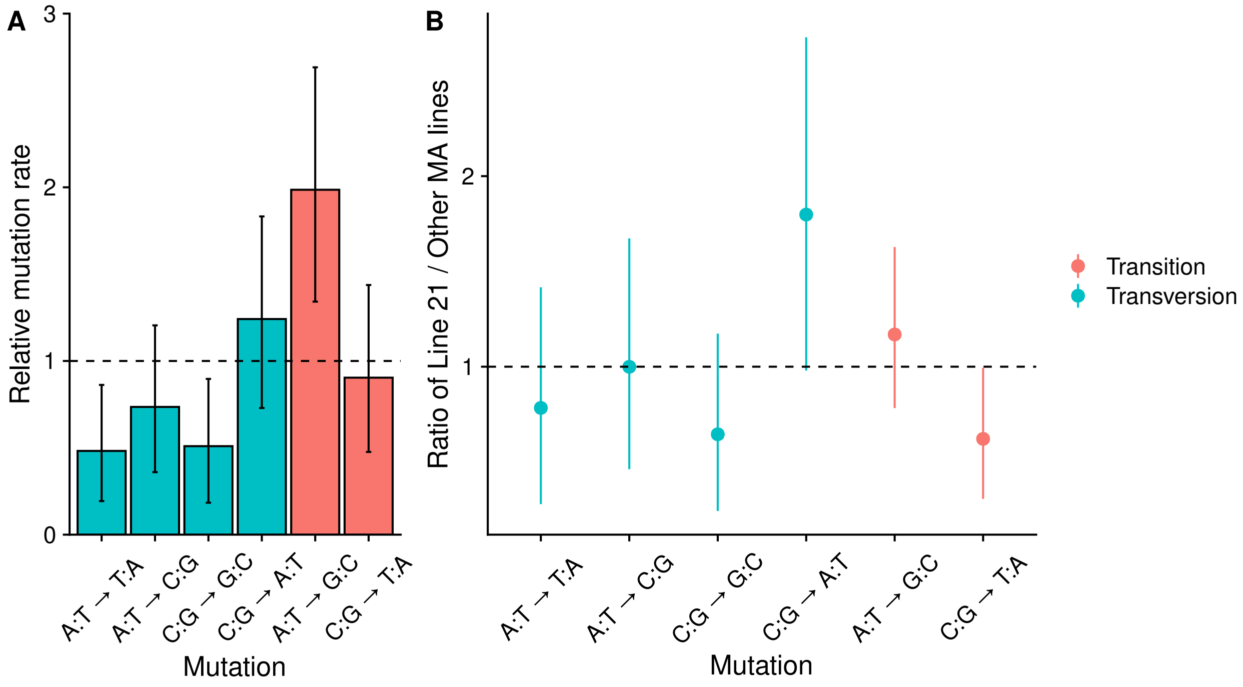

Figure S3: Mutation spectra for the MA line 21. A) Relative mutation rates. B) Ratios of relative mutation rates for line 21 / rest of the MA lines. Intervals for C:G → A:T transversions and C:G → T:A transitions barely overlap one.

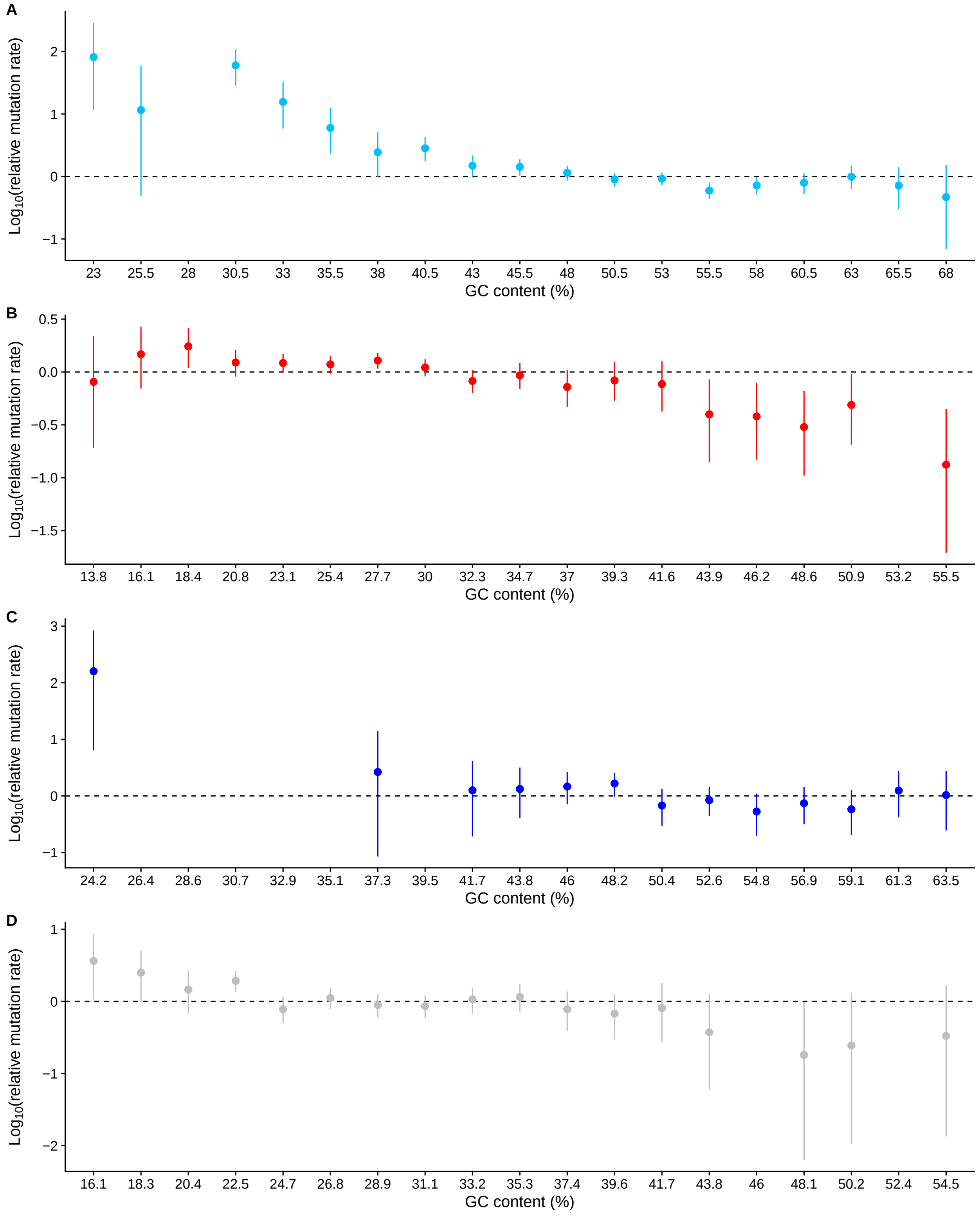

Figure S4: GC-content and relative mutation rate within domains. Relative mutation rates for windows of 200 bp binned for GC-content at 2.5 percentage point intervals. Ticks on the horizontal axis are at the end points of intervals. Note that y-axis is on a $\log_{10}$ scale, the dashed line indicates relative mutation rate of one. Some bins did not contain any mutations, so estimates are missing for those bins. A) Euchromatic regions B) H3K9me3 domains C) H3K27me3 domains D) Centromeric regions.

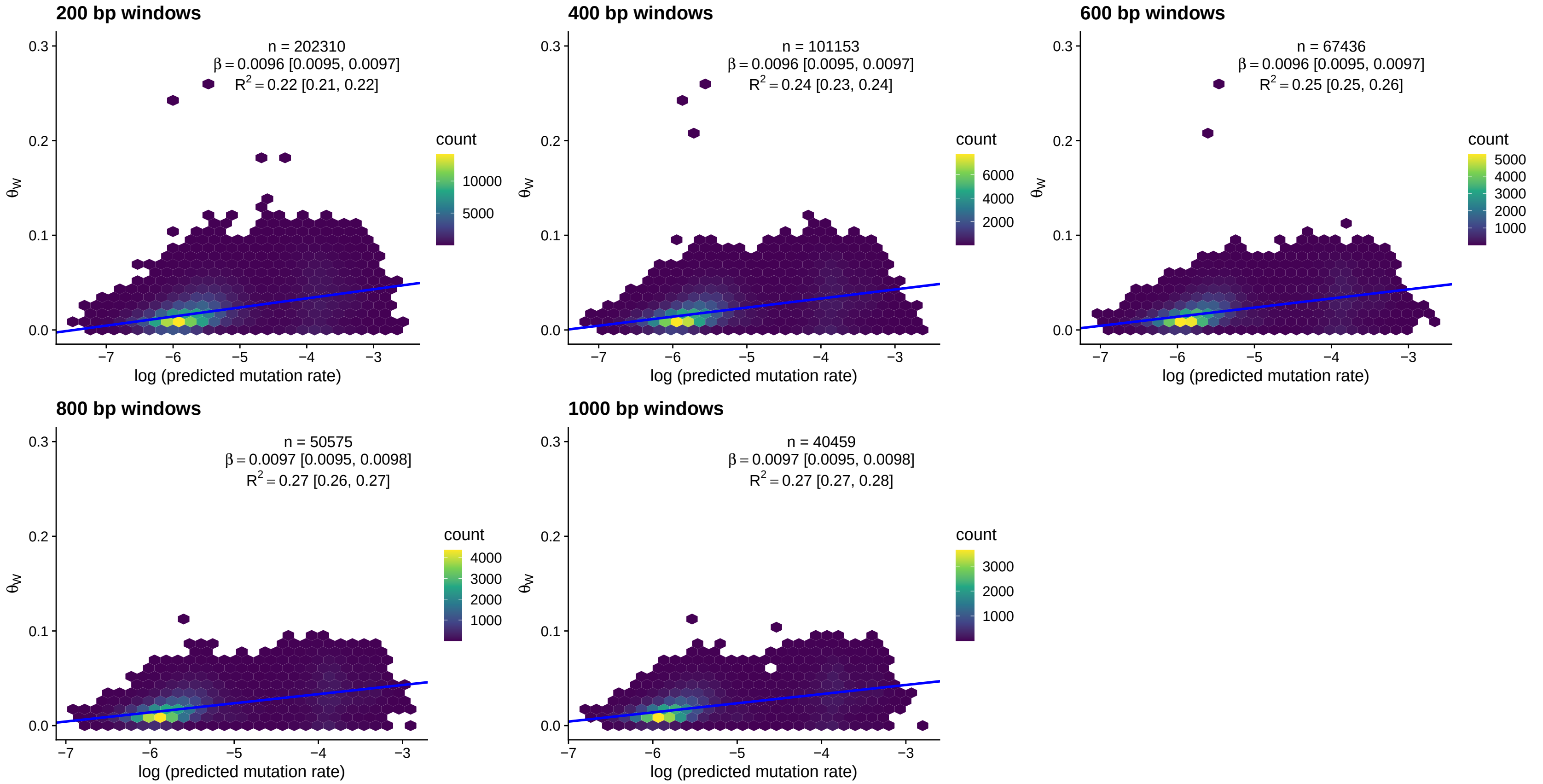

Figure S5: Effect of window size on regression between the predicted mutation rate and the observed nucleotide polymorphism in natural populations. Results have been calculated for different window sizes. n is the number of windows, β is the slope of the regression line, and R^2 is the Baysian R^2 value, a measure of model fit.

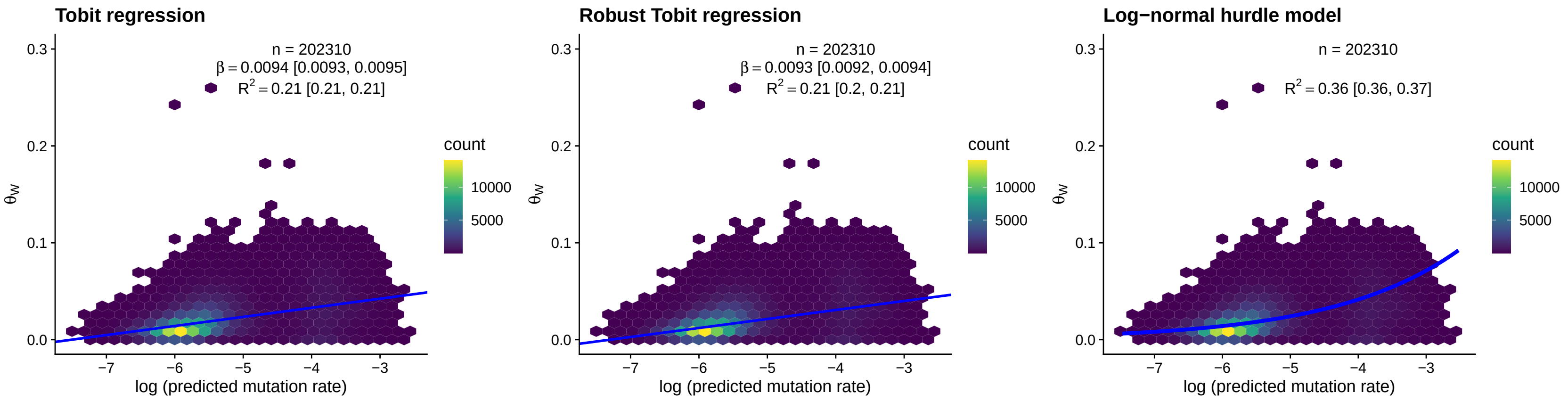

Figure S6: Effect of different models on regression between the predicted mutation rate and the observed nucleotide polymorphism. To check the robustness of results to windows where $\theta = 0$, different models were used. Window size = 200 bp, n is the number of windows, β is the slope of the regression line, and R^2 is the Baysian R^2 value, a measure of model fit.

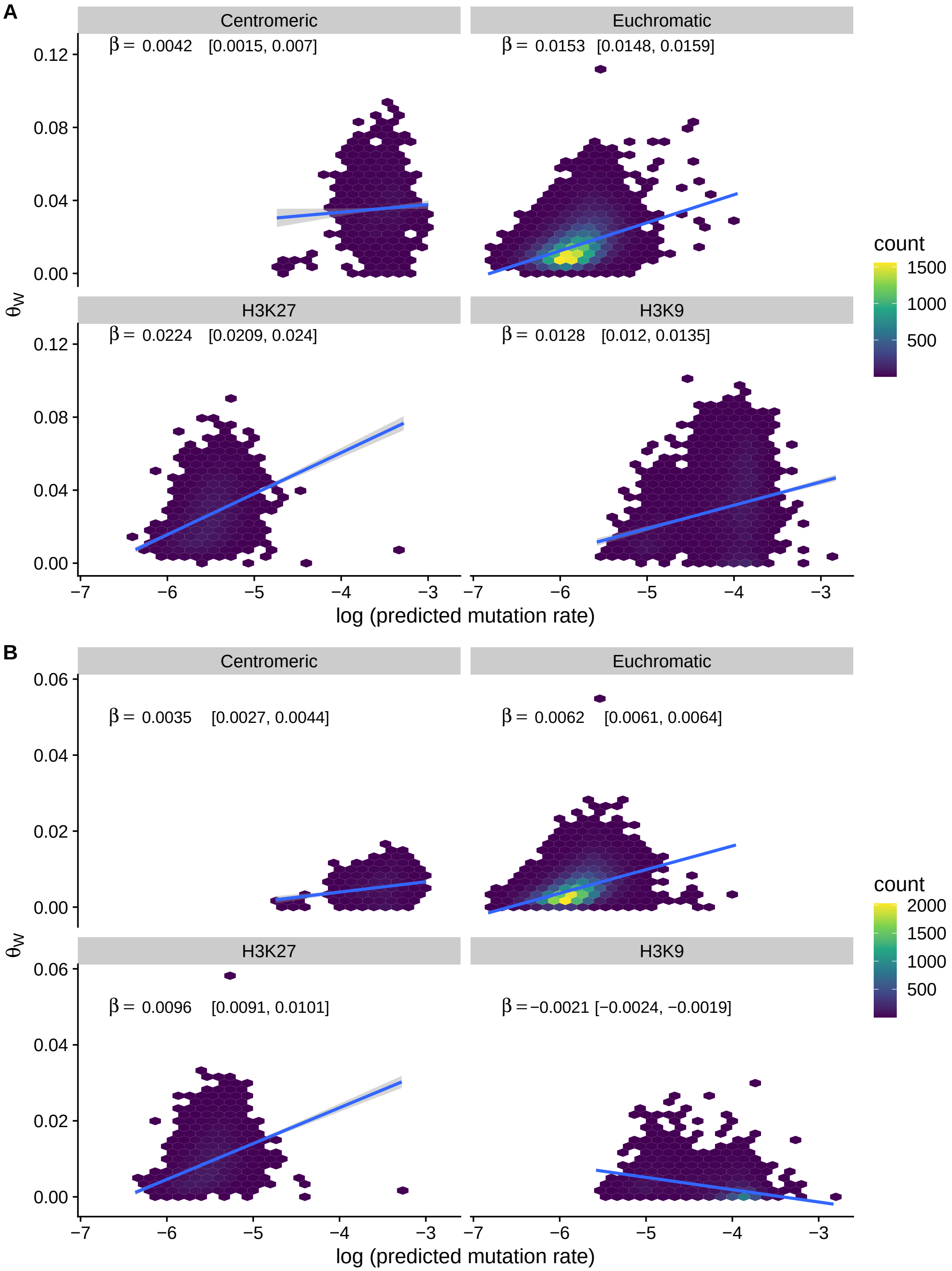

Figure S7: Regression between the predicted mutation rate and the observed nucleotide polymorphism within different regions of the genome. Window size was set to 1000 bp in both panels, as there are large number of windows where $\theta = 0$ for small window sizes in the transversions only data. $n = 40459$, β is the slope of the regression line. A) θ has been calculated for all SNPs, $R^2 = 0.32$ [0.32, 0.33]. B) θ has been calculated only for SNPs that represent transversions, $R^2 = 0.29$ [0.28, 0.30].

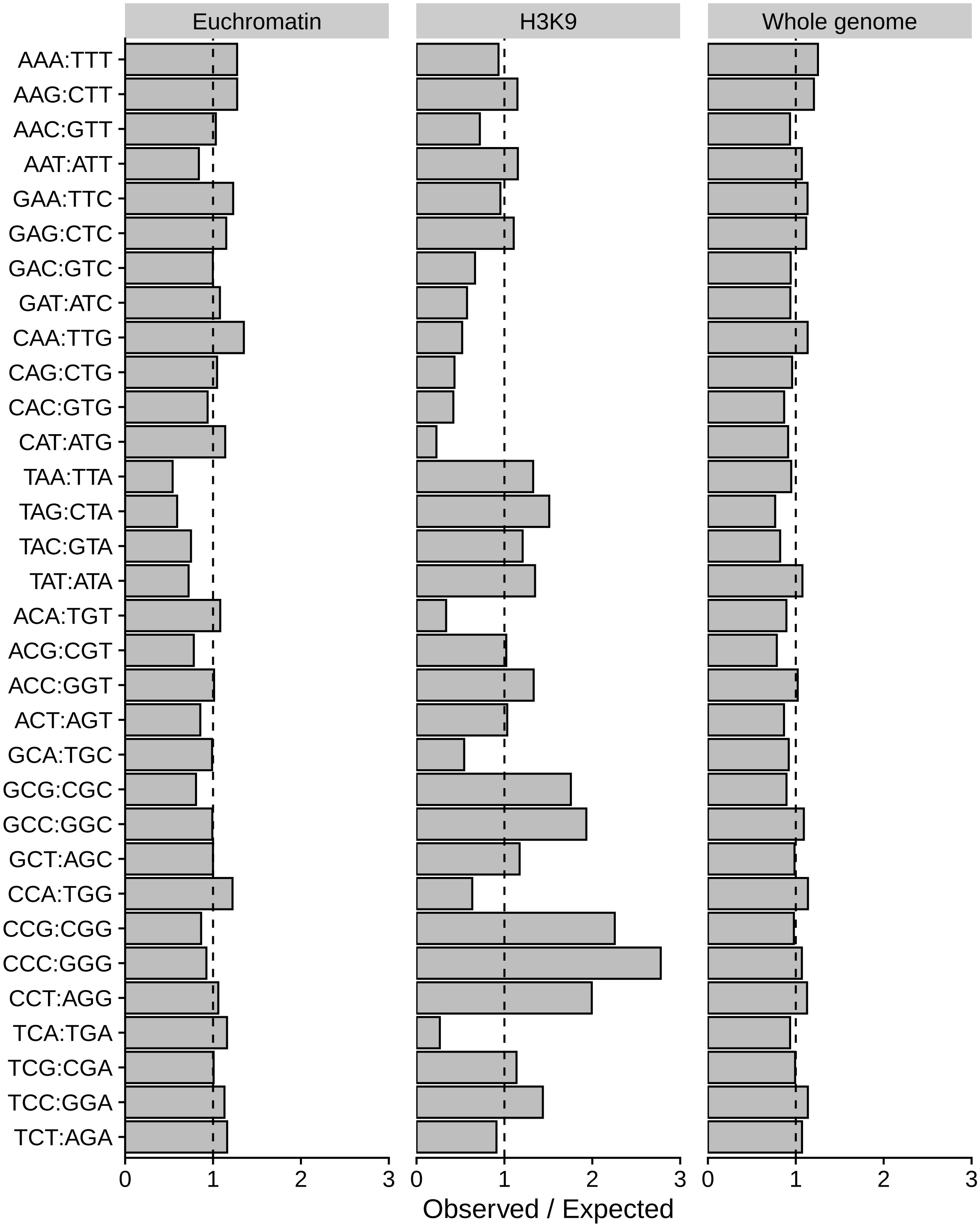

Figure S8: Observerved deviations of trinucleotide frequencies from expectations for different parts of the genome. Observed trinucleotide frequencies were divided by their expected frequencies based on GC-content. The dashed line shows the expected ratio of one.

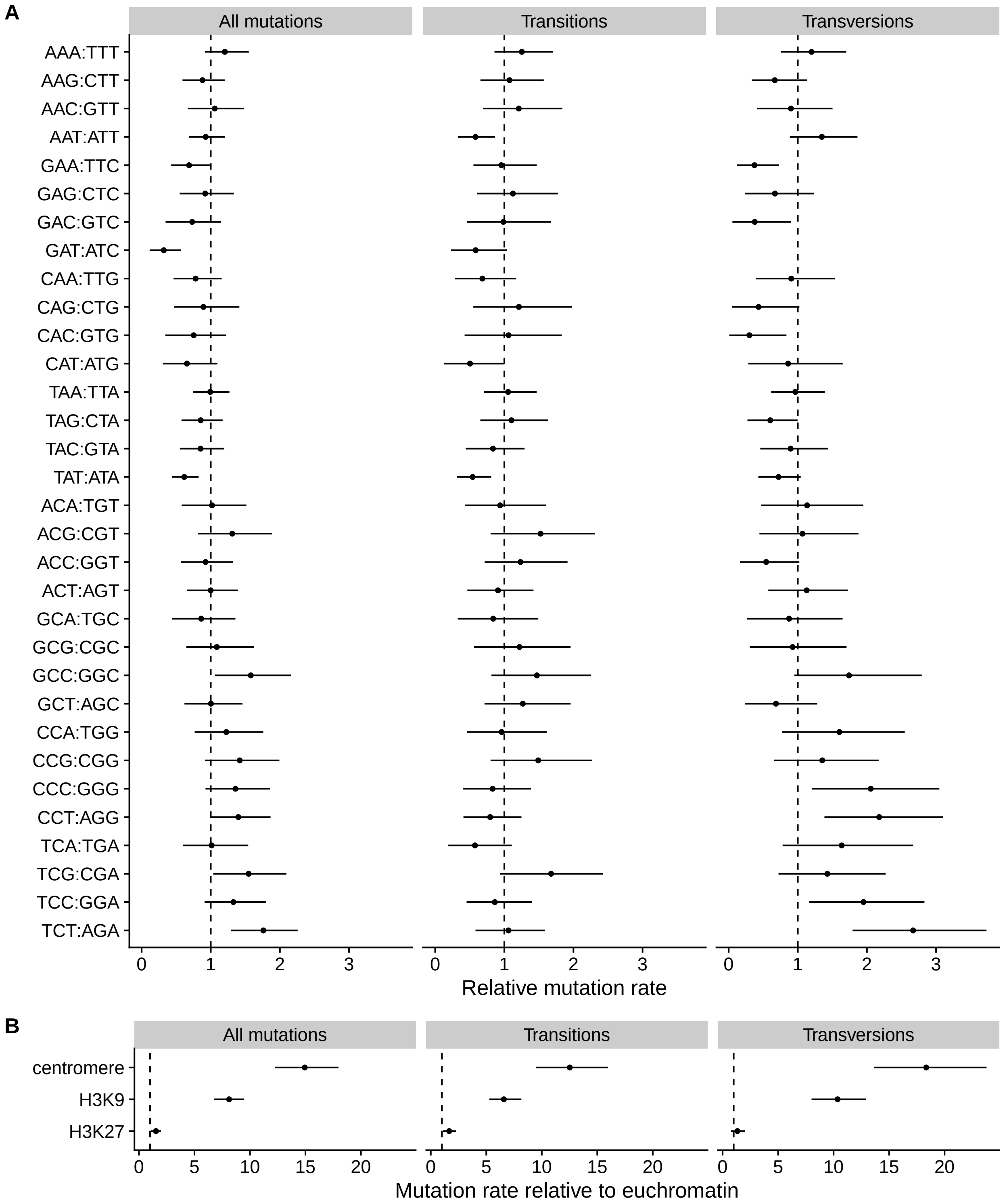

Figure S9: A) Relative mutation rates for the 32 different trinucleotide classes. B) Model estimates for relative mutation rates for centromeric, H3K9me3 and H3K27me3 domains from the trinucleotide model. Estimates are medians and range shows 95% HPD intervals.

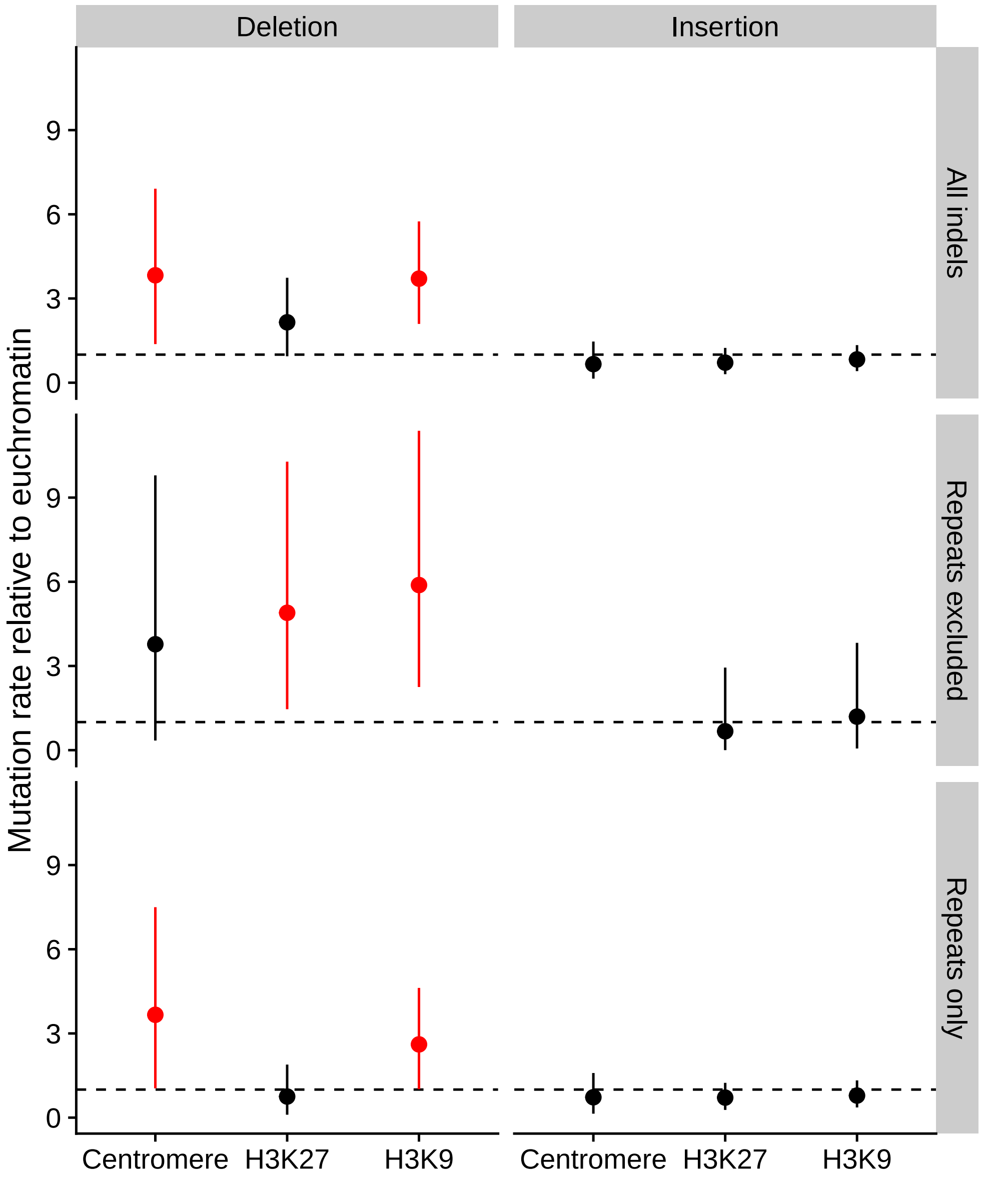

Figure S10: Mutation rates for indels in the different domains relative to euchromatin. The dashed line shows the relative mutation rate of one. Facets show deletions and insertions for all indels, for indels that did not occur in repeats, and for indels that occurred in repeated sequences. Estimates are medians and ranges show 95% HPD intervals. Intervals that do not overlap with one, that is, those where mutation rate is higher than in euchromatin are colored red.

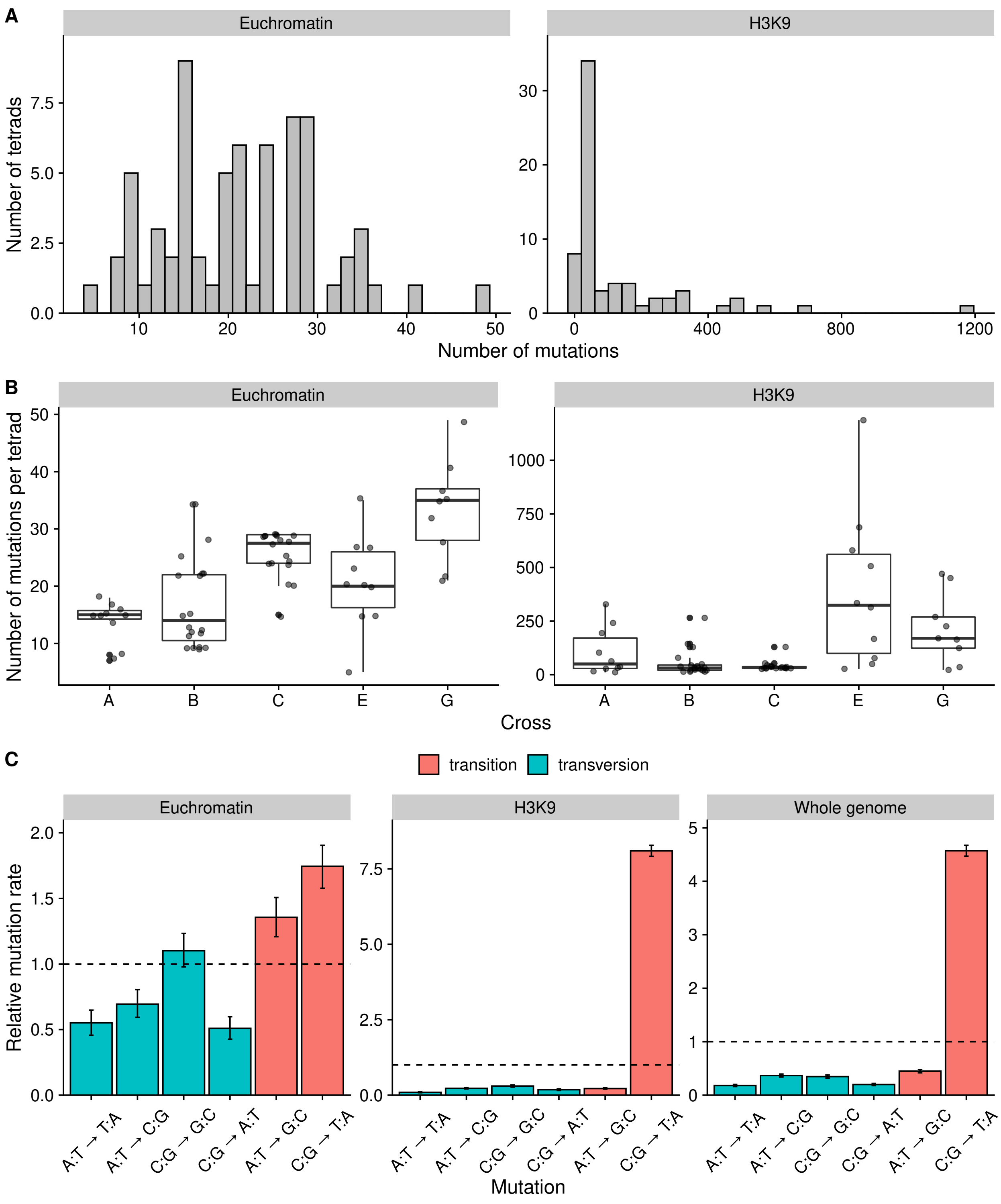

Figure S11: Mutations that occurred during sexual reproduction. Data is from Wang et al. (2020). Note that y-axis scales are different in different panels. A) The distribution of the number of mutations in the tetrads in euchromatin and H3K9 methylated domains. B) The number of mutations per tetrad for the different crosses. C) Spectrum of mutations for different regions of the genome. Error bars are 95% HPD intervals.

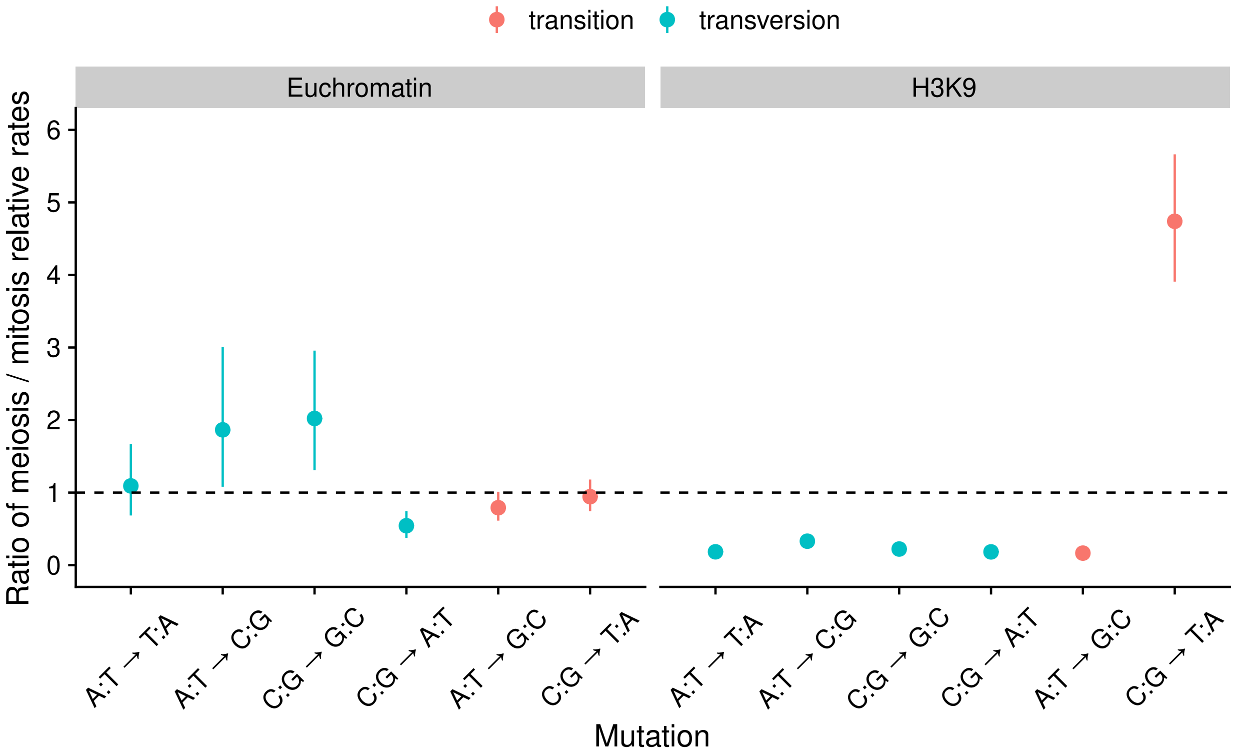

Figure S12: Ratios of the relative mutation rates during meiosis over mitosis. Points are medians and ranges show 95% HPD interval of the ratios. If the interval estimate is higher than one, mutation rate in meiosis is higher, if the interval estimate is lower than one, mutation rate in mitosis is higher.

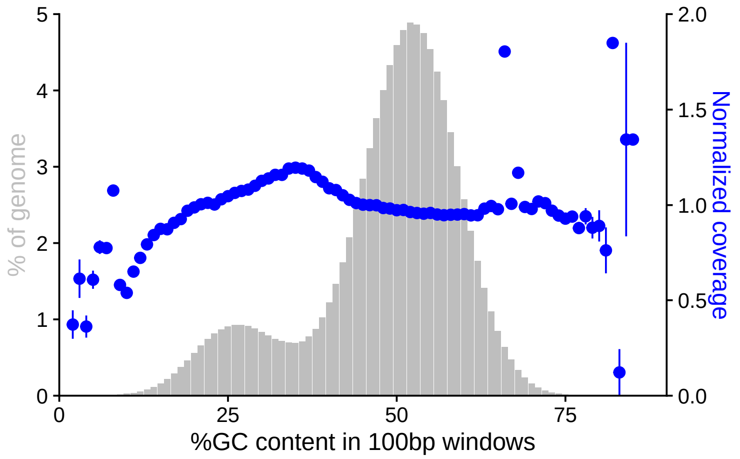

Figure S13: Sequencing coverage plotted against GC-content of the genome for the *mat A* ancestor. Other samples had similar profiles.

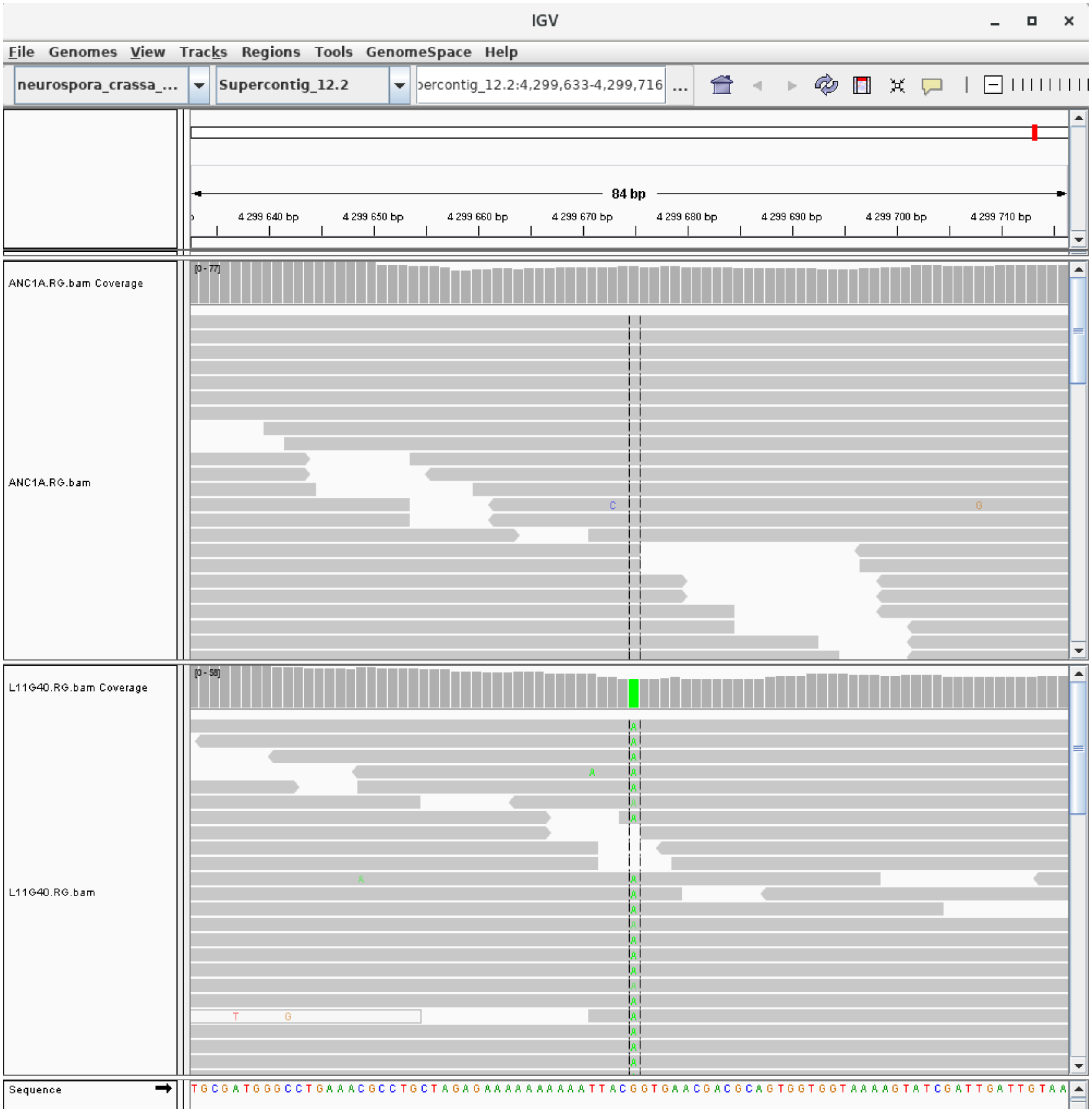

Figure S14: Screenshot of a mutation viewed in IGV. Upper track is the ancestor and lower track is MA line 11. Mutation is in chromosome 2, position 4 299 675, in euchromatin. Genotype quality of the mutation is 99.

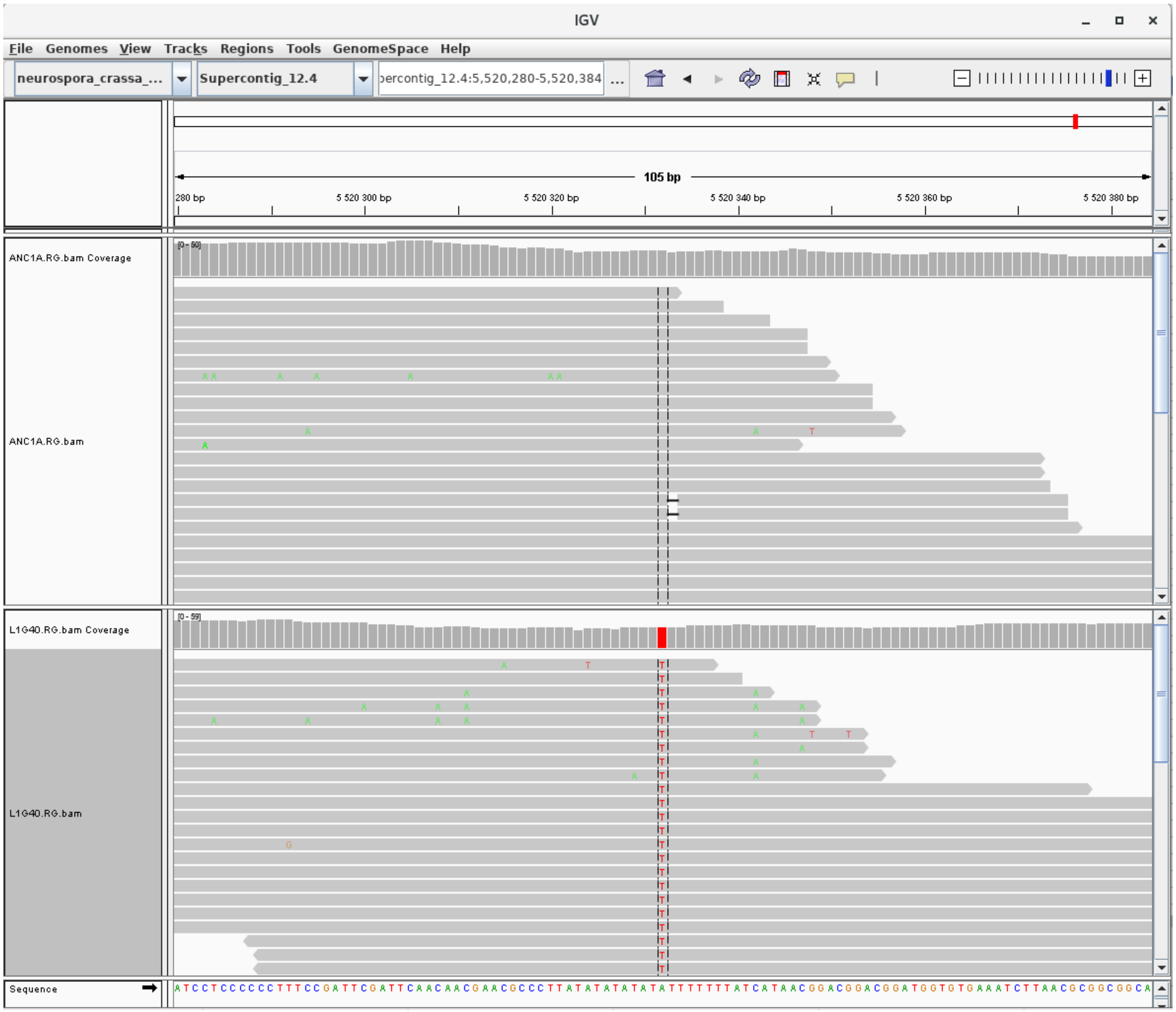

Figure S15: Screenshot of a mutation viewed in IGV. Upper track is the ancestor and lower track is MA line 1. Mutation is in chromosome 4, position 5 520 332, in euchromatin. Genotype quality of the mutation is 75.

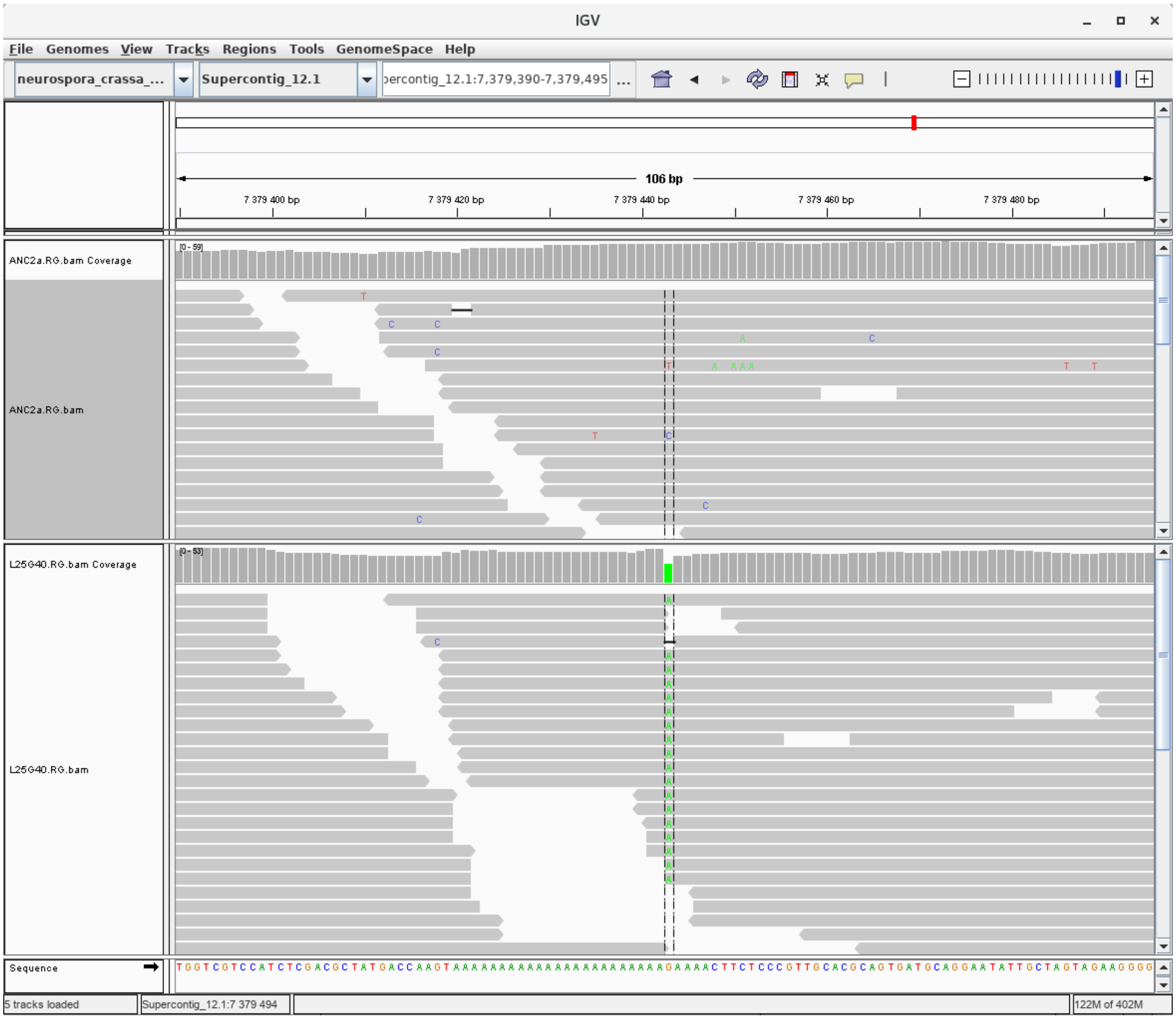

Figure S16: Screenshot of a mutation viewed in IGV. Upper track is the ancestor and lower track is MA line 25. Mutation is in chromosome 1, position 7 379 443, in euchromatin. Genotype quality of the mutation is 45 as the mutation is located in a repetitive region. This mutation was confirmed by Sanger sequencing.

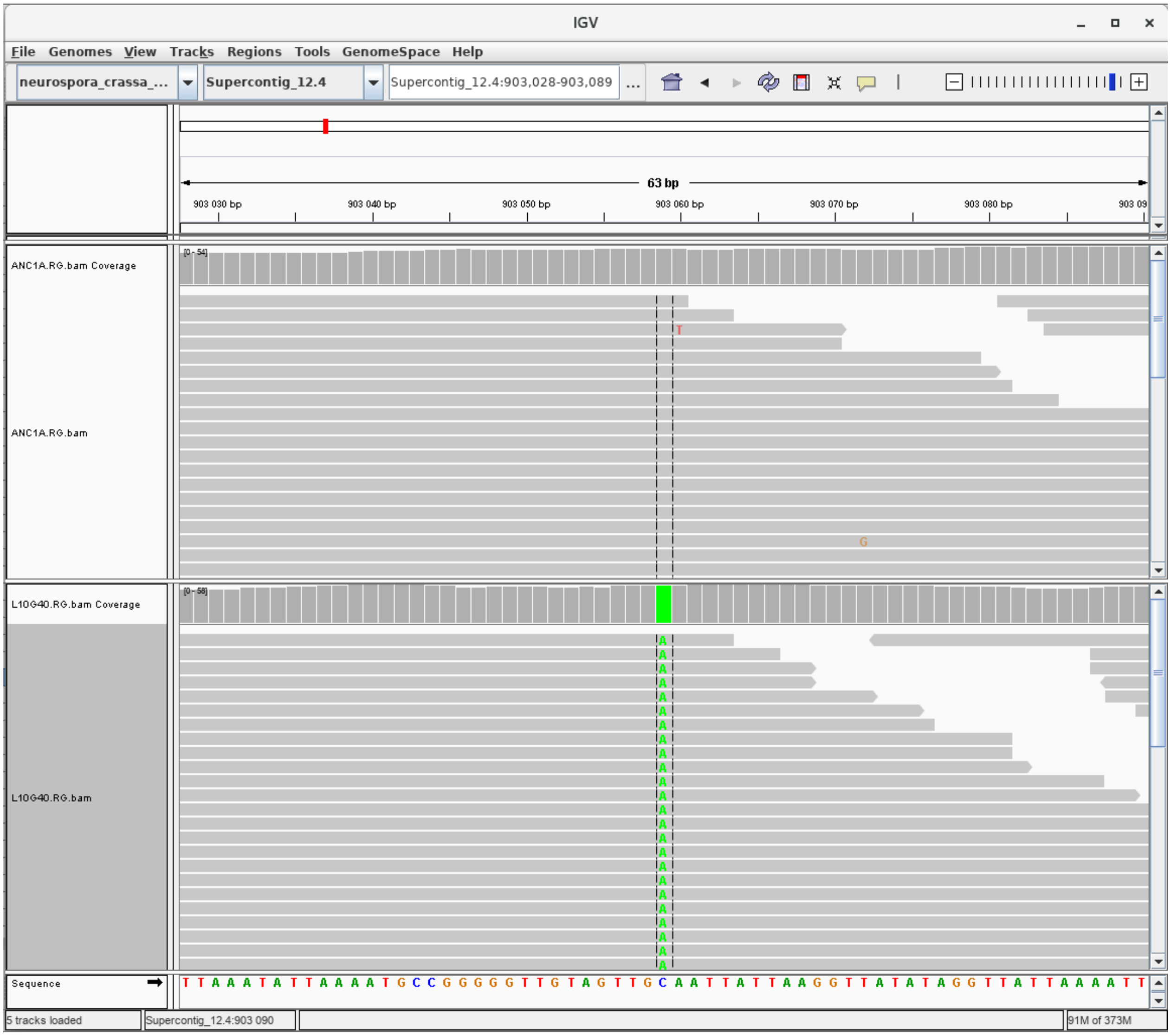

Figure S17: Screenshot of a mutation viewed in IGV. Upper track is the ancestor and lower track is MA line 10. Mutation is in chromosome 4, position 903 059, in centromeric region. Genotype quality of the mutation is 99.

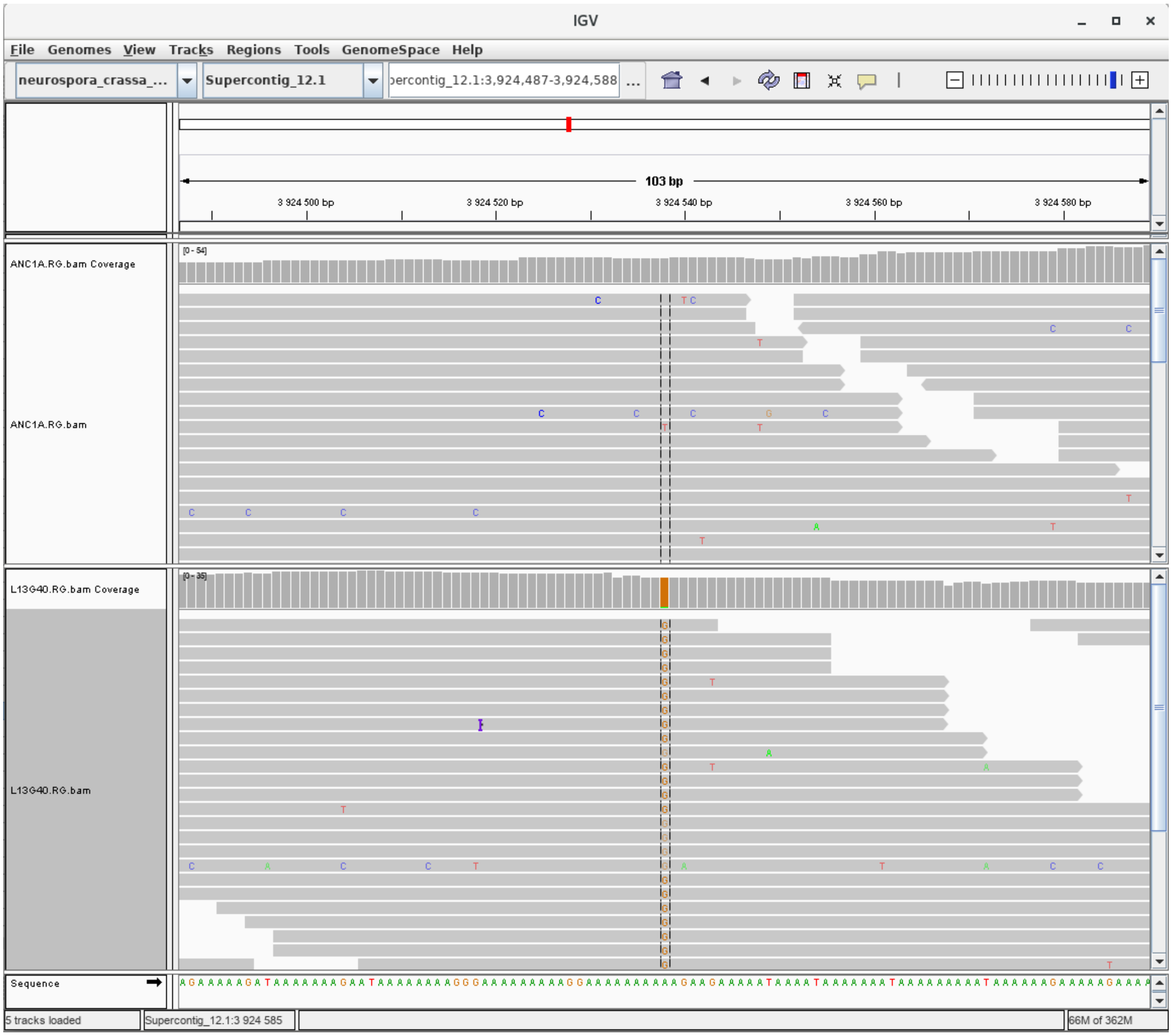

Figure S18: Screenshot of a mutation viewed in IGV. Upper track is the ancestor and lower track is MA line 13. Mutation is in chromosome 1, position 3 924 538, in centromeric region. Genotype quality of the mutation is 67 as the mutation is located in a repetitive region.

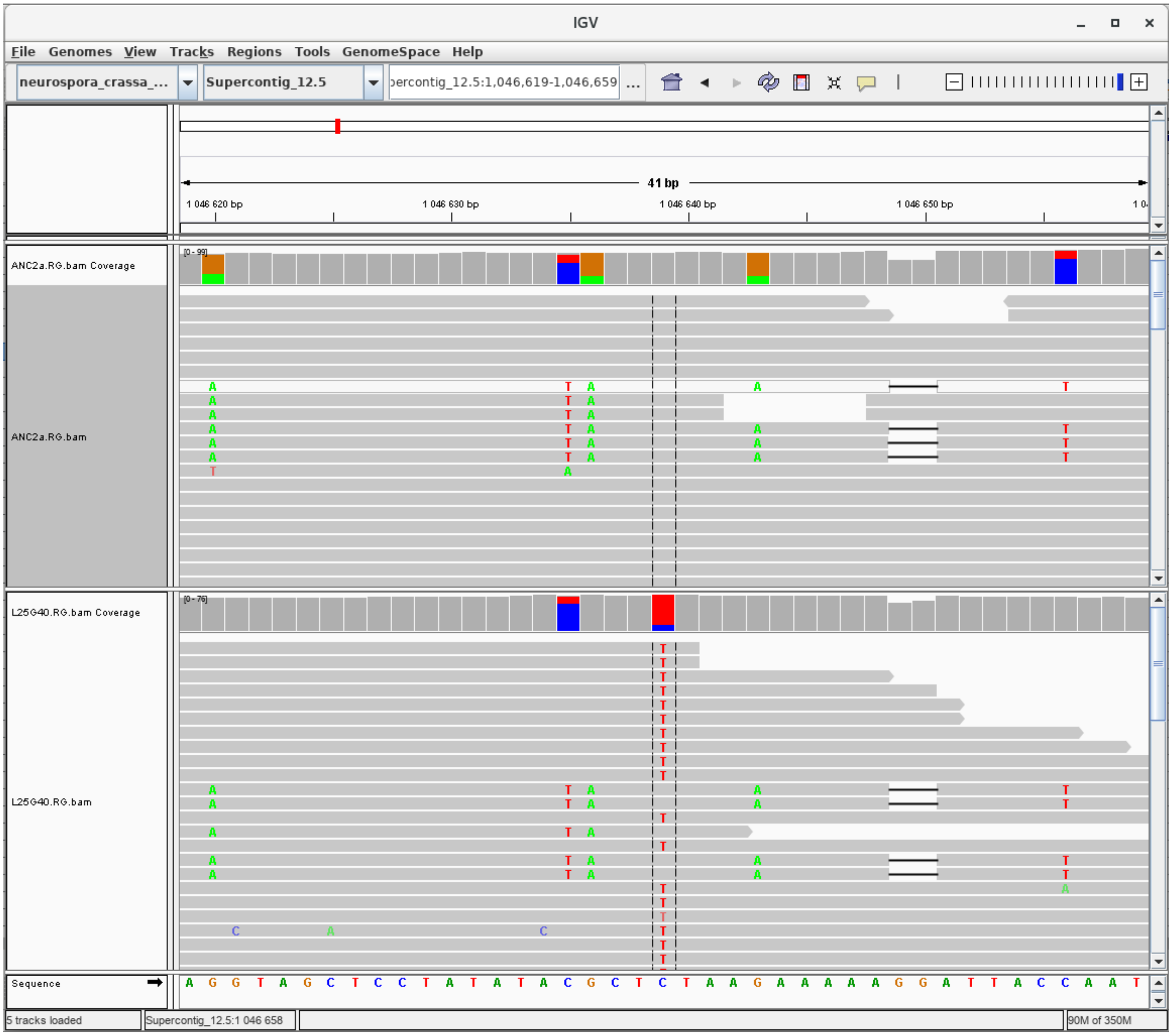

Figure S19: Screenshot of a mutation viewed in IGV. Upper track is the ancestor and lower track is MA line 25. Mutation is in chromosome 5, position 1 046 639, in centromeric region. Genotype quality of the mutation is 45 as the mutation is located in region with reduced mapping quality. Some reads that do not support the mutation map to this location. However, those reads also have other changes that are not supported by other reads. This suggest that reads not supporting the mutation are mapping errors. This mutation was confirmed by Sanger sequencing.

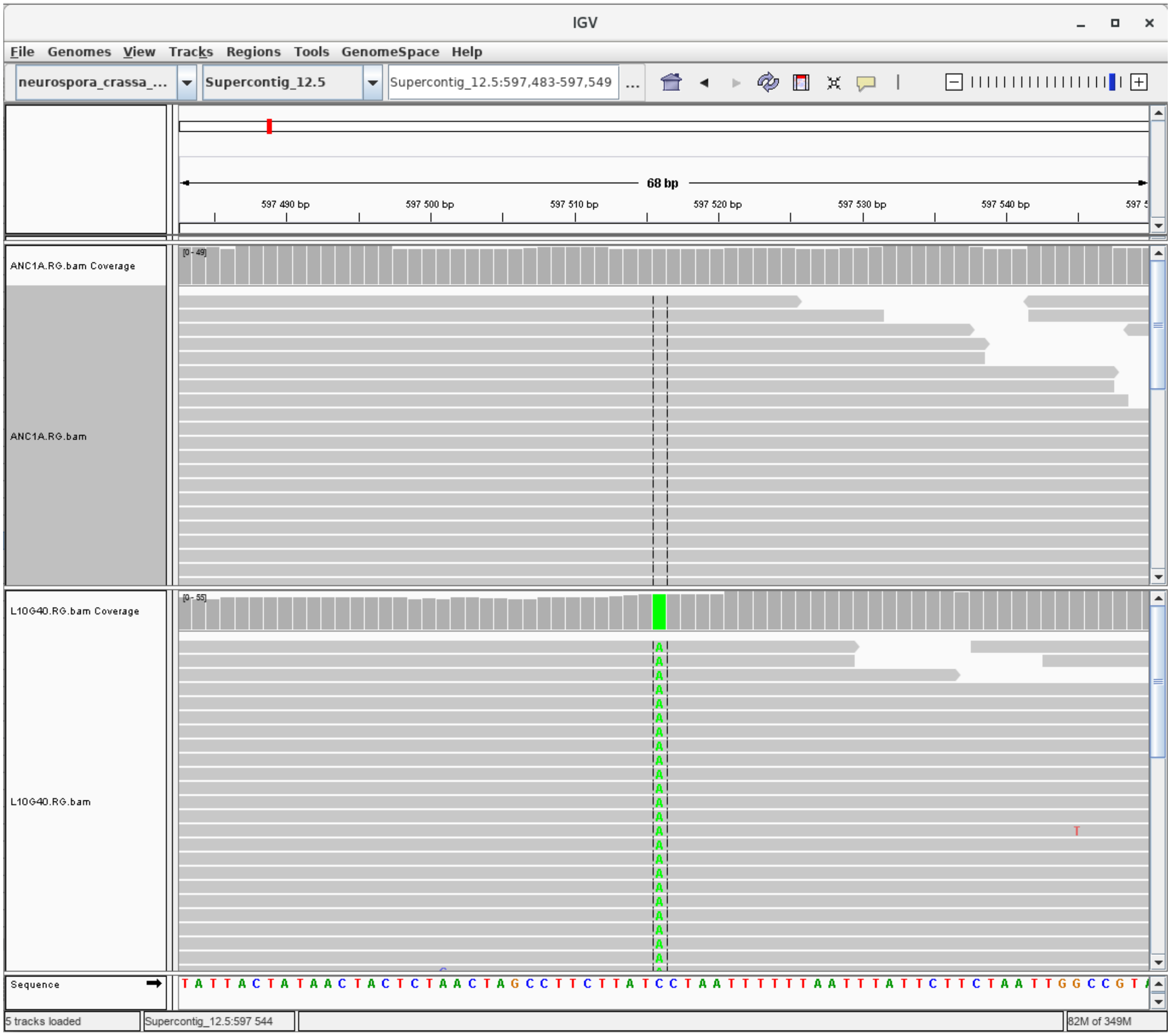

Figure S20: Screenshot of a mutation viewed in IGV. Upper track is the ancestor and lower track is MA line 10. Mutation is in chromosome 5, position 597 516, in region marked by H3K9 methylation. Genotype quality is 99.

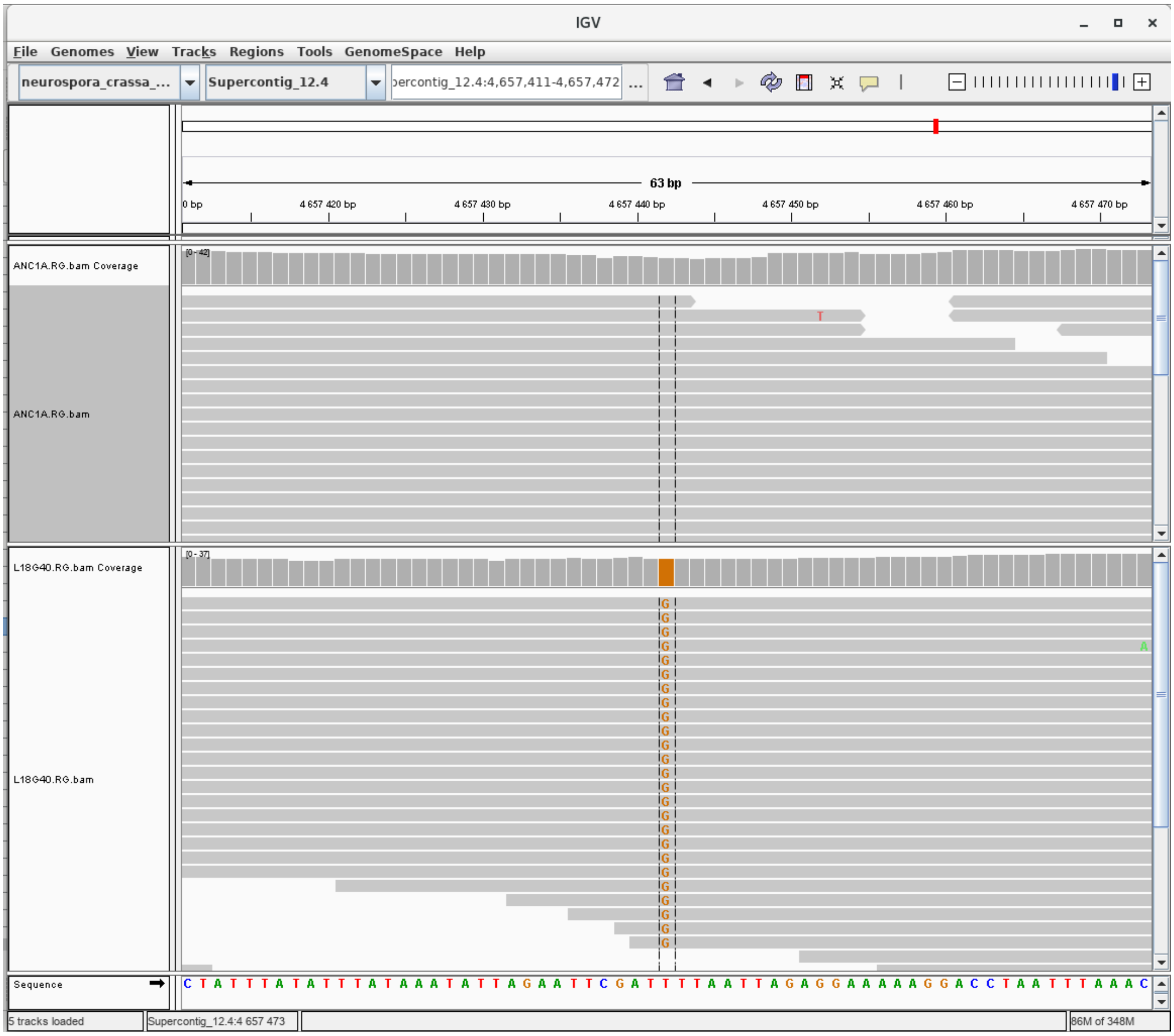

Figure S21: Screenshot of a mutation viewed in IGV. Upper track is the ancestor and lower track is MA line 18. Mutation is in chromosome 4, position 5 657 442, in region marked by H3K9 methylation. Genotype quality is 72.

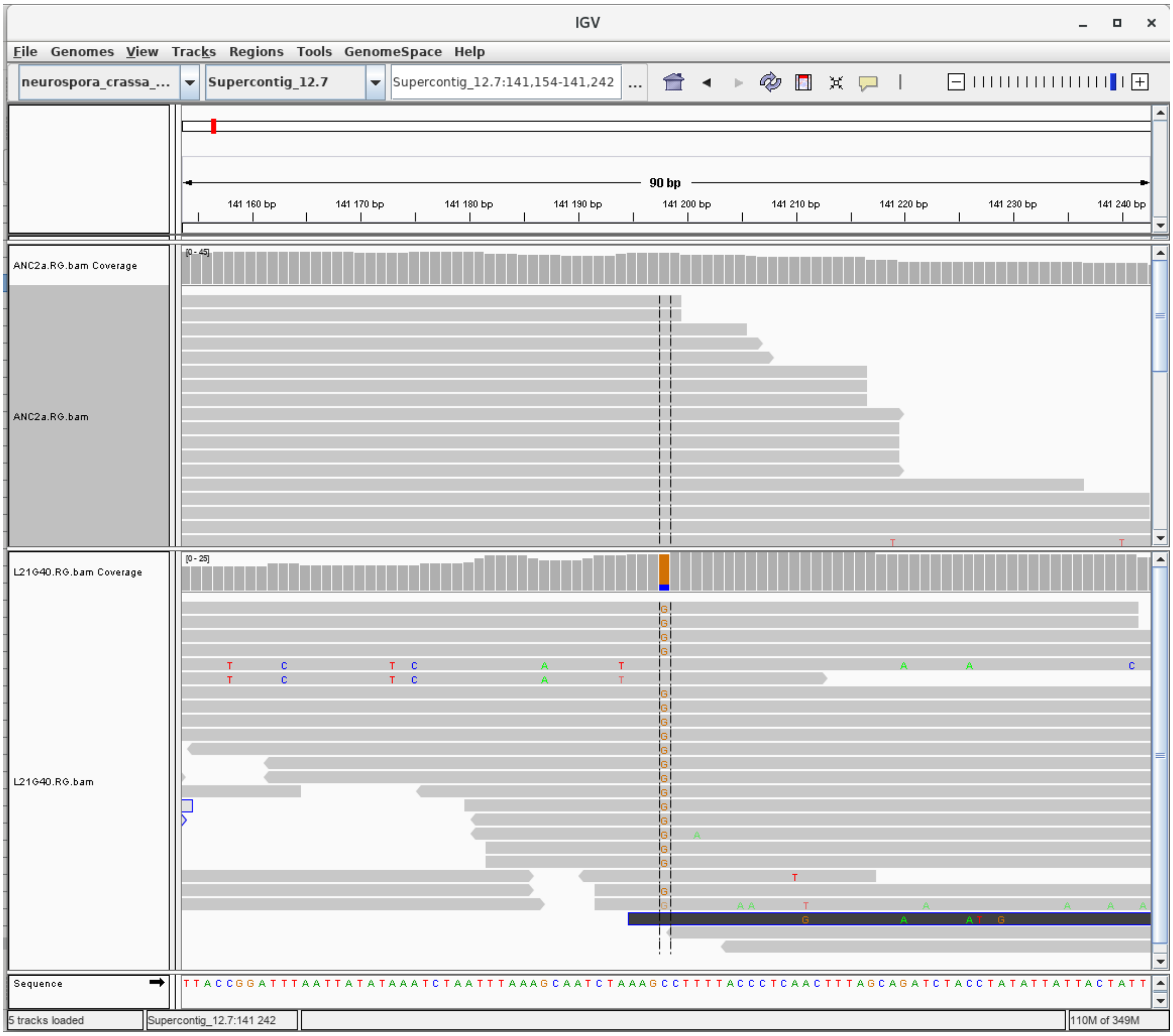

Figure S22: Screenshot of a mutation viewed in IGV. Upper track is the ancestor and lower track is MA line 21. Mutation is in chromosome 7, position 141 198, in region marked by H3K9 methylation. Genotype quality is 45. Some reads do not support the mutation. However, those reads have other changes that suggest a read mapping error. This mutation was confirmed by Sanger sequencing.

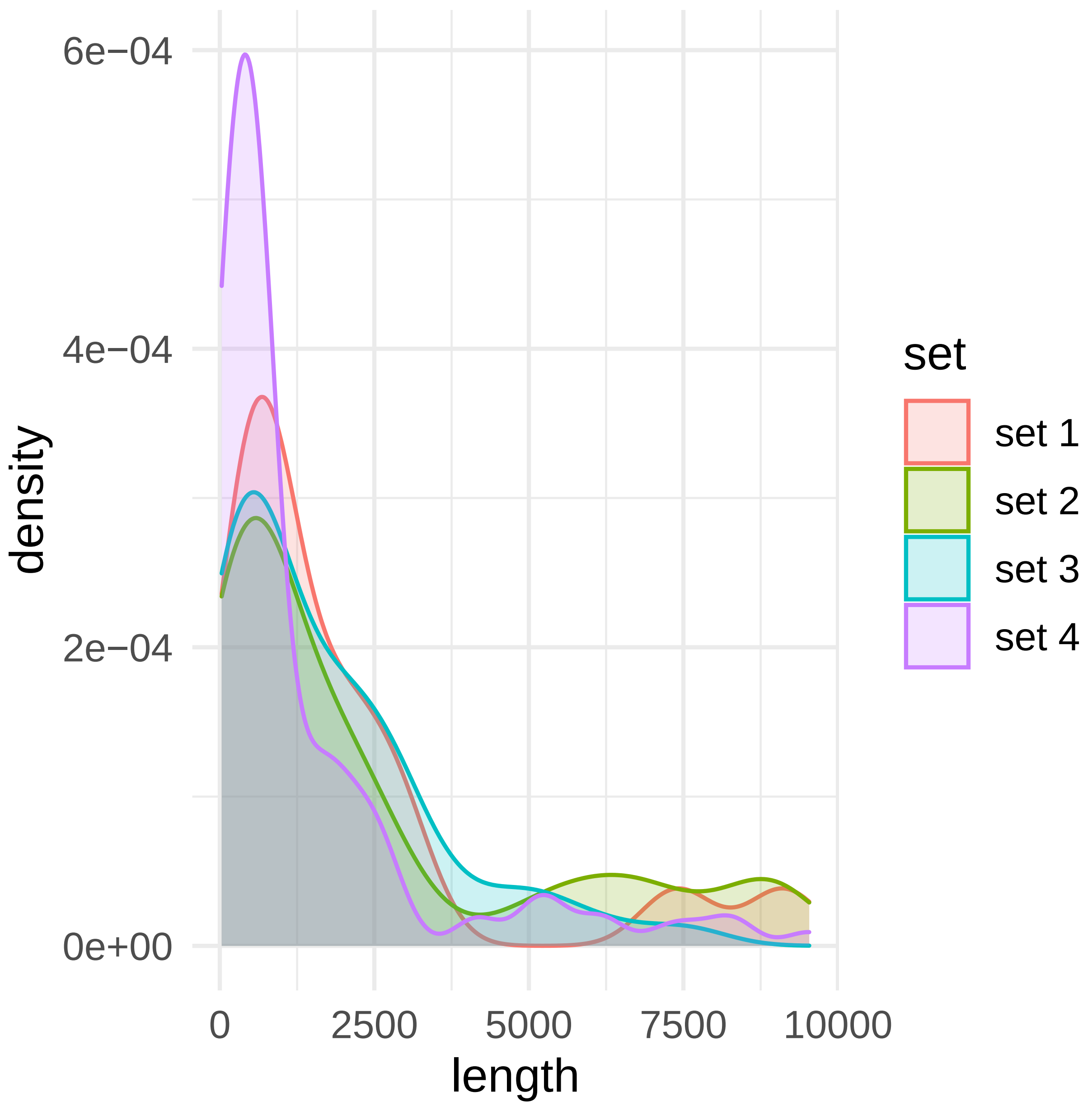

Figure S23: Densities for the length of distributions of SVs simulated using survivor. The characteristics of each simulated set are specified in the supplementary table S6.

Supplementary Tables

Table S1: Summary of alignment metrics for genomes used in this study. The ancestors used to start the MA experiment were: B 26708, which is 2489 *mat A*, and B 26709, which is 2489 *mat a*. Lines L1–L20 are *mat A* and L21–L40 are *mat a*.

Natural strains				MA lines			
Line	Number of reads	Depth	Mapped reads (%)	Line	Number of reads	Depth	Mapped reads (%)
10882	30683221	112	87.2	B 26708	15183018	55	98.5
10883	20920000	76	69.7	B 26709	18679696	68	98.3
10884	32294020	118	72.8	L1	13215012	48	98.2
10886	15265173	56	92.7	L2	15658340	57	98.2
10892	32124218	117	62.3	L3	14699884	54	98.3
10904	14718008	54	91.1	L4	16424902	60	98.2
10906	14935778	55	89.7	L5	16328002	60	97.6
10907	32213115	118	68.1	L6	15189850	55	98.3
10908	13924689	51	91.7	L7	14006386	51	98.4
10912	14829204	54	92	L8	13888092	51	98
10914	30158241	110	61.1	L9	14386986	53	98.3
10915	24410441	89	61.1	L10	15458492	56	98.6
10918	40163515	147	65.4	L11	13884490	51	98.1
10923	16231892	59	92	L12	15913482	58	98.2
10925	37031022	135	85.2	L13	20192750	74	97.8
10926	32682749	119	66	L14	17445964	64	98.1
10927	17741573	65	73.1	L15	14776588	54	98.5
10928	15048163	55	92	L16	14562124	53	98
10932	13402421	49	91.1	L17	16043040	59	97.9
10935	22304835	81	81.1	L18	14755826	54	97.7
10937	29757556	109	85.4	L19	13712542	50	98.1
10943	32838278	120	72.8	L20	18685746	68	96.9
10946	17261401	63	90.1	L21	14814794	54	98.5
10948	13835447	50	91	L22	15849832	58	98
10950	14364201	52	98	L23	17528602	64	98.3
10951	13354998	49	89.5	L24	14415798	53	97.8
10983	38349194	140	72.8	L25	14773870	54	98.1
1131	16974127	62	88.7	L26	17436754	64	98.2
1133	14540257	53	88.3	L27	14047860	51	98.2
1165	15095250	55	90	L28	16295790	59	98.3
3210	13681263	50	90.4	L29	16244750	59	98.1
3211	13908534	51	89.4	L30	19847786	72	98.1
3223	14095397	51	92.1	L31	19613222	72	97.9
3943	143993668	525	82.6	L33	33430658	122	97.9
3975	13671543	50	88.8	L34	15456254	56	97.9
4708	14310459	52	87.7	L35	14405808	53	98
4712	12602174	46	86	L36	15567642	57	97.8
4716	18336337	67	86.7	L37	15232240	56	98
4730	15588817	57	87.4	L38	14265830	52	98
4824	12921275	47	88.3	L39	16540462	60	98.1
5910	12494976	46	83.4	L40	18856392	69	98.3
6203	13266522	48	89.3				
851	12956372	47	89.7				
8783	15381794	56	88				
8790	13205097	48	90.1				
8816	15452652	56	89.6				
8819	13736103	50	87.5				
8845	15868843	58	86.9				
8850	12695261	46	89.3				
P4452	172179164	628	86.9				
P4463	21708426	79	69.7				
P4468	21129155	77	68.4				
P4471	24828717	91	72.4				
P4476	92452134	337	86.8				
P4479	22220857	81	72.5				
P4489	34624044	126	72.5				

Table S2: Model comparisons among different models that predict the mutation rate by GC-content and chromatin modifications. Model terms are different linear model parts, α is the intercept, β_{GC} is the slope effect of GC-content, β_{K9} is the effect of H3K9 domain, β_{K27} is the effect of H3K27 domain, β_C is the effect of centromeric domain, β_I is the interaction effect between GC-content and H3K9 domain, β_{I2} is the interaction effect between GC-content and centromeric domain, β_{I3} is the interaction effect between GC-content and H3K27 domain. d_i, g_i, and c_i are indicator variables, and x_i is GC-content in percentage points. WAIC = widely applicable information criterion, SE = standard error.

Model terms	WAIC	diff (± SE)	weight
$\alpha + \beta_{GC}x_i + \beta_{K9}d_i + \beta_{K27}g_i + \beta_C c_i + \beta_I x_i d_i$	454.47	0 (0)	0.63
$\alpha + \beta_{GC}x_i + \beta_{K9}d_i + \beta_{K27}g_i + \beta_C c_i + \beta_I x_i d_i + \beta_{I3}x_i g_i$	456.86	2.39 (2.19)	0.19
$\alpha + \beta_{GC}x_i + \beta_{K9}d_i + \beta_C c_i + \beta_I x_i d_i$	458.67	4.2 (6.94)	0
$\alpha + \beta_{GC}x_i + \beta_{K9}d_i$	458.84	4.37 (10.59)	0
$\alpha + \beta_{GC}x_i + \beta_{K9}d_i + \beta_C c_i + \beta_I x_i d_i + \beta_{I2}x_i c_i$	460.35	5.88 (7.17)	0
$\alpha + \beta_{GC}x_i + \beta_{K9}d_i + \beta_I x_i d_i$	495.86	41.39 (19.86)	0
$\alpha + \beta_{GC}x_i + \beta_{K9}d_i$	496.65	42.18 (22.43)	0
$\alpha + \beta_{GC}x_i$	546.62	92.15 (33.84)	0
$\alpha + \beta_{K9}d_i + \beta_C c_i$	614.83	160.36 (42.53)	0
$\alpha + \beta_{K9}d_i$	645.82	191.35 (49.19)	0
$\alpha + \beta_C c_i$	1290.02	835.55 (255.52)	0
α	1689.56	1235.09 (264.15)	0

Table S3: Model estimates for a model predicting mutation rate by GC-content, centromeric, H3K9, and H3K27 domains, α is the intercept, β_{GC} is the slope effect of GC-content, β_{K9} is the effect of H3K9me domain, β_{K27} is the effect of the H3K27me3 domain, β_C is the effect of centromeric domain, and β_I is the interaction effect between GC-content and H3K9me domain.

Parameter	Estimate [95% HPDI]
α	-2.61 [-3.34, -1.88]
β_C	0.51 [0.35, 0.67]
β_{K9}	-0.14 [-0.93, 0.63]
β_{K27}	0.32 [0.08, 0.55]
β_{GC}	-0.06 [-0.08, -0.05]
β_I	0.02 [0.00, 0.04]

Table S4: Model comparison among different models that predict the mutation rate by trinucleotide class and chromatin modifications. Model terms are different linear model parts. α is the intercept, β_t is a vector of effects for the 32 trinucleotide classes, β_{K9} is the effect of H3K9 domain, β_{K27} is the effect of H3K27 domain, β_C is the effect of centromeric domain, β_I is the interaction effect between trinucleotide class and H3K9 domain, β_{I2} is the interaction effect between trinucleotide class and centromeric domain. d_i, g_i, and c_i are indicator variables, and $x_{[t]}$ is the trinucleotide class. WAIC = widely applicable information criterion, SE = standard error.

Model terms	WAIC	diff (± SE)	weight
$\beta_t x_{[t]} + \beta_{K9} d_i + \beta_{K27} g_i + \beta_C c_i$	621.31	0 (0)	0.96
$\beta_t x_{[t]} + \beta_{K9} d_i + \beta_C c_i$	627.79	6.47 (6.95)	0.04
$\beta_t x_{[t]} + \beta_{K9} d_i + \beta_{K27} g_i + \beta_C c_i + \beta_I x_{[t]} d_i$	640.35	19.04 (14.69)	0
$\beta_t x_{[t]} + \beta_{K9} d_i + \beta_C c_i + \beta_I x_{[t]} d_i$	647.17	25.85 (16.26)	0
$\beta_t x_{[t]} + \beta_{K9} d_i + \beta_C c_i + \beta_I x_{[t]} d_i + \beta_{I2} x_{[t]} c_i$	652.37	31.06 (18.55)	0
$\beta_t x_{[t]} + \beta_{K9} d_i$	693.47	72.15 (21.8)	0
$\beta_t x_{[t]} + \beta_{K9} d_i + \beta_I x_{[t]} d_i$	717.79	96.48 (26.11)	0
$\beta_{K9} d_i + \beta_C c_i$	996.07	374.76 (67.31)	0
$\beta_{K9} d_i$	1043.35	422.04 (67.64)	0
$\beta_C c_i$	1370.5	749.19 (114.3)	0
α	1776.5	1155.19 (135.53)	0
$\beta_t x_{[t]}$	1916.35	1295.03 (134.38)	0

Table S5: Natural strains with sequencing data included in this study. Strains were obtained from FGSC. 33 strains were sequenced in this study and data for 23 strains were obtained from Zhao et al. (2015).

Strain	Source	Strain	Source
10948	This study	P4452	(Zhao et al., 2015)
10886	This study	P4463	(Zhao et al., 2015)
10932	This study	P4468	(Zhao et al., 2015)
1165	This study	P4471	(Zhao et al., 2015)
8816	This study	P4476	(Zhao et al., 2015)
3223	This study	P4479	(Zhao et al., 2015)
8845	This study	10882	(Zhao et al., 2015)
10908	This study	10883	(Zhao et al., 2015)
10904	This study	10884	(Zhao et al., 2015)
851	This study	10892	(Zhao et al., 2015)
1131	This study	10907	(Zhao et al., 2015)
8850	This study	10914	(Zhao et al., 2015)
8819	This study	10915	(Zhao et al., 2015)
4708	This study	10918	(Zhao et al., 2015)
4712	This study	10925	(Zhao et al., 2015)
6203	This study	10926	(Zhao et al., 2015)
4824	This study	10927	(Zhao et al., 2015)
8783	This study	10935	(Zhao et al., 2015)
8790	This study	10937	(Zhao et al., 2015)
3975	This study	10943	(Zhao et al., 2015)
10928	This study	10983	(Zhao et al., 2015)
10912	This study	3943	(Zhao et al., 2015)
3210	This study	P4489	(Zhao et al., 2015)
10923	This study		
10950	This study		
10951	This study		
10946	This study		
3211	This study		
10906	This study		
5910	This study		
4730	This study		
1133	This study		
4716	This study		

Table S6: Detecting structural variants with different callers from simulated data. Callers tested were DELLY, Lumpy, SVaba, and Pindel. Different sets are simulations with different numbers of structural variants.

								DELLY		Lumpy		SVaba		Pindel	
set	Deletion	Duplication	Inversion	Translocation	Insertion	Inv-del	Total number of SV	sensitivity	FDR	sensitivity	FDR	sensitivity	FDR	sensitivity	FDR
1	1	4	4	4	5	0	18	0.90	0	0.90	0.55	0.60	0.60	0.55	0.65
2	7	15	5	8	5	0	40	0.84	0.02	0.84	0.54	0.51	0.55	0.48	0.60
3	8	10	10	15	7	0	50	0.88	0	0.88	0.54	0.64	0.52	0.38	0.61
4	6	20	20	20	14	0	100	0.54	0.33	0.54	0.60	0.36	0.77	0.28	0.94

Table S7: Calling CNVs on simulated data using either CNVnator with two different bin sizes, CNV-seq, or both callers together.

	Sensitivity score	FDR score
CNVnator (1670 bin size)	0.375	0.556
CNVnator (75 bin size)	0.968	0.797
CNV-seq	0.937	0.999
Both callers	0.906	0.482
